# Supplementary material for: Protein composition analysis of human plasma-derived and recombinant human serum albumin preparations based on 4D label-free proteomics
Source: PeerJ. 2025 Jun 30;13:e19624. doi: 10.7717/peerj.19624 (PMC12225626; doi:10.7717/peerj.19624)
Supplement: Supplemental Information 2 — Supplemental Figures: Figure S1A: Peptide length, peptides per protein, distribution of coverage (%) and MW (kDa) of the LC-MS/MS analysis of rHSA from company A. Figure S2B: Peptide length, peptides per protein, distribution of coverage (%) and MW(kDa) of the LC-MS/MS analysis of rHSA from company B. Figure S3C: Peptide length, peptides per protein, distribution of coverage (%) and MW(kDa) of the LC-MS/MS analysis of pHSA from company C. Figure S4D: Peptide length, peptides per protein, distribution of coverage (%) and MW(kDa) of the LC-MS/MS analysis of pHSA from company D. Figure S5E: Peptide length, peptides per protein, distribution of coverage (%) and MW(kDa) of the LC-MS/MS analysis of pHSA from company E. Figure S6F: Peptide length, peptides per protein, distribution of coverage (%) and MW(kDa) of the LC-MS/MS analysis of pHSA from company F. Figure S7G: Peptide length, peptides per protein, distribution of coverage (%) and MW(kDa) of the LC-MS/MS analysis of pHSA from company G. Figure S8H: Peptide length, peptides per protein, distribution of coverage (%) and MW(kDa) of the LC-MS/MS analysis of pHSA from company H. Figure S9: GO enrichment analysis of the APs in pHSA. Figure S10: Subcellular localization prediction of the APs in pHSA. Figure S11: COG/KOG enrichment analysis of the APs in pHSA. Figure S12: KEGG pathway enrichment analysis of the APs in pHSA. Supplemental Tables: Table S1A: The protein and peptide identified in rHSA from company A. Table S2B: The protein and peptide identified in rHSA from company B. Table S3C: The protein and peptide identified in pHSA from company C. Table S4D: The protein and peptide identified in pHSA from company D. Table S5E: The protein and peptide identified in pHSA from company E. Table S6F: The protein and peptide identified in pHSA from company F. Table S7G: The protein and peptide identified in pHSA from company G. Table S8H: The protein and peptide identified in pHSA from company H. Table S9: The relative abunda [file peerj-13-19624-s002.zip › Supplementary/Supplementary File/Supplementary File1/2-Functional_classification/ident-KEGG_map_classify.pdf]

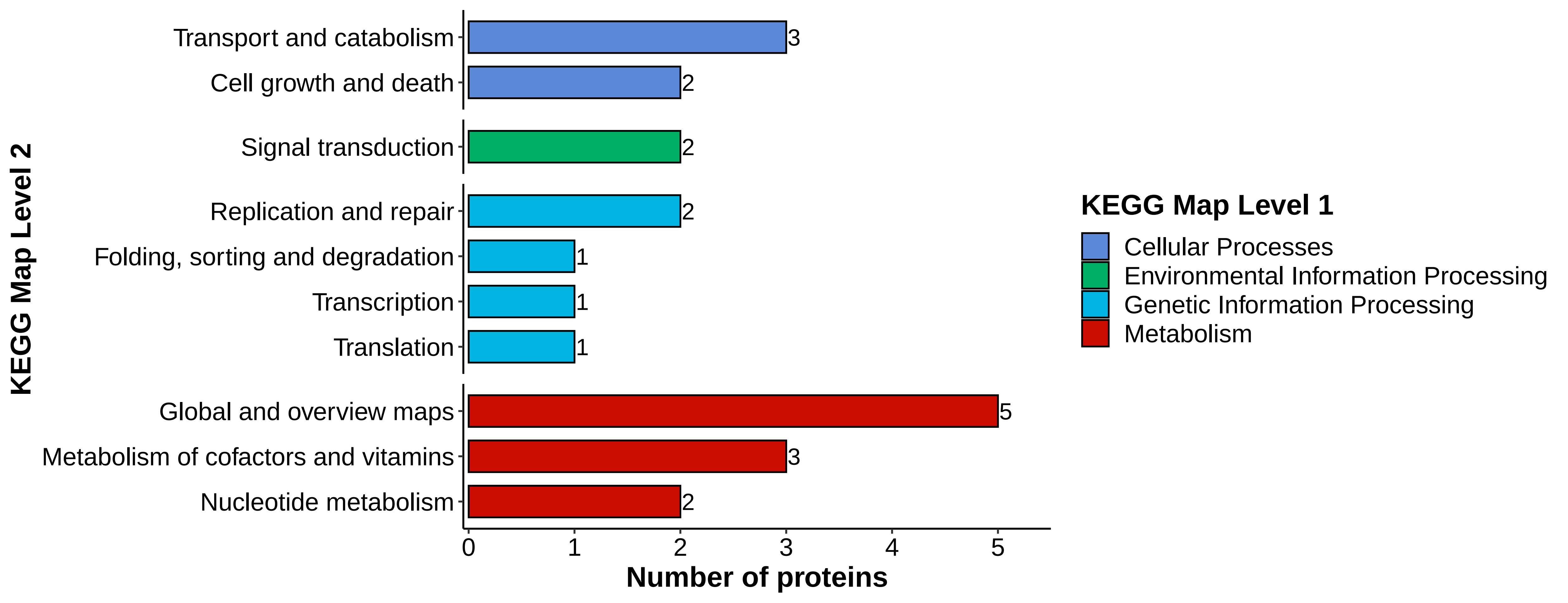

KEGG Map Level 2
Transport and catabolism
Cell growth and death
Signal transduction
Replication and repair
Folding, sorting and degradation
Transcription
Translation
Global and overview maps
Metabolism of cofactors and vitamins
Nucleotide metabolism
3
2
2
2
1
1
1
5
3
2
0
1
2
3
4
5
Number of proteins
KEGG Map Level 1
Cellular Processes
Environmental Information Processing
Genetic Information Processing
Metabolism
