# Supplementary material for: Protein composition analysis of human plasma-derived and recombinant human serum albumin preparations based on 4D label-free proteomics
Source: PeerJ. 2025 Jun 30;13:e19624. doi: 10.7717/peerj.19624 (PMC12225626; doi:10.7717/peerj.19624)
Supplement: Supplemental Information 2 — Supplemental Figures: Figure S1A: Peptide length, peptides per protein, distribution of coverage (%) and MW (kDa) of the LC-MS/MS analysis of rHSA from company A. Figure S2B: Peptide length, peptides per protein, distribution of coverage (%) and MW(kDa) of the LC-MS/MS analysis of rHSA from company B. Figure S3C: Peptide length, peptides per protein, distribution of coverage (%) and MW(kDa) of the LC-MS/MS analysis of pHSA from company C. Figure S4D: Peptide length, peptides per protein, distribution of coverage (%) and MW(kDa) of the LC-MS/MS analysis of pHSA from company D. Figure S5E: Peptide length, peptides per protein, distribution of coverage (%) and MW(kDa) of the LC-MS/MS analysis of pHSA from company E. Figure S6F: Peptide length, peptides per protein, distribution of coverage (%) and MW(kDa) of the LC-MS/MS analysis of pHSA from company F. Figure S7G: Peptide length, peptides per protein, distribution of coverage (%) and MW(kDa) of the LC-MS/MS analysis of pHSA from company G. Figure S8H: Peptide length, peptides per protein, distribution of coverage (%) and MW(kDa) of the LC-MS/MS analysis of pHSA from company H. Figure S9: GO enrichment analysis of the APs in pHSA. Figure S10: Subcellular localization prediction of the APs in pHSA. Figure S11: COG/KOG enrichment analysis of the APs in pHSA. Figure S12: KEGG pathway enrichment analysis of the APs in pHSA. Supplemental Tables: Table S1A: The protein and peptide identified in rHSA from company A. Table S2B: The protein and peptide identified in rHSA from company B. Table S3C: The protein and peptide identified in pHSA from company C. Table S4D: The protein and peptide identified in pHSA from company D. Table S5E: The protein and peptide identified in pHSA from company E. Table S6F: The protein and peptide identified in pHSA from company F. Table S7G: The protein and peptide identified in pHSA from company G. Table S8H: The protein and peptide identified in pHSA from company H. Table S9: The relative abunda [file peerj-13-19624-s002.zip › Supplementary/Supplementary File/Supplementary File1/3-Functional_enrichment/ident-BP_cirplot.pdf]

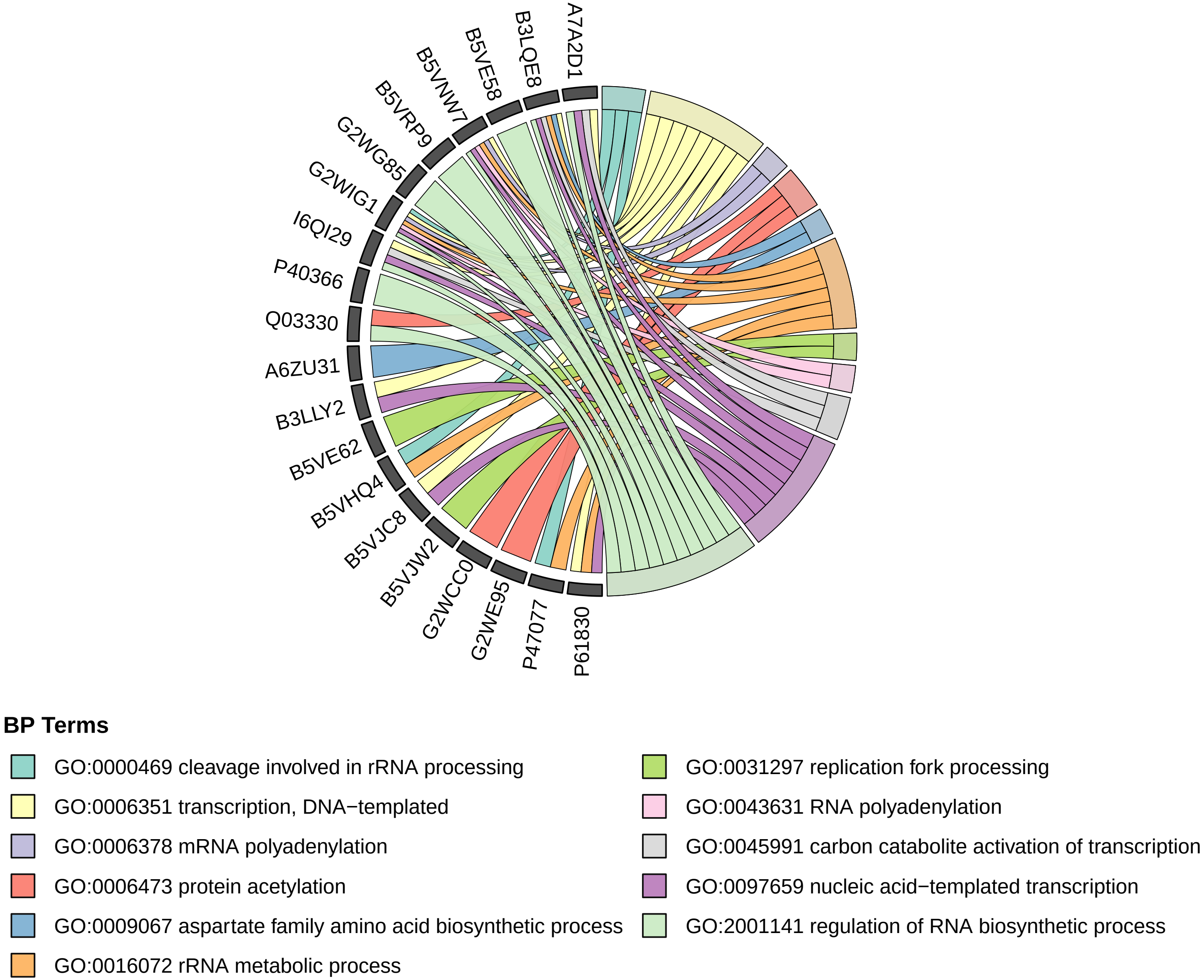
A7A2D1
B3LQE8
B5VE58
B5VNW7
B5VRP9
G2WG85
G2WIG1
I6QI29
P40366
Q03330
A6ZU31
B3LLY2
B5VE62
B5VHQ4
B5VJC8
B5VJW2
G2WCC0
G2WE95
P47077
P61830
BP Terms
GO:0000469 cleavage involved in rRNA processing
GO:0006351 transcription, DNA−templated
GO:0006378 mRNA polyadenylation
GO:0006473 protein acetylation
GO:0009067 aspartate family amino acid biosynthetic process
GO:0016072 rRNA metabolic process
GO:0031297 replication fork processing
GO:0043631 RNA polyadenylation
GO:0045991 carbon catabolite activation of transcription
GO:0097659 nucleic acid−templated transcription
GO:2001141 regulation of RNA biosynthetic process
