# Supplementary material for: Protein composition analysis of human plasma-derived and recombinant human serum albumin preparations based on 4D label-free proteomics
Source: PeerJ. 2025 Jun 30;13:e19624. doi: 10.7717/peerj.19624 (PMC12225626; doi:10.7717/peerj.19624)
Supplement: Supplemental Information 2 — Supplemental Figures: Figure S1A: Peptide length, peptides per protein, distribution of coverage (%) and MW (kDa) of the LC-MS/MS analysis of rHSA from company A. Figure S2B: Peptide length, peptides per protein, distribution of coverage (%) and MW(kDa) of the LC-MS/MS analysis of rHSA from company B. Figure S3C: Peptide length, peptides per protein, distribution of coverage (%) and MW(kDa) of the LC-MS/MS analysis of pHSA from company C. Figure S4D: Peptide length, peptides per protein, distribution of coverage (%) and MW(kDa) of the LC-MS/MS analysis of pHSA from company D. Figure S5E: Peptide length, peptides per protein, distribution of coverage (%) and MW(kDa) of the LC-MS/MS analysis of pHSA from company E. Figure S6F: Peptide length, peptides per protein, distribution of coverage (%) and MW(kDa) of the LC-MS/MS analysis of pHSA from company F. Figure S7G: Peptide length, peptides per protein, distribution of coverage (%) and MW(kDa) of the LC-MS/MS analysis of pHSA from company G. Figure S8H: Peptide length, peptides per protein, distribution of coverage (%) and MW(kDa) of the LC-MS/MS analysis of pHSA from company H. Figure S9: GO enrichment analysis of the APs in pHSA. Figure S10: Subcellular localization prediction of the APs in pHSA. Figure S11: COG/KOG enrichment analysis of the APs in pHSA. Figure S12: KEGG pathway enrichment analysis of the APs in pHSA. Supplemental Tables: Table S1A: The protein and peptide identified in rHSA from company A. Table S2B: The protein and peptide identified in rHSA from company B. Table S3C: The protein and peptide identified in pHSA from company C. Table S4D: The protein and peptide identified in pHSA from company D. Table S5E: The protein and peptide identified in pHSA from company E. Table S6F: The protein and peptide identified in pHSA from company F. Table S7G: The protein and peptide identified in pHSA from company G. Table S8H: The protein and peptide identified in pHSA from company H. Table S9: The relative abunda [file peerj-13-19624-s002.zip › Supplementary/Supplementary File/Supplementary File2/2-Functional_classification/ident-Subcell_classify.pdf]

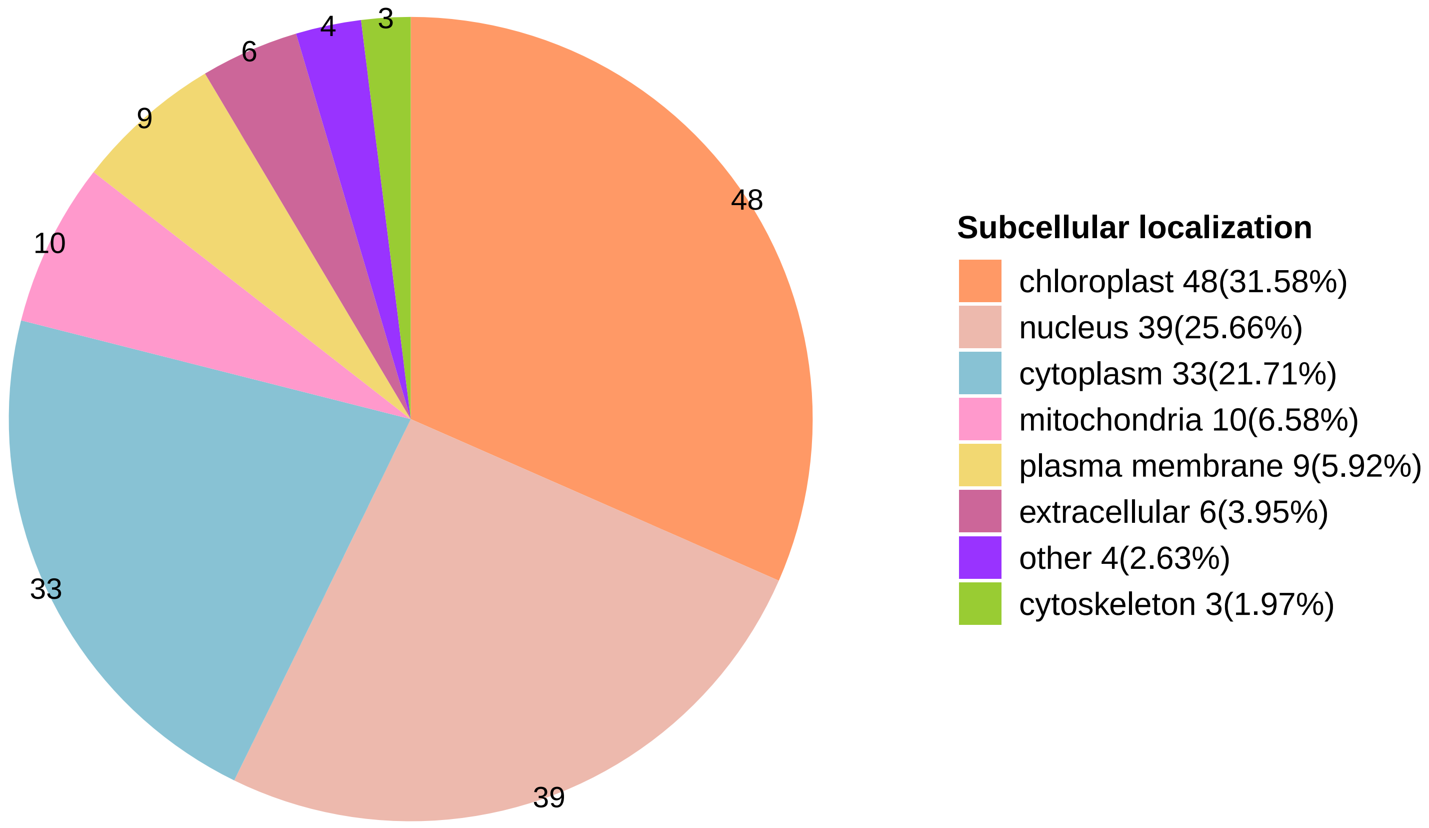
48
39
33
10
9
6
4
3
Subcellular localization
chloroplast 48(31.58%)
nucleus 39(25.66%)
cytoplasm 33(21.71%)
mitochondria 10(6.58%)
plasma membrane 9(5.92%)
extracellular 6(3.95%)
other 4(2.63%)
cytoskeleton 3(1.97%)
