# Supplementary figures and images for: Protein composition analysis of human plasma-derived and recombinant human serum albumin preparations based on 4D label-free proteomics
Source: PeerJ. 2025 Jun 30;13:e19624. doi: 10.7717/peerj.19624 (PMC12225626; doi:10.7717/peerj.19624)

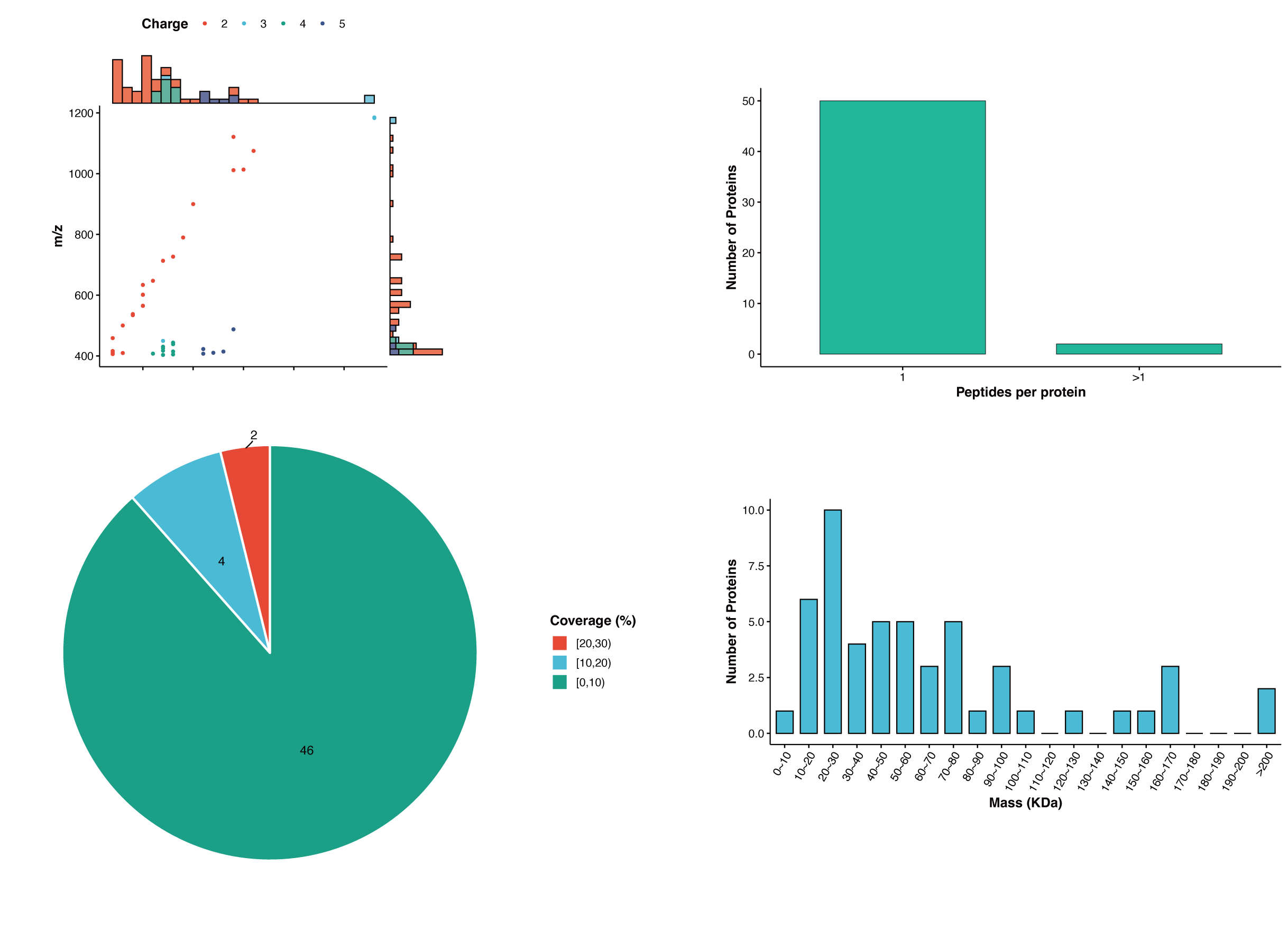

Supplement: Supplemental Information 2 — Supplemental Figures: Figure S1A: Peptide length, peptides per protein, distribution of coverage (%) and MW (kDa) of the LC-MS/MS analysis of rHSA from company A. Figure S2B: Peptide length, peptides per protein, distribution of coverage (%) and MW(kDa) of the LC-MS/MS analysis of rHSA from company B. Figure S3C: Peptide length, peptides per protein, distribution of coverage (%) and MW(kDa) of the LC-MS/MS analysis of pHSA from company C. Figure S4D: Peptide length, peptides per protein, distribution of coverage (%) and MW(kDa) of the LC-MS/MS analysis of pHSA from company D. Figure S5E: Peptide length, peptides per protein, distribution of coverage (%) and MW(kDa) of the LC-MS/MS analysis of pHSA from company E. Figure S6F: Peptide length, peptides per protein, distribution of coverage (%) and MW(kDa) of the LC-MS/MS analysis of pHSA from company F. Figure S7G: Peptide length, peptides per protein, distribution of coverage (%) and MW(kDa) of the LC-MS/MS analysis of pHSA from company G. Figure S8H: Peptide length, peptides per protein, distribution of coverage (%) and MW(kDa) of the LC-MS/MS analysis of pHSA from company H. Figure S9: GO enrichment analysis of the APs in pHSA. Figure S10: Subcellular localization prediction of the APs in pHSA. Figure S11: COG/KOG enrichment analysis of the APs in pHSA. Figure S12: KEGG pathway enrichment analysis of the APs in pHSA. Supplemental Tables: Table S1A: The protein and peptide identified in rHSA from company A. Table S2B: The protein and peptide identified in rHSA from company B. Table S3C: The protein and peptide identified in pHSA from company C. Table S4D: The protein and peptide identified in pHSA from company D. Table S5E: The protein and peptide identified in pHSA from company E. Table S6F: The protein and peptide identified in pHSA from company F. Table S7G: The protein and peptide identified in pHSA from company G. Table S8H: The protein and peptide identified in pHSA from company H. Table S9: The relative abunda [file peerj-13-19624-s002.zip › Supplementary/Supplementary Figure/Figure S1 A.tif]

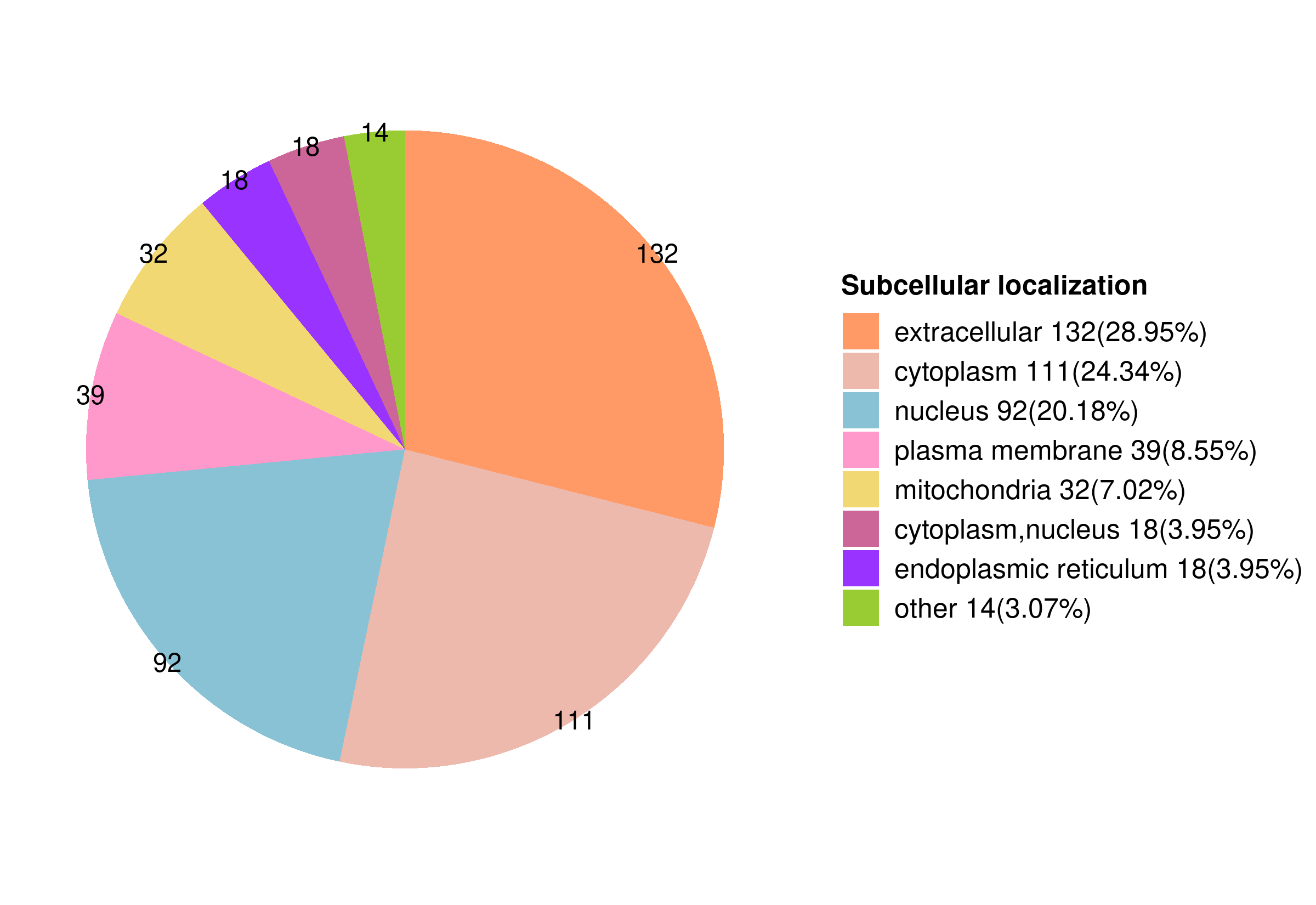

Supplement: Supplemental Information 2 — Supplemental Figures: Figure S1A: Peptide length, peptides per protein, distribution of coverage (%) and MW (kDa) of the LC-MS/MS analysis of rHSA from company A. Figure S2B: Peptide length, peptides per protein, distribution of coverage (%) and MW(kDa) of the LC-MS/MS analysis of rHSA from company B. Figure S3C: Peptide length, peptides per protein, distribution of coverage (%) and MW(kDa) of the LC-MS/MS analysis of pHSA from company C. Figure S4D: Peptide length, peptides per protein, distribution of coverage (%) and MW(kDa) of the LC-MS/MS analysis of pHSA from company D. Figure S5E: Peptide length, peptides per protein, distribution of coverage (%) and MW(kDa) of the LC-MS/MS analysis of pHSA from company E. Figure S6F: Peptide length, peptides per protein, distribution of coverage (%) and MW(kDa) of the LC-MS/MS analysis of pHSA from company F. Figure S7G: Peptide length, peptides per protein, distribution of coverage (%) and MW(kDa) of the LC-MS/MS analysis of pHSA from company G. Figure S8H: Peptide length, peptides per protein, distribution of coverage (%) and MW(kDa) of the LC-MS/MS analysis of pHSA from company H. Figure S9: GO enrichment analysis of the APs in pHSA. Figure S10: Subcellular localization prediction of the APs in pHSA. Figure S11: COG/KOG enrichment analysis of the APs in pHSA. Figure S12: KEGG pathway enrichment analysis of the APs in pHSA. Supplemental Tables: Table S1A: The protein and peptide identified in rHSA from company A. Table S2B: The protein and peptide identified in rHSA from company B. Table S3C: The protein and peptide identified in pHSA from company C. Table S4D: The protein and peptide identified in pHSA from company D. Table S5E: The protein and peptide identified in pHSA from company E. Table S6F: The protein and peptide identified in pHSA from company F. Table S7G: The protein and peptide identified in pHSA from company G. Table S8H: The protein and peptide identified in pHSA from company H. Table S9: The relative abunda [file peerj-13-19624-s002.zip › Supplementary/Supplementary Figure/Figure S10 ident-Subcell_classify.png]

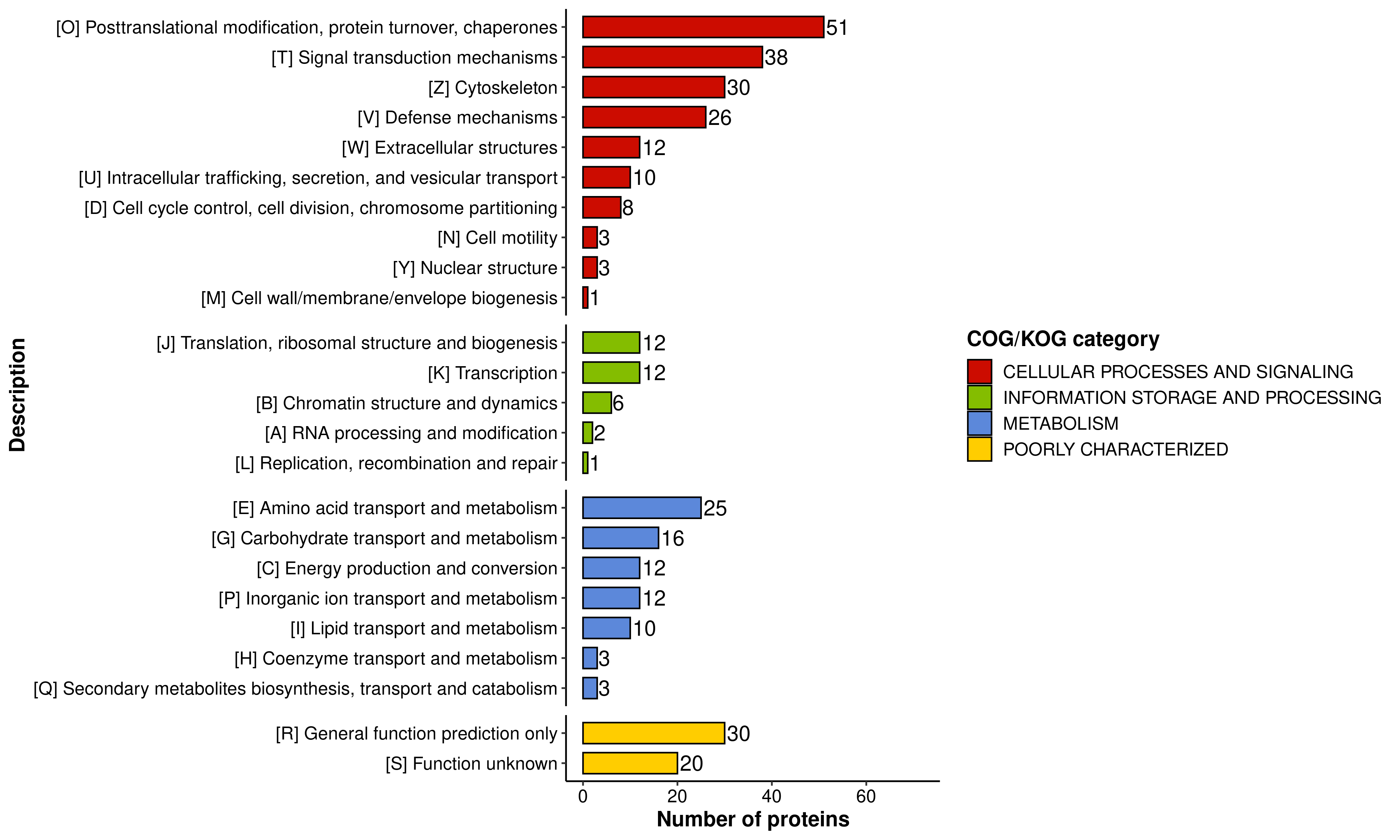

Supplement: Supplemental Information 2 — Supplemental Figures: Figure S1A: Peptide length, peptides per protein, distribution of coverage (%) and MW (kDa) of the LC-MS/MS analysis of rHSA from company A. Figure S2B: Peptide length, peptides per protein, distribution of coverage (%) and MW(kDa) of the LC-MS/MS analysis of rHSA from company B. Figure S3C: Peptide length, peptides per protein, distribution of coverage (%) and MW(kDa) of the LC-MS/MS analysis of pHSA from company C. Figure S4D: Peptide length, peptides per protein, distribution of coverage (%) and MW(kDa) of the LC-MS/MS analysis of pHSA from company D. Figure S5E: Peptide length, peptides per protein, distribution of coverage (%) and MW(kDa) of the LC-MS/MS analysis of pHSA from company E. Figure S6F: Peptide length, peptides per protein, distribution of coverage (%) and MW(kDa) of the LC-MS/MS analysis of pHSA from company F. Figure S7G: Peptide length, peptides per protein, distribution of coverage (%) and MW(kDa) of the LC-MS/MS analysis of pHSA from company G. Figure S8H: Peptide length, peptides per protein, distribution of coverage (%) and MW(kDa) of the LC-MS/MS analysis of pHSA from company H. Figure S9: GO enrichment analysis of the APs in pHSA. Figure S10: Subcellular localization prediction of the APs in pHSA. Figure S11: COG/KOG enrichment analysis of the APs in pHSA. Figure S12: KEGG pathway enrichment analysis of the APs in pHSA. Supplemental Tables: Table S1A: The protein and peptide identified in rHSA from company A. Table S2B: The protein and peptide identified in rHSA from company B. Table S3C: The protein and peptide identified in pHSA from company C. Table S4D: The protein and peptide identified in pHSA from company D. Table S5E: The protein and peptide identified in pHSA from company E. Table S6F: The protein and peptide identified in pHSA from company F. Table S7G: The protein and peptide identified in pHSA from company G. Table S8H: The protein and peptide identified in pHSA from company H. Table S9: The relative abunda [file peerj-13-19624-s002.zip › Supplementary/Supplementary Figure/Figure S11 ident-COG_classify.png]

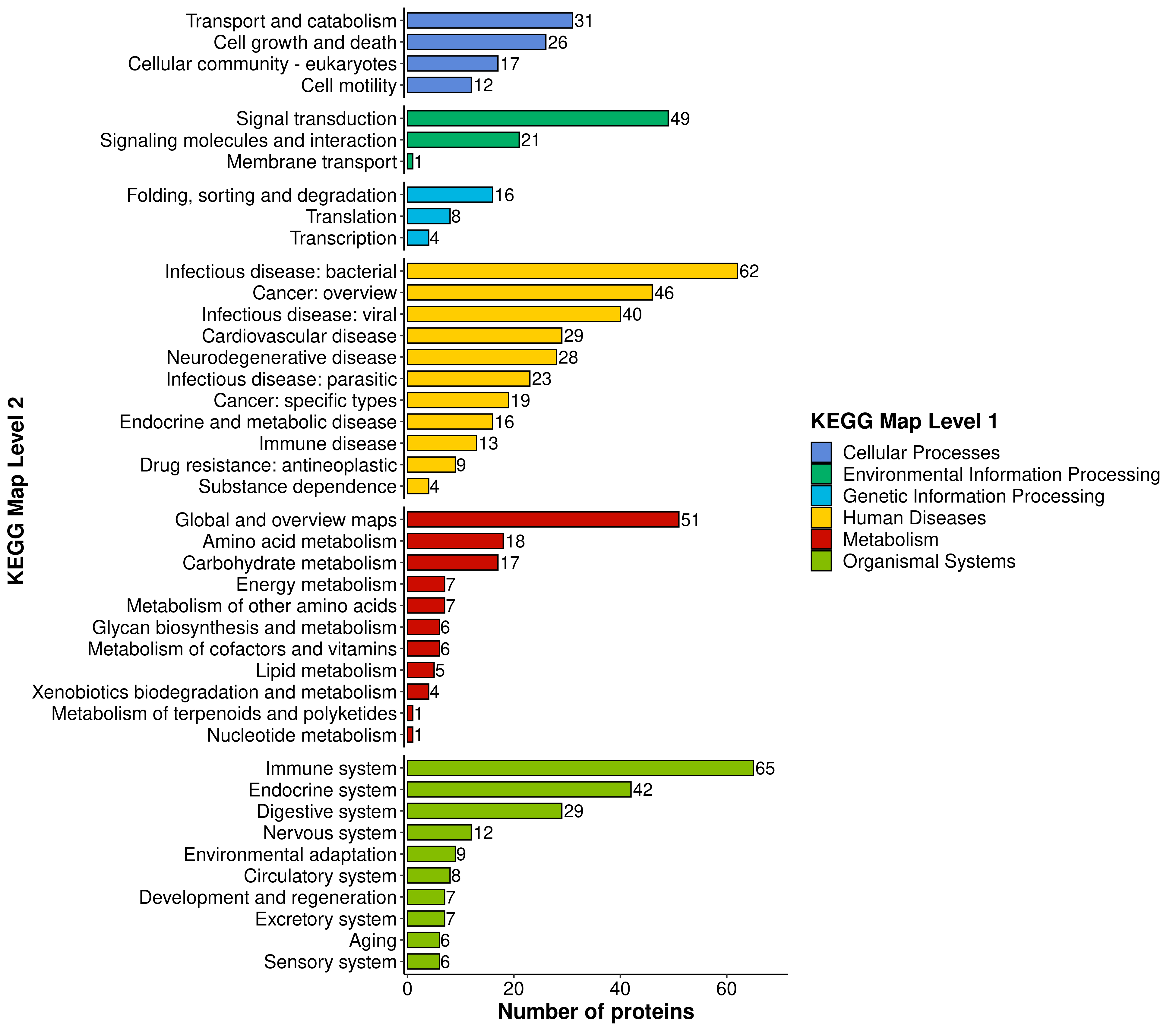

Supplement: Supplemental Information 2 — Supplemental Figures: Figure S1A: Peptide length, peptides per protein, distribution of coverage (%) and MW (kDa) of the LC-MS/MS analysis of rHSA from company A. Figure S2B: Peptide length, peptides per protein, distribution of coverage (%) and MW(kDa) of the LC-MS/MS analysis of rHSA from company B. Figure S3C: Peptide length, peptides per protein, distribution of coverage (%) and MW(kDa) of the LC-MS/MS analysis of pHSA from company C. Figure S4D: Peptide length, peptides per protein, distribution of coverage (%) and MW(kDa) of the LC-MS/MS analysis of pHSA from company D. Figure S5E: Peptide length, peptides per protein, distribution of coverage (%) and MW(kDa) of the LC-MS/MS analysis of pHSA from company E. Figure S6F: Peptide length, peptides per protein, distribution of coverage (%) and MW(kDa) of the LC-MS/MS analysis of pHSA from company F. Figure S7G: Peptide length, peptides per protein, distribution of coverage (%) and MW(kDa) of the LC-MS/MS analysis of pHSA from company G. Figure S8H: Peptide length, peptides per protein, distribution of coverage (%) and MW(kDa) of the LC-MS/MS analysis of pHSA from company H. Figure S9: GO enrichment analysis of the APs in pHSA. Figure S10: Subcellular localization prediction of the APs in pHSA. Figure S11: COG/KOG enrichment analysis of the APs in pHSA. Figure S12: KEGG pathway enrichment analysis of the APs in pHSA. Supplemental Tables: Table S1A: The protein and peptide identified in rHSA from company A. Table S2B: The protein and peptide identified in rHSA from company B. Table S3C: The protein and peptide identified in pHSA from company C. Table S4D: The protein and peptide identified in pHSA from company D. Table S5E: The protein and peptide identified in pHSA from company E. Table S6F: The protein and peptide identified in pHSA from company F. Table S7G: The protein and peptide identified in pHSA from company G. Table S8H: The protein and peptide identified in pHSA from company H. Table S9: The relative abunda [file peerj-13-19624-s002.zip › Supplementary/Supplementary Figure/Figure S12 ident-KEGG_map_classify.png]

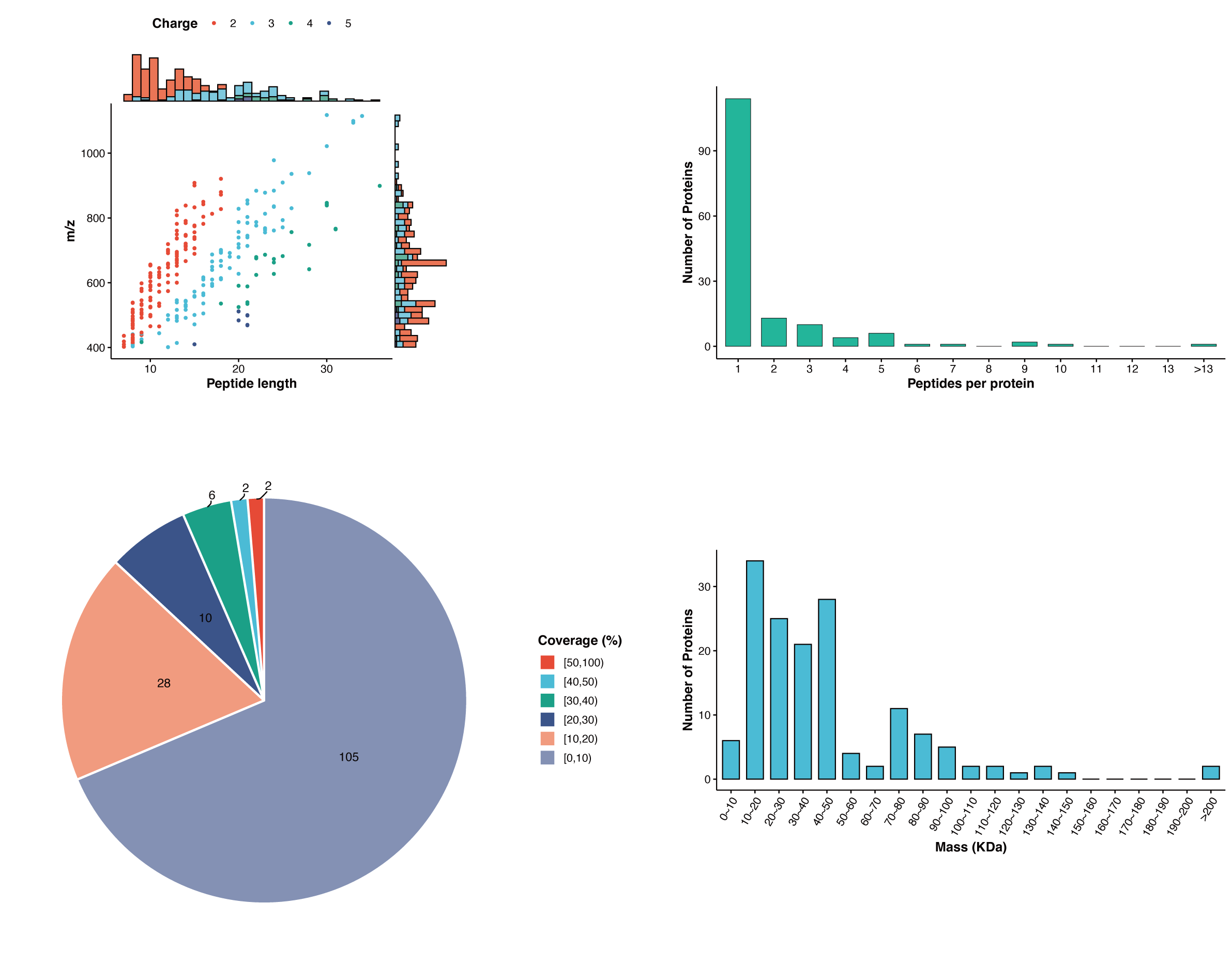

Supplement: Supplemental Information 2 — Supplemental Figures: Figure S1A: Peptide length, peptides per protein, distribution of coverage (%) and MW (kDa) of the LC-MS/MS analysis of rHSA from company A. Figure S2B: Peptide length, peptides per protein, distribution of coverage (%) and MW(kDa) of the LC-MS/MS analysis of rHSA from company B. Figure S3C: Peptide length, peptides per protein, distribution of coverage (%) and MW(kDa) of the LC-MS/MS analysis of pHSA from company C. Figure S4D: Peptide length, peptides per protein, distribution of coverage (%) and MW(kDa) of the LC-MS/MS analysis of pHSA from company D. Figure S5E: Peptide length, peptides per protein, distribution of coverage (%) and MW(kDa) of the LC-MS/MS analysis of pHSA from company E. Figure S6F: Peptide length, peptides per protein, distribution of coverage (%) and MW(kDa) of the LC-MS/MS analysis of pHSA from company F. Figure S7G: Peptide length, peptides per protein, distribution of coverage (%) and MW(kDa) of the LC-MS/MS analysis of pHSA from company G. Figure S8H: Peptide length, peptides per protein, distribution of coverage (%) and MW(kDa) of the LC-MS/MS analysis of pHSA from company H. Figure S9: GO enrichment analysis of the APs in pHSA. Figure S10: Subcellular localization prediction of the APs in pHSA. Figure S11: COG/KOG enrichment analysis of the APs in pHSA. Figure S12: KEGG pathway enrichment analysis of the APs in pHSA. Supplemental Tables: Table S1A: The protein and peptide identified in rHSA from company A. Table S2B: The protein and peptide identified in rHSA from company B. Table S3C: The protein and peptide identified in pHSA from company C. Table S4D: The protein and peptide identified in pHSA from company D. Table S5E: The protein and peptide identified in pHSA from company E. Table S6F: The protein and peptide identified in pHSA from company F. Table S7G: The protein and peptide identified in pHSA from company G. Table S8H: The protein and peptide identified in pHSA from company H. Table S9: The relative abunda [file peerj-13-19624-s002.zip › Supplementary/Supplementary Figure/Figure S2 B.tif]

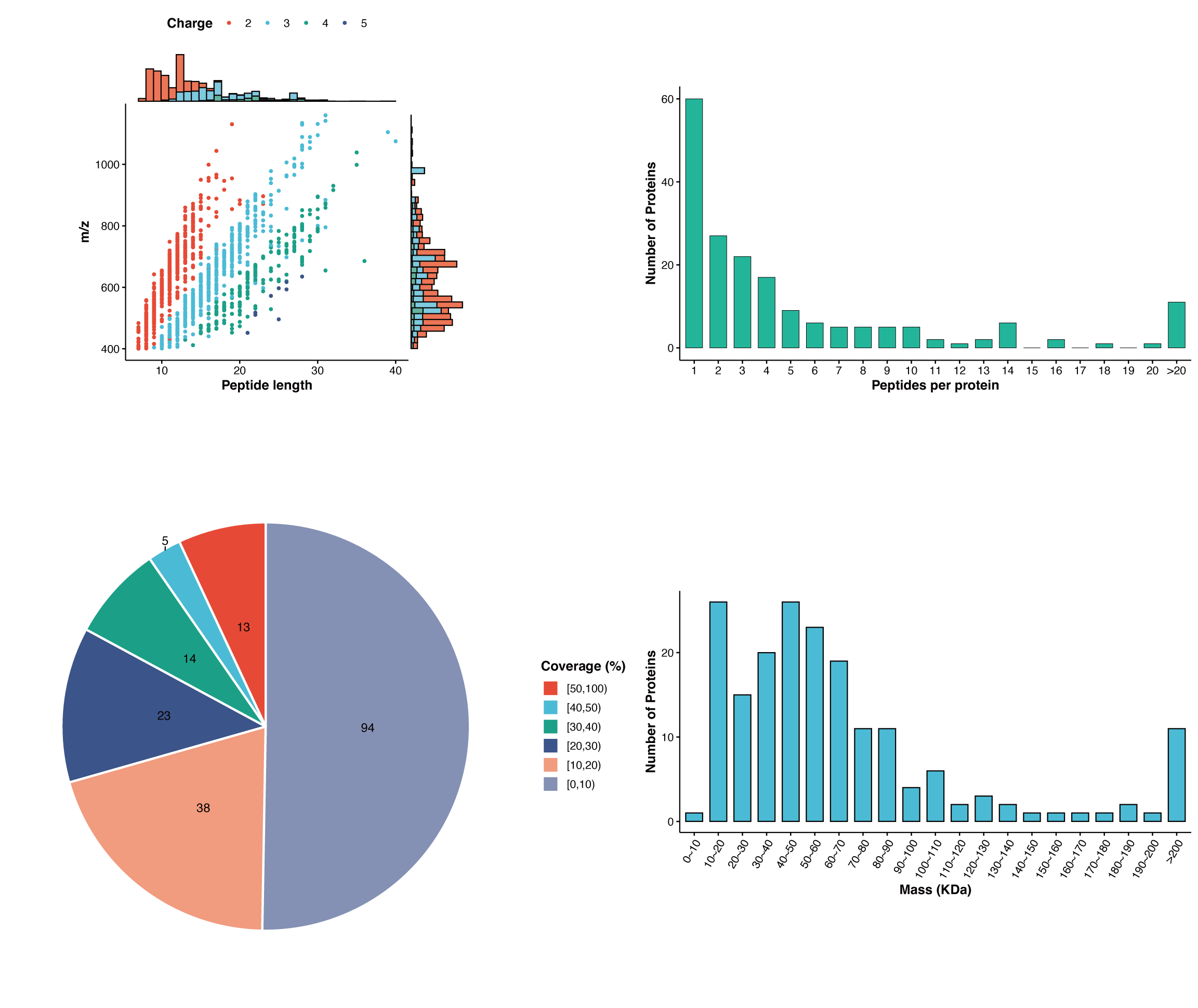

Supplement: Supplemental Information 2 — Supplemental Figures: Figure S1A: Peptide length, peptides per protein, distribution of coverage (%) and MW (kDa) of the LC-MS/MS analysis of rHSA from company A. Figure S2B: Peptide length, peptides per protein, distribution of coverage (%) and MW(kDa) of the LC-MS/MS analysis of rHSA from company B. Figure S3C: Peptide length, peptides per protein, distribution of coverage (%) and MW(kDa) of the LC-MS/MS analysis of pHSA from company C. Figure S4D: Peptide length, peptides per protein, distribution of coverage (%) and MW(kDa) of the LC-MS/MS analysis of pHSA from company D. Figure S5E: Peptide length, peptides per protein, distribution of coverage (%) and MW(kDa) of the LC-MS/MS analysis of pHSA from company E. Figure S6F: Peptide length, peptides per protein, distribution of coverage (%) and MW(kDa) of the LC-MS/MS analysis of pHSA from company F. Figure S7G: Peptide length, peptides per protein, distribution of coverage (%) and MW(kDa) of the LC-MS/MS analysis of pHSA from company G. Figure S8H: Peptide length, peptides per protein, distribution of coverage (%) and MW(kDa) of the LC-MS/MS analysis of pHSA from company H. Figure S9: GO enrichment analysis of the APs in pHSA. Figure S10: Subcellular localization prediction of the APs in pHSA. Figure S11: COG/KOG enrichment analysis of the APs in pHSA. Figure S12: KEGG pathway enrichment analysis of the APs in pHSA. Supplemental Tables: Table S1A: The protein and peptide identified in rHSA from company A. Table S2B: The protein and peptide identified in rHSA from company B. Table S3C: The protein and peptide identified in pHSA from company C. Table S4D: The protein and peptide identified in pHSA from company D. Table S5E: The protein and peptide identified in pHSA from company E. Table S6F: The protein and peptide identified in pHSA from company F. Table S7G: The protein and peptide identified in pHSA from company G. Table S8H: The protein and peptide identified in pHSA from company H. Table S9: The relative abunda [file peerj-13-19624-s002.zip › Supplementary/Supplementary Figure/Figure S3 C.tif]

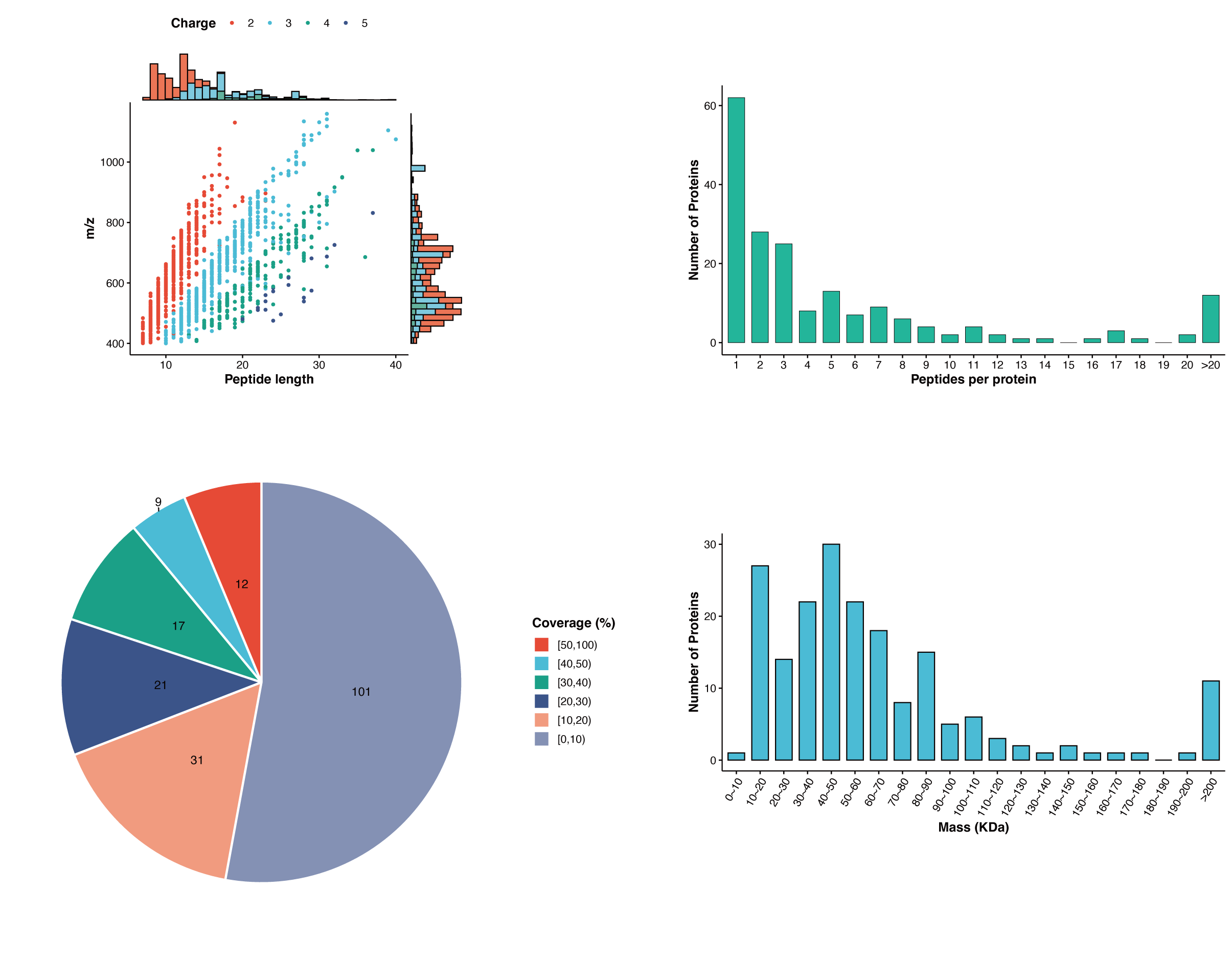

Supplement: Supplemental Information 2 — Supplemental Figures: Figure S1A: Peptide length, peptides per protein, distribution of coverage (%) and MW (kDa) of the LC-MS/MS analysis of rHSA from company A. Figure S2B: Peptide length, peptides per protein, distribution of coverage (%) and MW(kDa) of the LC-MS/MS analysis of rHSA from company B. Figure S3C: Peptide length, peptides per protein, distribution of coverage (%) and MW(kDa) of the LC-MS/MS analysis of pHSA from company C. Figure S4D: Peptide length, peptides per protein, distribution of coverage (%) and MW(kDa) of the LC-MS/MS analysis of pHSA from company D. Figure S5E: Peptide length, peptides per protein, distribution of coverage (%) and MW(kDa) of the LC-MS/MS analysis of pHSA from company E. Figure S6F: Peptide length, peptides per protein, distribution of coverage (%) and MW(kDa) of the LC-MS/MS analysis of pHSA from company F. Figure S7G: Peptide length, peptides per protein, distribution of coverage (%) and MW(kDa) of the LC-MS/MS analysis of pHSA from company G. Figure S8H: Peptide length, peptides per protein, distribution of coverage (%) and MW(kDa) of the LC-MS/MS analysis of pHSA from company H. Figure S9: GO enrichment analysis of the APs in pHSA. Figure S10: Subcellular localization prediction of the APs in pHSA. Figure S11: COG/KOG enrichment analysis of the APs in pHSA. Figure S12: KEGG pathway enrichment analysis of the APs in pHSA. Supplemental Tables: Table S1A: The protein and peptide identified in rHSA from company A. Table S2B: The protein and peptide identified in rHSA from company B. Table S3C: The protein and peptide identified in pHSA from company C. Table S4D: The protein and peptide identified in pHSA from company D. Table S5E: The protein and peptide identified in pHSA from company E. Table S6F: The protein and peptide identified in pHSA from company F. Table S7G: The protein and peptide identified in pHSA from company G. Table S8H: The protein and peptide identified in pHSA from company H. Table S9: The relative abunda [file peerj-13-19624-s002.zip › Supplementary/Supplementary Figure/Figure S4 D.tif]

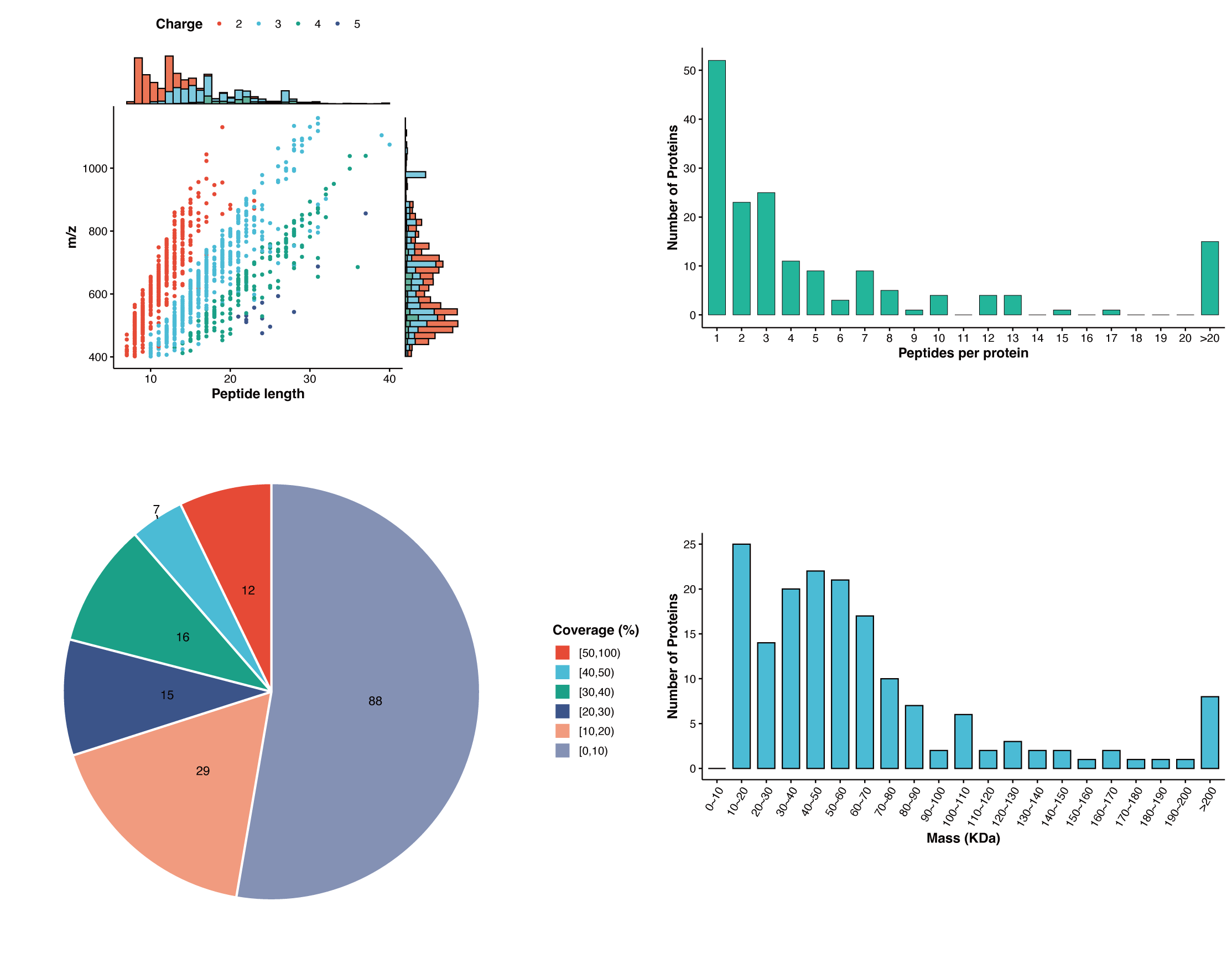

Supplement: Supplemental Information 2 — Supplemental Figures: Figure S1A: Peptide length, peptides per protein, distribution of coverage (%) and MW (kDa) of the LC-MS/MS analysis of rHSA from company A. Figure S2B: Peptide length, peptides per protein, distribution of coverage (%) and MW(kDa) of the LC-MS/MS analysis of rHSA from company B. Figure S3C: Peptide length, peptides per protein, distribution of coverage (%) and MW(kDa) of the LC-MS/MS analysis of pHSA from company C. Figure S4D: Peptide length, peptides per protein, distribution of coverage (%) and MW(kDa) of the LC-MS/MS analysis of pHSA from company D. Figure S5E: Peptide length, peptides per protein, distribution of coverage (%) and MW(kDa) of the LC-MS/MS analysis of pHSA from company E. Figure S6F: Peptide length, peptides per protein, distribution of coverage (%) and MW(kDa) of the LC-MS/MS analysis of pHSA from company F. Figure S7G: Peptide length, peptides per protein, distribution of coverage (%) and MW(kDa) of the LC-MS/MS analysis of pHSA from company G. Figure S8H: Peptide length, peptides per protein, distribution of coverage (%) and MW(kDa) of the LC-MS/MS analysis of pHSA from company H. Figure S9: GO enrichment analysis of the APs in pHSA. Figure S10: Subcellular localization prediction of the APs in pHSA. Figure S11: COG/KOG enrichment analysis of the APs in pHSA. Figure S12: KEGG pathway enrichment analysis of the APs in pHSA. Supplemental Tables: Table S1A: The protein and peptide identified in rHSA from company A. Table S2B: The protein and peptide identified in rHSA from company B. Table S3C: The protein and peptide identified in pHSA from company C. Table S4D: The protein and peptide identified in pHSA from company D. Table S5E: The protein and peptide identified in pHSA from company E. Table S6F: The protein and peptide identified in pHSA from company F. Table S7G: The protein and peptide identified in pHSA from company G. Table S8H: The protein and peptide identified in pHSA from company H. Table S9: The relative abunda [file peerj-13-19624-s002.zip › Supplementary/Supplementary Figure/Figure S5 E.tif]

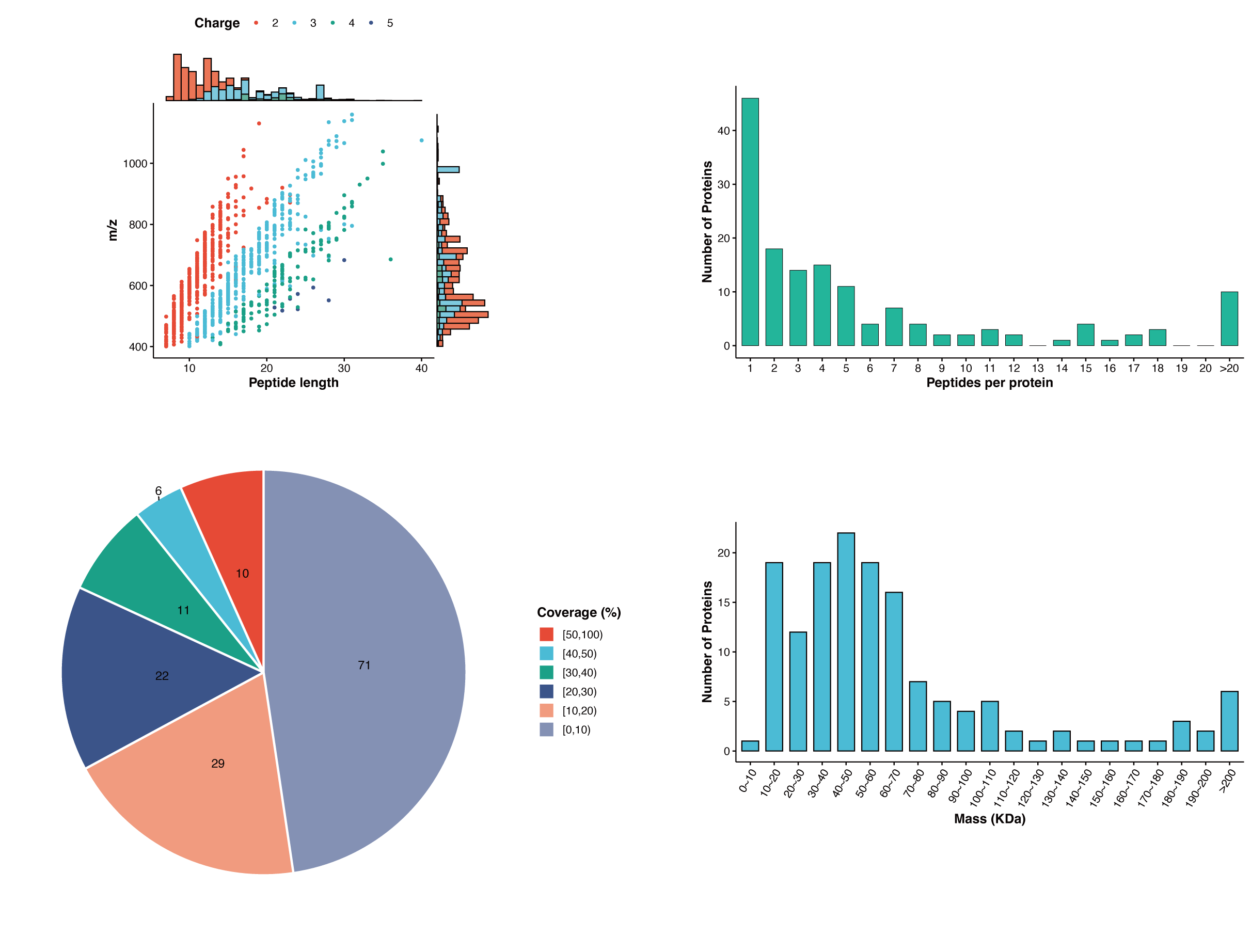

Supplement: Supplemental Information 2 — Supplemental Figures: Figure S1A: Peptide length, peptides per protein, distribution of coverage (%) and MW (kDa) of the LC-MS/MS analysis of rHSA from company A. Figure S2B: Peptide length, peptides per protein, distribution of coverage (%) and MW(kDa) of the LC-MS/MS analysis of rHSA from company B. Figure S3C: Peptide length, peptides per protein, distribution of coverage (%) and MW(kDa) of the LC-MS/MS analysis of pHSA from company C. Figure S4D: Peptide length, peptides per protein, distribution of coverage (%) and MW(kDa) of the LC-MS/MS analysis of pHSA from company D. Figure S5E: Peptide length, peptides per protein, distribution of coverage (%) and MW(kDa) of the LC-MS/MS analysis of pHSA from company E. Figure S6F: Peptide length, peptides per protein, distribution of coverage (%) and MW(kDa) of the LC-MS/MS analysis of pHSA from company F. Figure S7G: Peptide length, peptides per protein, distribution of coverage (%) and MW(kDa) of the LC-MS/MS analysis of pHSA from company G. Figure S8H: Peptide length, peptides per protein, distribution of coverage (%) and MW(kDa) of the LC-MS/MS analysis of pHSA from company H. Figure S9: GO enrichment analysis of the APs in pHSA. Figure S10: Subcellular localization prediction of the APs in pHSA. Figure S11: COG/KOG enrichment analysis of the APs in pHSA. Figure S12: KEGG pathway enrichment analysis of the APs in pHSA. Supplemental Tables: Table S1A: The protein and peptide identified in rHSA from company A. Table S2B: The protein and peptide identified in rHSA from company B. Table S3C: The protein and peptide identified in pHSA from company C. Table S4D: The protein and peptide identified in pHSA from company D. Table S5E: The protein and peptide identified in pHSA from company E. Table S6F: The protein and peptide identified in pHSA from company F. Table S7G: The protein and peptide identified in pHSA from company G. Table S8H: The protein and peptide identified in pHSA from company H. Table S9: The relative abunda [file peerj-13-19624-s002.zip › Supplementary/Supplementary Figure/Figure S6 F.tif]

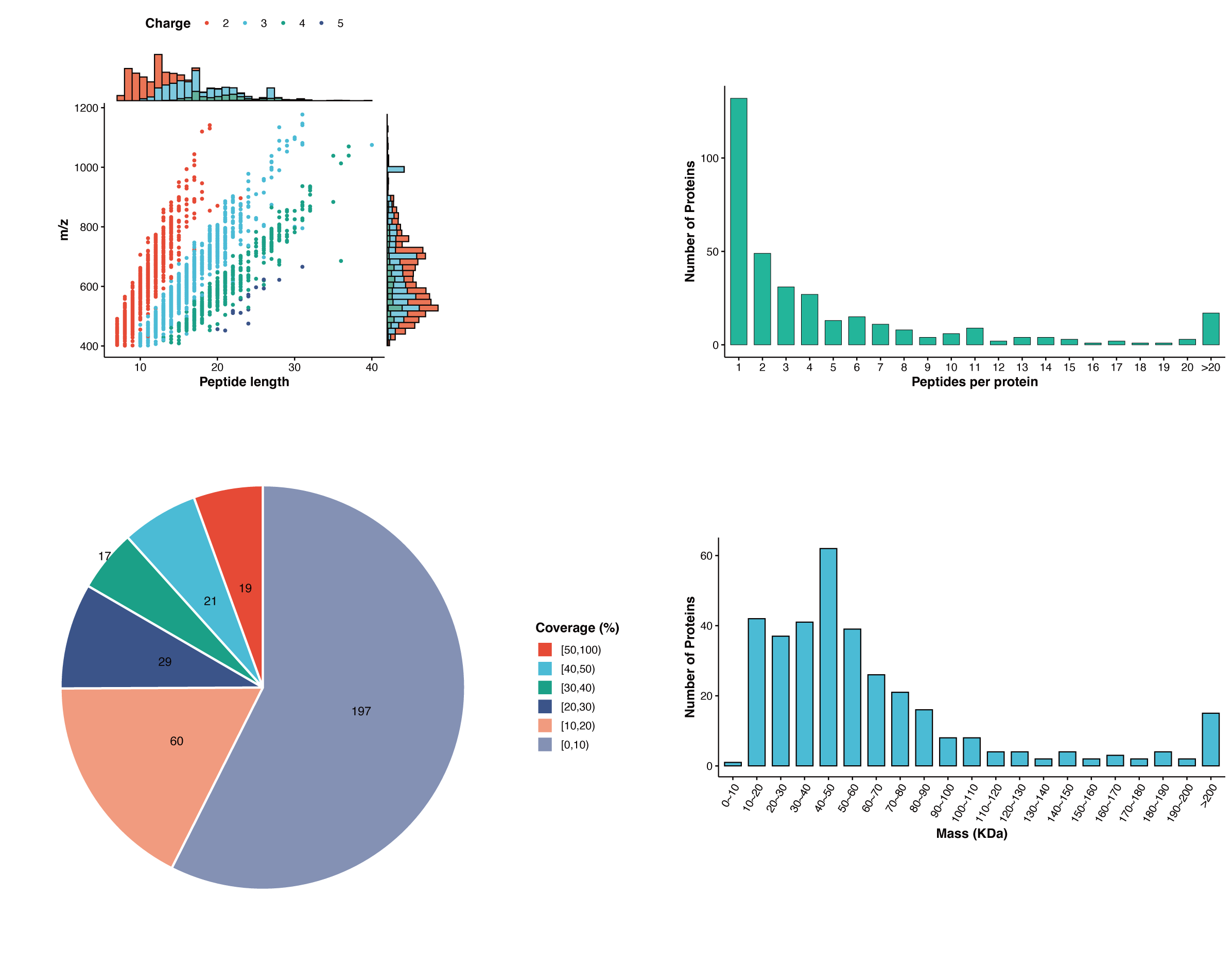

Supplement: Supplemental Information 2 — Supplemental Figures: Figure S1A: Peptide length, peptides per protein, distribution of coverage (%) and MW (kDa) of the LC-MS/MS analysis of rHSA from company A. Figure S2B: Peptide length, peptides per protein, distribution of coverage (%) and MW(kDa) of the LC-MS/MS analysis of rHSA from company B. Figure S3C: Peptide length, peptides per protein, distribution of coverage (%) and MW(kDa) of the LC-MS/MS analysis of pHSA from company C. Figure S4D: Peptide length, peptides per protein, distribution of coverage (%) and MW(kDa) of the LC-MS/MS analysis of pHSA from company D. Figure S5E: Peptide length, peptides per protein, distribution of coverage (%) and MW(kDa) of the LC-MS/MS analysis of pHSA from company E. Figure S6F: Peptide length, peptides per protein, distribution of coverage (%) and MW(kDa) of the LC-MS/MS analysis of pHSA from company F. Figure S7G: Peptide length, peptides per protein, distribution of coverage (%) and MW(kDa) of the LC-MS/MS analysis of pHSA from company G. Figure S8H: Peptide length, peptides per protein, distribution of coverage (%) and MW(kDa) of the LC-MS/MS analysis of pHSA from company H. Figure S9: GO enrichment analysis of the APs in pHSA. Figure S10: Subcellular localization prediction of the APs in pHSA. Figure S11: COG/KOG enrichment analysis of the APs in pHSA. Figure S12: KEGG pathway enrichment analysis of the APs in pHSA. Supplemental Tables: Table S1A: The protein and peptide identified in rHSA from company A. Table S2B: The protein and peptide identified in rHSA from company B. Table S3C: The protein and peptide identified in pHSA from company C. Table S4D: The protein and peptide identified in pHSA from company D. Table S5E: The protein and peptide identified in pHSA from company E. Table S6F: The protein and peptide identified in pHSA from company F. Table S7G: The protein and peptide identified in pHSA from company G. Table S8H: The protein and peptide identified in pHSA from company H. Table S9: The relative abunda [file peerj-13-19624-s002.zip › Supplementary/Supplementary Figure/Figure S7 G.tif]

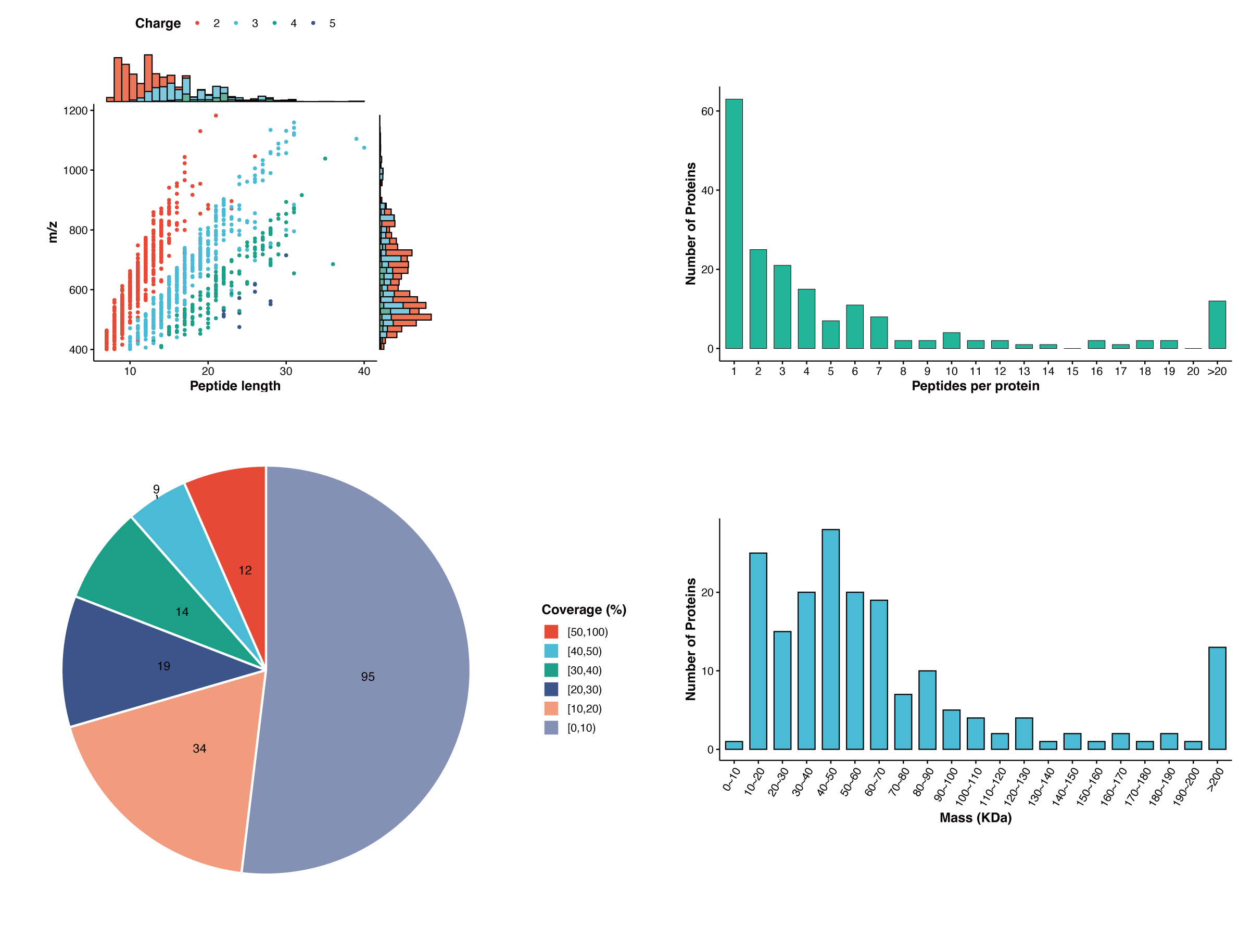

Supplement: Supplemental Information 2 — Supplemental Figures: Figure S1A: Peptide length, peptides per protein, distribution of coverage (%) and MW (kDa) of the LC-MS/MS analysis of rHSA from company A. Figure S2B: Peptide length, peptides per protein, distribution of coverage (%) and MW(kDa) of the LC-MS/MS analysis of rHSA from company B. Figure S3C: Peptide length, peptides per protein, distribution of coverage (%) and MW(kDa) of the LC-MS/MS analysis of pHSA from company C. Figure S4D: Peptide length, peptides per protein, distribution of coverage (%) and MW(kDa) of the LC-MS/MS analysis of pHSA from company D. Figure S5E: Peptide length, peptides per protein, distribution of coverage (%) and MW(kDa) of the LC-MS/MS analysis of pHSA from company E. Figure S6F: Peptide length, peptides per protein, distribution of coverage (%) and MW(kDa) of the LC-MS/MS analysis of pHSA from company F. Figure S7G: Peptide length, peptides per protein, distribution of coverage (%) and MW(kDa) of the LC-MS/MS analysis of pHSA from company G. Figure S8H: Peptide length, peptides per protein, distribution of coverage (%) and MW(kDa) of the LC-MS/MS analysis of pHSA from company H. Figure S9: GO enrichment analysis of the APs in pHSA. Figure S10: Subcellular localization prediction of the APs in pHSA. Figure S11: COG/KOG enrichment analysis of the APs in pHSA. Figure S12: KEGG pathway enrichment analysis of the APs in pHSA. Supplemental Tables: Table S1A: The protein and peptide identified in rHSA from company A. Table S2B: The protein and peptide identified in rHSA from company B. Table S3C: The protein and peptide identified in pHSA from company C. Table S4D: The protein and peptide identified in pHSA from company D. Table S5E: The protein and peptide identified in pHSA from company E. Table S6F: The protein and peptide identified in pHSA from company F. Table S7G: The protein and peptide identified in pHSA from company G. Table S8H: The protein and peptide identified in pHSA from company H. Table S9: The relative abunda [file peerj-13-19624-s002.zip › Supplementary/Supplementary Figure/Figure S8 H.tif]

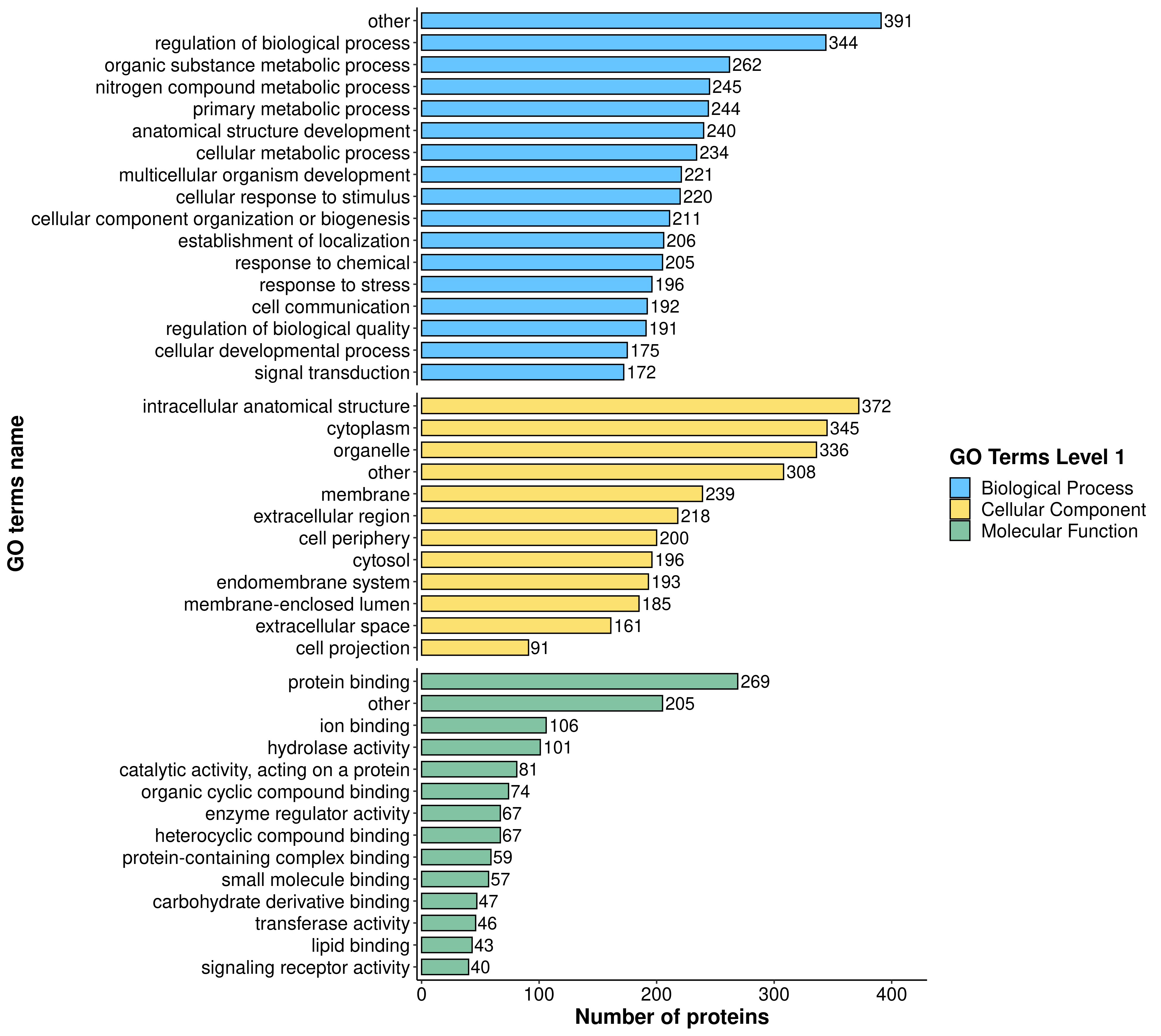

Supplement: Supplemental Information 2 — Supplemental Figures: Figure S1A: Peptide length, peptides per protein, distribution of coverage (%) and MW (kDa) of the LC-MS/MS analysis of rHSA from company A. Figure S2B: Peptide length, peptides per protein, distribution of coverage (%) and MW(kDa) of the LC-MS/MS analysis of rHSA from company B. Figure S3C: Peptide length, peptides per protein, distribution of coverage (%) and MW(kDa) of the LC-MS/MS analysis of pHSA from company C. Figure S4D: Peptide length, peptides per protein, distribution of coverage (%) and MW(kDa) of the LC-MS/MS analysis of pHSA from company D. Figure S5E: Peptide length, peptides per protein, distribution of coverage (%) and MW(kDa) of the LC-MS/MS analysis of pHSA from company E. Figure S6F: Peptide length, peptides per protein, distribution of coverage (%) and MW(kDa) of the LC-MS/MS analysis of pHSA from company F. Figure S7G: Peptide length, peptides per protein, distribution of coverage (%) and MW(kDa) of the LC-MS/MS analysis of pHSA from company G. Figure S8H: Peptide length, peptides per protein, distribution of coverage (%) and MW(kDa) of the LC-MS/MS analysis of pHSA from company H. Figure S9: GO enrichment analysis of the APs in pHSA. Figure S10: Subcellular localization prediction of the APs in pHSA. Figure S11: COG/KOG enrichment analysis of the APs in pHSA. Figure S12: KEGG pathway enrichment analysis of the APs in pHSA. Supplemental Tables: Table S1A: The protein and peptide identified in rHSA from company A. Table S2B: The protein and peptide identified in rHSA from company B. Table S3C: The protein and peptide identified in pHSA from company C. Table S4D: The protein and peptide identified in pHSA from company D. Table S5E: The protein and peptide identified in pHSA from company E. Table S6F: The protein and peptide identified in pHSA from company F. Table S7G: The protein and peptide identified in pHSA from company G. Table S8H: The protein and peptide identified in pHSA from company H. Table S9: The relative abunda [file peerj-13-19624-s002.zip › Supplementary/Supplementary Figure/Figure S9 ident-GO_classify.png]

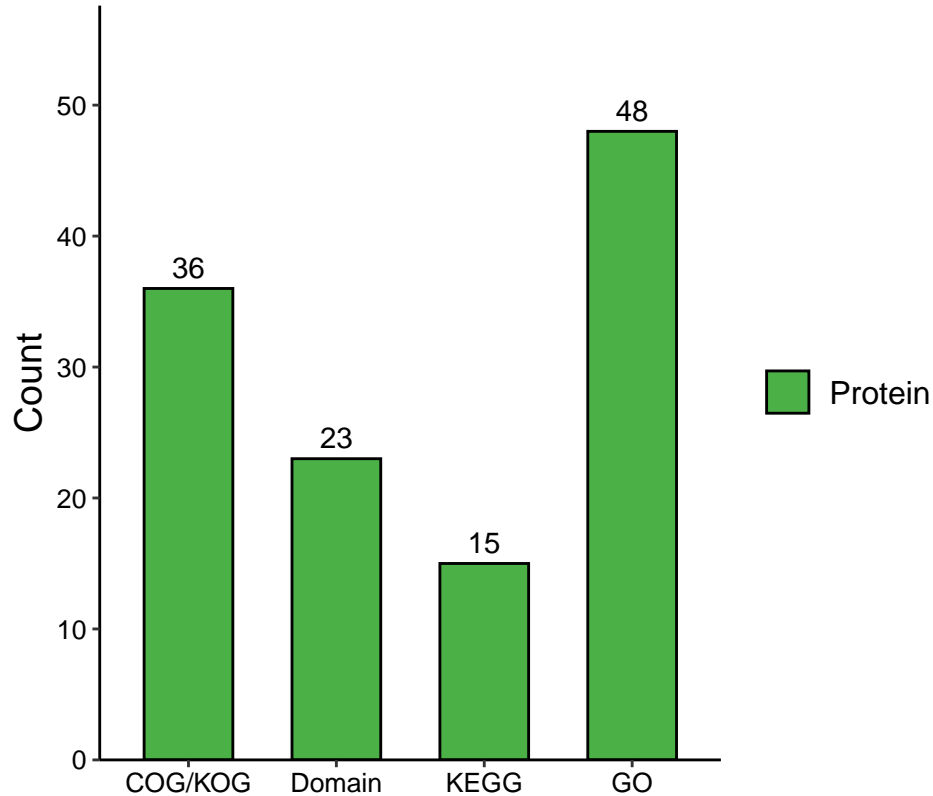

Supplement: Supplemental Information 2 — Supplemental Figures: Figure S1A: Peptide length, peptides per protein, distribution of coverage (%) and MW (kDa) of the LC-MS/MS analysis of rHSA from company A. Figure S2B: Peptide length, peptides per protein, distribution of coverage (%) and MW(kDa) of the LC-MS/MS analysis of rHSA from company B. Figure S3C: Peptide length, peptides per protein, distribution of coverage (%) and MW(kDa) of the LC-MS/MS analysis of pHSA from company C. Figure S4D: Peptide length, peptides per protein, distribution of coverage (%) and MW(kDa) of the LC-MS/MS analysis of pHSA from company D. Figure S5E: Peptide length, peptides per protein, distribution of coverage (%) and MW(kDa) of the LC-MS/MS analysis of pHSA from company E. Figure S6F: Peptide length, peptides per protein, distribution of coverage (%) and MW(kDa) of the LC-MS/MS analysis of pHSA from company F. Figure S7G: Peptide length, peptides per protein, distribution of coverage (%) and MW(kDa) of the LC-MS/MS analysis of pHSA from company G. Figure S8H: Peptide length, peptides per protein, distribution of coverage (%) and MW(kDa) of the LC-MS/MS analysis of pHSA from company H. Figure S9: GO enrichment analysis of the APs in pHSA. Figure S10: Subcellular localization prediction of the APs in pHSA. Figure S11: COG/KOG enrichment analysis of the APs in pHSA. Figure S12: KEGG pathway enrichment analysis of the APs in pHSA. Supplemental Tables: Table S1A: The protein and peptide identified in rHSA from company A. Table S2B: The protein and peptide identified in rHSA from company B. Table S3C: The protein and peptide identified in pHSA from company C. Table S4D: The protein and peptide identified in pHSA from company D. Table S5E: The protein and peptide identified in pHSA from company E. Table S6F: The protein and peptide identified in pHSA from company F. Table S7G: The protein and peptide identified in pHSA from company G. Table S8H: The protein and peptide identified in pHSA from company H. Table S9: The relative abunda [file peerj-13-19624-s002.zip › Supplementary/Supplementary File/Supplementary File1/1-Functional_annotation/barAnnotation_plot.pdf]

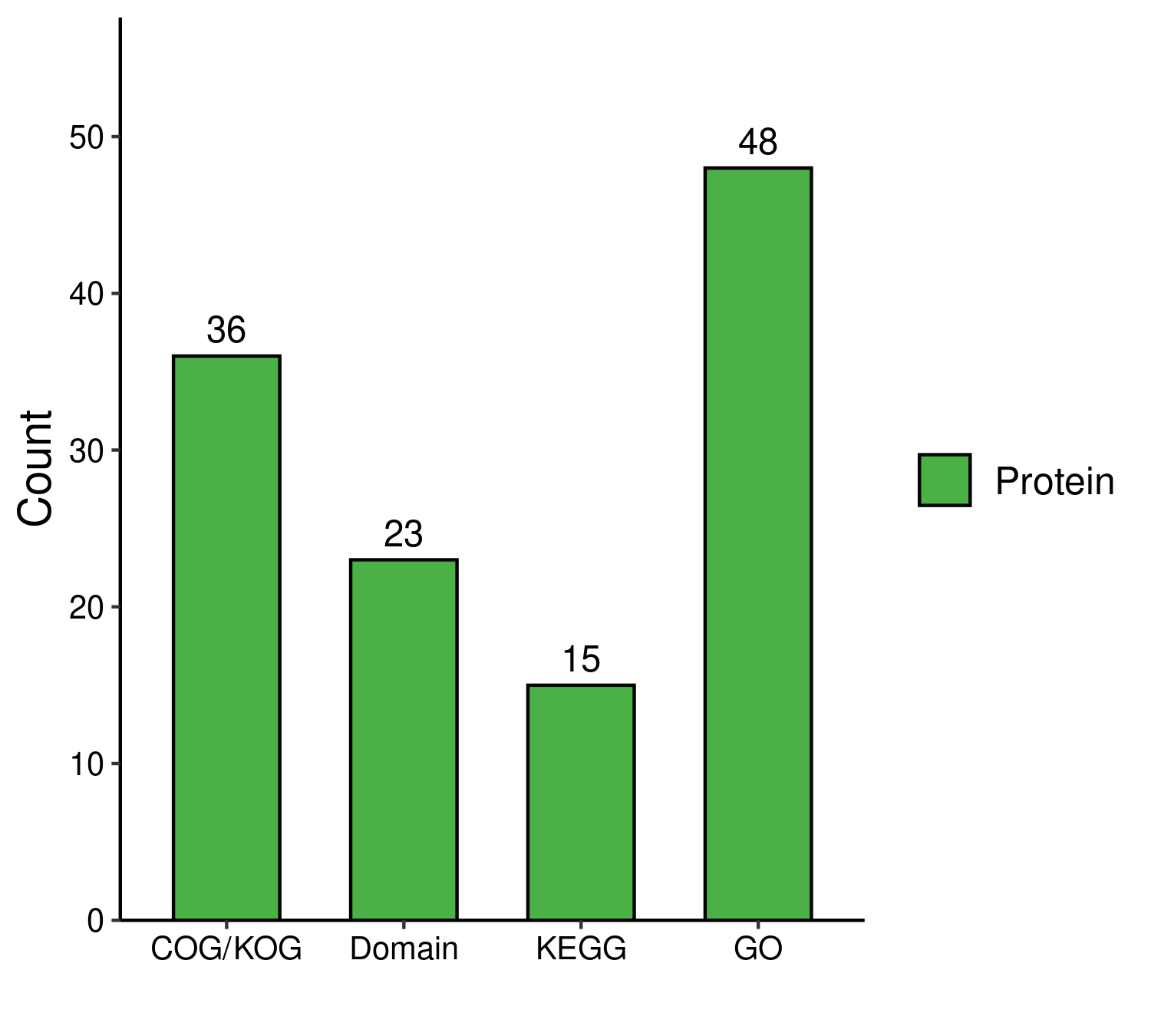

Supplement: Supplemental Information 2 — Supplemental Figures: Figure S1A: Peptide length, peptides per protein, distribution of coverage (%) and MW (kDa) of the LC-MS/MS analysis of rHSA from company A. Figure S2B: Peptide length, peptides per protein, distribution of coverage (%) and MW(kDa) of the LC-MS/MS analysis of rHSA from company B. Figure S3C: Peptide length, peptides per protein, distribution of coverage (%) and MW(kDa) of the LC-MS/MS analysis of pHSA from company C. Figure S4D: Peptide length, peptides per protein, distribution of coverage (%) and MW(kDa) of the LC-MS/MS analysis of pHSA from company D. Figure S5E: Peptide length, peptides per protein, distribution of coverage (%) and MW(kDa) of the LC-MS/MS analysis of pHSA from company E. Figure S6F: Peptide length, peptides per protein, distribution of coverage (%) and MW(kDa) of the LC-MS/MS analysis of pHSA from company F. Figure S7G: Peptide length, peptides per protein, distribution of coverage (%) and MW(kDa) of the LC-MS/MS analysis of pHSA from company G. Figure S8H: Peptide length, peptides per protein, distribution of coverage (%) and MW(kDa) of the LC-MS/MS analysis of pHSA from company H. Figure S9: GO enrichment analysis of the APs in pHSA. Figure S10: Subcellular localization prediction of the APs in pHSA. Figure S11: COG/KOG enrichment analysis of the APs in pHSA. Figure S12: KEGG pathway enrichment analysis of the APs in pHSA. Supplemental Tables: Table S1A: The protein and peptide identified in rHSA from company A. Table S2B: The protein and peptide identified in rHSA from company B. Table S3C: The protein and peptide identified in pHSA from company C. Table S4D: The protein and peptide identified in pHSA from company D. Table S5E: The protein and peptide identified in pHSA from company E. Table S6F: The protein and peptide identified in pHSA from company F. Table S7G: The protein and peptide identified in pHSA from company G. Table S8H: The protein and peptide identified in pHSA from company H. Table S9: The relative abunda [file peerj-13-19624-s002.zip › Supplementary/Supplementary File/Supplementary File1/1-Functional_annotation/barAnnotation_plot.png]

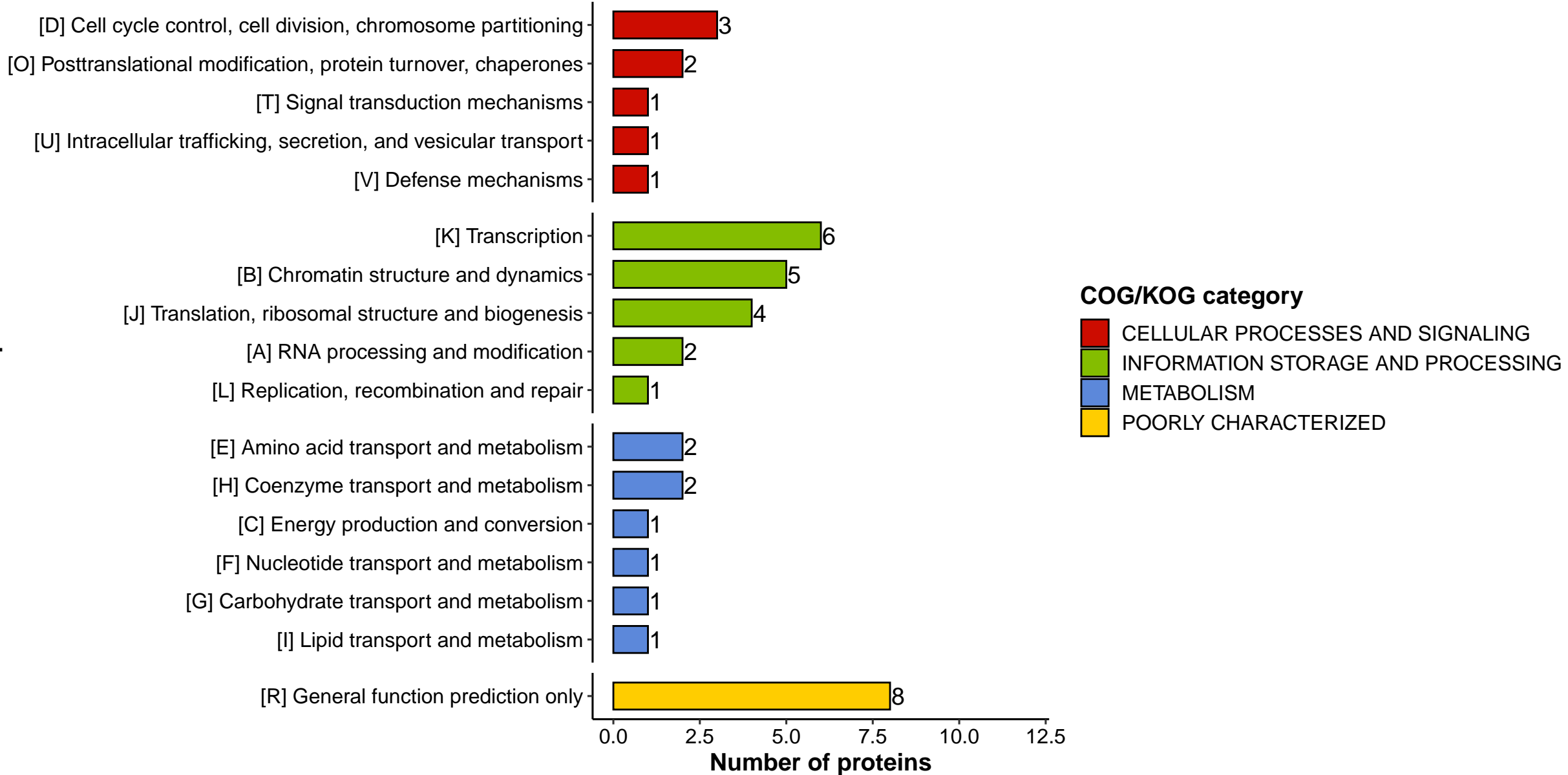

Supplement: Supplemental Information 2 — Supplemental Figures: Figure S1A: Peptide length, peptides per protein, distribution of coverage (%) and MW (kDa) of the LC-MS/MS analysis of rHSA from company A. Figure S2B: Peptide length, peptides per protein, distribution of coverage (%) and MW(kDa) of the LC-MS/MS analysis of rHSA from company B. Figure S3C: Peptide length, peptides per protein, distribution of coverage (%) and MW(kDa) of the LC-MS/MS analysis of pHSA from company C. Figure S4D: Peptide length, peptides per protein, distribution of coverage (%) and MW(kDa) of the LC-MS/MS analysis of pHSA from company D. Figure S5E: Peptide length, peptides per protein, distribution of coverage (%) and MW(kDa) of the LC-MS/MS analysis of pHSA from company E. Figure S6F: Peptide length, peptides per protein, distribution of coverage (%) and MW(kDa) of the LC-MS/MS analysis of pHSA from company F. Figure S7G: Peptide length, peptides per protein, distribution of coverage (%) and MW(kDa) of the LC-MS/MS analysis of pHSA from company G. Figure S8H: Peptide length, peptides per protein, distribution of coverage (%) and MW(kDa) of the LC-MS/MS analysis of pHSA from company H. Figure S9: GO enrichment analysis of the APs in pHSA. Figure S10: Subcellular localization prediction of the APs in pHSA. Figure S11: COG/KOG enrichment analysis of the APs in pHSA. Figure S12: KEGG pathway enrichment analysis of the APs in pHSA. Supplemental Tables: Table S1A: The protein and peptide identified in rHSA from company A. Table S2B: The protein and peptide identified in rHSA from company B. Table S3C: The protein and peptide identified in pHSA from company C. Table S4D: The protein and peptide identified in pHSA from company D. Table S5E: The protein and peptide identified in pHSA from company E. Table S6F: The protein and peptide identified in pHSA from company F. Table S7G: The protein and peptide identified in pHSA from company G. Table S8H: The protein and peptide identified in pHSA from company H. Table S9: The relative abunda [file peerj-13-19624-s002.zip › Supplementary/Supplementary File/Supplementary File1/2-Functional_classification/ident-COG_classify.pdf]

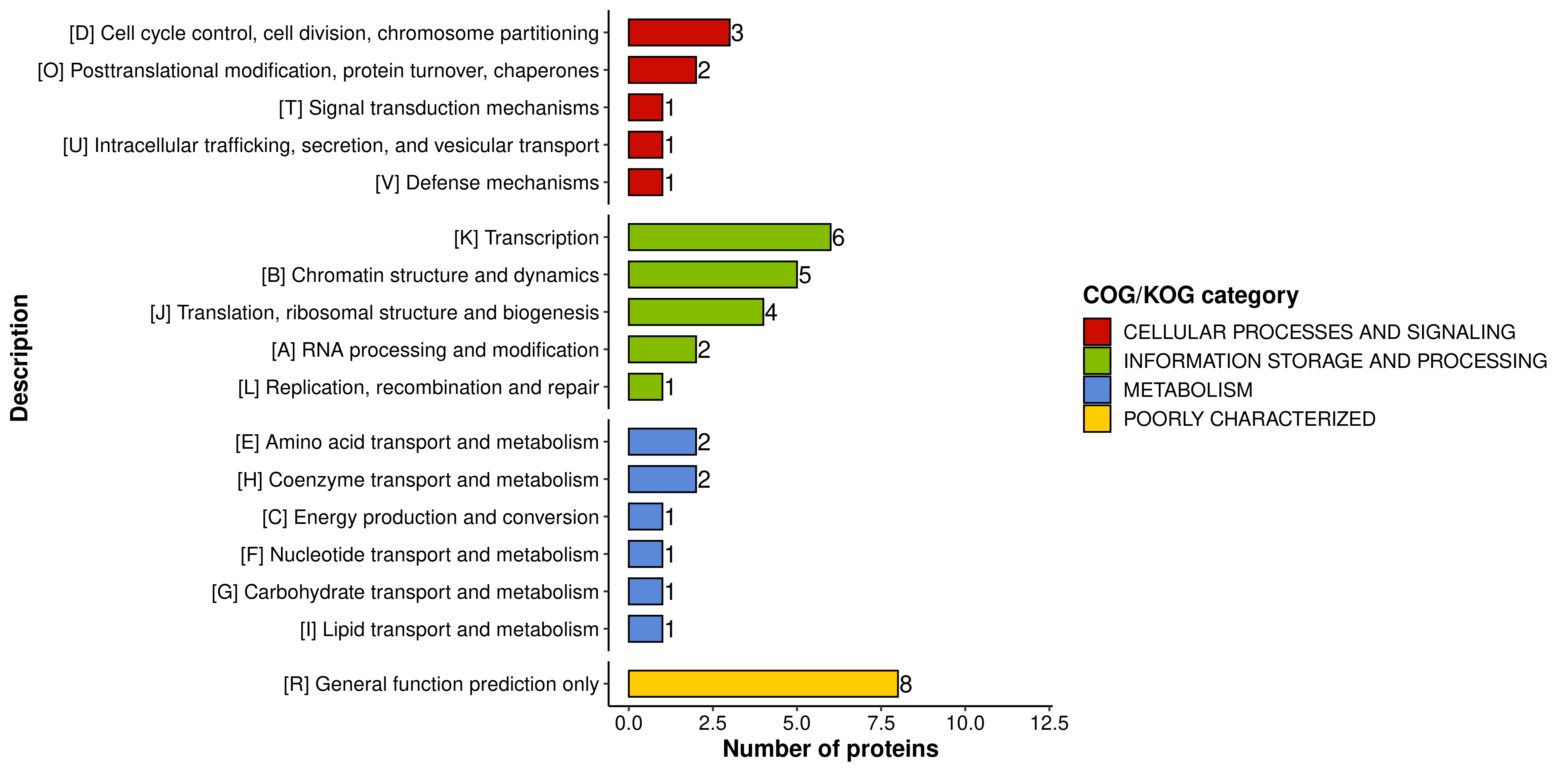

Supplement: Supplemental Information 2 — Supplemental Figures: Figure S1A: Peptide length, peptides per protein, distribution of coverage (%) and MW (kDa) of the LC-MS/MS analysis of rHSA from company A. Figure S2B: Peptide length, peptides per protein, distribution of coverage (%) and MW(kDa) of the LC-MS/MS analysis of rHSA from company B. Figure S3C: Peptide length, peptides per protein, distribution of coverage (%) and MW(kDa) of the LC-MS/MS analysis of pHSA from company C. Figure S4D: Peptide length, peptides per protein, distribution of coverage (%) and MW(kDa) of the LC-MS/MS analysis of pHSA from company D. Figure S5E: Peptide length, peptides per protein, distribution of coverage (%) and MW(kDa) of the LC-MS/MS analysis of pHSA from company E. Figure S6F: Peptide length, peptides per protein, distribution of coverage (%) and MW(kDa) of the LC-MS/MS analysis of pHSA from company F. Figure S7G: Peptide length, peptides per protein, distribution of coverage (%) and MW(kDa) of the LC-MS/MS analysis of pHSA from company G. Figure S8H: Peptide length, peptides per protein, distribution of coverage (%) and MW(kDa) of the LC-MS/MS analysis of pHSA from company H. Figure S9: GO enrichment analysis of the APs in pHSA. Figure S10: Subcellular localization prediction of the APs in pHSA. Figure S11: COG/KOG enrichment analysis of the APs in pHSA. Figure S12: KEGG pathway enrichment analysis of the APs in pHSA. Supplemental Tables: Table S1A: The protein and peptide identified in rHSA from company A. Table S2B: The protein and peptide identified in rHSA from company B. Table S3C: The protein and peptide identified in pHSA from company C. Table S4D: The protein and peptide identified in pHSA from company D. Table S5E: The protein and peptide identified in pHSA from company E. Table S6F: The protein and peptide identified in pHSA from company F. Table S7G: The protein and peptide identified in pHSA from company G. Table S8H: The protein and peptide identified in pHSA from company H. Table S9: The relative abunda [file peerj-13-19624-s002.zip › Supplementary/Supplementary File/Supplementary File1/2-Functional_classification/ident-COG_classify.png]

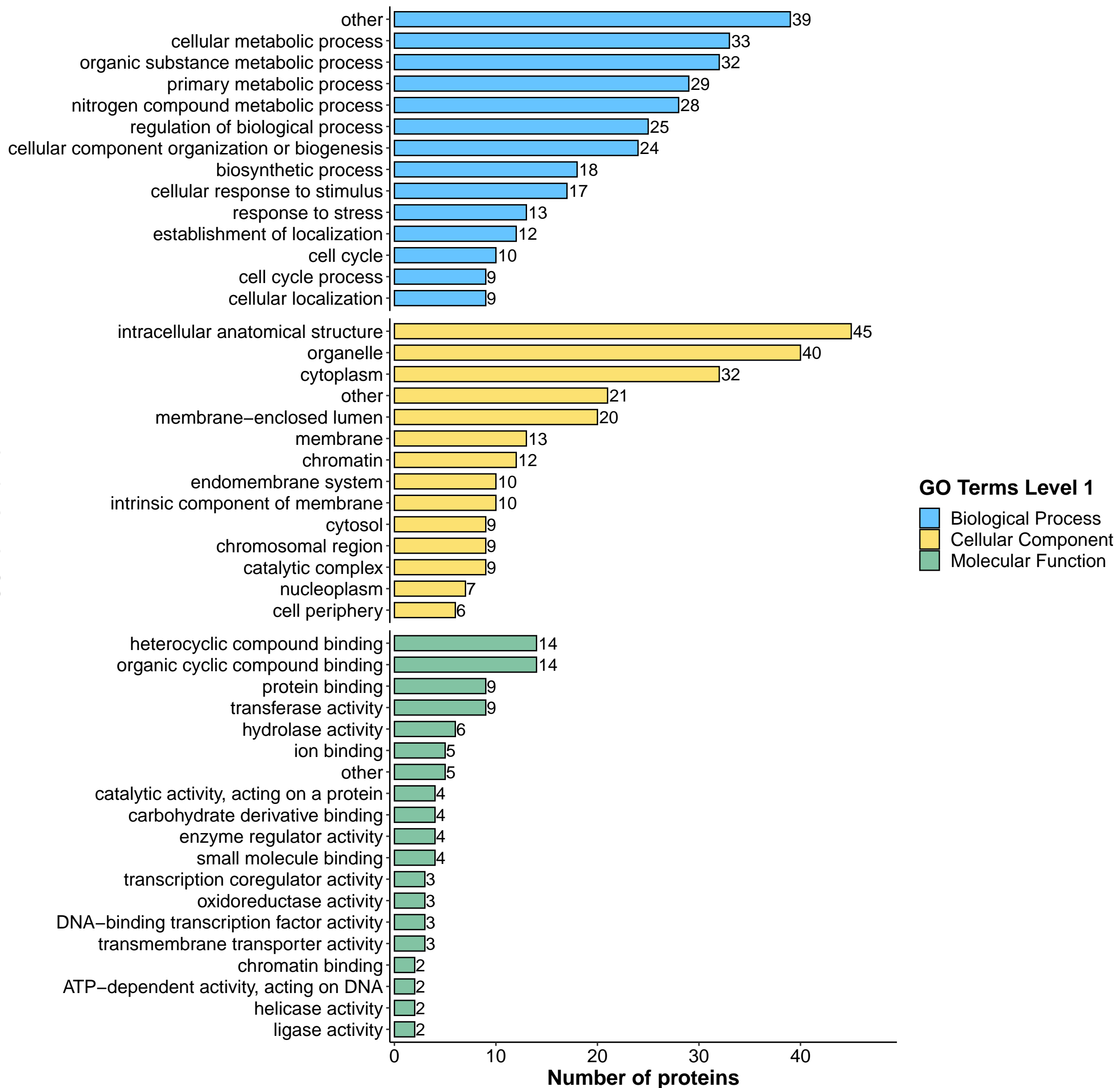

Supplement: Supplemental Information 2 — Supplemental Figures: Figure S1A: Peptide length, peptides per protein, distribution of coverage (%) and MW (kDa) of the LC-MS/MS analysis of rHSA from company A. Figure S2B: Peptide length, peptides per protein, distribution of coverage (%) and MW(kDa) of the LC-MS/MS analysis of rHSA from company B. Figure S3C: Peptide length, peptides per protein, distribution of coverage (%) and MW(kDa) of the LC-MS/MS analysis of pHSA from company C. Figure S4D: Peptide length, peptides per protein, distribution of coverage (%) and MW(kDa) of the LC-MS/MS analysis of pHSA from company D. Figure S5E: Peptide length, peptides per protein, distribution of coverage (%) and MW(kDa) of the LC-MS/MS analysis of pHSA from company E. Figure S6F: Peptide length, peptides per protein, distribution of coverage (%) and MW(kDa) of the LC-MS/MS analysis of pHSA from company F. Figure S7G: Peptide length, peptides per protein, distribution of coverage (%) and MW(kDa) of the LC-MS/MS analysis of pHSA from company G. Figure S8H: Peptide length, peptides per protein, distribution of coverage (%) and MW(kDa) of the LC-MS/MS analysis of pHSA from company H. Figure S9: GO enrichment analysis of the APs in pHSA. Figure S10: Subcellular localization prediction of the APs in pHSA. Figure S11: COG/KOG enrichment analysis of the APs in pHSA. Figure S12: KEGG pathway enrichment analysis of the APs in pHSA. Supplemental Tables: Table S1A: The protein and peptide identified in rHSA from company A. Table S2B: The protein and peptide identified in rHSA from company B. Table S3C: The protein and peptide identified in pHSA from company C. Table S4D: The protein and peptide identified in pHSA from company D. Table S5E: The protein and peptide identified in pHSA from company E. Table S6F: The protein and peptide identified in pHSA from company F. Table S7G: The protein and peptide identified in pHSA from company G. Table S8H: The protein and peptide identified in pHSA from company H. Table S9: The relative abunda [file peerj-13-19624-s002.zip › Supplementary/Supplementary File/Supplementary File1/2-Functional_classification/ident-GO_classify.pdf]

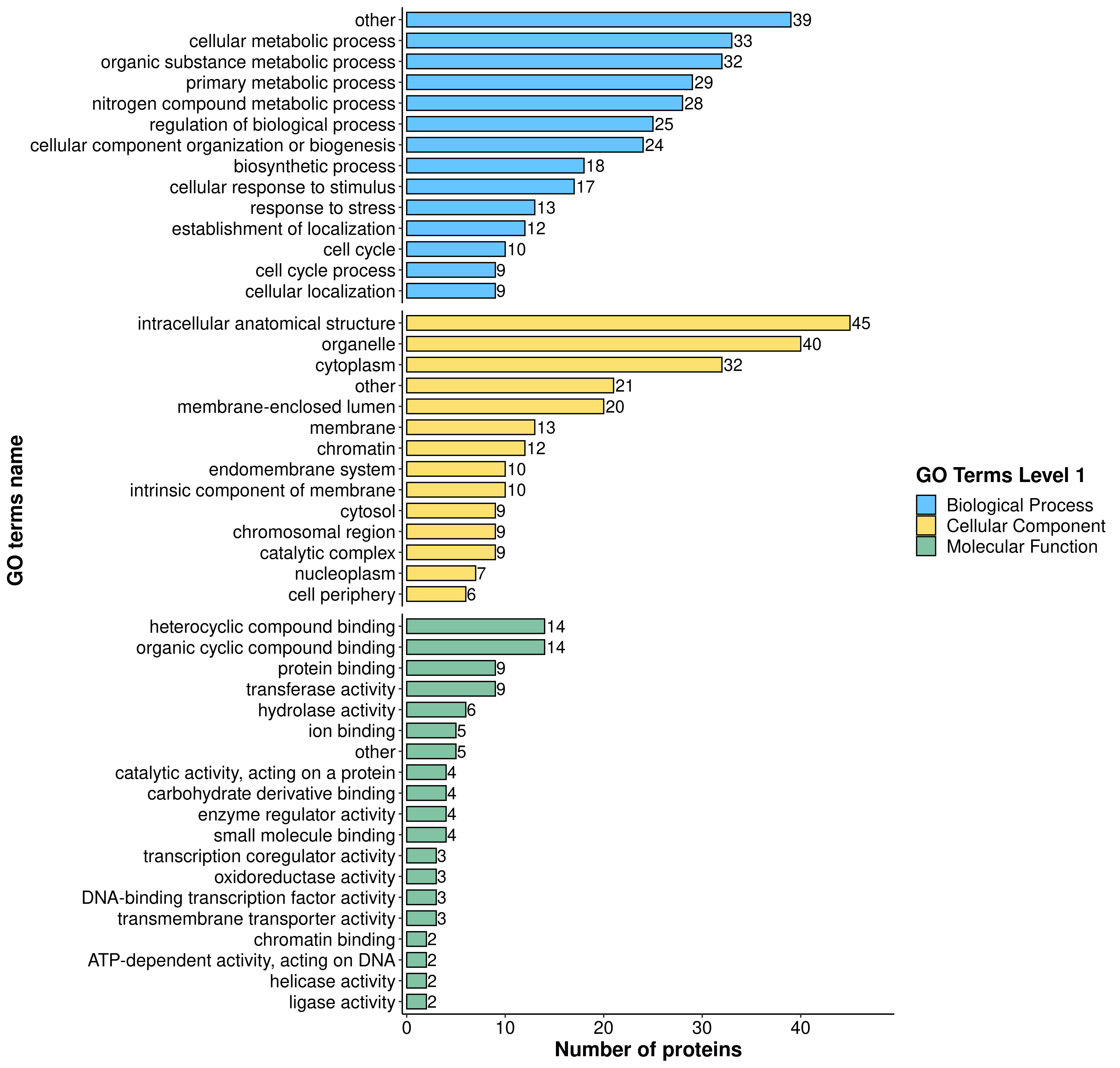

Supplement: Supplemental Information 2 — Supplemental Figures: Figure S1A: Peptide length, peptides per protein, distribution of coverage (%) and MW (kDa) of the LC-MS/MS analysis of rHSA from company A. Figure S2B: Peptide length, peptides per protein, distribution of coverage (%) and MW(kDa) of the LC-MS/MS analysis of rHSA from company B. Figure S3C: Peptide length, peptides per protein, distribution of coverage (%) and MW(kDa) of the LC-MS/MS analysis of pHSA from company C. Figure S4D: Peptide length, peptides per protein, distribution of coverage (%) and MW(kDa) of the LC-MS/MS analysis of pHSA from company D. Figure S5E: Peptide length, peptides per protein, distribution of coverage (%) and MW(kDa) of the LC-MS/MS analysis of pHSA from company E. Figure S6F: Peptide length, peptides per protein, distribution of coverage (%) and MW(kDa) of the LC-MS/MS analysis of pHSA from company F. Figure S7G: Peptide length, peptides per protein, distribution of coverage (%) and MW(kDa) of the LC-MS/MS analysis of pHSA from company G. Figure S8H: Peptide length, peptides per protein, distribution of coverage (%) and MW(kDa) of the LC-MS/MS analysis of pHSA from company H. Figure S9: GO enrichment analysis of the APs in pHSA. Figure S10: Subcellular localization prediction of the APs in pHSA. Figure S11: COG/KOG enrichment analysis of the APs in pHSA. Figure S12: KEGG pathway enrichment analysis of the APs in pHSA. Supplemental Tables: Table S1A: The protein and peptide identified in rHSA from company A. Table S2B: The protein and peptide identified in rHSA from company B. Table S3C: The protein and peptide identified in pHSA from company C. Table S4D: The protein and peptide identified in pHSA from company D. Table S5E: The protein and peptide identified in pHSA from company E. Table S6F: The protein and peptide identified in pHSA from company F. Table S7G: The protein and peptide identified in pHSA from company G. Table S8H: The protein and peptide identified in pHSA from company H. Table S9: The relative abunda [file peerj-13-19624-s002.zip › Supplementary/Supplementary File/Supplementary File1/2-Functional_classification/ident-GO_classify.png]

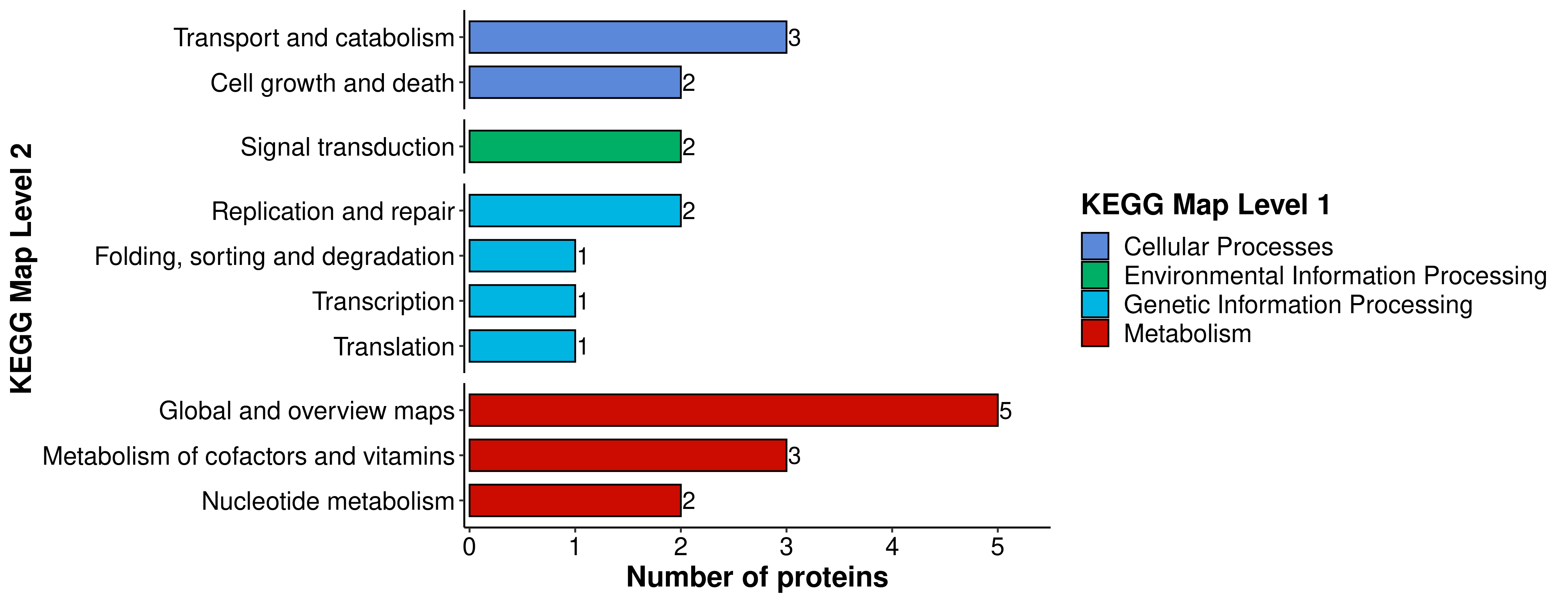

Supplement: Supplemental Information 2 — Supplemental Figures: Figure S1A: Peptide length, peptides per protein, distribution of coverage (%) and MW (kDa) of the LC-MS/MS analysis of rHSA from company A. Figure S2B: Peptide length, peptides per protein, distribution of coverage (%) and MW(kDa) of the LC-MS/MS analysis of rHSA from company B. Figure S3C: Peptide length, peptides per protein, distribution of coverage (%) and MW(kDa) of the LC-MS/MS analysis of pHSA from company C. Figure S4D: Peptide length, peptides per protein, distribution of coverage (%) and MW(kDa) of the LC-MS/MS analysis of pHSA from company D. Figure S5E: Peptide length, peptides per protein, distribution of coverage (%) and MW(kDa) of the LC-MS/MS analysis of pHSA from company E. Figure S6F: Peptide length, peptides per protein, distribution of coverage (%) and MW(kDa) of the LC-MS/MS analysis of pHSA from company F. Figure S7G: Peptide length, peptides per protein, distribution of coverage (%) and MW(kDa) of the LC-MS/MS analysis of pHSA from company G. Figure S8H: Peptide length, peptides per protein, distribution of coverage (%) and MW(kDa) of the LC-MS/MS analysis of pHSA from company H. Figure S9: GO enrichment analysis of the APs in pHSA. Figure S10: Subcellular localization prediction of the APs in pHSA. Figure S11: COG/KOG enrichment analysis of the APs in pHSA. Figure S12: KEGG pathway enrichment analysis of the APs in pHSA. Supplemental Tables: Table S1A: The protein and peptide identified in rHSA from company A. Table S2B: The protein and peptide identified in rHSA from company B. Table S3C: The protein and peptide identified in pHSA from company C. Table S4D: The protein and peptide identified in pHSA from company D. Table S5E: The protein and peptide identified in pHSA from company E. Table S6F: The protein and peptide identified in pHSA from company F. Table S7G: The protein and peptide identified in pHSA from company G. Table S8H: The protein and peptide identified in pHSA from company H. Table S9: The relative abunda [file peerj-13-19624-s002.zip › Supplementary/Supplementary File/Supplementary File1/2-Functional_classification/ident-KEGG_map_classify 2.png]

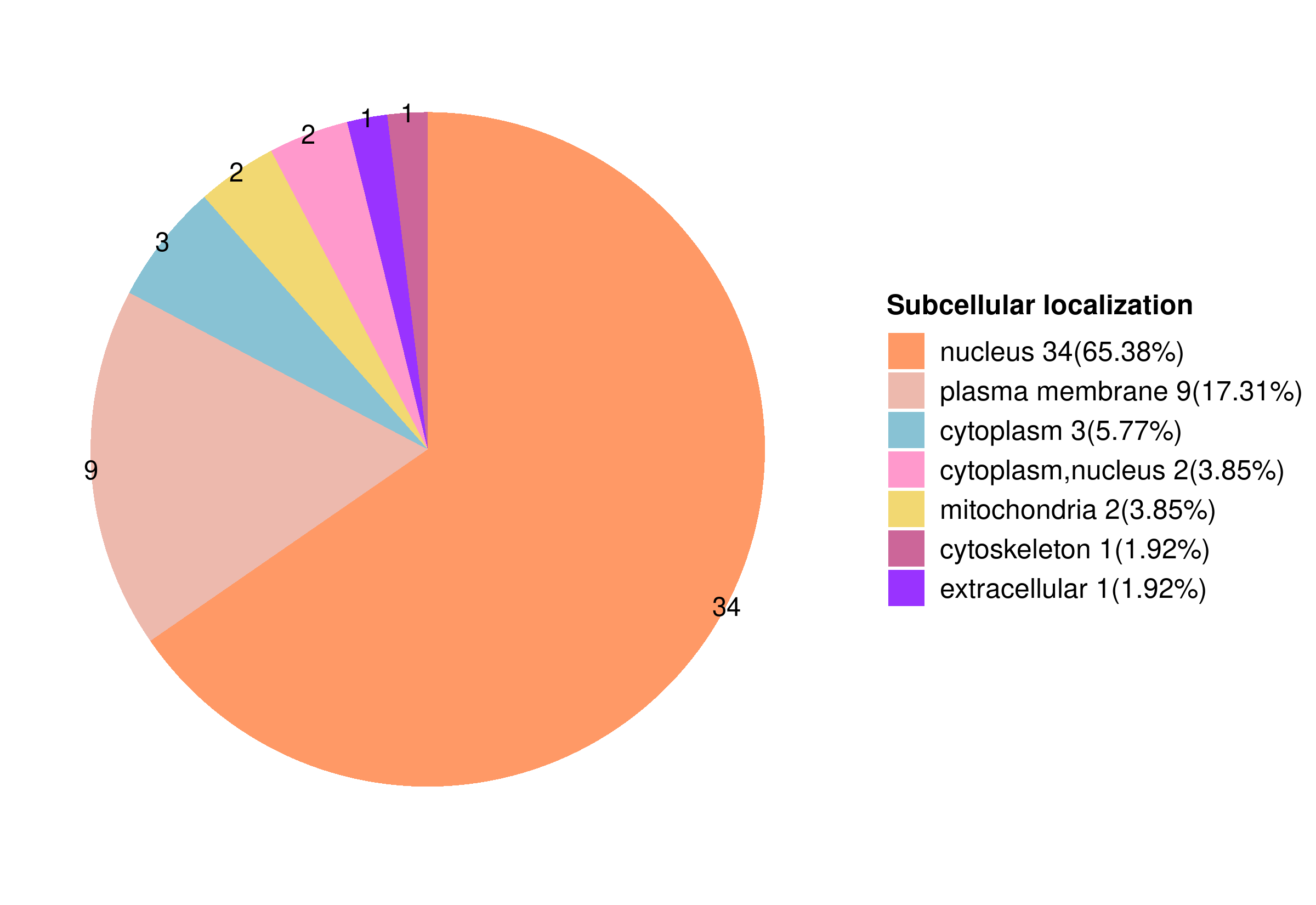

Supplement: Supplemental Information 2 — Supplemental Figures: Figure S1A: Peptide length, peptides per protein, distribution of coverage (%) and MW (kDa) of the LC-MS/MS analysis of rHSA from company A. Figure S2B: Peptide length, peptides per protein, distribution of coverage (%) and MW(kDa) of the LC-MS/MS analysis of rHSA from company B. Figure S3C: Peptide length, peptides per protein, distribution of coverage (%) and MW(kDa) of the LC-MS/MS analysis of pHSA from company C. Figure S4D: Peptide length, peptides per protein, distribution of coverage (%) and MW(kDa) of the LC-MS/MS analysis of pHSA from company D. Figure S5E: Peptide length, peptides per protein, distribution of coverage (%) and MW(kDa) of the LC-MS/MS analysis of pHSA from company E. Figure S6F: Peptide length, peptides per protein, distribution of coverage (%) and MW(kDa) of the LC-MS/MS analysis of pHSA from company F. Figure S7G: Peptide length, peptides per protein, distribution of coverage (%) and MW(kDa) of the LC-MS/MS analysis of pHSA from company G. Figure S8H: Peptide length, peptides per protein, distribution of coverage (%) and MW(kDa) of the LC-MS/MS analysis of pHSA from company H. Figure S9: GO enrichment analysis of the APs in pHSA. Figure S10: Subcellular localization prediction of the APs in pHSA. Figure S11: COG/KOG enrichment analysis of the APs in pHSA. Figure S12: KEGG pathway enrichment analysis of the APs in pHSA. Supplemental Tables: Table S1A: The protein and peptide identified in rHSA from company A. Table S2B: The protein and peptide identified in rHSA from company B. Table S3C: The protein and peptide identified in pHSA from company C. Table S4D: The protein and peptide identified in pHSA from company D. Table S5E: The protein and peptide identified in pHSA from company E. Table S6F: The protein and peptide identified in pHSA from company F. Table S7G: The protein and peptide identified in pHSA from company G. Table S8H: The protein and peptide identified in pHSA from company H. Table S9: The relative abunda [file peerj-13-19624-s002.zip › Supplementary/Supplementary File/Supplementary File1/2-Functional_classification/ident-Subcell_classify.png]

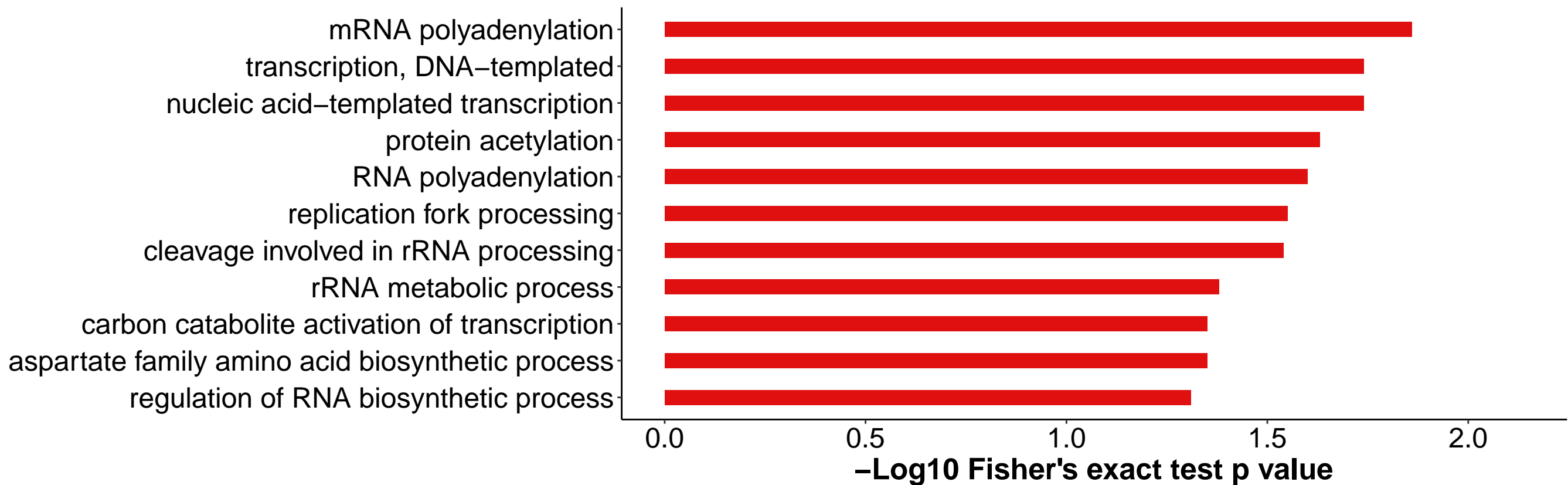

Supplement: Supplemental Information 2 — Supplemental Figures: Figure S1A: Peptide length, peptides per protein, distribution of coverage (%) and MW (kDa) of the LC-MS/MS analysis of rHSA from company A. Figure S2B: Peptide length, peptides per protein, distribution of coverage (%) and MW(kDa) of the LC-MS/MS analysis of rHSA from company B. Figure S3C: Peptide length, peptides per protein, distribution of coverage (%) and MW(kDa) of the LC-MS/MS analysis of pHSA from company C. Figure S4D: Peptide length, peptides per protein, distribution of coverage (%) and MW(kDa) of the LC-MS/MS analysis of pHSA from company D. Figure S5E: Peptide length, peptides per protein, distribution of coverage (%) and MW(kDa) of the LC-MS/MS analysis of pHSA from company E. Figure S6F: Peptide length, peptides per protein, distribution of coverage (%) and MW(kDa) of the LC-MS/MS analysis of pHSA from company F. Figure S7G: Peptide length, peptides per protein, distribution of coverage (%) and MW(kDa) of the LC-MS/MS analysis of pHSA from company G. Figure S8H: Peptide length, peptides per protein, distribution of coverage (%) and MW(kDa) of the LC-MS/MS analysis of pHSA from company H. Figure S9: GO enrichment analysis of the APs in pHSA. Figure S10: Subcellular localization prediction of the APs in pHSA. Figure S11: COG/KOG enrichment analysis of the APs in pHSA. Figure S12: KEGG pathway enrichment analysis of the APs in pHSA. Supplemental Tables: Table S1A: The protein and peptide identified in rHSA from company A. Table S2B: The protein and peptide identified in rHSA from company B. Table S3C: The protein and peptide identified in pHSA from company C. Table S4D: The protein and peptide identified in pHSA from company D. Table S5E: The protein and peptide identified in pHSA from company E. Table S6F: The protein and peptide identified in pHSA from company F. Table S7G: The protein and peptide identified in pHSA from company G. Table S8H: The protein and peptide identified in pHSA from company H. Table S9: The relative abunda [file peerj-13-19624-s002.zip › Supplementary/Supplementary File/Supplementary File1/3-Functional_enrichment/ident-BP_barplot.pdf]

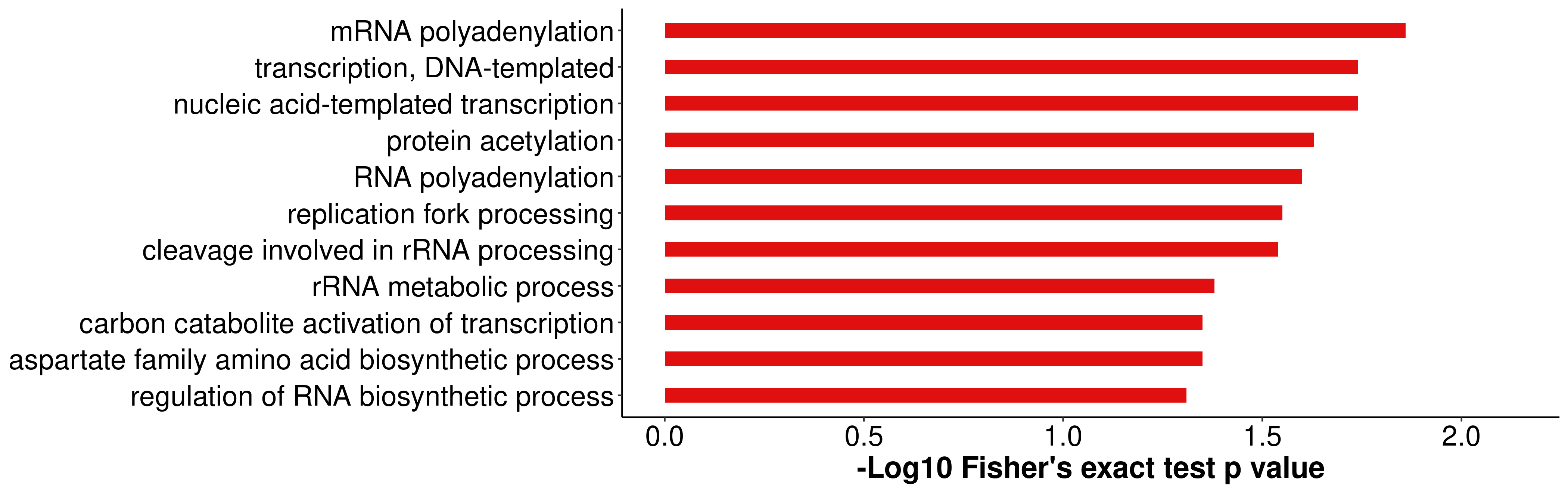

Supplement: Supplemental Information 2 — Supplemental Figures: Figure S1A: Peptide length, peptides per protein, distribution of coverage (%) and MW (kDa) of the LC-MS/MS analysis of rHSA from company A. Figure S2B: Peptide length, peptides per protein, distribution of coverage (%) and MW(kDa) of the LC-MS/MS analysis of rHSA from company B. Figure S3C: Peptide length, peptides per protein, distribution of coverage (%) and MW(kDa) of the LC-MS/MS analysis of pHSA from company C. Figure S4D: Peptide length, peptides per protein, distribution of coverage (%) and MW(kDa) of the LC-MS/MS analysis of pHSA from company D. Figure S5E: Peptide length, peptides per protein, distribution of coverage (%) and MW(kDa) of the LC-MS/MS analysis of pHSA from company E. Figure S6F: Peptide length, peptides per protein, distribution of coverage (%) and MW(kDa) of the LC-MS/MS analysis of pHSA from company F. Figure S7G: Peptide length, peptides per protein, distribution of coverage (%) and MW(kDa) of the LC-MS/MS analysis of pHSA from company G. Figure S8H: Peptide length, peptides per protein, distribution of coverage (%) and MW(kDa) of the LC-MS/MS analysis of pHSA from company H. Figure S9: GO enrichment analysis of the APs in pHSA. Figure S10: Subcellular localization prediction of the APs in pHSA. Figure S11: COG/KOG enrichment analysis of the APs in pHSA. Figure S12: KEGG pathway enrichment analysis of the APs in pHSA. Supplemental Tables: Table S1A: The protein and peptide identified in rHSA from company A. Table S2B: The protein and peptide identified in rHSA from company B. Table S3C: The protein and peptide identified in pHSA from company C. Table S4D: The protein and peptide identified in pHSA from company D. Table S5E: The protein and peptide identified in pHSA from company E. Table S6F: The protein and peptide identified in pHSA from company F. Table S7G: The protein and peptide identified in pHSA from company G. Table S8H: The protein and peptide identified in pHSA from company H. Table S9: The relative abunda [file peerj-13-19624-s002.zip › Supplementary/Supplementary File/Supplementary File1/3-Functional_enrichment/ident-BP_barplot.png]

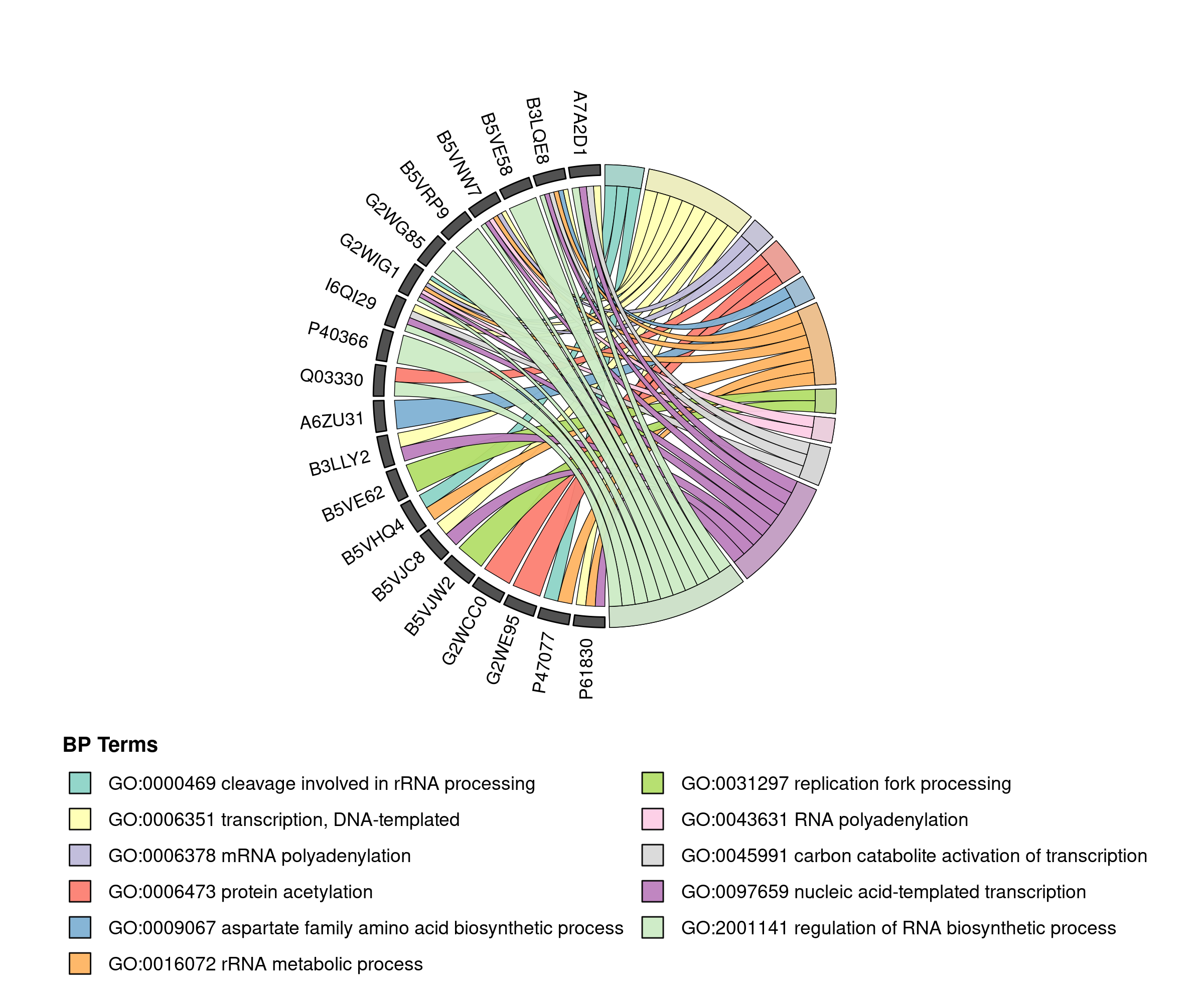

Supplement: Supplemental Information 2 — Supplemental Figures: Figure S1A: Peptide length, peptides per protein, distribution of coverage (%) and MW (kDa) of the LC-MS/MS analysis of rHSA from company A. Figure S2B: Peptide length, peptides per protein, distribution of coverage (%) and MW(kDa) of the LC-MS/MS analysis of rHSA from company B. Figure S3C: Peptide length, peptides per protein, distribution of coverage (%) and MW(kDa) of the LC-MS/MS analysis of pHSA from company C. Figure S4D: Peptide length, peptides per protein, distribution of coverage (%) and MW(kDa) of the LC-MS/MS analysis of pHSA from company D. Figure S5E: Peptide length, peptides per protein, distribution of coverage (%) and MW(kDa) of the LC-MS/MS analysis of pHSA from company E. Figure S6F: Peptide length, peptides per protein, distribution of coverage (%) and MW(kDa) of the LC-MS/MS analysis of pHSA from company F. Figure S7G: Peptide length, peptides per protein, distribution of coverage (%) and MW(kDa) of the LC-MS/MS analysis of pHSA from company G. Figure S8H: Peptide length, peptides per protein, distribution of coverage (%) and MW(kDa) of the LC-MS/MS analysis of pHSA from company H. Figure S9: GO enrichment analysis of the APs in pHSA. Figure S10: Subcellular localization prediction of the APs in pHSA. Figure S11: COG/KOG enrichment analysis of the APs in pHSA. Figure S12: KEGG pathway enrichment analysis of the APs in pHSA. Supplemental Tables: Table S1A: The protein and peptide identified in rHSA from company A. Table S2B: The protein and peptide identified in rHSA from company B. Table S3C: The protein and peptide identified in pHSA from company C. Table S4D: The protein and peptide identified in pHSA from company D. Table S5E: The protein and peptide identified in pHSA from company E. Table S6F: The protein and peptide identified in pHSA from company F. Table S7G: The protein and peptide identified in pHSA from company G. Table S8H: The protein and peptide identified in pHSA from company H. Table S9: The relative abunda [file peerj-13-19624-s002.zip › Supplementary/Supplementary File/Supplementary File1/3-Functional_enrichment/ident-BP_cirplot 2.png]

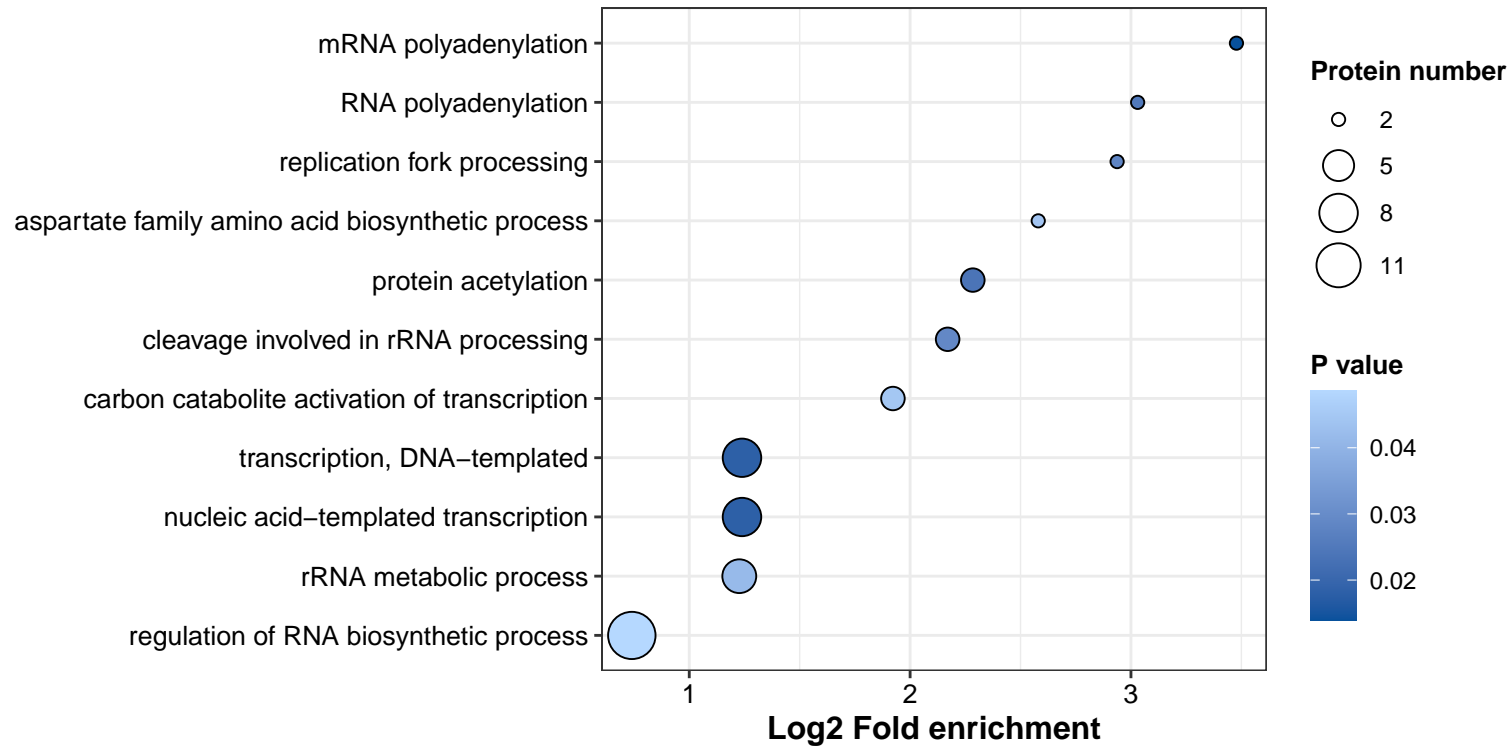

Supplement: Supplemental Information 2 — Supplemental Figures: Figure S1A: Peptide length, peptides per protein, distribution of coverage (%) and MW (kDa) of the LC-MS/MS analysis of rHSA from company A. Figure S2B: Peptide length, peptides per protein, distribution of coverage (%) and MW(kDa) of the LC-MS/MS analysis of rHSA from company B. Figure S3C: Peptide length, peptides per protein, distribution of coverage (%) and MW(kDa) of the LC-MS/MS analysis of pHSA from company C. Figure S4D: Peptide length, peptides per protein, distribution of coverage (%) and MW(kDa) of the LC-MS/MS analysis of pHSA from company D. Figure S5E: Peptide length, peptides per protein, distribution of coverage (%) and MW(kDa) of the LC-MS/MS analysis of pHSA from company E. Figure S6F: Peptide length, peptides per protein, distribution of coverage (%) and MW(kDa) of the LC-MS/MS analysis of pHSA from company F. Figure S7G: Peptide length, peptides per protein, distribution of coverage (%) and MW(kDa) of the LC-MS/MS analysis of pHSA from company G. Figure S8H: Peptide length, peptides per protein, distribution of coverage (%) and MW(kDa) of the LC-MS/MS analysis of pHSA from company H. Figure S9: GO enrichment analysis of the APs in pHSA. Figure S10: Subcellular localization prediction of the APs in pHSA. Figure S11: COG/KOG enrichment analysis of the APs in pHSA. Figure S12: KEGG pathway enrichment analysis of the APs in pHSA. Supplemental Tables: Table S1A: The protein and peptide identified in rHSA from company A. Table S2B: The protein and peptide identified in rHSA from company B. Table S3C: The protein and peptide identified in pHSA from company C. Table S4D: The protein and peptide identified in pHSA from company D. Table S5E: The protein and peptide identified in pHSA from company E. Table S6F: The protein and peptide identified in pHSA from company F. Table S7G: The protein and peptide identified in pHSA from company G. Table S8H: The protein and peptide identified in pHSA from company H. Table S9: The relative abunda [file peerj-13-19624-s002.zip › Supplementary/Supplementary File/Supplementary File1/3-Functional_enrichment/ident-BP_dotplot.pdf]

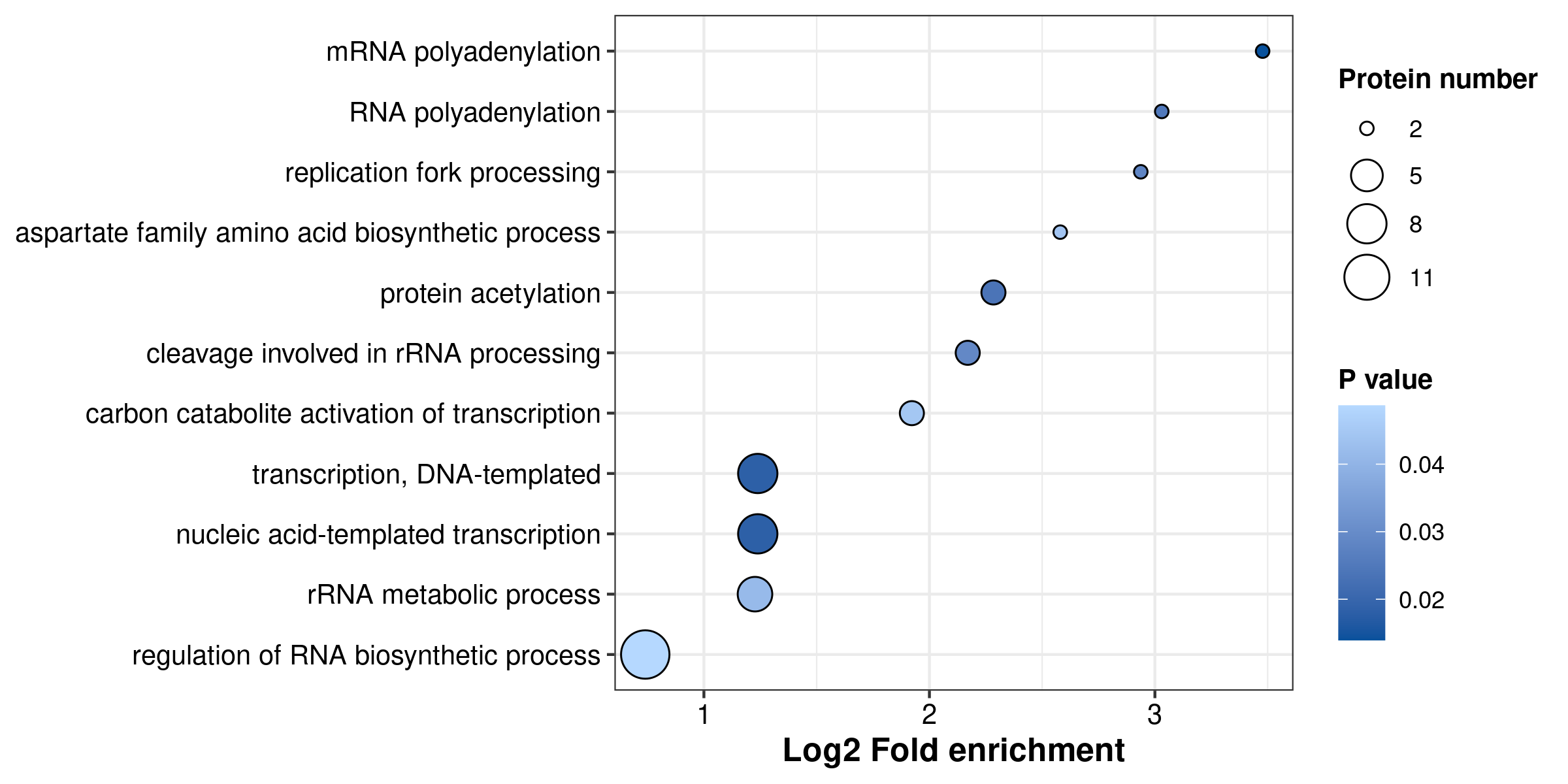

Supplement: Supplemental Information 2 — Supplemental Figures: Figure S1A: Peptide length, peptides per protein, distribution of coverage (%) and MW (kDa) of the LC-MS/MS analysis of rHSA from company A. Figure S2B: Peptide length, peptides per protein, distribution of coverage (%) and MW(kDa) of the LC-MS/MS analysis of rHSA from company B. Figure S3C: Peptide length, peptides per protein, distribution of coverage (%) and MW(kDa) of the LC-MS/MS analysis of pHSA from company C. Figure S4D: Peptide length, peptides per protein, distribution of coverage (%) and MW(kDa) of the LC-MS/MS analysis of pHSA from company D. Figure S5E: Peptide length, peptides per protein, distribution of coverage (%) and MW(kDa) of the LC-MS/MS analysis of pHSA from company E. Figure S6F: Peptide length, peptides per protein, distribution of coverage (%) and MW(kDa) of the LC-MS/MS analysis of pHSA from company F. Figure S7G: Peptide length, peptides per protein, distribution of coverage (%) and MW(kDa) of the LC-MS/MS analysis of pHSA from company G. Figure S8H: Peptide length, peptides per protein, distribution of coverage (%) and MW(kDa) of the LC-MS/MS analysis of pHSA from company H. Figure S9: GO enrichment analysis of the APs in pHSA. Figure S10: Subcellular localization prediction of the APs in pHSA. Figure S11: COG/KOG enrichment analysis of the APs in pHSA. Figure S12: KEGG pathway enrichment analysis of the APs in pHSA. Supplemental Tables: Table S1A: The protein and peptide identified in rHSA from company A. Table S2B: The protein and peptide identified in rHSA from company B. Table S3C: The protein and peptide identified in pHSA from company C. Table S4D: The protein and peptide identified in pHSA from company D. Table S5E: The protein and peptide identified in pHSA from company E. Table S6F: The protein and peptide identified in pHSA from company F. Table S7G: The protein and peptide identified in pHSA from company G. Table S8H: The protein and peptide identified in pHSA from company H. Table S9: The relative abunda [file peerj-13-19624-s002.zip › Supplementary/Supplementary File/Supplementary File1/3-Functional_enrichment/ident-BP_dotplot.png]

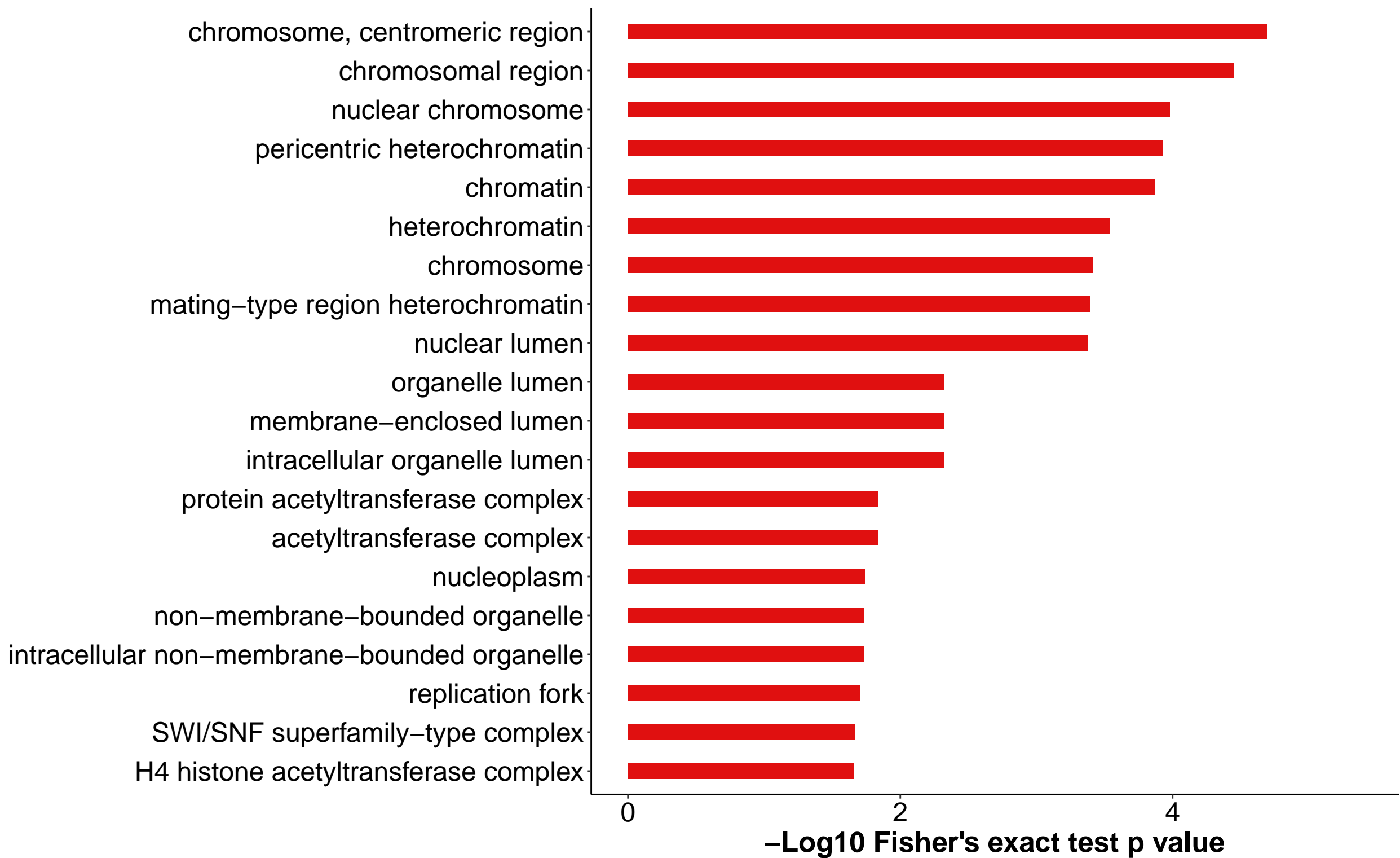

Supplement: Supplemental Information 2 — Supplemental Figures: Figure S1A: Peptide length, peptides per protein, distribution of coverage (%) and MW (kDa) of the LC-MS/MS analysis of rHSA from company A. Figure S2B: Peptide length, peptides per protein, distribution of coverage (%) and MW(kDa) of the LC-MS/MS analysis of rHSA from company B. Figure S3C: Peptide length, peptides per protein, distribution of coverage (%) and MW(kDa) of the LC-MS/MS analysis of pHSA from company C. Figure S4D: Peptide length, peptides per protein, distribution of coverage (%) and MW(kDa) of the LC-MS/MS analysis of pHSA from company D. Figure S5E: Peptide length, peptides per protein, distribution of coverage (%) and MW(kDa) of the LC-MS/MS analysis of pHSA from company E. Figure S6F: Peptide length, peptides per protein, distribution of coverage (%) and MW(kDa) of the LC-MS/MS analysis of pHSA from company F. Figure S7G: Peptide length, peptides per protein, distribution of coverage (%) and MW(kDa) of the LC-MS/MS analysis of pHSA from company G. Figure S8H: Peptide length, peptides per protein, distribution of coverage (%) and MW(kDa) of the LC-MS/MS analysis of pHSA from company H. Figure S9: GO enrichment analysis of the APs in pHSA. Figure S10: Subcellular localization prediction of the APs in pHSA. Figure S11: COG/KOG enrichment analysis of the APs in pHSA. Figure S12: KEGG pathway enrichment analysis of the APs in pHSA. Supplemental Tables: Table S1A: The protein and peptide identified in rHSA from company A. Table S2B: The protein and peptide identified in rHSA from company B. Table S3C: The protein and peptide identified in pHSA from company C. Table S4D: The protein and peptide identified in pHSA from company D. Table S5E: The protein and peptide identified in pHSA from company E. Table S6F: The protein and peptide identified in pHSA from company F. Table S7G: The protein and peptide identified in pHSA from company G. Table S8H: The protein and peptide identified in pHSA from company H. Table S9: The relative abunda [file peerj-13-19624-s002.zip › Supplementary/Supplementary File/Supplementary File1/3-Functional_enrichment/ident-CC_barplot.pdf]

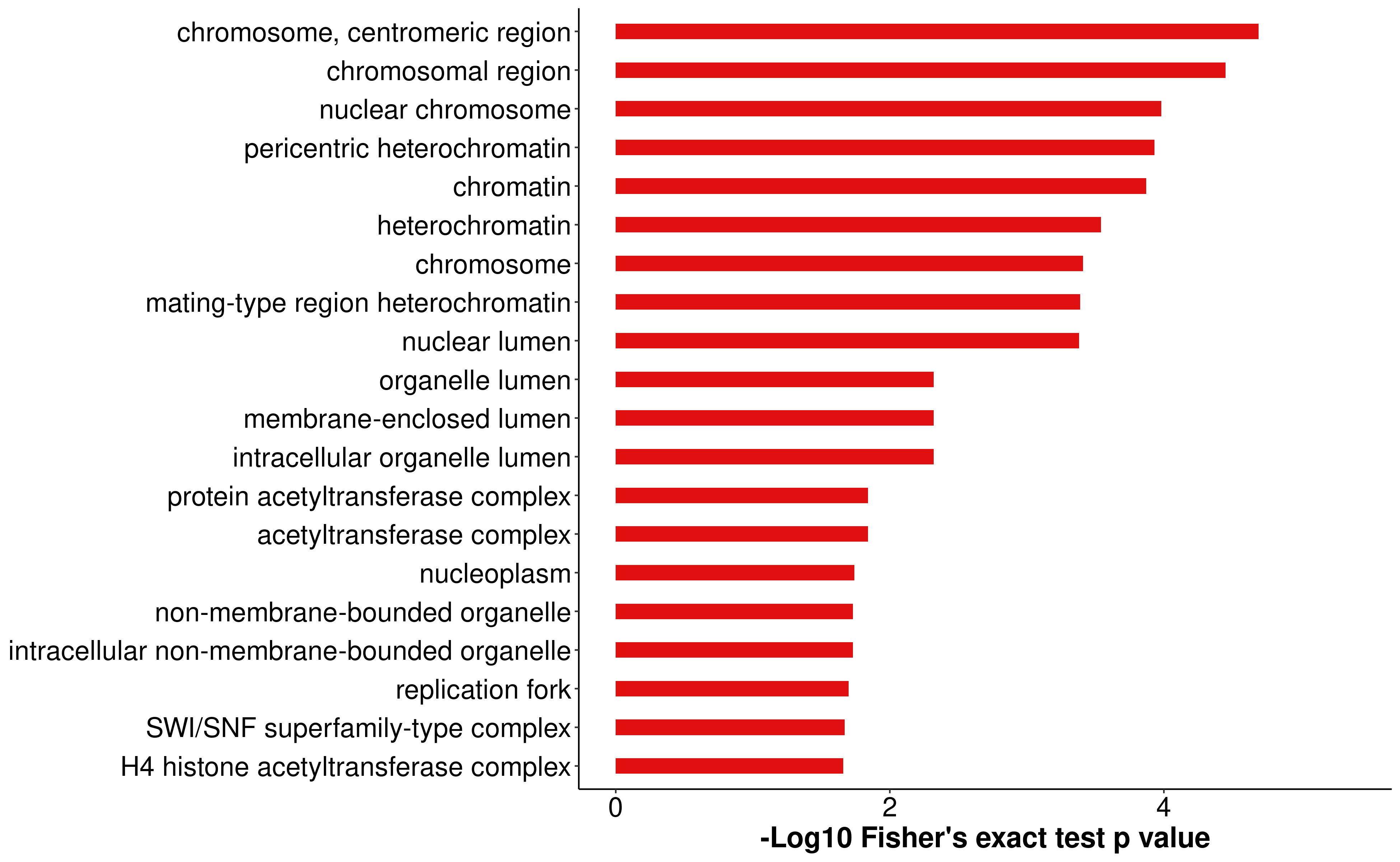

Supplement: Supplemental Information 2 — Supplemental Figures: Figure S1A: Peptide length, peptides per protein, distribution of coverage (%) and MW (kDa) of the LC-MS/MS analysis of rHSA from company A. Figure S2B: Peptide length, peptides per protein, distribution of coverage (%) and MW(kDa) of the LC-MS/MS analysis of rHSA from company B. Figure S3C: Peptide length, peptides per protein, distribution of coverage (%) and MW(kDa) of the LC-MS/MS analysis of pHSA from company C. Figure S4D: Peptide length, peptides per protein, distribution of coverage (%) and MW(kDa) of the LC-MS/MS analysis of pHSA from company D. Figure S5E: Peptide length, peptides per protein, distribution of coverage (%) and MW(kDa) of the LC-MS/MS analysis of pHSA from company E. Figure S6F: Peptide length, peptides per protein, distribution of coverage (%) and MW(kDa) of the LC-MS/MS analysis of pHSA from company F. Figure S7G: Peptide length, peptides per protein, distribution of coverage (%) and MW(kDa) of the LC-MS/MS analysis of pHSA from company G. Figure S8H: Peptide length, peptides per protein, distribution of coverage (%) and MW(kDa) of the LC-MS/MS analysis of pHSA from company H. Figure S9: GO enrichment analysis of the APs in pHSA. Figure S10: Subcellular localization prediction of the APs in pHSA. Figure S11: COG/KOG enrichment analysis of the APs in pHSA. Figure S12: KEGG pathway enrichment analysis of the APs in pHSA. Supplemental Tables: Table S1A: The protein and peptide identified in rHSA from company A. Table S2B: The protein and peptide identified in rHSA from company B. Table S3C: The protein and peptide identified in pHSA from company C. Table S4D: The protein and peptide identified in pHSA from company D. Table S5E: The protein and peptide identified in pHSA from company E. Table S6F: The protein and peptide identified in pHSA from company F. Table S7G: The protein and peptide identified in pHSA from company G. Table S8H: The protein and peptide identified in pHSA from company H. Table S9: The relative abunda [file peerj-13-19624-s002.zip › Supplementary/Supplementary File/Supplementary File1/3-Functional_enrichment/ident-CC_barplot.png]

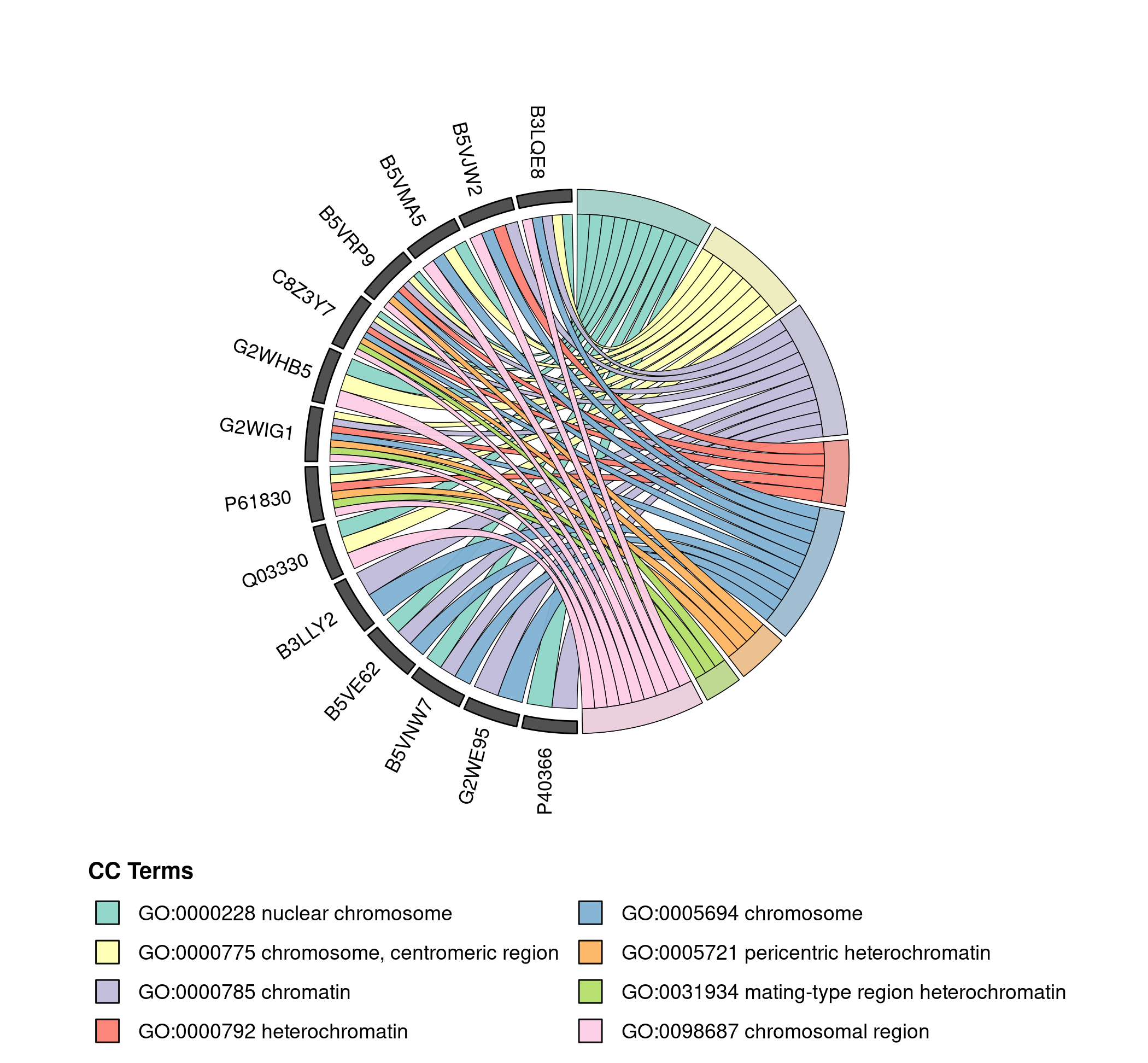

Supplement: Supplemental Information 2 — Supplemental Figures: Figure S1A: Peptide length, peptides per protein, distribution of coverage (%) and MW (kDa) of the LC-MS/MS analysis of rHSA from company A. Figure S2B: Peptide length, peptides per protein, distribution of coverage (%) and MW(kDa) of the LC-MS/MS analysis of rHSA from company B. Figure S3C: Peptide length, peptides per protein, distribution of coverage (%) and MW(kDa) of the LC-MS/MS analysis of pHSA from company C. Figure S4D: Peptide length, peptides per protein, distribution of coverage (%) and MW(kDa) of the LC-MS/MS analysis of pHSA from company D. Figure S5E: Peptide length, peptides per protein, distribution of coverage (%) and MW(kDa) of the LC-MS/MS analysis of pHSA from company E. Figure S6F: Peptide length, peptides per protein, distribution of coverage (%) and MW(kDa) of the LC-MS/MS analysis of pHSA from company F. Figure S7G: Peptide length, peptides per protein, distribution of coverage (%) and MW(kDa) of the LC-MS/MS analysis of pHSA from company G. Figure S8H: Peptide length, peptides per protein, distribution of coverage (%) and MW(kDa) of the LC-MS/MS analysis of pHSA from company H. Figure S9: GO enrichment analysis of the APs in pHSA. Figure S10: Subcellular localization prediction of the APs in pHSA. Figure S11: COG/KOG enrichment analysis of the APs in pHSA. Figure S12: KEGG pathway enrichment analysis of the APs in pHSA. Supplemental Tables: Table S1A: The protein and peptide identified in rHSA from company A. Table S2B: The protein and peptide identified in rHSA from company B. Table S3C: The protein and peptide identified in pHSA from company C. Table S4D: The protein and peptide identified in pHSA from company D. Table S5E: The protein and peptide identified in pHSA from company E. Table S6F: The protein and peptide identified in pHSA from company F. Table S7G: The protein and peptide identified in pHSA from company G. Table S8H: The protein and peptide identified in pHSA from company H. Table S9: The relative abunda [file peerj-13-19624-s002.zip › Supplementary/Supplementary File/Supplementary File1/3-Functional_enrichment/ident-CC_cirplot.png]

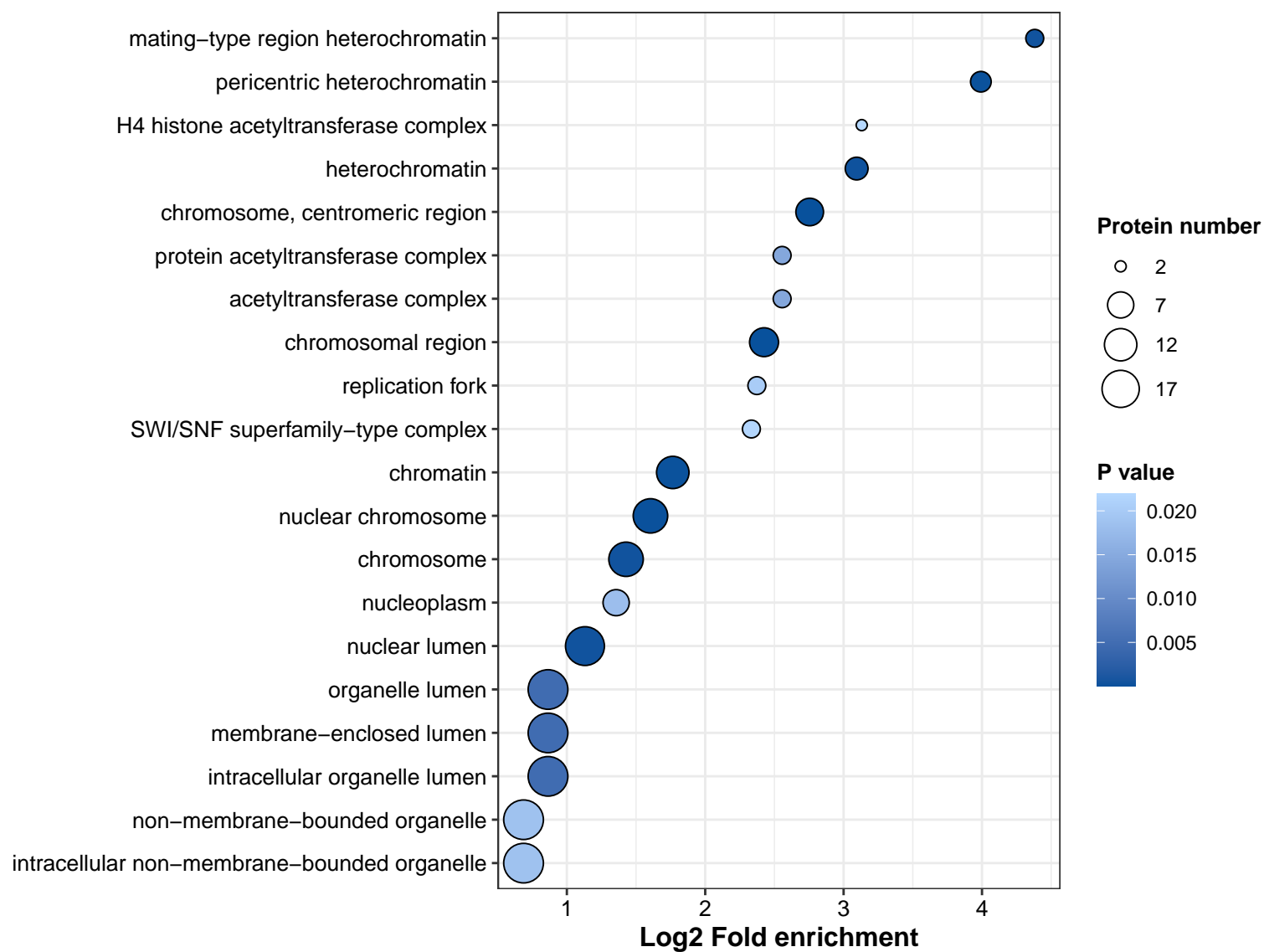

Supplement: Supplemental Information 2 — Supplemental Figures: Figure S1A: Peptide length, peptides per protein, distribution of coverage (%) and MW (kDa) of the LC-MS/MS analysis of rHSA from company A. Figure S2B: Peptide length, peptides per protein, distribution of coverage (%) and MW(kDa) of the LC-MS/MS analysis of rHSA from company B. Figure S3C: Peptide length, peptides per protein, distribution of coverage (%) and MW(kDa) of the LC-MS/MS analysis of pHSA from company C. Figure S4D: Peptide length, peptides per protein, distribution of coverage (%) and MW(kDa) of the LC-MS/MS analysis of pHSA from company D. Figure S5E: Peptide length, peptides per protein, distribution of coverage (%) and MW(kDa) of the LC-MS/MS analysis of pHSA from company E. Figure S6F: Peptide length, peptides per protein, distribution of coverage (%) and MW(kDa) of the LC-MS/MS analysis of pHSA from company F. Figure S7G: Peptide length, peptides per protein, distribution of coverage (%) and MW(kDa) of the LC-MS/MS analysis of pHSA from company G. Figure S8H: Peptide length, peptides per protein, distribution of coverage (%) and MW(kDa) of the LC-MS/MS analysis of pHSA from company H. Figure S9: GO enrichment analysis of the APs in pHSA. Figure S10: Subcellular localization prediction of the APs in pHSA. Figure S11: COG/KOG enrichment analysis of the APs in pHSA. Figure S12: KEGG pathway enrichment analysis of the APs in pHSA. Supplemental Tables: Table S1A: The protein and peptide identified in rHSA from company A. Table S2B: The protein and peptide identified in rHSA from company B. Table S3C: The protein and peptide identified in pHSA from company C. Table S4D: The protein and peptide identified in pHSA from company D. Table S5E: The protein and peptide identified in pHSA from company E. Table S6F: The protein and peptide identified in pHSA from company F. Table S7G: The protein and peptide identified in pHSA from company G. Table S8H: The protein and peptide identified in pHSA from company H. Table S9: The relative abunda [file peerj-13-19624-s002.zip › Supplementary/Supplementary File/Supplementary File1/3-Functional_enrichment/ident-CC_dotplot.pdf]

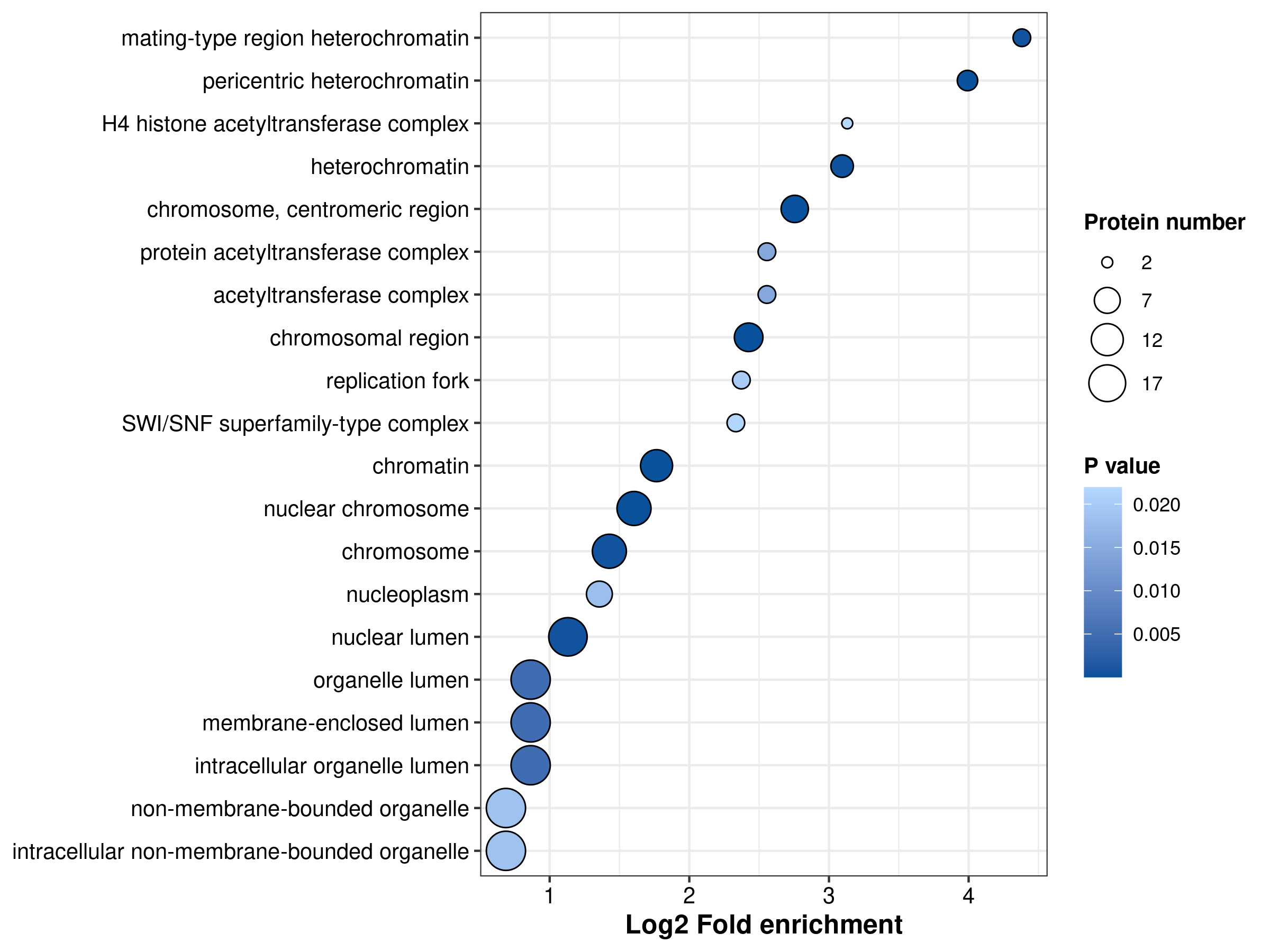

Supplement: Supplemental Information 2 — Supplemental Figures: Figure S1A: Peptide length, peptides per protein, distribution of coverage (%) and MW (kDa) of the LC-MS/MS analysis of rHSA from company A. Figure S2B: Peptide length, peptides per protein, distribution of coverage (%) and MW(kDa) of the LC-MS/MS analysis of rHSA from company B. Figure S3C: Peptide length, peptides per protein, distribution of coverage (%) and MW(kDa) of the LC-MS/MS analysis of pHSA from company C. Figure S4D: Peptide length, peptides per protein, distribution of coverage (%) and MW(kDa) of the LC-MS/MS analysis of pHSA from company D. Figure S5E: Peptide length, peptides per protein, distribution of coverage (%) and MW(kDa) of the LC-MS/MS analysis of pHSA from company E. Figure S6F: Peptide length, peptides per protein, distribution of coverage (%) and MW(kDa) of the LC-MS/MS analysis of pHSA from company F. Figure S7G: Peptide length, peptides per protein, distribution of coverage (%) and MW(kDa) of the LC-MS/MS analysis of pHSA from company G. Figure S8H: Peptide length, peptides per protein, distribution of coverage (%) and MW(kDa) of the LC-MS/MS analysis of pHSA from company H. Figure S9: GO enrichment analysis of the APs in pHSA. Figure S10: Subcellular localization prediction of the APs in pHSA. Figure S11: COG/KOG enrichment analysis of the APs in pHSA. Figure S12: KEGG pathway enrichment analysis of the APs in pHSA. Supplemental Tables: Table S1A: The protein and peptide identified in rHSA from company A. Table S2B: The protein and peptide identified in rHSA from company B. Table S3C: The protein and peptide identified in pHSA from company C. Table S4D: The protein and peptide identified in pHSA from company D. Table S5E: The protein and peptide identified in pHSA from company E. Table S6F: The protein and peptide identified in pHSA from company F. Table S7G: The protein and peptide identified in pHSA from company G. Table S8H: The protein and peptide identified in pHSA from company H. Table S9: The relative abunda [file peerj-13-19624-s002.zip › Supplementary/Supplementary File/Supplementary File1/3-Functional_enrichment/ident-CC_dotplot.png]

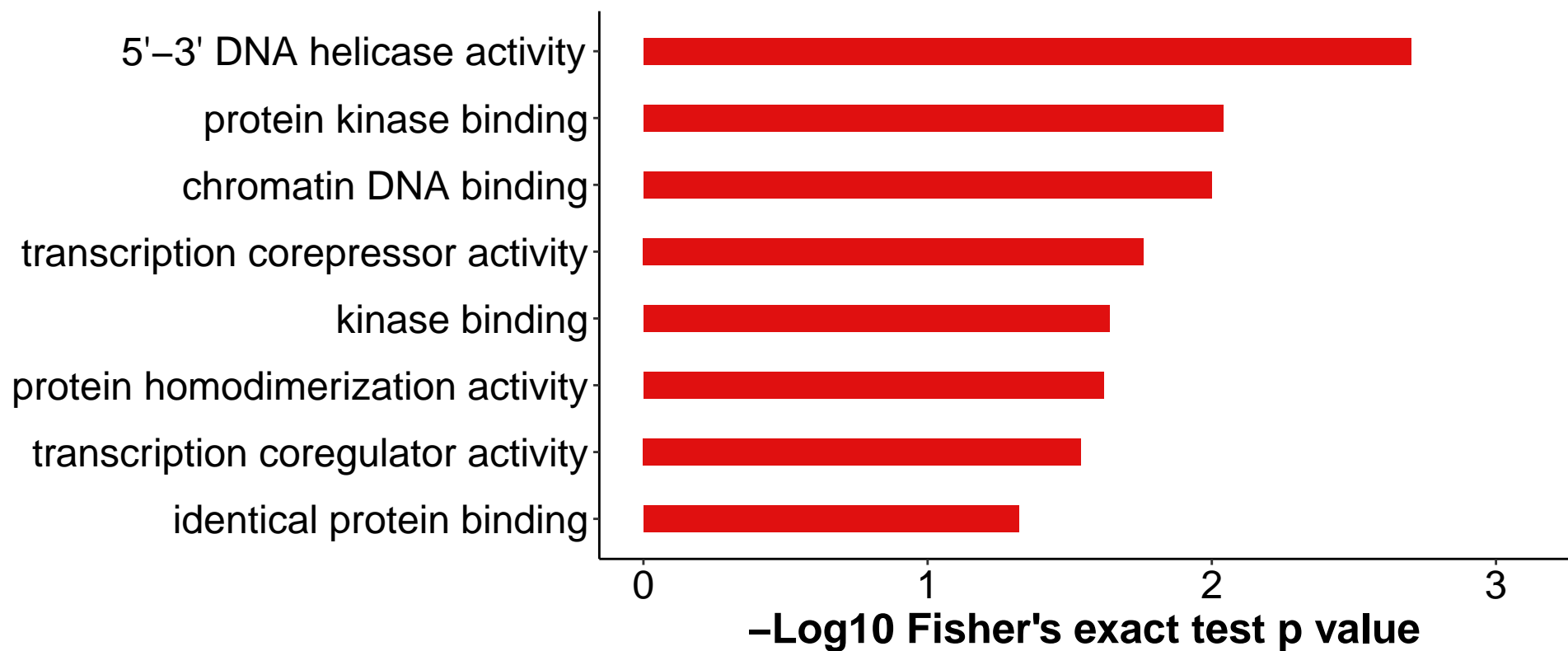

Supplement: Supplemental Information 2 — Supplemental Figures: Figure S1A: Peptide length, peptides per protein, distribution of coverage (%) and MW (kDa) of the LC-MS/MS analysis of rHSA from company A. Figure S2B: Peptide length, peptides per protein, distribution of coverage (%) and MW(kDa) of the LC-MS/MS analysis of rHSA from company B. Figure S3C: Peptide length, peptides per protein, distribution of coverage (%) and MW(kDa) of the LC-MS/MS analysis of pHSA from company C. Figure S4D: Peptide length, peptides per protein, distribution of coverage (%) and MW(kDa) of the LC-MS/MS analysis of pHSA from company D. Figure S5E: Peptide length, peptides per protein, distribution of coverage (%) and MW(kDa) of the LC-MS/MS analysis of pHSA from company E. Figure S6F: Peptide length, peptides per protein, distribution of coverage (%) and MW(kDa) of the LC-MS/MS analysis of pHSA from company F. Figure S7G: Peptide length, peptides per protein, distribution of coverage (%) and MW(kDa) of the LC-MS/MS analysis of pHSA from company G. Figure S8H: Peptide length, peptides per protein, distribution of coverage (%) and MW(kDa) of the LC-MS/MS analysis of pHSA from company H. Figure S9: GO enrichment analysis of the APs in pHSA. Figure S10: Subcellular localization prediction of the APs in pHSA. Figure S11: COG/KOG enrichment analysis of the APs in pHSA. Figure S12: KEGG pathway enrichment analysis of the APs in pHSA. Supplemental Tables: Table S1A: The protein and peptide identified in rHSA from company A. Table S2B: The protein and peptide identified in rHSA from company B. Table S3C: The protein and peptide identified in pHSA from company C. Table S4D: The protein and peptide identified in pHSA from company D. Table S5E: The protein and peptide identified in pHSA from company E. Table S6F: The protein and peptide identified in pHSA from company F. Table S7G: The protein and peptide identified in pHSA from company G. Table S8H: The protein and peptide identified in pHSA from company H. Table S9: The relative abunda [file peerj-13-19624-s002.zip › Supplementary/Supplementary File/Supplementary File1/3-Functional_enrichment/ident-MF_barplot.pdf]

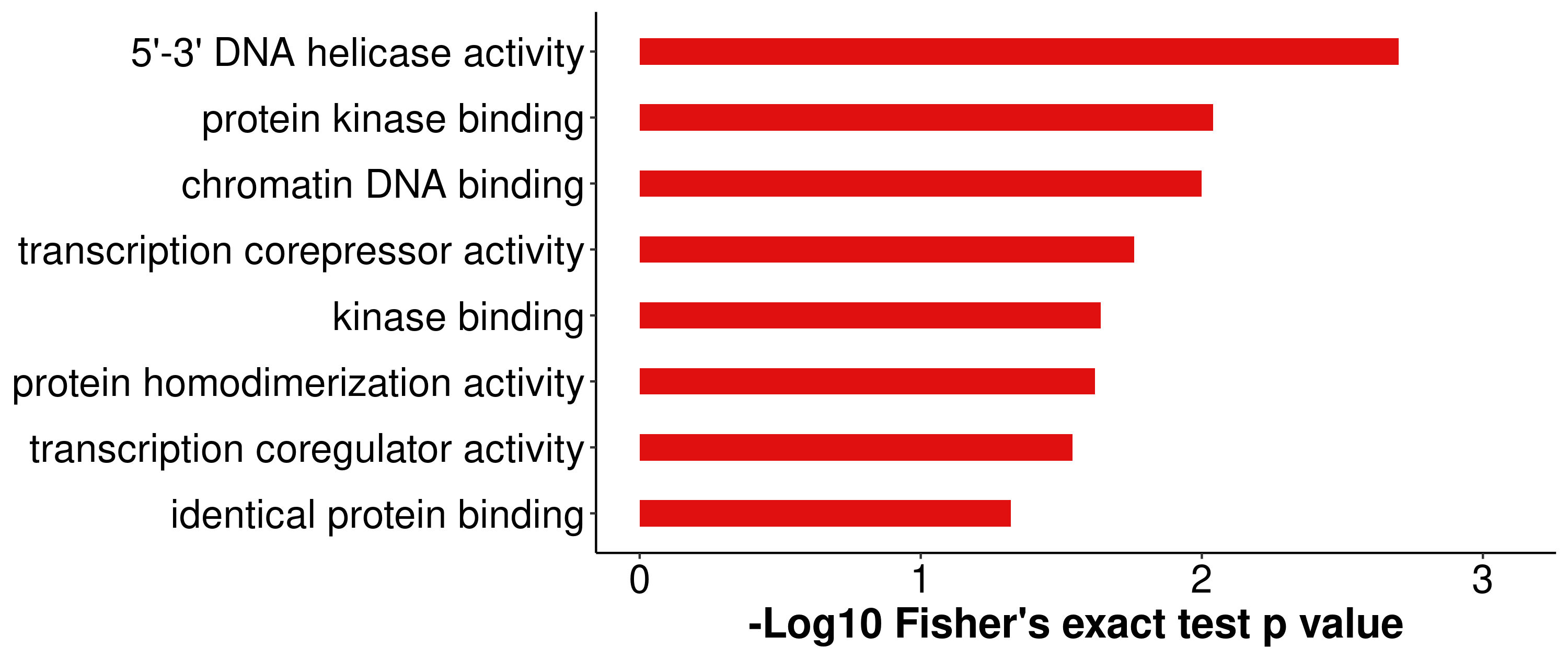

Supplement: Supplemental Information 2 — Supplemental Figures: Figure S1A: Peptide length, peptides per protein, distribution of coverage (%) and MW (kDa) of the LC-MS/MS analysis of rHSA from company A. Figure S2B: Peptide length, peptides per protein, distribution of coverage (%) and MW(kDa) of the LC-MS/MS analysis of rHSA from company B. Figure S3C: Peptide length, peptides per protein, distribution of coverage (%) and MW(kDa) of the LC-MS/MS analysis of pHSA from company C. Figure S4D: Peptide length, peptides per protein, distribution of coverage (%) and MW(kDa) of the LC-MS/MS analysis of pHSA from company D. Figure S5E: Peptide length, peptides per protein, distribution of coverage (%) and MW(kDa) of the LC-MS/MS analysis of pHSA from company E. Figure S6F: Peptide length, peptides per protein, distribution of coverage (%) and MW(kDa) of the LC-MS/MS analysis of pHSA from company F. Figure S7G: Peptide length, peptides per protein, distribution of coverage (%) and MW(kDa) of the LC-MS/MS analysis of pHSA from company G. Figure S8H: Peptide length, peptides per protein, distribution of coverage (%) and MW(kDa) of the LC-MS/MS analysis of pHSA from company H. Figure S9: GO enrichment analysis of the APs in pHSA. Figure S10: Subcellular localization prediction of the APs in pHSA. Figure S11: COG/KOG enrichment analysis of the APs in pHSA. Figure S12: KEGG pathway enrichment analysis of the APs in pHSA. Supplemental Tables: Table S1A: The protein and peptide identified in rHSA from company A. Table S2B: The protein and peptide identified in rHSA from company B. Table S3C: The protein and peptide identified in pHSA from company C. Table S4D: The protein and peptide identified in pHSA from company D. Table S5E: The protein and peptide identified in pHSA from company E. Table S6F: The protein and peptide identified in pHSA from company F. Table S7G: The protein and peptide identified in pHSA from company G. Table S8H: The protein and peptide identified in pHSA from company H. Table S9: The relative abunda [file peerj-13-19624-s002.zip › Supplementary/Supplementary File/Supplementary File1/3-Functional_enrichment/ident-MF_barplot.png]

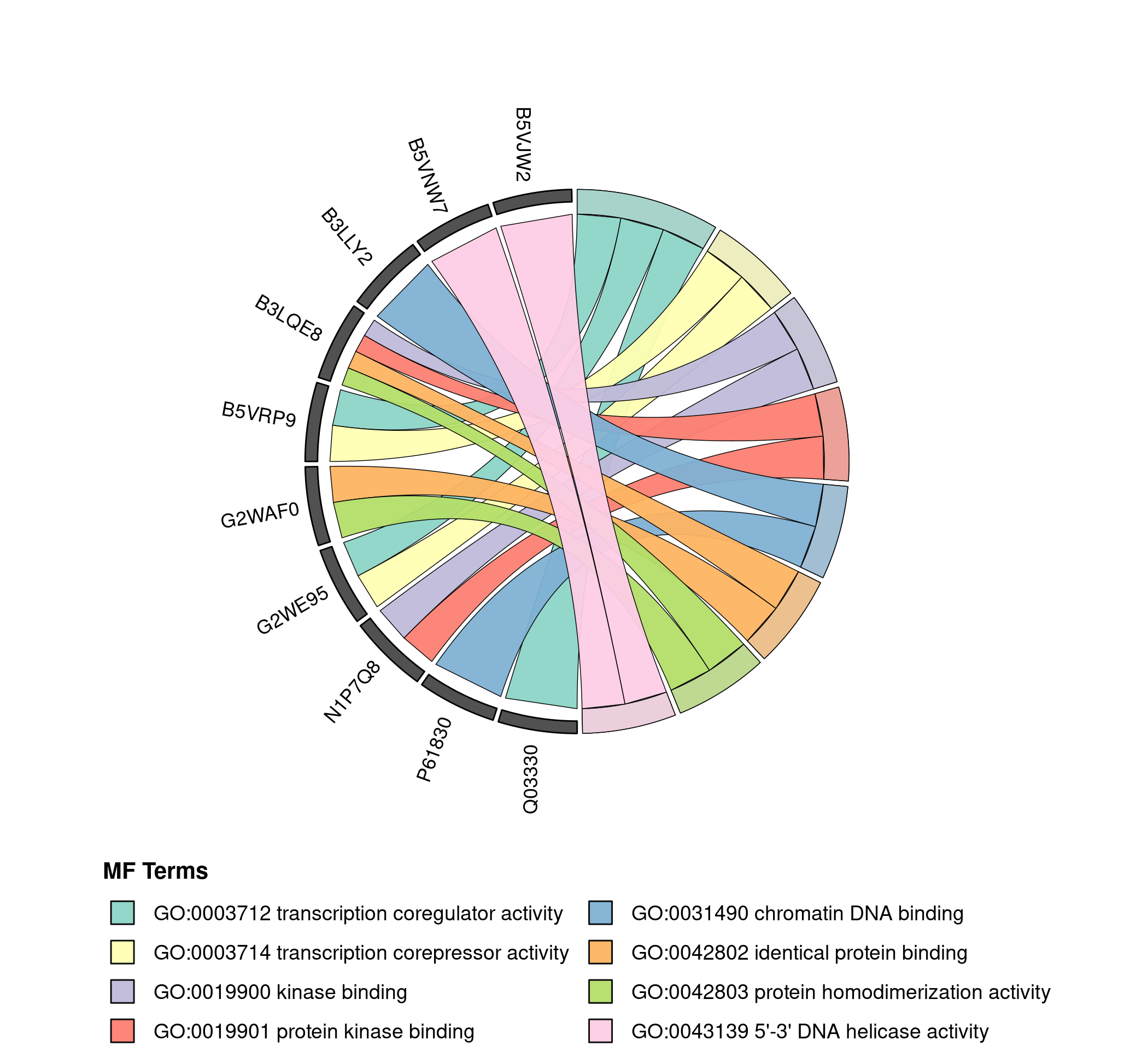

Supplement: Supplemental Information 2 — Supplemental Figures: Figure S1A: Peptide length, peptides per protein, distribution of coverage (%) and MW (kDa) of the LC-MS/MS analysis of rHSA from company A. Figure S2B: Peptide length, peptides per protein, distribution of coverage (%) and MW(kDa) of the LC-MS/MS analysis of rHSA from company B. Figure S3C: Peptide length, peptides per protein, distribution of coverage (%) and MW(kDa) of the LC-MS/MS analysis of pHSA from company C. Figure S4D: Peptide length, peptides per protein, distribution of coverage (%) and MW(kDa) of the LC-MS/MS analysis of pHSA from company D. Figure S5E: Peptide length, peptides per protein, distribution of coverage (%) and MW(kDa) of the LC-MS/MS analysis of pHSA from company E. Figure S6F: Peptide length, peptides per protein, distribution of coverage (%) and MW(kDa) of the LC-MS/MS analysis of pHSA from company F. Figure S7G: Peptide length, peptides per protein, distribution of coverage (%) and MW(kDa) of the LC-MS/MS analysis of pHSA from company G. Figure S8H: Peptide length, peptides per protein, distribution of coverage (%) and MW(kDa) of the LC-MS/MS analysis of pHSA from company H. Figure S9: GO enrichment analysis of the APs in pHSA. Figure S10: Subcellular localization prediction of the APs in pHSA. Figure S11: COG/KOG enrichment analysis of the APs in pHSA. Figure S12: KEGG pathway enrichment analysis of the APs in pHSA. Supplemental Tables: Table S1A: The protein and peptide identified in rHSA from company A. Table S2B: The protein and peptide identified in rHSA from company B. Table S3C: The protein and peptide identified in pHSA from company C. Table S4D: The protein and peptide identified in pHSA from company D. Table S5E: The protein and peptide identified in pHSA from company E. Table S6F: The protein and peptide identified in pHSA from company F. Table S7G: The protein and peptide identified in pHSA from company G. Table S8H: The protein and peptide identified in pHSA from company H. Table S9: The relative abunda [file peerj-13-19624-s002.zip › Supplementary/Supplementary File/Supplementary File1/3-Functional_enrichment/ident-MF_cirplot.png]

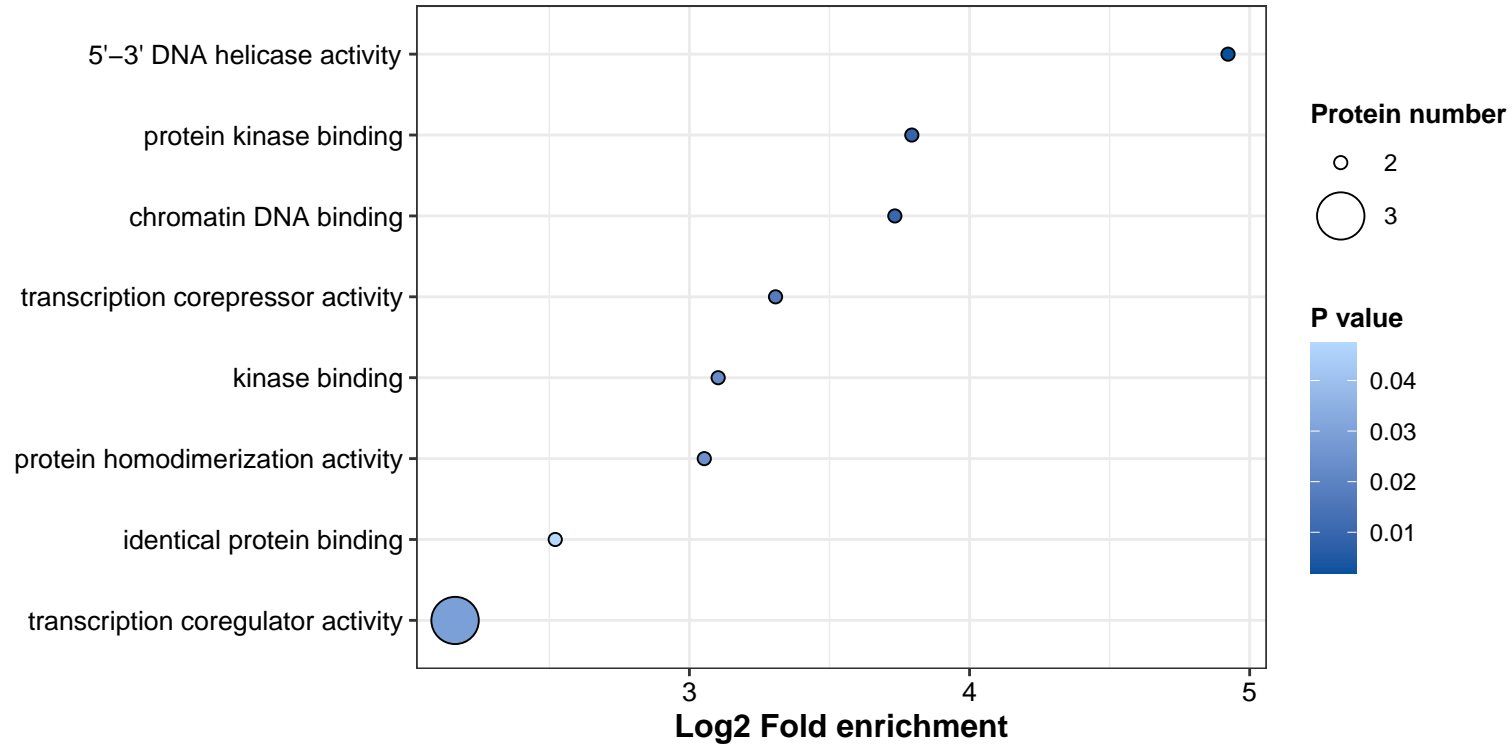

Supplement: Supplemental Information 2 — Supplemental Figures: Figure S1A: Peptide length, peptides per protein, distribution of coverage (%) and MW (kDa) of the LC-MS/MS analysis of rHSA from company A. Figure S2B: Peptide length, peptides per protein, distribution of coverage (%) and MW(kDa) of the LC-MS/MS analysis of rHSA from company B. Figure S3C: Peptide length, peptides per protein, distribution of coverage (%) and MW(kDa) of the LC-MS/MS analysis of pHSA from company C. Figure S4D: Peptide length, peptides per protein, distribution of coverage (%) and MW(kDa) of the LC-MS/MS analysis of pHSA from company D. Figure S5E: Peptide length, peptides per protein, distribution of coverage (%) and MW(kDa) of the LC-MS/MS analysis of pHSA from company E. Figure S6F: Peptide length, peptides per protein, distribution of coverage (%) and MW(kDa) of the LC-MS/MS analysis of pHSA from company F. Figure S7G: Peptide length, peptides per protein, distribution of coverage (%) and MW(kDa) of the LC-MS/MS analysis of pHSA from company G. Figure S8H: Peptide length, peptides per protein, distribution of coverage (%) and MW(kDa) of the LC-MS/MS analysis of pHSA from company H. Figure S9: GO enrichment analysis of the APs in pHSA. Figure S10: Subcellular localization prediction of the APs in pHSA. Figure S11: COG/KOG enrichment analysis of the APs in pHSA. Figure S12: KEGG pathway enrichment analysis of the APs in pHSA. Supplemental Tables: Table S1A: The protein and peptide identified in rHSA from company A. Table S2B: The protein and peptide identified in rHSA from company B. Table S3C: The protein and peptide identified in pHSA from company C. Table S4D: The protein and peptide identified in pHSA from company D. Table S5E: The protein and peptide identified in pHSA from company E. Table S6F: The protein and peptide identified in pHSA from company F. Table S7G: The protein and peptide identified in pHSA from company G. Table S8H: The protein and peptide identified in pHSA from company H. Table S9: The relative abunda [file peerj-13-19624-s002.zip › Supplementary/Supplementary File/Supplementary File1/3-Functional_enrichment/ident-MF_dotplot.pdf]

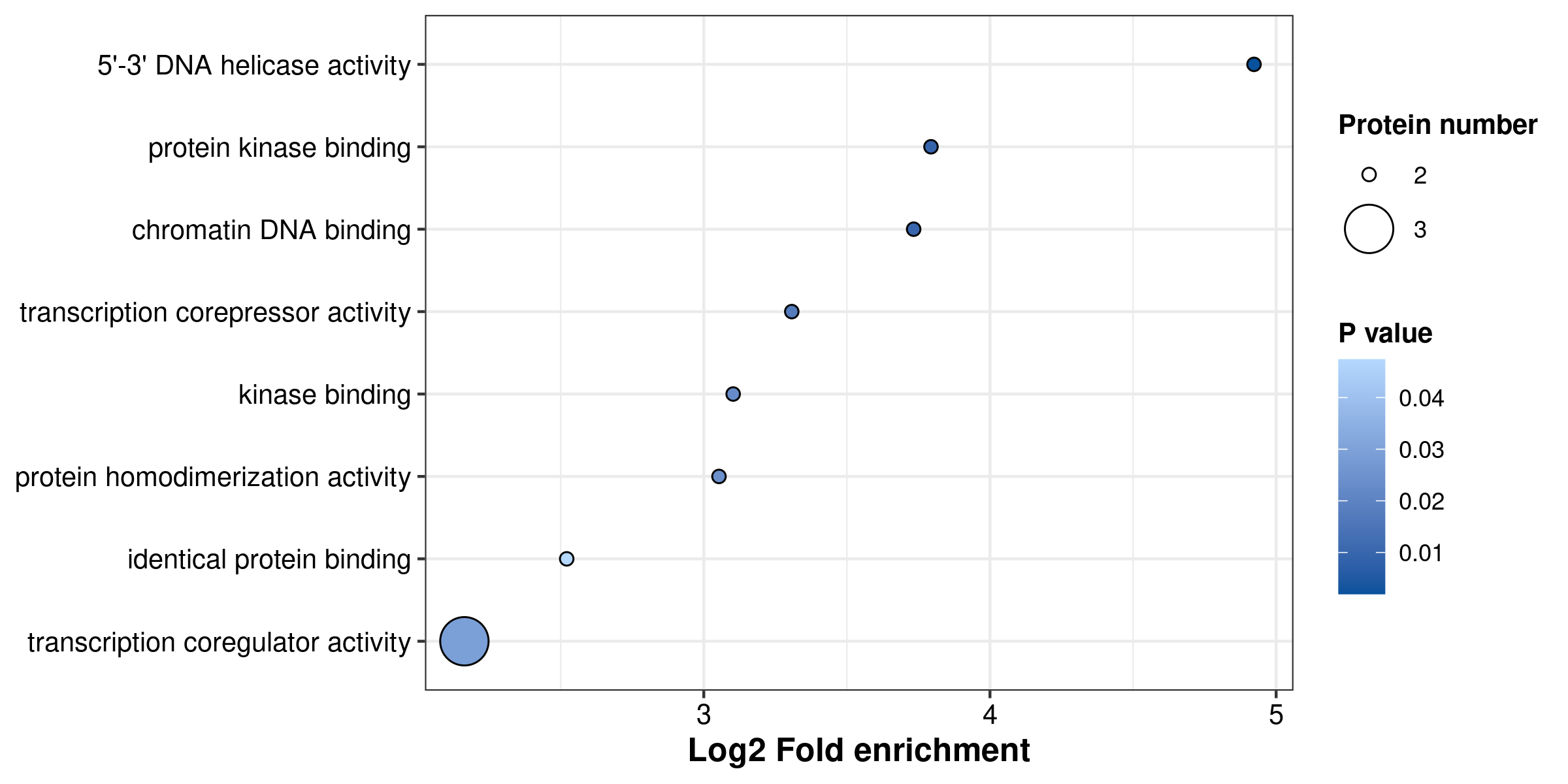

Supplement: Supplemental Information 2 — Supplemental Figures: Figure S1A: Peptide length, peptides per protein, distribution of coverage (%) and MW (kDa) of the LC-MS/MS analysis of rHSA from company A. Figure S2B: Peptide length, peptides per protein, distribution of coverage (%) and MW(kDa) of the LC-MS/MS analysis of rHSA from company B. Figure S3C: Peptide length, peptides per protein, distribution of coverage (%) and MW(kDa) of the LC-MS/MS analysis of pHSA from company C. Figure S4D: Peptide length, peptides per protein, distribution of coverage (%) and MW(kDa) of the LC-MS/MS analysis of pHSA from company D. Figure S5E: Peptide length, peptides per protein, distribution of coverage (%) and MW(kDa) of the LC-MS/MS analysis of pHSA from company E. Figure S6F: Peptide length, peptides per protein, distribution of coverage (%) and MW(kDa) of the LC-MS/MS analysis of pHSA from company F. Figure S7G: Peptide length, peptides per protein, distribution of coverage (%) and MW(kDa) of the LC-MS/MS analysis of pHSA from company G. Figure S8H: Peptide length, peptides per protein, distribution of coverage (%) and MW(kDa) of the LC-MS/MS analysis of pHSA from company H. Figure S9: GO enrichment analysis of the APs in pHSA. Figure S10: Subcellular localization prediction of the APs in pHSA. Figure S11: COG/KOG enrichment analysis of the APs in pHSA. Figure S12: KEGG pathway enrichment analysis of the APs in pHSA. Supplemental Tables: Table S1A: The protein and peptide identified in rHSA from company A. Table S2B: The protein and peptide identified in rHSA from company B. Table S3C: The protein and peptide identified in pHSA from company C. Table S4D: The protein and peptide identified in pHSA from company D. Table S5E: The protein and peptide identified in pHSA from company E. Table S6F: The protein and peptide identified in pHSA from company F. Table S7G: The protein and peptide identified in pHSA from company G. Table S8H: The protein and peptide identified in pHSA from company H. Table S9: The relative abunda [file peerj-13-19624-s002.zip › Supplementary/Supplementary File/Supplementary File1/3-Functional_enrichment/ident-MF_dotplot.png]

map00230 Purine metabolism

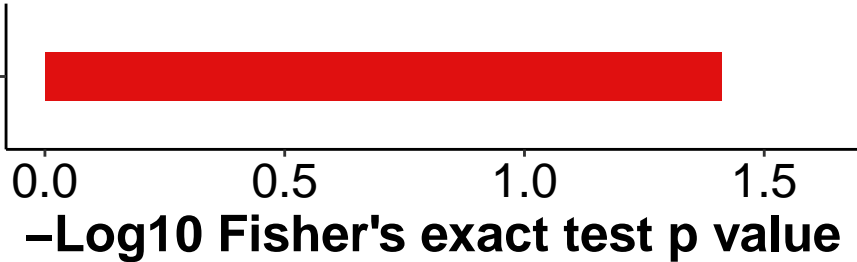

Supplement: Supplemental Information 2 — Supplemental Figures: Figure S1A: Peptide length, peptides per protein, distribution of coverage (%) and MW (kDa) of the LC-MS/MS analysis of rHSA from company A. Figure S2B: Peptide length, peptides per protein, distribution of coverage (%) and MW(kDa) of the LC-MS/MS analysis of rHSA from company B. Figure S3C: Peptide length, peptides per protein, distribution of coverage (%) and MW(kDa) of the LC-MS/MS analysis of pHSA from company C. Figure S4D: Peptide length, peptides per protein, distribution of coverage (%) and MW(kDa) of the LC-MS/MS analysis of pHSA from company D. Figure S5E: Peptide length, peptides per protein, distribution of coverage (%) and MW(kDa) of the LC-MS/MS analysis of pHSA from company E. Figure S6F: Peptide length, peptides per protein, distribution of coverage (%) and MW(kDa) of the LC-MS/MS analysis of pHSA from company F. Figure S7G: Peptide length, peptides per protein, distribution of coverage (%) and MW(kDa) of the LC-MS/MS analysis of pHSA from company G. Figure S8H: Peptide length, peptides per protein, distribution of coverage (%) and MW(kDa) of the LC-MS/MS analysis of pHSA from company H. Figure S9: GO enrichment analysis of the APs in pHSA. Figure S10: Subcellular localization prediction of the APs in pHSA. Figure S11: COG/KOG enrichment analysis of the APs in pHSA. Figure S12: KEGG pathway enrichment analysis of the APs in pHSA. Supplemental Tables: Table S1A: The protein and peptide identified in rHSA from company A. Table S2B: The protein and peptide identified in rHSA from company B. Table S3C: The protein and peptide identified in pHSA from company C. Table S4D: The protein and peptide identified in pHSA from company D. Table S5E: The protein and peptide identified in pHSA from company E. Table S6F: The protein and peptide identified in pHSA from company F. Table S7G: The protein and peptide identified in pHSA from company G. Table S8H: The protein and peptide identified in pHSA from company H. Table S9: The relative abunda [file peerj-13-19624-s002.zip › Supplementary/Supplementary File/Supplementary File1/3-Functional_enrichment/ident-kegg_barplot.pdf]

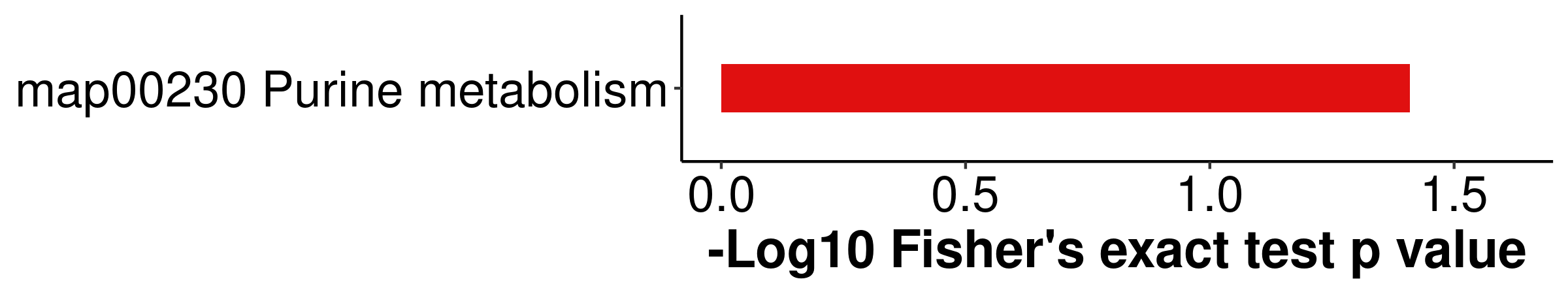

Supplement: Supplemental Information 2 — Supplemental Figures: Figure S1A: Peptide length, peptides per protein, distribution of coverage (%) and MW (kDa) of the LC-MS/MS analysis of rHSA from company A. Figure S2B: Peptide length, peptides per protein, distribution of coverage (%) and MW(kDa) of the LC-MS/MS analysis of rHSA from company B. Figure S3C: Peptide length, peptides per protein, distribution of coverage (%) and MW(kDa) of the LC-MS/MS analysis of pHSA from company C. Figure S4D: Peptide length, peptides per protein, distribution of coverage (%) and MW(kDa) of the LC-MS/MS analysis of pHSA from company D. Figure S5E: Peptide length, peptides per protein, distribution of coverage (%) and MW(kDa) of the LC-MS/MS analysis of pHSA from company E. Figure S6F: Peptide length, peptides per protein, distribution of coverage (%) and MW(kDa) of the LC-MS/MS analysis of pHSA from company F. Figure S7G: Peptide length, peptides per protein, distribution of coverage (%) and MW(kDa) of the LC-MS/MS analysis of pHSA from company G. Figure S8H: Peptide length, peptides per protein, distribution of coverage (%) and MW(kDa) of the LC-MS/MS analysis of pHSA from company H. Figure S9: GO enrichment analysis of the APs in pHSA. Figure S10: Subcellular localization prediction of the APs in pHSA. Figure S11: COG/KOG enrichment analysis of the APs in pHSA. Figure S12: KEGG pathway enrichment analysis of the APs in pHSA. Supplemental Tables: Table S1A: The protein and peptide identified in rHSA from company A. Table S2B: The protein and peptide identified in rHSA from company B. Table S3C: The protein and peptide identified in pHSA from company C. Table S4D: The protein and peptide identified in pHSA from company D. Table S5E: The protein and peptide identified in pHSA from company E. Table S6F: The protein and peptide identified in pHSA from company F. Table S7G: The protein and peptide identified in pHSA from company G. Table S8H: The protein and peptide identified in pHSA from company H. Table S9: The relative abunda [file peerj-13-19624-s002.zip › Supplementary/Supplementary File/Supplementary File1/3-Functional_enrichment/ident-kegg_barplot.png]

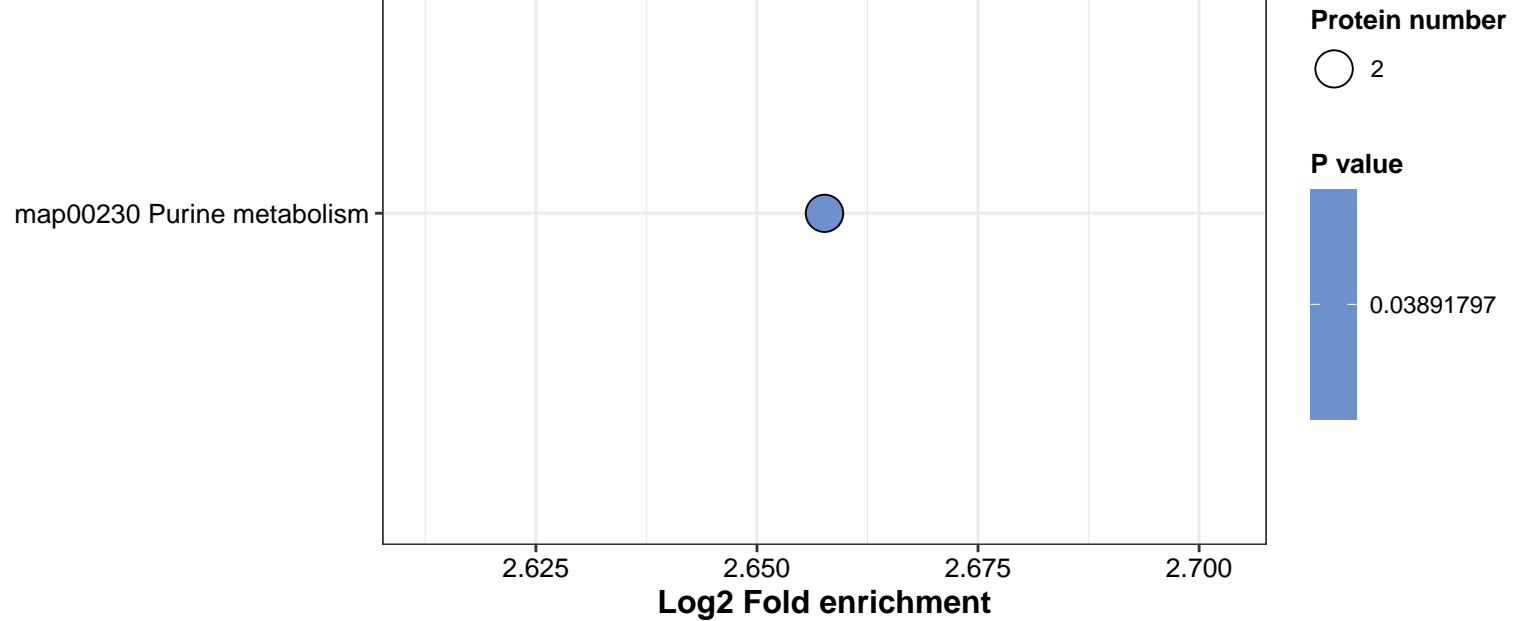

Supplement: Supplemental Information 2 — Supplemental Figures: Figure S1A: Peptide length, peptides per protein, distribution of coverage (%) and MW (kDa) of the LC-MS/MS analysis of rHSA from company A. Figure S2B: Peptide length, peptides per protein, distribution of coverage (%) and MW(kDa) of the LC-MS/MS analysis of rHSA from company B. Figure S3C: Peptide length, peptides per protein, distribution of coverage (%) and MW(kDa) of the LC-MS/MS analysis of pHSA from company C. Figure S4D: Peptide length, peptides per protein, distribution of coverage (%) and MW(kDa) of the LC-MS/MS analysis of pHSA from company D. Figure S5E: Peptide length, peptides per protein, distribution of coverage (%) and MW(kDa) of the LC-MS/MS analysis of pHSA from company E. Figure S6F: Peptide length, peptides per protein, distribution of coverage (%) and MW(kDa) of the LC-MS/MS analysis of pHSA from company F. Figure S7G: Peptide length, peptides per protein, distribution of coverage (%) and MW(kDa) of the LC-MS/MS analysis of pHSA from company G. Figure S8H: Peptide length, peptides per protein, distribution of coverage (%) and MW(kDa) of the LC-MS/MS analysis of pHSA from company H. Figure S9: GO enrichment analysis of the APs in pHSA. Figure S10: Subcellular localization prediction of the APs in pHSA. Figure S11: COG/KOG enrichment analysis of the APs in pHSA. Figure S12: KEGG pathway enrichment analysis of the APs in pHSA. Supplemental Tables: Table S1A: The protein and peptide identified in rHSA from company A. Table S2B: The protein and peptide identified in rHSA from company B. Table S3C: The protein and peptide identified in pHSA from company C. Table S4D: The protein and peptide identified in pHSA from company D. Table S5E: The protein and peptide identified in pHSA from company E. Table S6F: The protein and peptide identified in pHSA from company F. Table S7G: The protein and peptide identified in pHSA from company G. Table S8H: The protein and peptide identified in pHSA from company H. Table S9: The relative abunda [file peerj-13-19624-s002.zip › Supplementary/Supplementary File/Supplementary File1/3-Functional_enrichment/ident-kegg_dotplot.pdf]

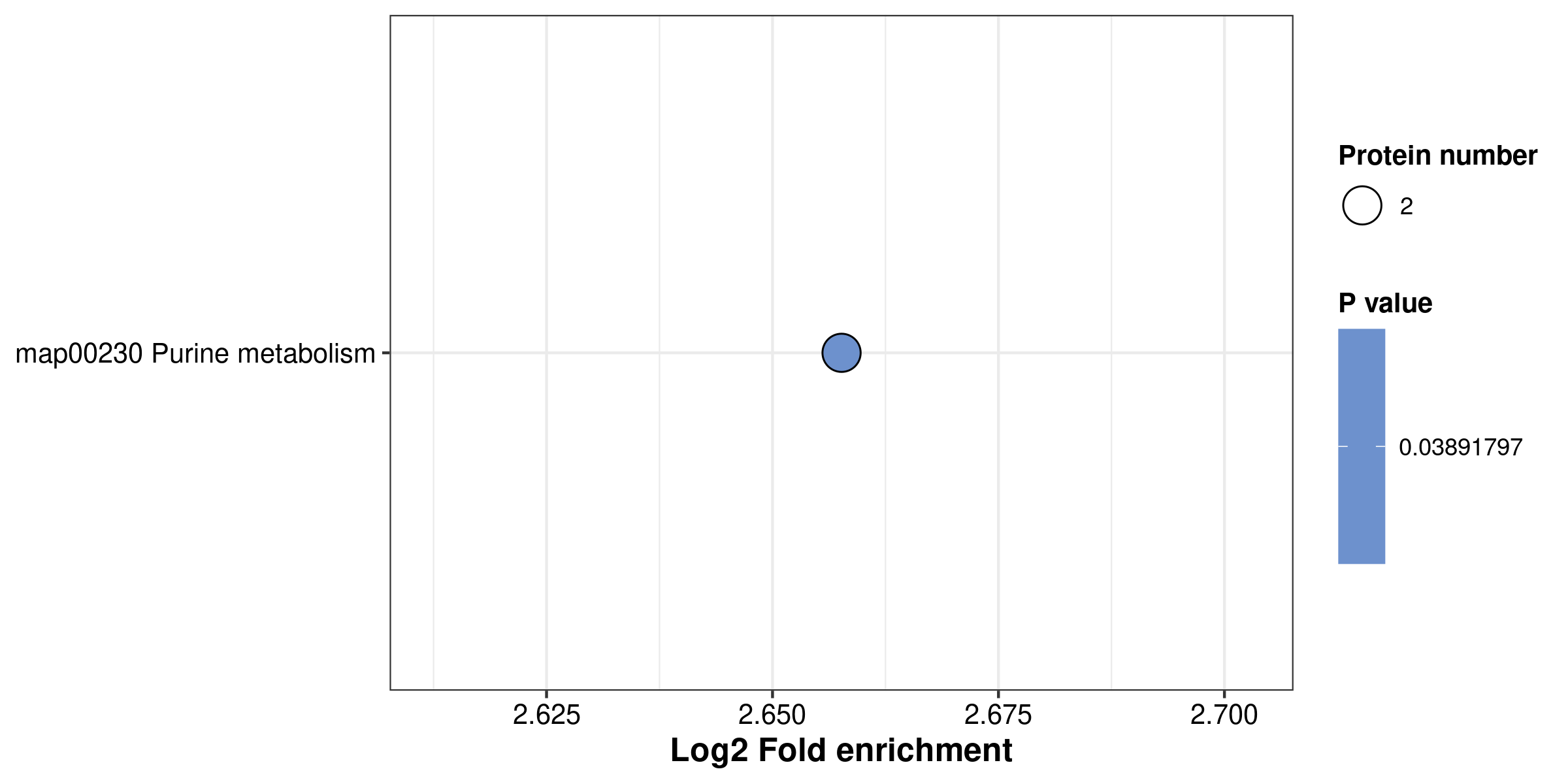

Supplement: Supplemental Information 2 — Supplemental Figures: Figure S1A: Peptide length, peptides per protein, distribution of coverage (%) and MW (kDa) of the LC-MS/MS analysis of rHSA from company A. Figure S2B: Peptide length, peptides per protein, distribution of coverage (%) and MW(kDa) of the LC-MS/MS analysis of rHSA from company B. Figure S3C: Peptide length, peptides per protein, distribution of coverage (%) and MW(kDa) of the LC-MS/MS analysis of pHSA from company C. Figure S4D: Peptide length, peptides per protein, distribution of coverage (%) and MW(kDa) of the LC-MS/MS analysis of pHSA from company D. Figure S5E: Peptide length, peptides per protein, distribution of coverage (%) and MW(kDa) of the LC-MS/MS analysis of pHSA from company E. Figure S6F: Peptide length, peptides per protein, distribution of coverage (%) and MW(kDa) of the LC-MS/MS analysis of pHSA from company F. Figure S7G: Peptide length, peptides per protein, distribution of coverage (%) and MW(kDa) of the LC-MS/MS analysis of pHSA from company G. Figure S8H: Peptide length, peptides per protein, distribution of coverage (%) and MW(kDa) of the LC-MS/MS analysis of pHSA from company H. Figure S9: GO enrichment analysis of the APs in pHSA. Figure S10: Subcellular localization prediction of the APs in pHSA. Figure S11: COG/KOG enrichment analysis of the APs in pHSA. Figure S12: KEGG pathway enrichment analysis of the APs in pHSA. Supplemental Tables: Table S1A: The protein and peptide identified in rHSA from company A. Table S2B: The protein and peptide identified in rHSA from company B. Table S3C: The protein and peptide identified in pHSA from company C. Table S4D: The protein and peptide identified in pHSA from company D. Table S5E: The protein and peptide identified in pHSA from company E. Table S6F: The protein and peptide identified in pHSA from company F. Table S7G: The protein and peptide identified in pHSA from company G. Table S8H: The protein and peptide identified in pHSA from company H. Table S9: The relative abunda [file peerj-13-19624-s002.zip › Supplementary/Supplementary File/Supplementary File1/3-Functional_enrichment/ident-kegg_dotplot.png]

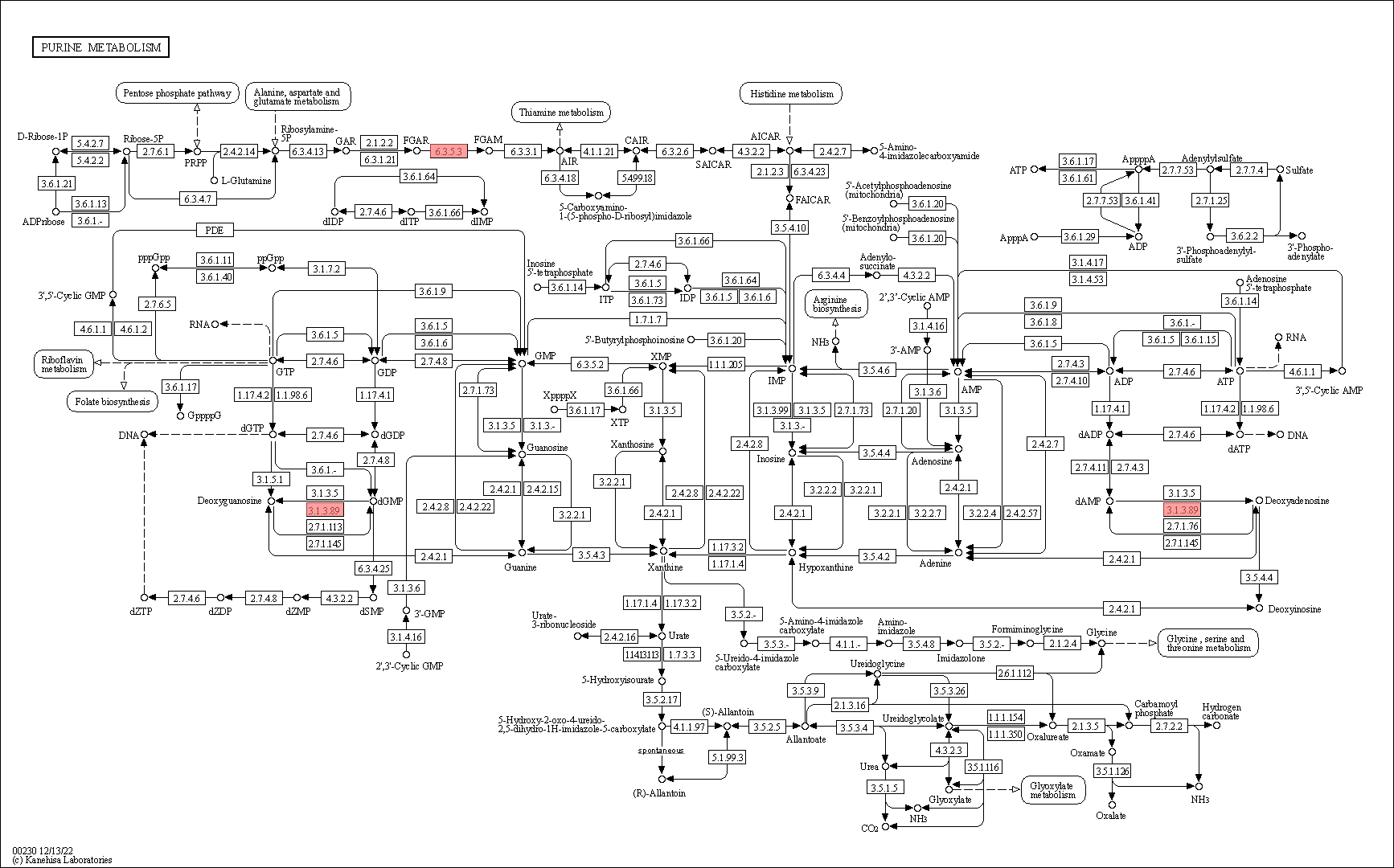

Supplement: Supplemental Information 2 — Supplemental Figures: Figure S1A: Peptide length, peptides per protein, distribution of coverage (%) and MW (kDa) of the LC-MS/MS analysis of rHSA from company A. Figure S2B: Peptide length, peptides per protein, distribution of coverage (%) and MW(kDa) of the LC-MS/MS analysis of rHSA from company B. Figure S3C: Peptide length, peptides per protein, distribution of coverage (%) and MW(kDa) of the LC-MS/MS analysis of pHSA from company C. Figure S4D: Peptide length, peptides per protein, distribution of coverage (%) and MW(kDa) of the LC-MS/MS analysis of pHSA from company D. Figure S5E: Peptide length, peptides per protein, distribution of coverage (%) and MW(kDa) of the LC-MS/MS analysis of pHSA from company E. Figure S6F: Peptide length, peptides per protein, distribution of coverage (%) and MW(kDa) of the LC-MS/MS analysis of pHSA from company F. Figure S7G: Peptide length, peptides per protein, distribution of coverage (%) and MW(kDa) of the LC-MS/MS analysis of pHSA from company G. Figure S8H: Peptide length, peptides per protein, distribution of coverage (%) and MW(kDa) of the LC-MS/MS analysis of pHSA from company H. Figure S9: GO enrichment analysis of the APs in pHSA. Figure S10: Subcellular localization prediction of the APs in pHSA. Figure S11: COG/KOG enrichment analysis of the APs in pHSA. Figure S12: KEGG pathway enrichment analysis of the APs in pHSA. Supplemental Tables: Table S1A: The protein and peptide identified in rHSA from company A. Table S2B: The protein and peptide identified in rHSA from company B. Table S3C: The protein and peptide identified in pHSA from company C. Table S4D: The protein and peptide identified in pHSA from company D. Table S5E: The protein and peptide identified in pHSA from company E. Table S6F: The protein and peptide identified in pHSA from company F. Table S7G: The protein and peptide identified in pHSA from company G. Table S8H: The protein and peptide identified in pHSA from company H. Table S9: The relative abunda [file peerj-13-19624-s002.zip › Supplementary/Supplementary File/Supplementary File1/4-Enrichment_pathway_image/image/map00230 2.png]

Count

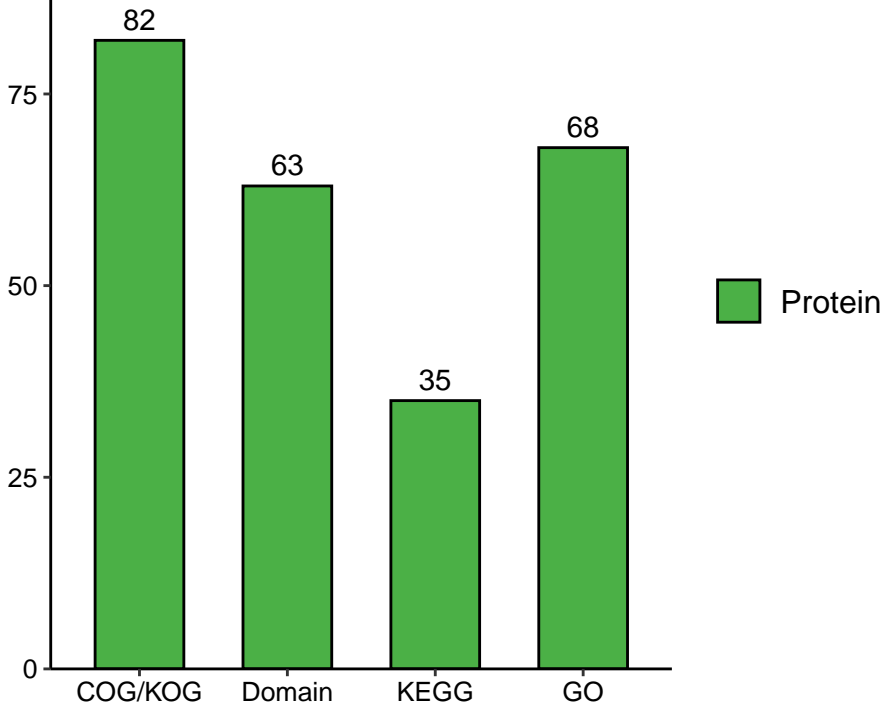

Supplement: Supplemental Information 2 — Supplemental Figures: Figure S1A: Peptide length, peptides per protein, distribution of coverage (%) and MW (kDa) of the LC-MS/MS analysis of rHSA from company A. Figure S2B: Peptide length, peptides per protein, distribution of coverage (%) and MW(kDa) of the LC-MS/MS analysis of rHSA from company B. Figure S3C: Peptide length, peptides per protein, distribution of coverage (%) and MW(kDa) of the LC-MS/MS analysis of pHSA from company C. Figure S4D: Peptide length, peptides per protein, distribution of coverage (%) and MW(kDa) of the LC-MS/MS analysis of pHSA from company D. Figure S5E: Peptide length, peptides per protein, distribution of coverage (%) and MW(kDa) of the LC-MS/MS analysis of pHSA from company E. Figure S6F: Peptide length, peptides per protein, distribution of coverage (%) and MW(kDa) of the LC-MS/MS analysis of pHSA from company F. Figure S7G: Peptide length, peptides per protein, distribution of coverage (%) and MW(kDa) of the LC-MS/MS analysis of pHSA from company G. Figure S8H: Peptide length, peptides per protein, distribution of coverage (%) and MW(kDa) of the LC-MS/MS analysis of pHSA from company H. Figure S9: GO enrichment analysis of the APs in pHSA. Figure S10: Subcellular localization prediction of the APs in pHSA. Figure S11: COG/KOG enrichment analysis of the APs in pHSA. Figure S12: KEGG pathway enrichment analysis of the APs in pHSA. Supplemental Tables: Table S1A: The protein and peptide identified in rHSA from company A. Table S2B: The protein and peptide identified in rHSA from company B. Table S3C: The protein and peptide identified in pHSA from company C. Table S4D: The protein and peptide identified in pHSA from company D. Table S5E: The protein and peptide identified in pHSA from company E. Table S6F: The protein and peptide identified in pHSA from company F. Table S7G: The protein and peptide identified in pHSA from company G. Table S8H: The protein and peptide identified in pHSA from company H. Table S9: The relative abunda [file peerj-13-19624-s002.zip › Supplementary/Supplementary File/Supplementary File2/1-Functional_annotation/barAnnotation_plot.pdf]

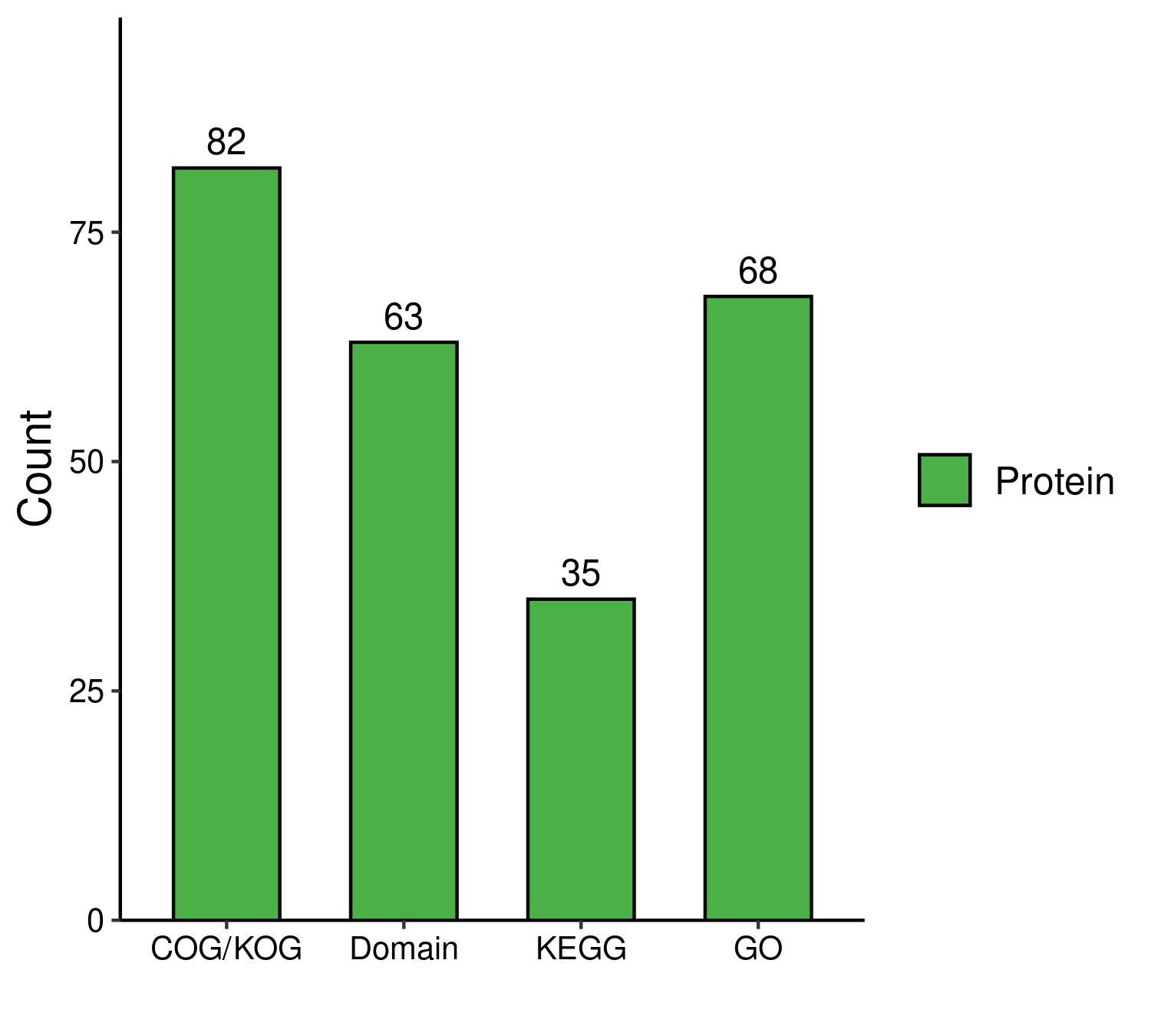

Supplement: Supplemental Information 2 — Supplemental Figures: Figure S1A: Peptide length, peptides per protein, distribution of coverage (%) and MW (kDa) of the LC-MS/MS analysis of rHSA from company A. Figure S2B: Peptide length, peptides per protein, distribution of coverage (%) and MW(kDa) of the LC-MS/MS analysis of rHSA from company B. Figure S3C: Peptide length, peptides per protein, distribution of coverage (%) and MW(kDa) of the LC-MS/MS analysis of pHSA from company C. Figure S4D: Peptide length, peptides per protein, distribution of coverage (%) and MW(kDa) of the LC-MS/MS analysis of pHSA from company D. Figure S5E: Peptide length, peptides per protein, distribution of coverage (%) and MW(kDa) of the LC-MS/MS analysis of pHSA from company E. Figure S6F: Peptide length, peptides per protein, distribution of coverage (%) and MW(kDa) of the LC-MS/MS analysis of pHSA from company F. Figure S7G: Peptide length, peptides per protein, distribution of coverage (%) and MW(kDa) of the LC-MS/MS analysis of pHSA from company G. Figure S8H: Peptide length, peptides per protein, distribution of coverage (%) and MW(kDa) of the LC-MS/MS analysis of pHSA from company H. Figure S9: GO enrichment analysis of the APs in pHSA. Figure S10: Subcellular localization prediction of the APs in pHSA. Figure S11: COG/KOG enrichment analysis of the APs in pHSA. Figure S12: KEGG pathway enrichment analysis of the APs in pHSA. Supplemental Tables: Table S1A: The protein and peptide identified in rHSA from company A. Table S2B: The protein and peptide identified in rHSA from company B. Table S3C: The protein and peptide identified in pHSA from company C. Table S4D: The protein and peptide identified in pHSA from company D. Table S5E: The protein and peptide identified in pHSA from company E. Table S6F: The protein and peptide identified in pHSA from company F. Table S7G: The protein and peptide identified in pHSA from company G. Table S8H: The protein and peptide identified in pHSA from company H. Table S9: The relative abunda [file peerj-13-19624-s002.zip › Supplementary/Supplementary File/Supplementary File2/1-Functional_annotation/barAnnotation_plot.png]

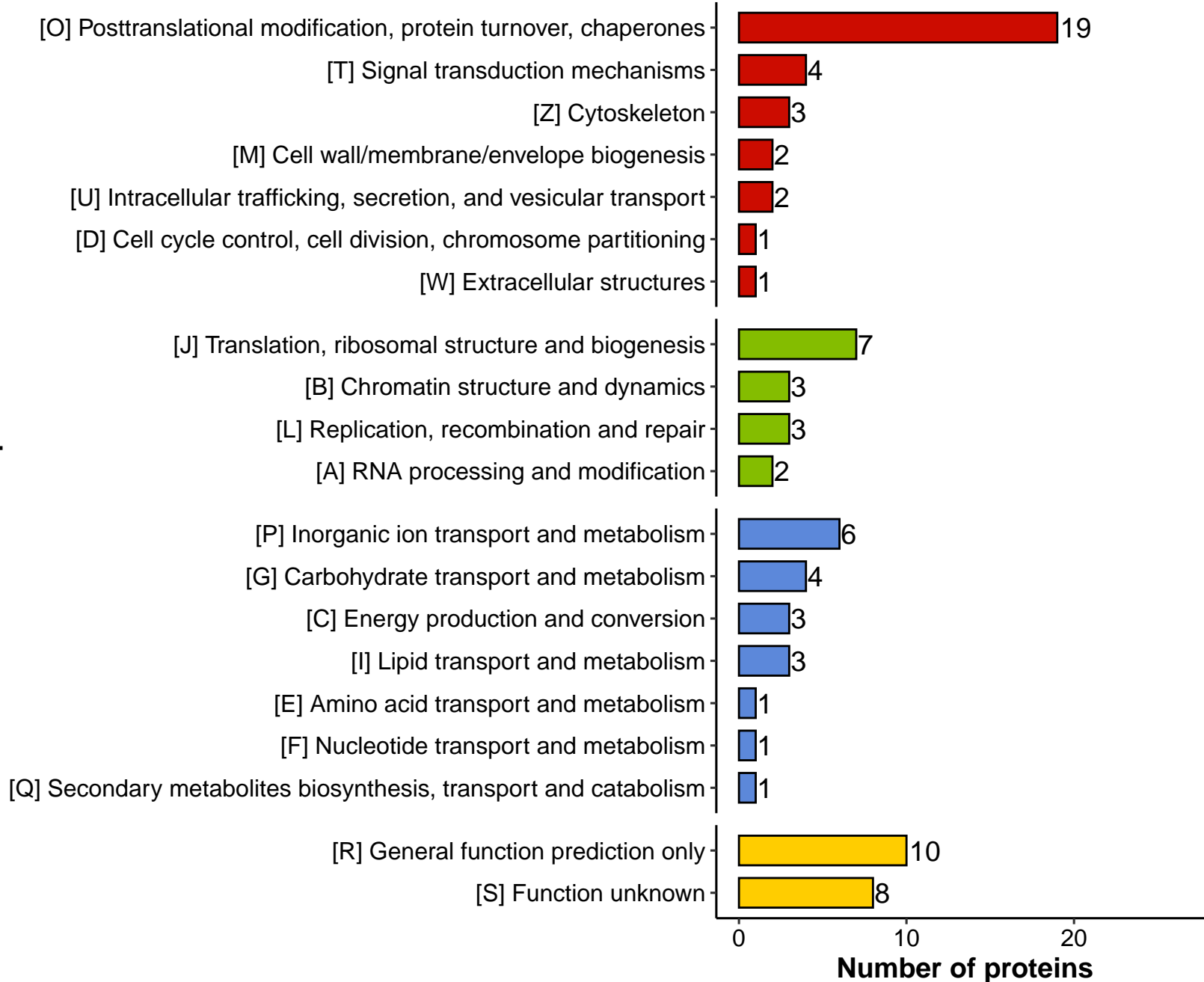**COG/KOG category**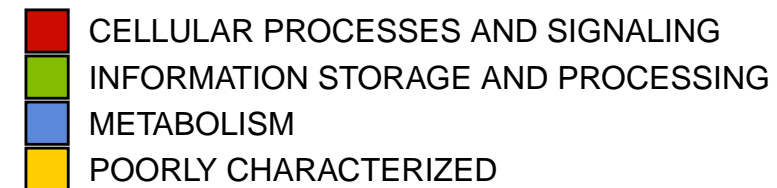

Supplement: Supplemental Information 2 — Supplemental Figures: Figure S1A: Peptide length, peptides per protein, distribution of coverage (%) and MW (kDa) of the LC-MS/MS analysis of rHSA from company A. Figure S2B: Peptide length, peptides per protein, distribution of coverage (%) and MW(kDa) of the LC-MS/MS analysis of rHSA from company B. Figure S3C: Peptide length, peptides per protein, distribution of coverage (%) and MW(kDa) of the LC-MS/MS analysis of pHSA from company C. Figure S4D: Peptide length, peptides per protein, distribution of coverage (%) and MW(kDa) of the LC-MS/MS analysis of pHSA from company D. Figure S5E: Peptide length, peptides per protein, distribution of coverage (%) and MW(kDa) of the LC-MS/MS analysis of pHSA from company E. Figure S6F: Peptide length, peptides per protein, distribution of coverage (%) and MW(kDa) of the LC-MS/MS analysis of pHSA from company F. Figure S7G: Peptide length, peptides per protein, distribution of coverage (%) and MW(kDa) of the LC-MS/MS analysis of pHSA from company G. Figure S8H: Peptide length, peptides per protein, distribution of coverage (%) and MW(kDa) of the LC-MS/MS analysis of pHSA from company H. Figure S9: GO enrichment analysis of the APs in pHSA. Figure S10: Subcellular localization prediction of the APs in pHSA. Figure S11: COG/KOG enrichment analysis of the APs in pHSA. Figure S12: KEGG pathway enrichment analysis of the APs in pHSA. Supplemental Tables: Table S1A: The protein and peptide identified in rHSA from company A. Table S2B: The protein and peptide identified in rHSA from company B. Table S3C: The protein and peptide identified in pHSA from company C. Table S4D: The protein and peptide identified in pHSA from company D. Table S5E: The protein and peptide identified in pHSA from company E. Table S6F: The protein and peptide identified in pHSA from company F. Table S7G: The protein and peptide identified in pHSA from company G. Table S8H: The protein and peptide identified in pHSA from company H. Table S9: The relative abunda [file peerj-13-19624-s002.zip › Supplementary/Supplementary File/Supplementary File2/2-Functional_classification/ident-COG_classify.pdf]

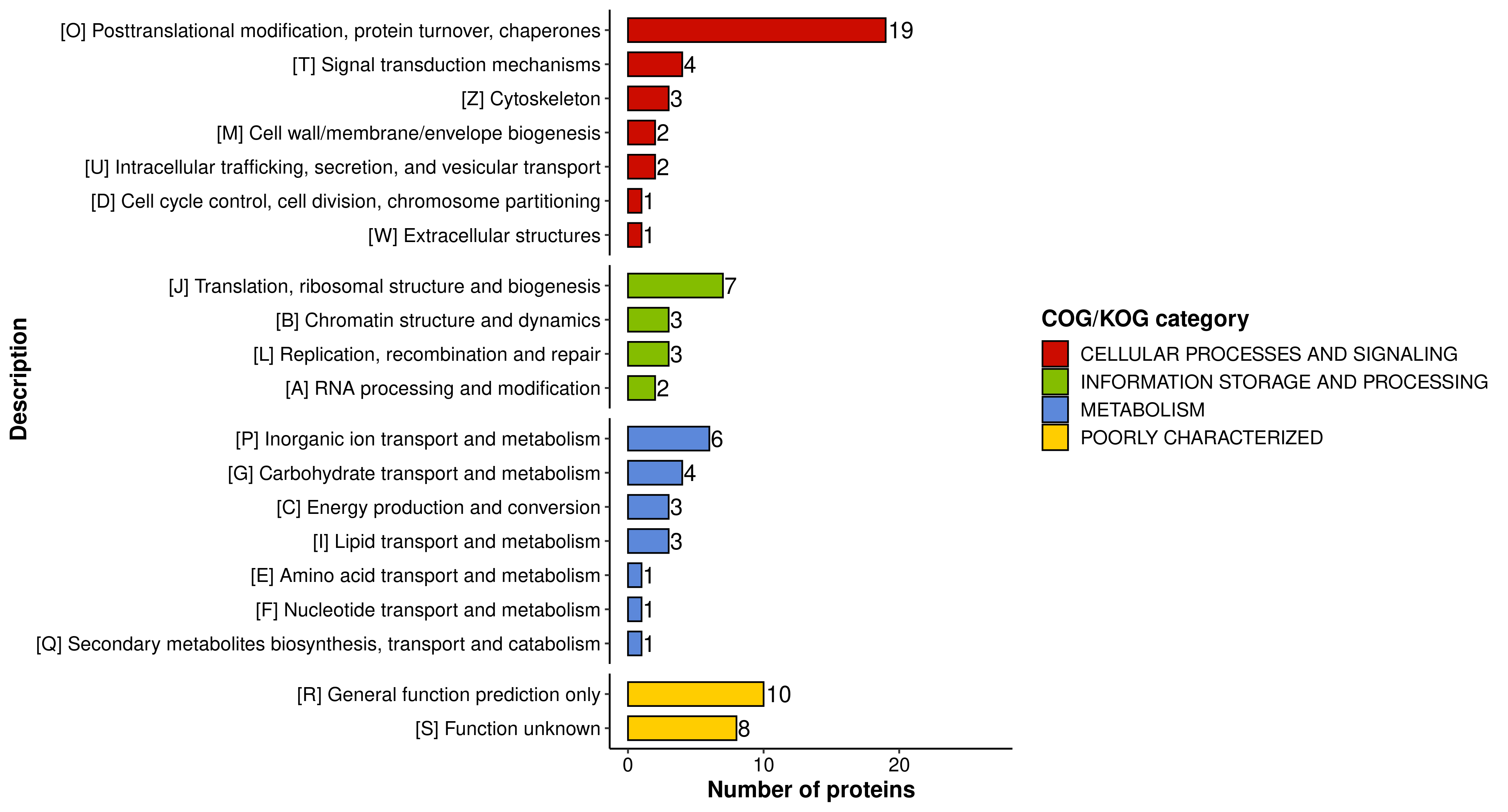

Supplement: Supplemental Information 2 — Supplemental Figures: Figure S1A: Peptide length, peptides per protein, distribution of coverage (%) and MW (kDa) of the LC-MS/MS analysis of rHSA from company A. Figure S2B: Peptide length, peptides per protein, distribution of coverage (%) and MW(kDa) of the LC-MS/MS analysis of rHSA from company B. Figure S3C: Peptide length, peptides per protein, distribution of coverage (%) and MW(kDa) of the LC-MS/MS analysis of pHSA from company C. Figure S4D: Peptide length, peptides per protein, distribution of coverage (%) and MW(kDa) of the LC-MS/MS analysis of pHSA from company D. Figure S5E: Peptide length, peptides per protein, distribution of coverage (%) and MW(kDa) of the LC-MS/MS analysis of pHSA from company E. Figure S6F: Peptide length, peptides per protein, distribution of coverage (%) and MW(kDa) of the LC-MS/MS analysis of pHSA from company F. Figure S7G: Peptide length, peptides per protein, distribution of coverage (%) and MW(kDa) of the LC-MS/MS analysis of pHSA from company G. Figure S8H: Peptide length, peptides per protein, distribution of coverage (%) and MW(kDa) of the LC-MS/MS analysis of pHSA from company H. Figure S9: GO enrichment analysis of the APs in pHSA. Figure S10: Subcellular localization prediction of the APs in pHSA. Figure S11: COG/KOG enrichment analysis of the APs in pHSA. Figure S12: KEGG pathway enrichment analysis of the APs in pHSA. Supplemental Tables: Table S1A: The protein and peptide identified in rHSA from company A. Table S2B: The protein and peptide identified in rHSA from company B. Table S3C: The protein and peptide identified in pHSA from company C. Table S4D: The protein and peptide identified in pHSA from company D. Table S5E: The protein and peptide identified in pHSA from company E. Table S6F: The protein and peptide identified in pHSA from company F. Table S7G: The protein and peptide identified in pHSA from company G. Table S8H: The protein and peptide identified in pHSA from company H. Table S9: The relative abunda [file peerj-13-19624-s002.zip › Supplementary/Supplementary File/Supplementary File2/2-Functional_classification/ident-COG_classify.png]

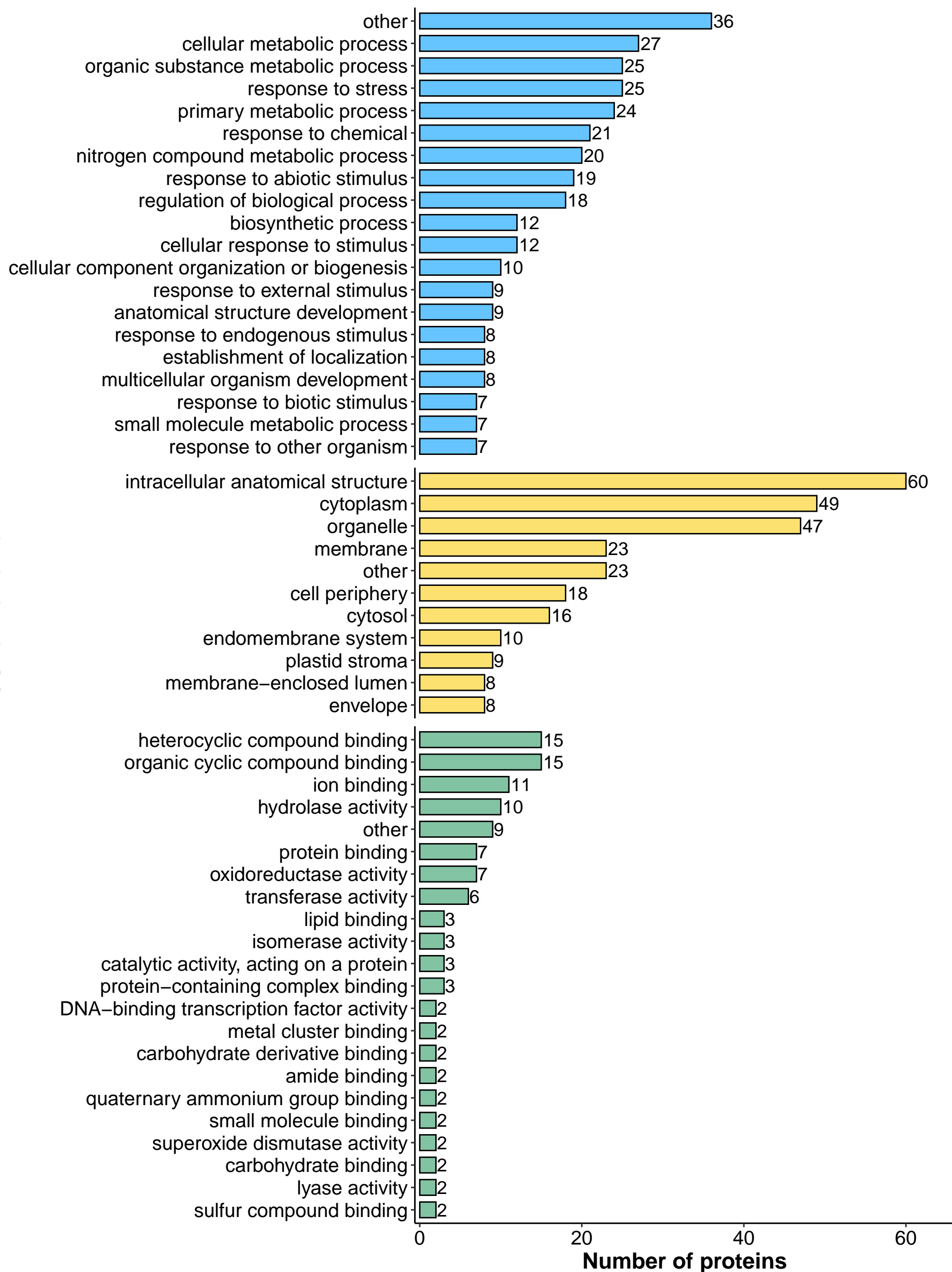

## GO Terms Level 1

- Biological Process
- Cellular Component
- Molecular Function

Supplement: Supplemental Information 2 — Supplemental Figures: Figure S1A: Peptide length, peptides per protein, distribution of coverage (%) and MW (kDa) of the LC-MS/MS analysis of rHSA from company A. Figure S2B: Peptide length, peptides per protein, distribution of coverage (%) and MW(kDa) of the LC-MS/MS analysis of rHSA from company B. Figure S3C: Peptide length, peptides per protein, distribution of coverage (%) and MW(kDa) of the LC-MS/MS analysis of pHSA from company C. Figure S4D: Peptide length, peptides per protein, distribution of coverage (%) and MW(kDa) of the LC-MS/MS analysis of pHSA from company D. Figure S5E: Peptide length, peptides per protein, distribution of coverage (%) and MW(kDa) of the LC-MS/MS analysis of pHSA from company E. Figure S6F: Peptide length, peptides per protein, distribution of coverage (%) and MW(kDa) of the LC-MS/MS analysis of pHSA from company F. Figure S7G: Peptide length, peptides per protein, distribution of coverage (%) and MW(kDa) of the LC-MS/MS analysis of pHSA from company G. Figure S8H: Peptide length, peptides per protein, distribution of coverage (%) and MW(kDa) of the LC-MS/MS analysis of pHSA from company H. Figure S9: GO enrichment analysis of the APs in pHSA. Figure S10: Subcellular localization prediction of the APs in pHSA. Figure S11: COG/KOG enrichment analysis of the APs in pHSA. Figure S12: KEGG pathway enrichment analysis of the APs in pHSA. Supplemental Tables: Table S1A: The protein and peptide identified in rHSA from company A. Table S2B: The protein and peptide identified in rHSA from company B. Table S3C: The protein and peptide identified in pHSA from company C. Table S4D: The protein and peptide identified in pHSA from company D. Table S5E: The protein and peptide identified in pHSA from company E. Table S6F: The protein and peptide identified in pHSA from company F. Table S7G: The protein and peptide identified in pHSA from company G. Table S8H: The protein and peptide identified in pHSA from company H. Table S9: The relative abunda [file peerj-13-19624-s002.zip › Supplementary/Supplementary File/Supplementary File2/2-Functional_classification/ident-GO_classify.pdf]

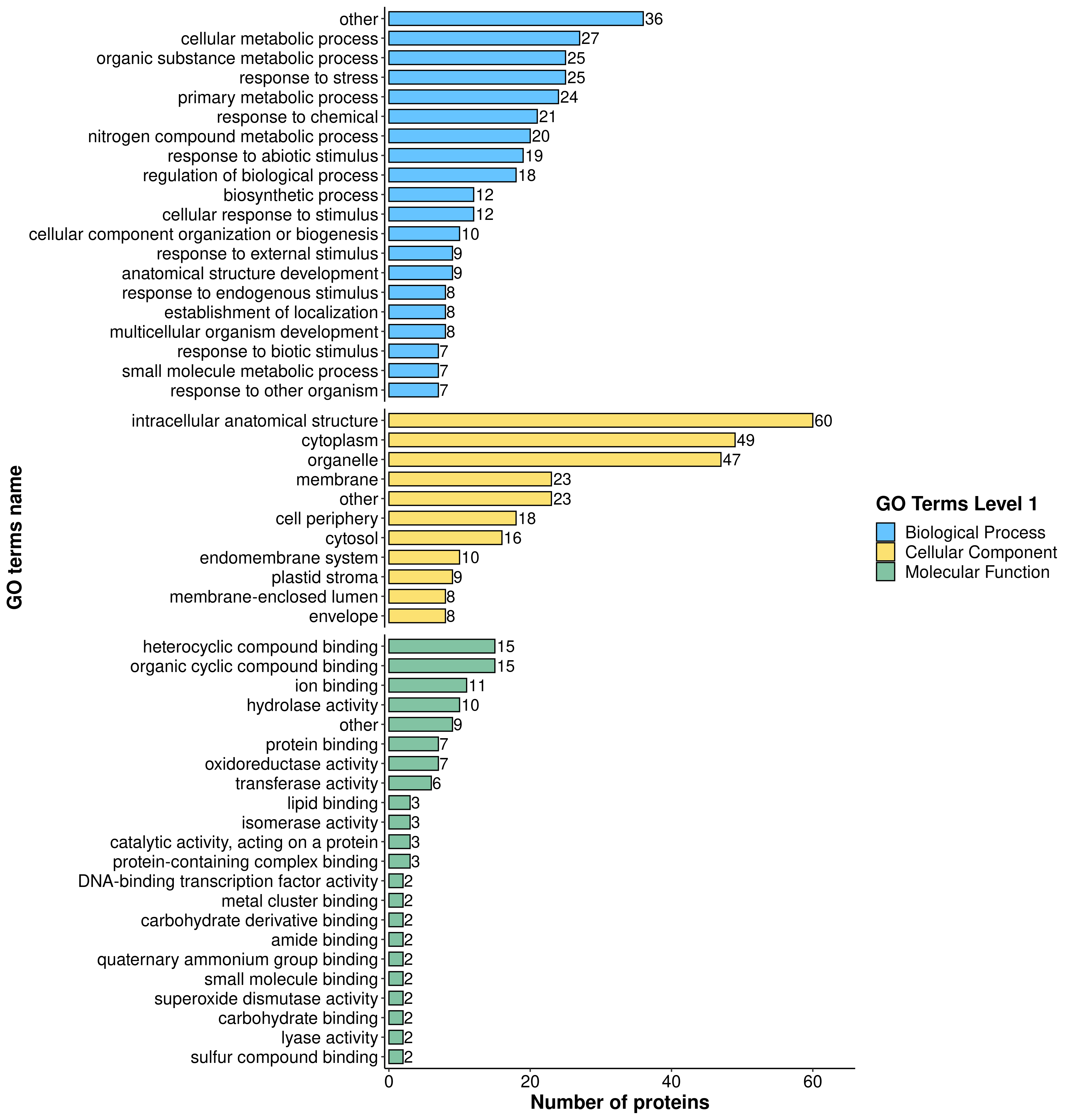

Supplement: Supplemental Information 2 — Supplemental Figures: Figure S1A: Peptide length, peptides per protein, distribution of coverage (%) and MW (kDa) of the LC-MS/MS analysis of rHSA from company A. Figure S2B: Peptide length, peptides per protein, distribution of coverage (%) and MW(kDa) of the LC-MS/MS analysis of rHSA from company B. Figure S3C: Peptide length, peptides per protein, distribution of coverage (%) and MW(kDa) of the LC-MS/MS analysis of pHSA from company C. Figure S4D: Peptide length, peptides per protein, distribution of coverage (%) and MW(kDa) of the LC-MS/MS analysis of pHSA from company D. Figure S5E: Peptide length, peptides per protein, distribution of coverage (%) and MW(kDa) of the LC-MS/MS analysis of pHSA from company E. Figure S6F: Peptide length, peptides per protein, distribution of coverage (%) and MW(kDa) of the LC-MS/MS analysis of pHSA from company F. Figure S7G: Peptide length, peptides per protein, distribution of coverage (%) and MW(kDa) of the LC-MS/MS analysis of pHSA from company G. Figure S8H: Peptide length, peptides per protein, distribution of coverage (%) and MW(kDa) of the LC-MS/MS analysis of pHSA from company H. Figure S9: GO enrichment analysis of the APs in pHSA. Figure S10: Subcellular localization prediction of the APs in pHSA. Figure S11: COG/KOG enrichment analysis of the APs in pHSA. Figure S12: KEGG pathway enrichment analysis of the APs in pHSA. Supplemental Tables: Table S1A: The protein and peptide identified in rHSA from company A. Table S2B: The protein and peptide identified in rHSA from company B. Table S3C: The protein and peptide identified in pHSA from company C. Table S4D: The protein and peptide identified in pHSA from company D. Table S5E: The protein and peptide identified in pHSA from company E. Table S6F: The protein and peptide identified in pHSA from company F. Table S7G: The protein and peptide identified in pHSA from company G. Table S8H: The protein and peptide identified in pHSA from company H. Table S9: The relative abunda [file peerj-13-19624-s002.zip › Supplementary/Supplementary File/Supplementary File2/2-Functional_classification/ident-GO_classify.png]

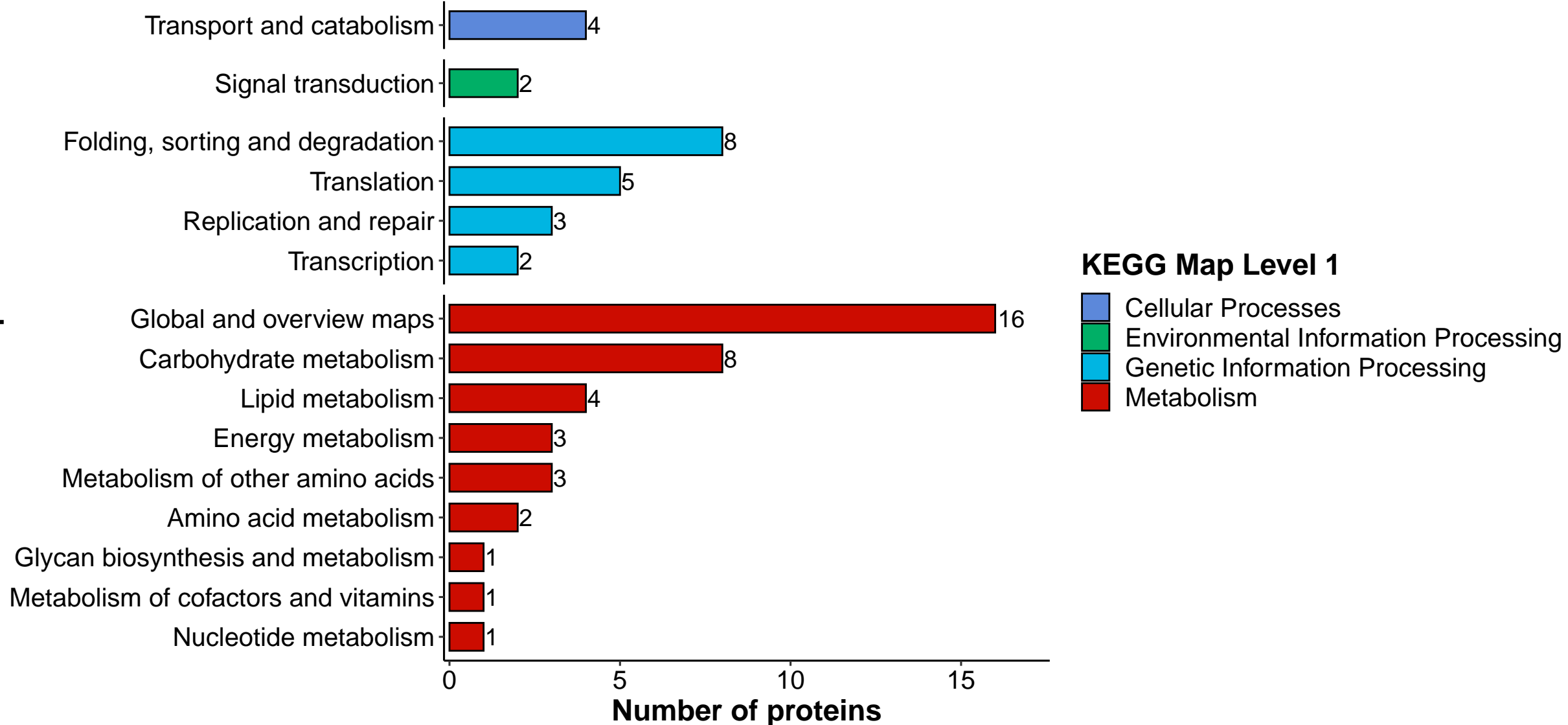

Supplement: Supplemental Information 2 — Supplemental Figures: Figure S1A: Peptide length, peptides per protein, distribution of coverage (%) and MW (kDa) of the LC-MS/MS analysis of rHSA from company A. Figure S2B: Peptide length, peptides per protein, distribution of coverage (%) and MW(kDa) of the LC-MS/MS analysis of rHSA from company B. Figure S3C: Peptide length, peptides per protein, distribution of coverage (%) and MW(kDa) of the LC-MS/MS analysis of pHSA from company C. Figure S4D: Peptide length, peptides per protein, distribution of coverage (%) and MW(kDa) of the LC-MS/MS analysis of pHSA from company D. Figure S5E: Peptide length, peptides per protein, distribution of coverage (%) and MW(kDa) of the LC-MS/MS analysis of pHSA from company E. Figure S6F: Peptide length, peptides per protein, distribution of coverage (%) and MW(kDa) of the LC-MS/MS analysis of pHSA from company F. Figure S7G: Peptide length, peptides per protein, distribution of coverage (%) and MW(kDa) of the LC-MS/MS analysis of pHSA from company G. Figure S8H: Peptide length, peptides per protein, distribution of coverage (%) and MW(kDa) of the LC-MS/MS analysis of pHSA from company H. Figure S9: GO enrichment analysis of the APs in pHSA. Figure S10: Subcellular localization prediction of the APs in pHSA. Figure S11: COG/KOG enrichment analysis of the APs in pHSA. Figure S12: KEGG pathway enrichment analysis of the APs in pHSA. Supplemental Tables: Table S1A: The protein and peptide identified in rHSA from company A. Table S2B: The protein and peptide identified in rHSA from company B. Table S3C: The protein and peptide identified in pHSA from company C. Table S4D: The protein and peptide identified in pHSA from company D. Table S5E: The protein and peptide identified in pHSA from company E. Table S6F: The protein and peptide identified in pHSA from company F. Table S7G: The protein and peptide identified in pHSA from company G. Table S8H: The protein and peptide identified in pHSA from company H. Table S9: The relative abunda [file peerj-13-19624-s002.zip › Supplementary/Supplementary File/Supplementary File2/2-Functional_classification/ident-KEGG_map_classify.pdf]

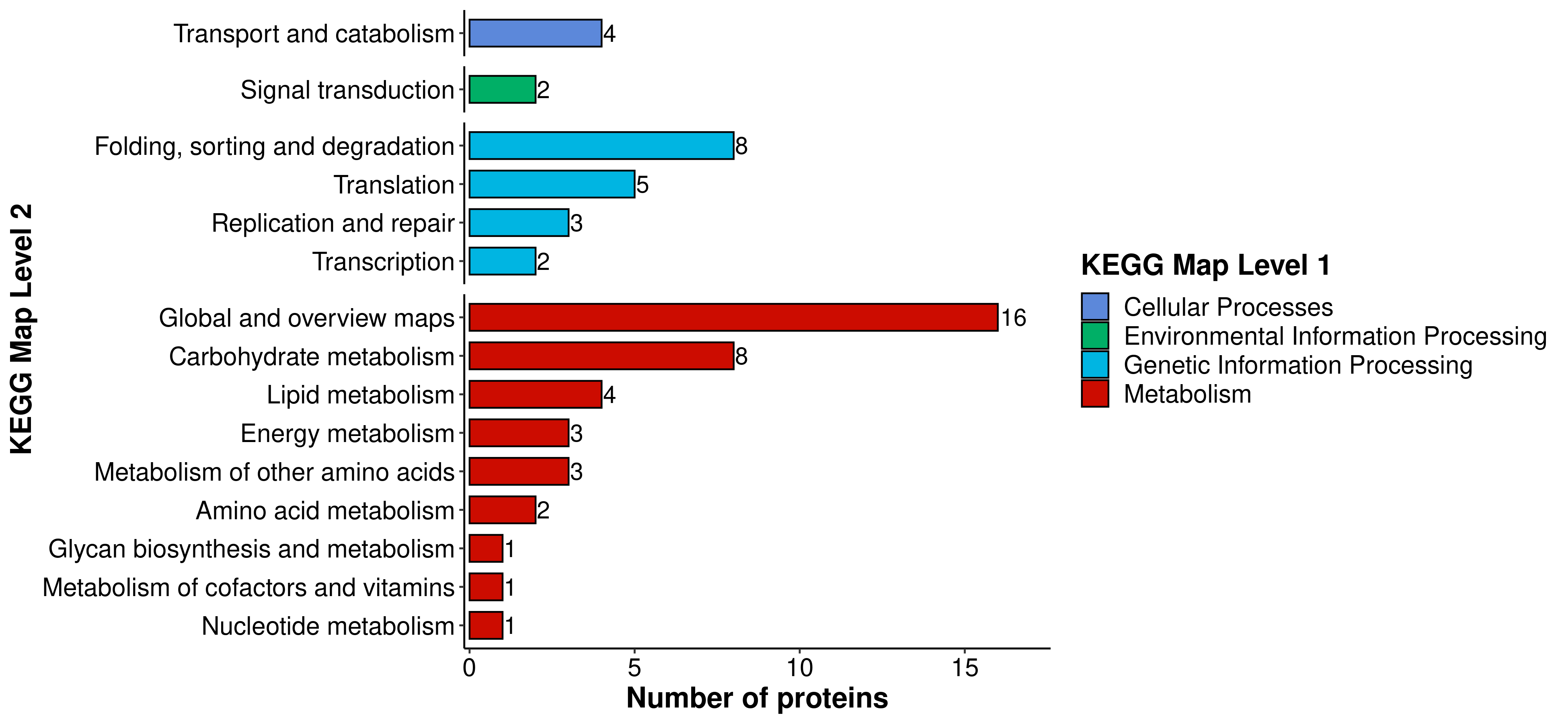

Supplement: Supplemental Information 2 — Supplemental Figures: Figure S1A: Peptide length, peptides per protein, distribution of coverage (%) and MW (kDa) of the LC-MS/MS analysis of rHSA from company A. Figure S2B: Peptide length, peptides per protein, distribution of coverage (%) and MW(kDa) of the LC-MS/MS analysis of rHSA from company B. Figure S3C: Peptide length, peptides per protein, distribution of coverage (%) and MW(kDa) of the LC-MS/MS analysis of pHSA from company C. Figure S4D: Peptide length, peptides per protein, distribution of coverage (%) and MW(kDa) of the LC-MS/MS analysis of pHSA from company D. Figure S5E: Peptide length, peptides per protein, distribution of coverage (%) and MW(kDa) of the LC-MS/MS analysis of pHSA from company E. Figure S6F: Peptide length, peptides per protein, distribution of coverage (%) and MW(kDa) of the LC-MS/MS analysis of pHSA from company F. Figure S7G: Peptide length, peptides per protein, distribution of coverage (%) and MW(kDa) of the LC-MS/MS analysis of pHSA from company G. Figure S8H: Peptide length, peptides per protein, distribution of coverage (%) and MW(kDa) of the LC-MS/MS analysis of pHSA from company H. Figure S9: GO enrichment analysis of the APs in pHSA. Figure S10: Subcellular localization prediction of the APs in pHSA. Figure S11: COG/KOG enrichment analysis of the APs in pHSA. Figure S12: KEGG pathway enrichment analysis of the APs in pHSA. Supplemental Tables: Table S1A: The protein and peptide identified in rHSA from company A. Table S2B: The protein and peptide identified in rHSA from company B. Table S3C: The protein and peptide identified in pHSA from company C. Table S4D: The protein and peptide identified in pHSA from company D. Table S5E: The protein and peptide identified in pHSA from company E. Table S6F: The protein and peptide identified in pHSA from company F. Table S7G: The protein and peptide identified in pHSA from company G. Table S8H: The protein and peptide identified in pHSA from company H. Table S9: The relative abunda [file peerj-13-19624-s002.zip › Supplementary/Supplementary File/Supplementary File2/2-Functional_classification/ident-KEGG_map_classify.png]

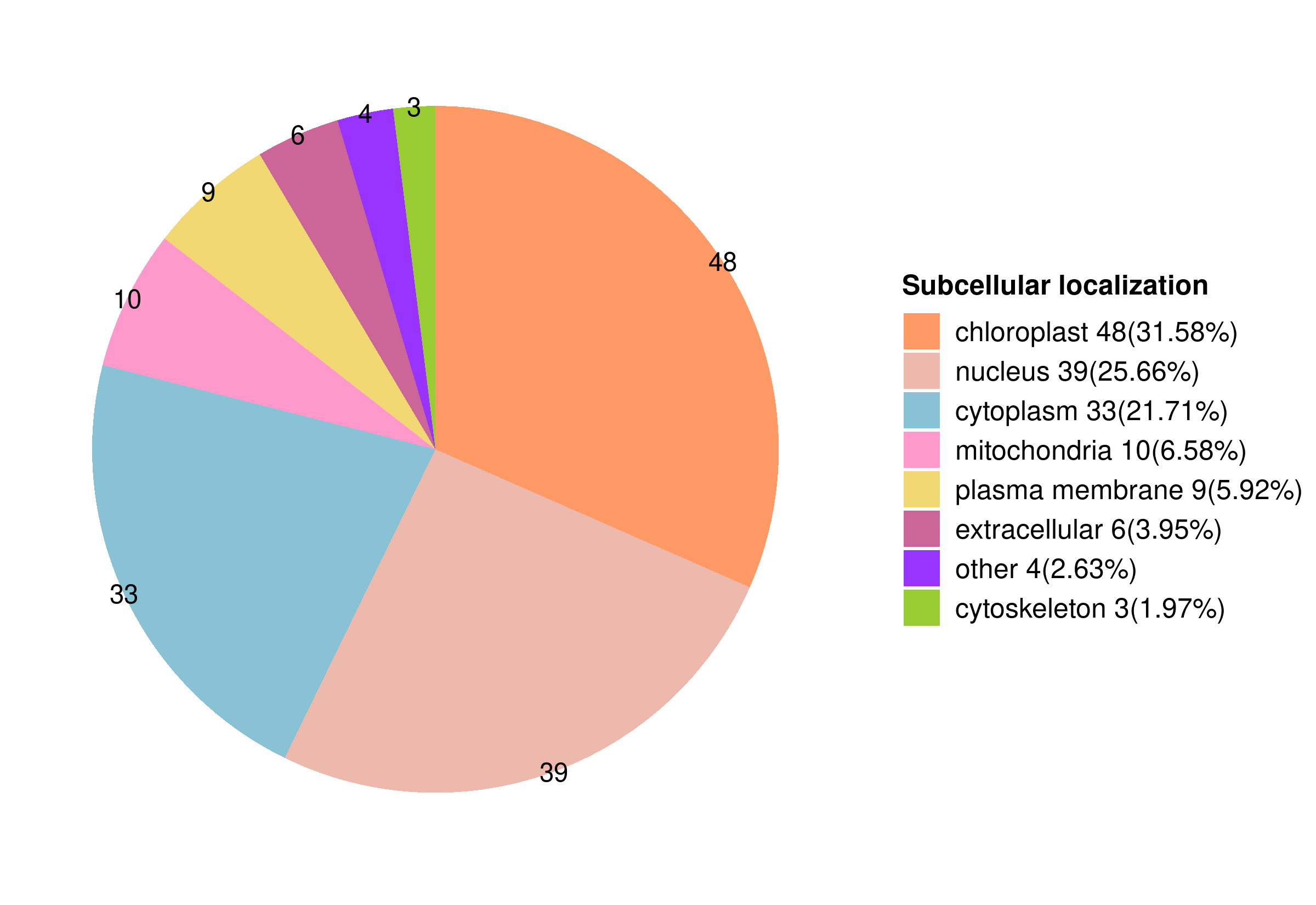

Supplement: Supplemental Information 2 — Supplemental Figures: Figure S1A: Peptide length, peptides per protein, distribution of coverage (%) and MW (kDa) of the LC-MS/MS analysis of rHSA from company A. Figure S2B: Peptide length, peptides per protein, distribution of coverage (%) and MW(kDa) of the LC-MS/MS analysis of rHSA from company B. Figure S3C: Peptide length, peptides per protein, distribution of coverage (%) and MW(kDa) of the LC-MS/MS analysis of pHSA from company C. Figure S4D: Peptide length, peptides per protein, distribution of coverage (%) and MW(kDa) of the LC-MS/MS analysis of pHSA from company D. Figure S5E: Peptide length, peptides per protein, distribution of coverage (%) and MW(kDa) of the LC-MS/MS analysis of pHSA from company E. Figure S6F: Peptide length, peptides per protein, distribution of coverage (%) and MW(kDa) of the LC-MS/MS analysis of pHSA from company F. Figure S7G: Peptide length, peptides per protein, distribution of coverage (%) and MW(kDa) of the LC-MS/MS analysis of pHSA from company G. Figure S8H: Peptide length, peptides per protein, distribution of coverage (%) and MW(kDa) of the LC-MS/MS analysis of pHSA from company H. Figure S9: GO enrichment analysis of the APs in pHSA. Figure S10: Subcellular localization prediction of the APs in pHSA. Figure S11: COG/KOG enrichment analysis of the APs in pHSA. Figure S12: KEGG pathway enrichment analysis of the APs in pHSA. Supplemental Tables: Table S1A: The protein and peptide identified in rHSA from company A. Table S2B: The protein and peptide identified in rHSA from company B. Table S3C: The protein and peptide identified in pHSA from company C. Table S4D: The protein and peptide identified in pHSA from company D. Table S5E: The protein and peptide identified in pHSA from company E. Table S6F: The protein and peptide identified in pHSA from company F. Table S7G: The protein and peptide identified in pHSA from company G. Table S8H: The protein and peptide identified in pHSA from company H. Table S9: The relative abunda [file peerj-13-19624-s002.zip › Supplementary/Supplementary File/Supplementary File2/2-Functional_classification/ident-Subcell_classify.png]

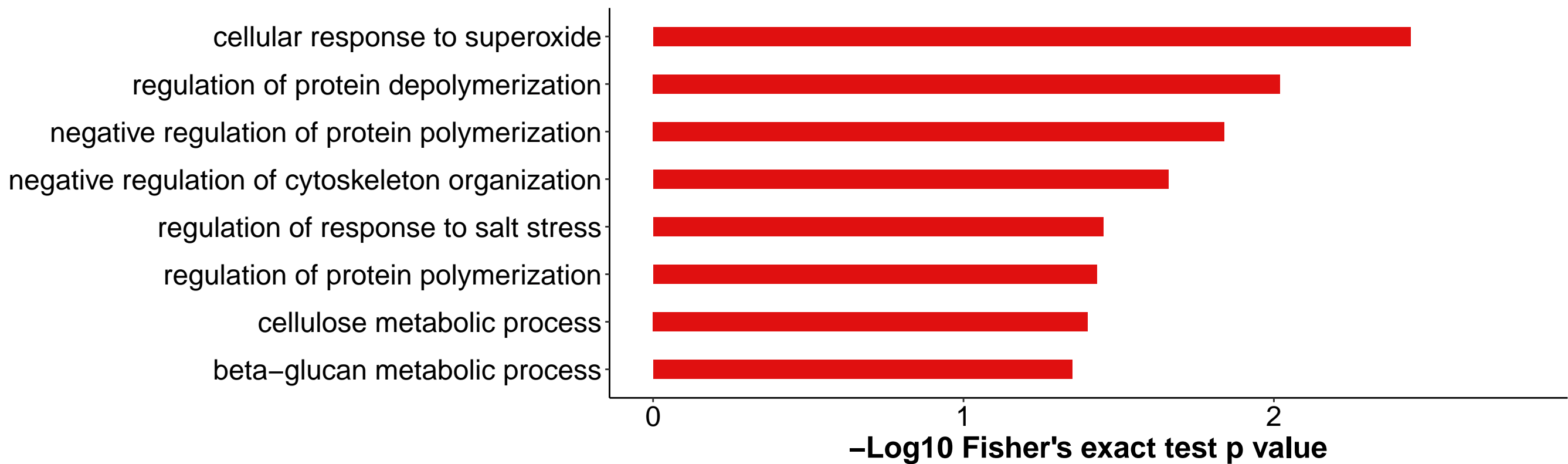

Supplement: Supplemental Information 2 — Supplemental Figures: Figure S1A: Peptide length, peptides per protein, distribution of coverage (%) and MW (kDa) of the LC-MS/MS analysis of rHSA from company A. Figure S2B: Peptide length, peptides per protein, distribution of coverage (%) and MW(kDa) of the LC-MS/MS analysis of rHSA from company B. Figure S3C: Peptide length, peptides per protein, distribution of coverage (%) and MW(kDa) of the LC-MS/MS analysis of pHSA from company C. Figure S4D: Peptide length, peptides per protein, distribution of coverage (%) and MW(kDa) of the LC-MS/MS analysis of pHSA from company D. Figure S5E: Peptide length, peptides per protein, distribution of coverage (%) and MW(kDa) of the LC-MS/MS analysis of pHSA from company E. Figure S6F: Peptide length, peptides per protein, distribution of coverage (%) and MW(kDa) of the LC-MS/MS analysis of pHSA from company F. Figure S7G: Peptide length, peptides per protein, distribution of coverage (%) and MW(kDa) of the LC-MS/MS analysis of pHSA from company G. Figure S8H: Peptide length, peptides per protein, distribution of coverage (%) and MW(kDa) of the LC-MS/MS analysis of pHSA from company H. Figure S9: GO enrichment analysis of the APs in pHSA. Figure S10: Subcellular localization prediction of the APs in pHSA. Figure S11: COG/KOG enrichment analysis of the APs in pHSA. Figure S12: KEGG pathway enrichment analysis of the APs in pHSA. Supplemental Tables: Table S1A: The protein and peptide identified in rHSA from company A. Table S2B: The protein and peptide identified in rHSA from company B. Table S3C: The protein and peptide identified in pHSA from company C. Table S4D: The protein and peptide identified in pHSA from company D. Table S5E: The protein and peptide identified in pHSA from company E. Table S6F: The protein and peptide identified in pHSA from company F. Table S7G: The protein and peptide identified in pHSA from company G. Table S8H: The protein and peptide identified in pHSA from company H. Table S9: The relative abunda [file peerj-13-19624-s002.zip › Supplementary/Supplementary File/Supplementary File2/3-Functional_enrichment/ident-BP_barplot.pdf]

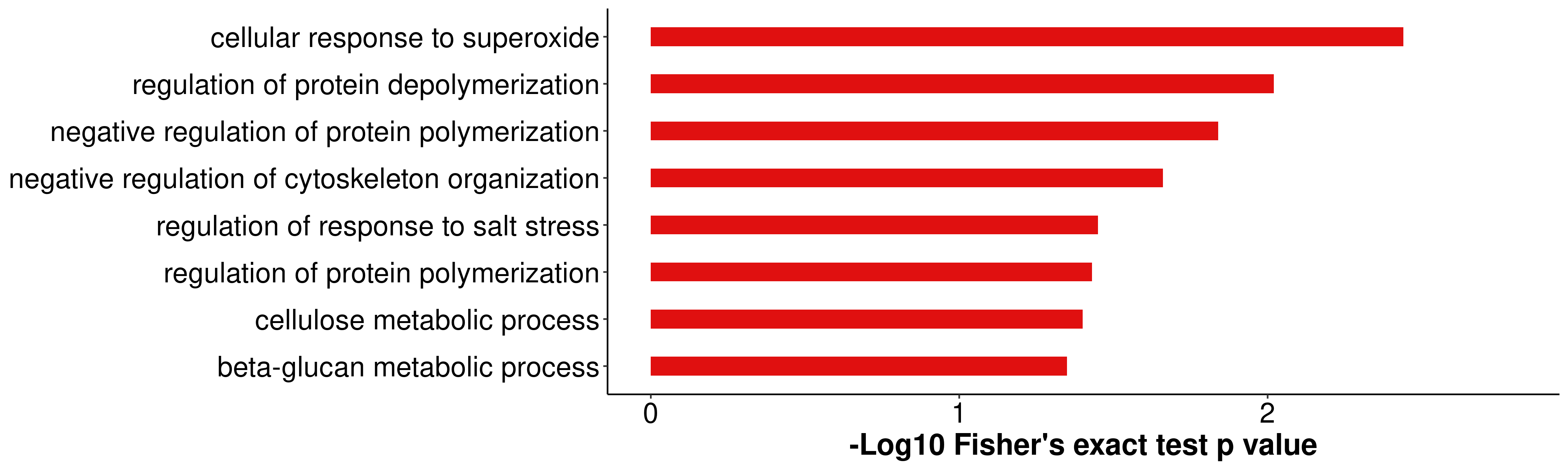

Supplement: Supplemental Information 2 — Supplemental Figures: Figure S1A: Peptide length, peptides per protein, distribution of coverage (%) and MW (kDa) of the LC-MS/MS analysis of rHSA from company A. Figure S2B: Peptide length, peptides per protein, distribution of coverage (%) and MW(kDa) of the LC-MS/MS analysis of rHSA from company B. Figure S3C: Peptide length, peptides per protein, distribution of coverage (%) and MW(kDa) of the LC-MS/MS analysis of pHSA from company C. Figure S4D: Peptide length, peptides per protein, distribution of coverage (%) and MW(kDa) of the LC-MS/MS analysis of pHSA from company D. Figure S5E: Peptide length, peptides per protein, distribution of coverage (%) and MW(kDa) of the LC-MS/MS analysis of pHSA from company E. Figure S6F: Peptide length, peptides per protein, distribution of coverage (%) and MW(kDa) of the LC-MS/MS analysis of pHSA from company F. Figure S7G: Peptide length, peptides per protein, distribution of coverage (%) and MW(kDa) of the LC-MS/MS analysis of pHSA from company G. Figure S8H: Peptide length, peptides per protein, distribution of coverage (%) and MW(kDa) of the LC-MS/MS analysis of pHSA from company H. Figure S9: GO enrichment analysis of the APs in pHSA. Figure S10: Subcellular localization prediction of the APs in pHSA. Figure S11: COG/KOG enrichment analysis of the APs in pHSA. Figure S12: KEGG pathway enrichment analysis of the APs in pHSA. Supplemental Tables: Table S1A: The protein and peptide identified in rHSA from company A. Table S2B: The protein and peptide identified in rHSA from company B. Table S3C: The protein and peptide identified in pHSA from company C. Table S4D: The protein and peptide identified in pHSA from company D. Table S5E: The protein and peptide identified in pHSA from company E. Table S6F: The protein and peptide identified in pHSA from company F. Table S7G: The protein and peptide identified in pHSA from company G. Table S8H: The protein and peptide identified in pHSA from company H. Table S9: The relative abunda [file peerj-13-19624-s002.zip › Supplementary/Supplementary File/Supplementary File2/3-Functional_enrichment/ident-BP_barplot.png]

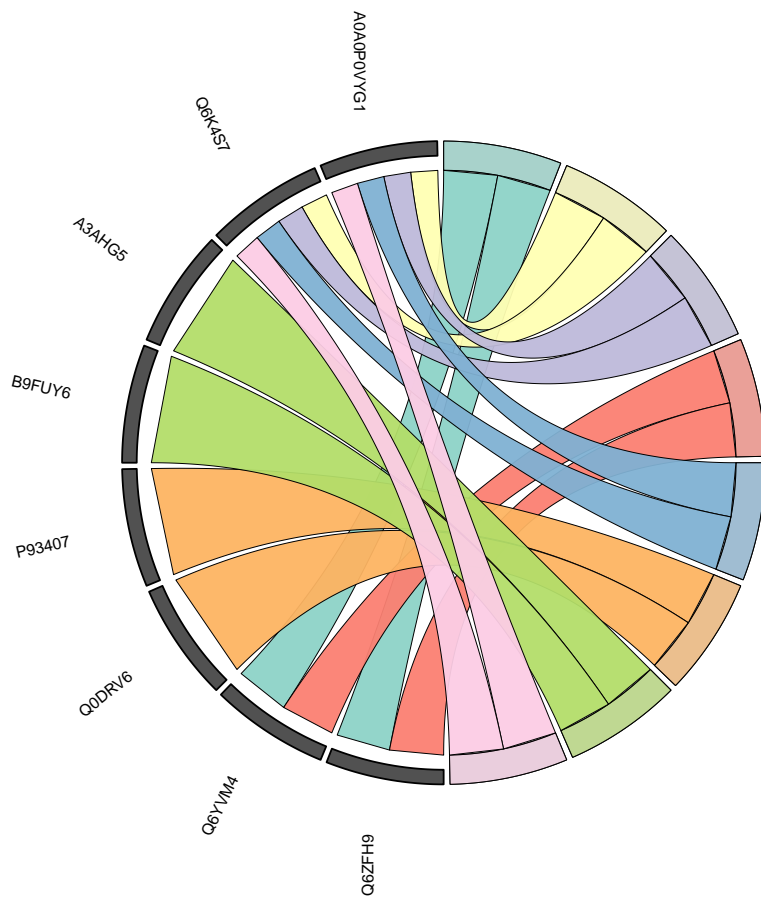

### BP Terms

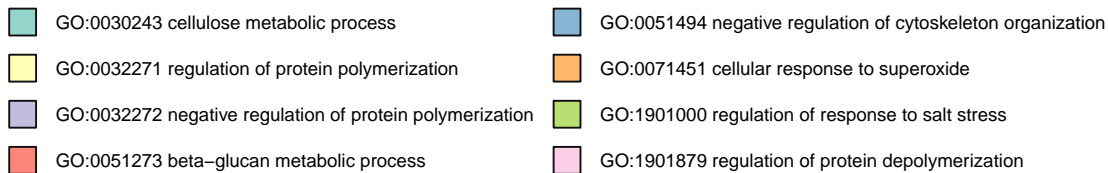

Supplement: Supplemental Information 2 — Supplemental Figures: Figure S1A: Peptide length, peptides per protein, distribution of coverage (%) and MW (kDa) of the LC-MS/MS analysis of rHSA from company A. Figure S2B: Peptide length, peptides per protein, distribution of coverage (%) and MW(kDa) of the LC-MS/MS analysis of rHSA from company B. Figure S3C: Peptide length, peptides per protein, distribution of coverage (%) and MW(kDa) of the LC-MS/MS analysis of pHSA from company C. Figure S4D: Peptide length, peptides per protein, distribution of coverage (%) and MW(kDa) of the LC-MS/MS analysis of pHSA from company D. Figure S5E: Peptide length, peptides per protein, distribution of coverage (%) and MW(kDa) of the LC-MS/MS analysis of pHSA from company E. Figure S6F: Peptide length, peptides per protein, distribution of coverage (%) and MW(kDa) of the LC-MS/MS analysis of pHSA from company F. Figure S7G: Peptide length, peptides per protein, distribution of coverage (%) and MW(kDa) of the LC-MS/MS analysis of pHSA from company G. Figure S8H: Peptide length, peptides per protein, distribution of coverage (%) and MW(kDa) of the LC-MS/MS analysis of pHSA from company H. Figure S9: GO enrichment analysis of the APs in pHSA. Figure S10: Subcellular localization prediction of the APs in pHSA. Figure S11: COG/KOG enrichment analysis of the APs in pHSA. Figure S12: KEGG pathway enrichment analysis of the APs in pHSA. Supplemental Tables: Table S1A: The protein and peptide identified in rHSA from company A. Table S2B: The protein and peptide identified in rHSA from company B. Table S3C: The protein and peptide identified in pHSA from company C. Table S4D: The protein and peptide identified in pHSA from company D. Table S5E: The protein and peptide identified in pHSA from company E. Table S6F: The protein and peptide identified in pHSA from company F. Table S7G: The protein and peptide identified in pHSA from company G. Table S8H: The protein and peptide identified in pHSA from company H. Table S9: The relative abunda [file peerj-13-19624-s002.zip › Supplementary/Supplementary File/Supplementary File2/3-Functional_enrichment/ident-BP_cirplot.pdf]

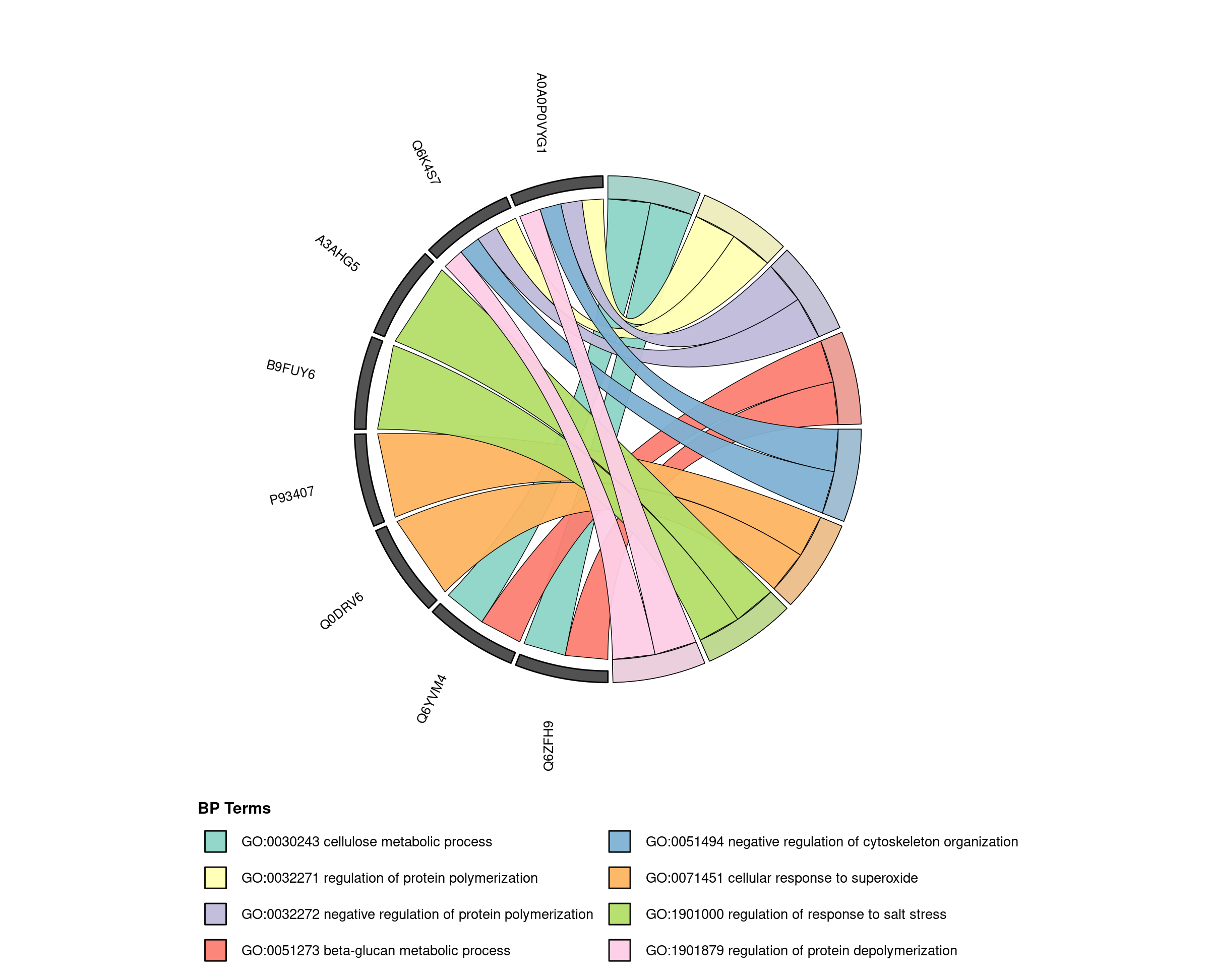

Supplement: Supplemental Information 2 — Supplemental Figures: Figure S1A: Peptide length, peptides per protein, distribution of coverage (%) and MW (kDa) of the LC-MS/MS analysis of rHSA from company A. Figure S2B: Peptide length, peptides per protein, distribution of coverage (%) and MW(kDa) of the LC-MS/MS analysis of rHSA from company B. Figure S3C: Peptide length, peptides per protein, distribution of coverage (%) and MW(kDa) of the LC-MS/MS analysis of pHSA from company C. Figure S4D: Peptide length, peptides per protein, distribution of coverage (%) and MW(kDa) of the LC-MS/MS analysis of pHSA from company D. Figure S5E: Peptide length, peptides per protein, distribution of coverage (%) and MW(kDa) of the LC-MS/MS analysis of pHSA from company E. Figure S6F: Peptide length, peptides per protein, distribution of coverage (%) and MW(kDa) of the LC-MS/MS analysis of pHSA from company F. Figure S7G: Peptide length, peptides per protein, distribution of coverage (%) and MW(kDa) of the LC-MS/MS analysis of pHSA from company G. Figure S8H: Peptide length, peptides per protein, distribution of coverage (%) and MW(kDa) of the LC-MS/MS analysis of pHSA from company H. Figure S9: GO enrichment analysis of the APs in pHSA. Figure S10: Subcellular localization prediction of the APs in pHSA. Figure S11: COG/KOG enrichment analysis of the APs in pHSA. Figure S12: KEGG pathway enrichment analysis of the APs in pHSA. Supplemental Tables: Table S1A: The protein and peptide identified in rHSA from company A. Table S2B: The protein and peptide identified in rHSA from company B. Table S3C: The protein and peptide identified in pHSA from company C. Table S4D: The protein and peptide identified in pHSA from company D. Table S5E: The protein and peptide identified in pHSA from company E. Table S6F: The protein and peptide identified in pHSA from company F. Table S7G: The protein and peptide identified in pHSA from company G. Table S8H: The protein and peptide identified in pHSA from company H. Table S9: The relative abunda [file peerj-13-19624-s002.zip › Supplementary/Supplementary File/Supplementary File2/3-Functional_enrichment/ident-BP_cirplot.png]

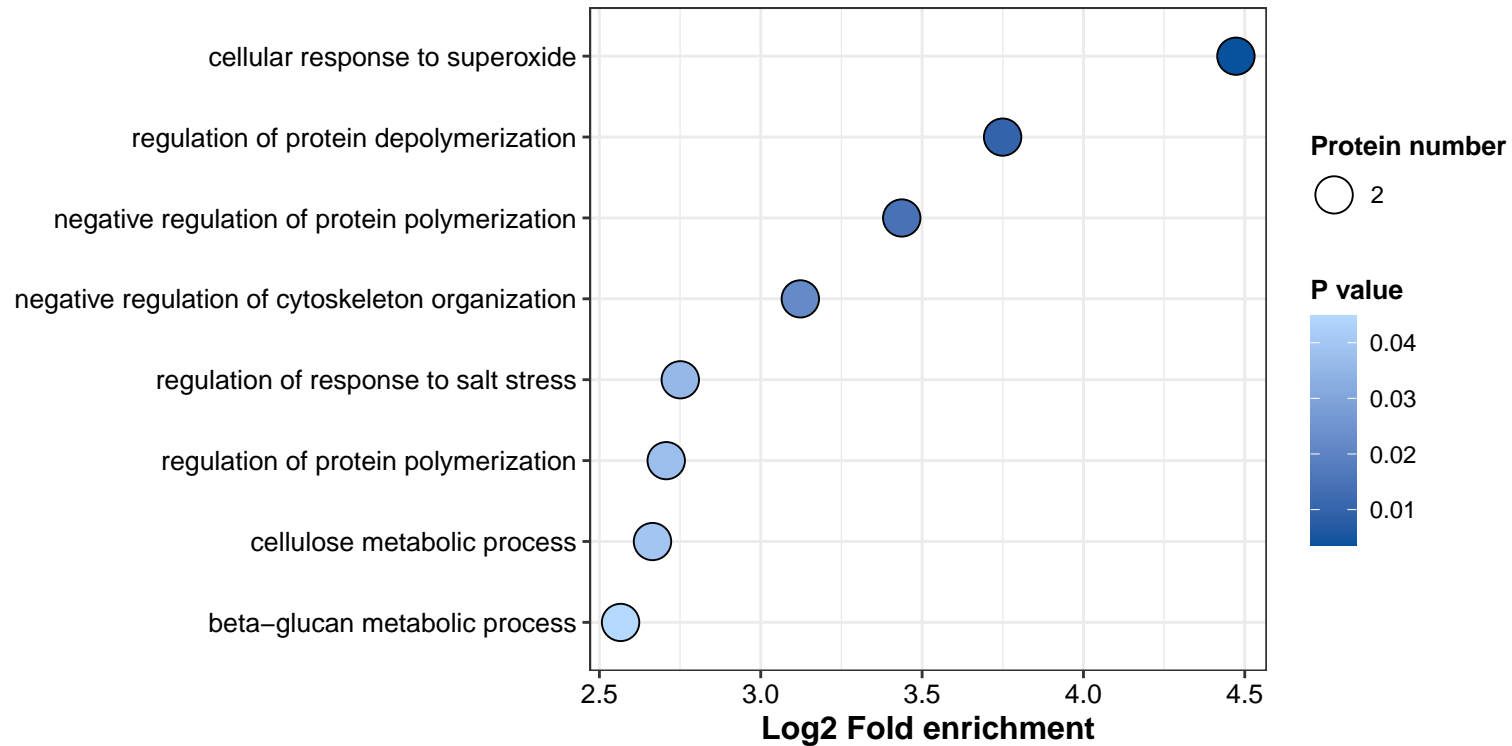

Supplement: Supplemental Information 2 — Supplemental Figures: Figure S1A: Peptide length, peptides per protein, distribution of coverage (%) and MW (kDa) of the LC-MS/MS analysis of rHSA from company A. Figure S2B: Peptide length, peptides per protein, distribution of coverage (%) and MW(kDa) of the LC-MS/MS analysis of rHSA from company B. Figure S3C: Peptide length, peptides per protein, distribution of coverage (%) and MW(kDa) of the LC-MS/MS analysis of pHSA from company C. Figure S4D: Peptide length, peptides per protein, distribution of coverage (%) and MW(kDa) of the LC-MS/MS analysis of pHSA from company D. Figure S5E: Peptide length, peptides per protein, distribution of coverage (%) and MW(kDa) of the LC-MS/MS analysis of pHSA from company E. Figure S6F: Peptide length, peptides per protein, distribution of coverage (%) and MW(kDa) of the LC-MS/MS analysis of pHSA from company F. Figure S7G: Peptide length, peptides per protein, distribution of coverage (%) and MW(kDa) of the LC-MS/MS analysis of pHSA from company G. Figure S8H: Peptide length, peptides per protein, distribution of coverage (%) and MW(kDa) of the LC-MS/MS analysis of pHSA from company H. Figure S9: GO enrichment analysis of the APs in pHSA. Figure S10: Subcellular localization prediction of the APs in pHSA. Figure S11: COG/KOG enrichment analysis of the APs in pHSA. Figure S12: KEGG pathway enrichment analysis of the APs in pHSA. Supplemental Tables: Table S1A: The protein and peptide identified in rHSA from company A. Table S2B: The protein and peptide identified in rHSA from company B. Table S3C: The protein and peptide identified in pHSA from company C. Table S4D: The protein and peptide identified in pHSA from company D. Table S5E: The protein and peptide identified in pHSA from company E. Table S6F: The protein and peptide identified in pHSA from company F. Table S7G: The protein and peptide identified in pHSA from company G. Table S8H: The protein and peptide identified in pHSA from company H. Table S9: The relative abunda [file peerj-13-19624-s002.zip › Supplementary/Supplementary File/Supplementary File2/3-Functional_enrichment/ident-BP_dotplot.pdf]

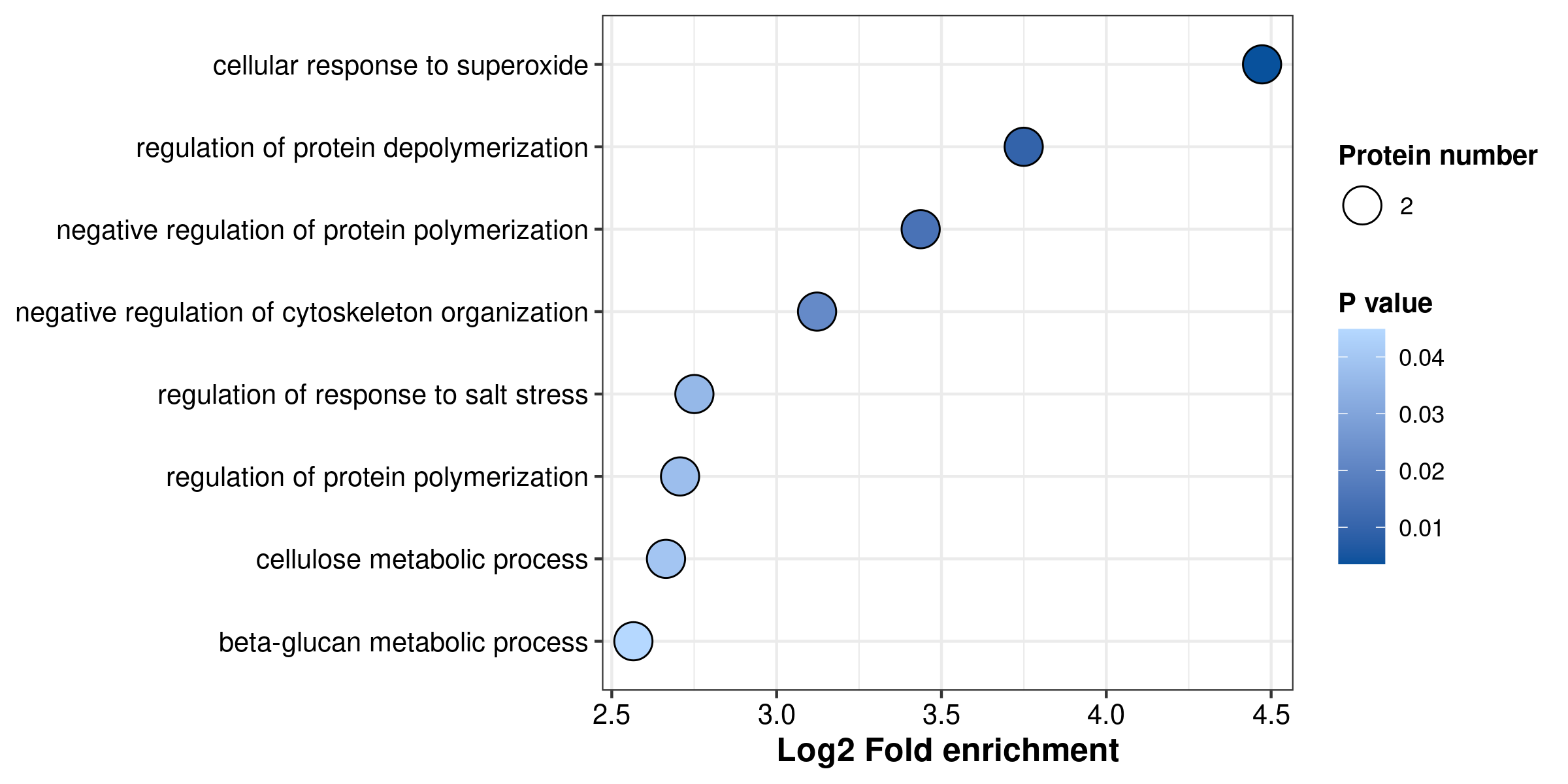

Supplement: Supplemental Information 2 — Supplemental Figures: Figure S1A: Peptide length, peptides per protein, distribution of coverage (%) and MW (kDa) of the LC-MS/MS analysis of rHSA from company A. Figure S2B: Peptide length, peptides per protein, distribution of coverage (%) and MW(kDa) of the LC-MS/MS analysis of rHSA from company B. Figure S3C: Peptide length, peptides per protein, distribution of coverage (%) and MW(kDa) of the LC-MS/MS analysis of pHSA from company C. Figure S4D: Peptide length, peptides per protein, distribution of coverage (%) and MW(kDa) of the LC-MS/MS analysis of pHSA from company D. Figure S5E: Peptide length, peptides per protein, distribution of coverage (%) and MW(kDa) of the LC-MS/MS analysis of pHSA from company E. Figure S6F: Peptide length, peptides per protein, distribution of coverage (%) and MW(kDa) of the LC-MS/MS analysis of pHSA from company F. Figure S7G: Peptide length, peptides per protein, distribution of coverage (%) and MW(kDa) of the LC-MS/MS analysis of pHSA from company G. Figure S8H: Peptide length, peptides per protein, distribution of coverage (%) and MW(kDa) of the LC-MS/MS analysis of pHSA from company H. Figure S9: GO enrichment analysis of the APs in pHSA. Figure S10: Subcellular localization prediction of the APs in pHSA. Figure S11: COG/KOG enrichment analysis of the APs in pHSA. Figure S12: KEGG pathway enrichment analysis of the APs in pHSA. Supplemental Tables: Table S1A: The protein and peptide identified in rHSA from company A. Table S2B: The protein and peptide identified in rHSA from company B. Table S3C: The protein and peptide identified in pHSA from company C. Table S4D: The protein and peptide identified in pHSA from company D. Table S5E: The protein and peptide identified in pHSA from company E. Table S6F: The protein and peptide identified in pHSA from company F. Table S7G: The protein and peptide identified in pHSA from company G. Table S8H: The protein and peptide identified in pHSA from company H. Table S9: The relative abunda [file peerj-13-19624-s002.zip › Supplementary/Supplementary File/Supplementary File2/3-Functional_enrichment/ident-BP_dotplot.png]

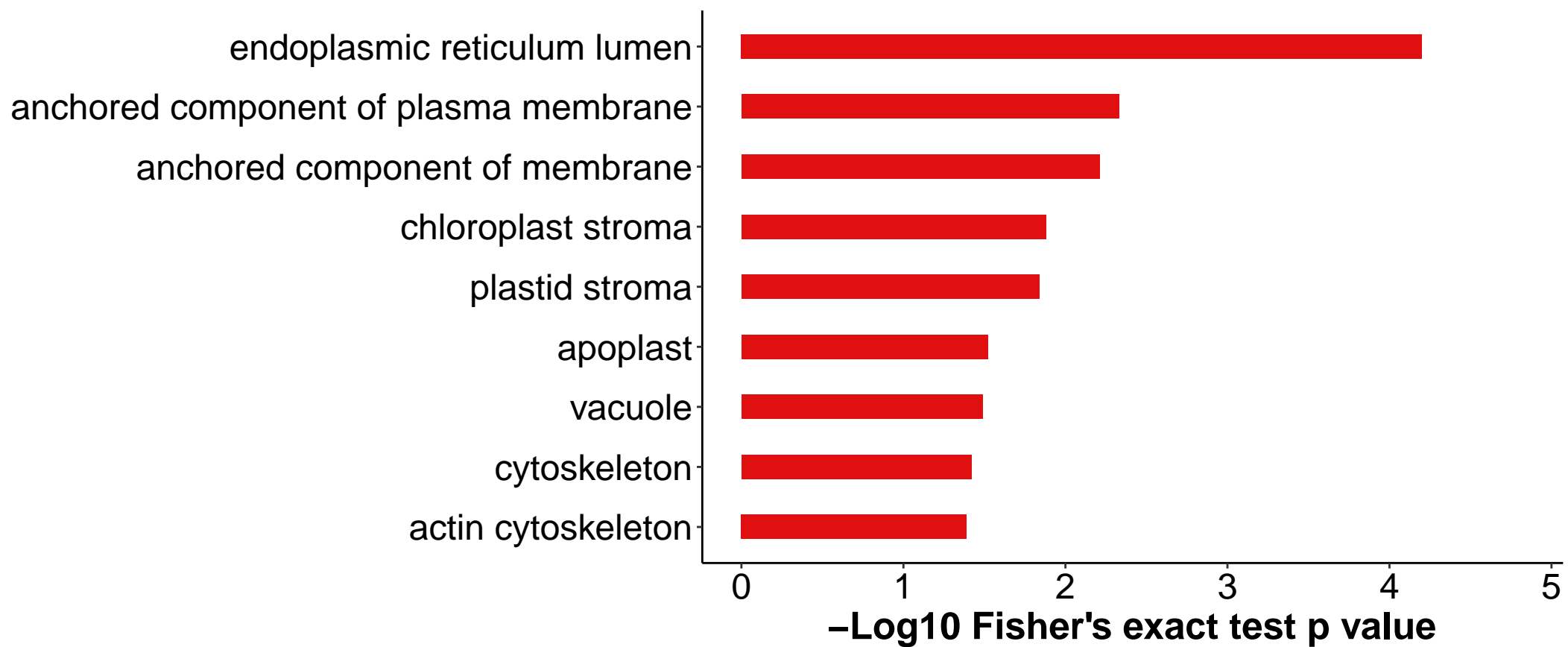

Supplement: Supplemental Information 2 — Supplemental Figures: Figure S1A: Peptide length, peptides per protein, distribution of coverage (%) and MW (kDa) of the LC-MS/MS analysis of rHSA from company A. Figure S2B: Peptide length, peptides per protein, distribution of coverage (%) and MW(kDa) of the LC-MS/MS analysis of rHSA from company B. Figure S3C: Peptide length, peptides per protein, distribution of coverage (%) and MW(kDa) of the LC-MS/MS analysis of pHSA from company C. Figure S4D: Peptide length, peptides per protein, distribution of coverage (%) and MW(kDa) of the LC-MS/MS analysis of pHSA from company D. Figure S5E: Peptide length, peptides per protein, distribution of coverage (%) and MW(kDa) of the LC-MS/MS analysis of pHSA from company E. Figure S6F: Peptide length, peptides per protein, distribution of coverage (%) and MW(kDa) of the LC-MS/MS analysis of pHSA from company F. Figure S7G: Peptide length, peptides per protein, distribution of coverage (%) and MW(kDa) of the LC-MS/MS analysis of pHSA from company G. Figure S8H: Peptide length, peptides per protein, distribution of coverage (%) and MW(kDa) of the LC-MS/MS analysis of pHSA from company H. Figure S9: GO enrichment analysis of the APs in pHSA. Figure S10: Subcellular localization prediction of the APs in pHSA. Figure S11: COG/KOG enrichment analysis of the APs in pHSA. Figure S12: KEGG pathway enrichment analysis of the APs in pHSA. Supplemental Tables: Table S1A: The protein and peptide identified in rHSA from company A. Table S2B: The protein and peptide identified in rHSA from company B. Table S3C: The protein and peptide identified in pHSA from company C. Table S4D: The protein and peptide identified in pHSA from company D. Table S5E: The protein and peptide identified in pHSA from company E. Table S6F: The protein and peptide identified in pHSA from company F. Table S7G: The protein and peptide identified in pHSA from company G. Table S8H: The protein and peptide identified in pHSA from company H. Table S9: The relative abunda [file peerj-13-19624-s002.zip › Supplementary/Supplementary File/Supplementary File2/3-Functional_enrichment/ident-CC_barplot.pdf]

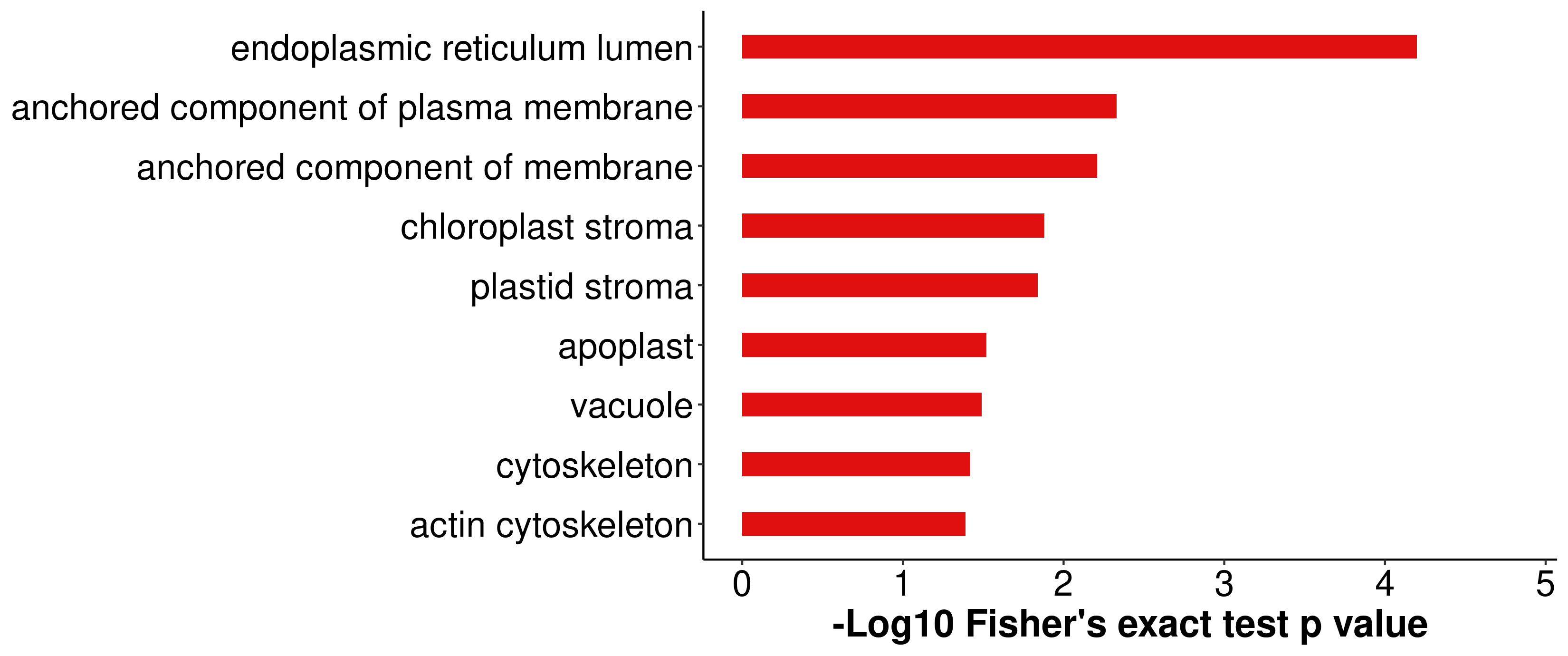

Supplement: Supplemental Information 2 — Supplemental Figures: Figure S1A: Peptide length, peptides per protein, distribution of coverage (%) and MW (kDa) of the LC-MS/MS analysis of rHSA from company A. Figure S2B: Peptide length, peptides per protein, distribution of coverage (%) and MW(kDa) of the LC-MS/MS analysis of rHSA from company B. Figure S3C: Peptide length, peptides per protein, distribution of coverage (%) and MW(kDa) of the LC-MS/MS analysis of pHSA from company C. Figure S4D: Peptide length, peptides per protein, distribution of coverage (%) and MW(kDa) of the LC-MS/MS analysis of pHSA from company D. Figure S5E: Peptide length, peptides per protein, distribution of coverage (%) and MW(kDa) of the LC-MS/MS analysis of pHSA from company E. Figure S6F: Peptide length, peptides per protein, distribution of coverage (%) and MW(kDa) of the LC-MS/MS analysis of pHSA from company F. Figure S7G: Peptide length, peptides per protein, distribution of coverage (%) and MW(kDa) of the LC-MS/MS analysis of pHSA from company G. Figure S8H: Peptide length, peptides per protein, distribution of coverage (%) and MW(kDa) of the LC-MS/MS analysis of pHSA from company H. Figure S9: GO enrichment analysis of the APs in pHSA. Figure S10: Subcellular localization prediction of the APs in pHSA. Figure S11: COG/KOG enrichment analysis of the APs in pHSA. Figure S12: KEGG pathway enrichment analysis of the APs in pHSA. Supplemental Tables: Table S1A: The protein and peptide identified in rHSA from company A. Table S2B: The protein and peptide identified in rHSA from company B. Table S3C: The protein and peptide identified in pHSA from company C. Table S4D: The protein and peptide identified in pHSA from company D. Table S5E: The protein and peptide identified in pHSA from company E. Table S6F: The protein and peptide identified in pHSA from company F. Table S7G: The protein and peptide identified in pHSA from company G. Table S8H: The protein and peptide identified in pHSA from company H. Table S9: The relative abunda [file peerj-13-19624-s002.zip › Supplementary/Supplementary File/Supplementary File2/3-Functional_enrichment/ident-CC_barplot.png]

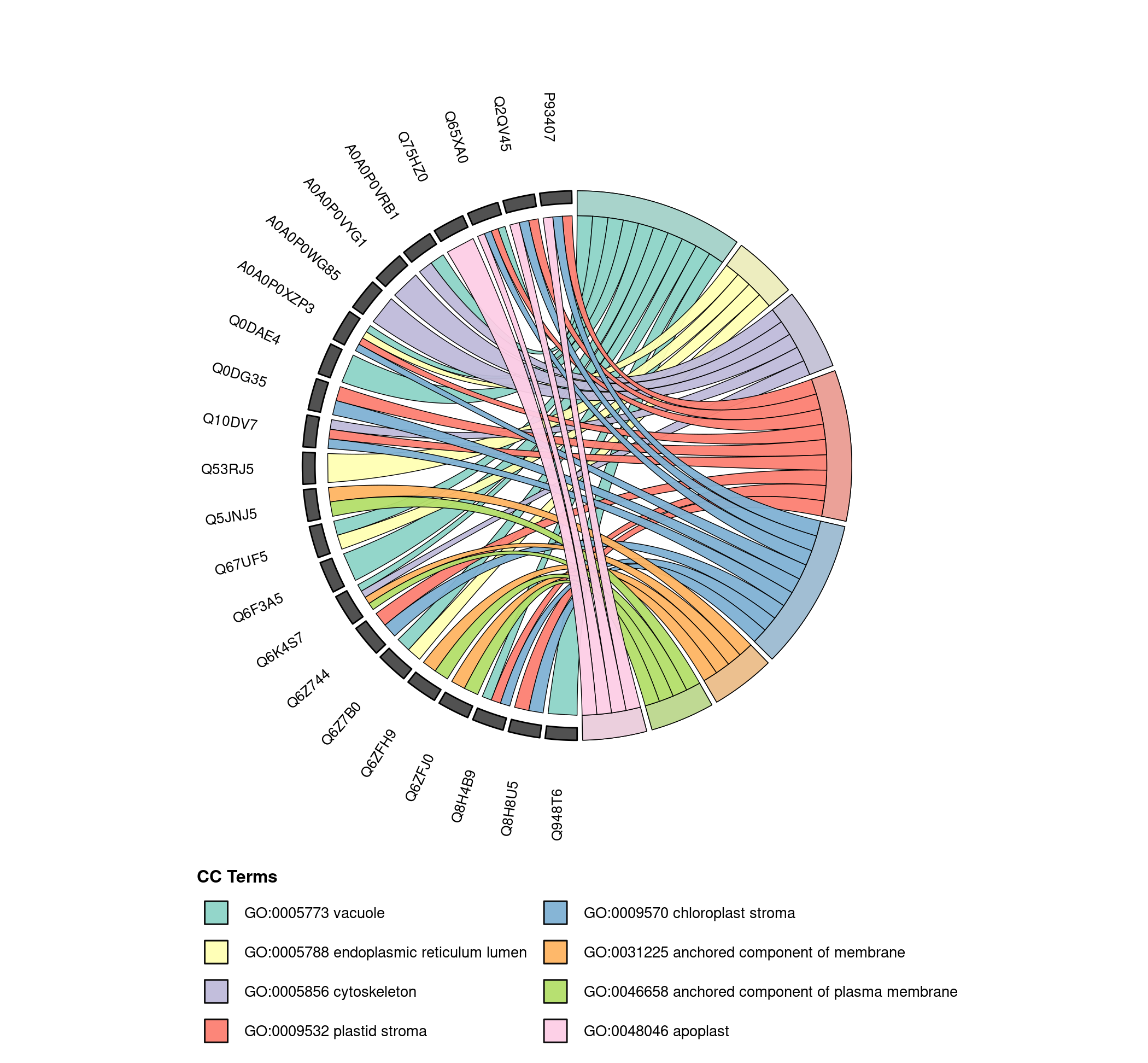

Supplement: Supplemental Information 2 — Supplemental Figures: Figure S1A: Peptide length, peptides per protein, distribution of coverage (%) and MW (kDa) of the LC-MS/MS analysis of rHSA from company A. Figure S2B: Peptide length, peptides per protein, distribution of coverage (%) and MW(kDa) of the LC-MS/MS analysis of rHSA from company B. Figure S3C: Peptide length, peptides per protein, distribution of coverage (%) and MW(kDa) of the LC-MS/MS analysis of pHSA from company C. Figure S4D: Peptide length, peptides per protein, distribution of coverage (%) and MW(kDa) of the LC-MS/MS analysis of pHSA from company D. Figure S5E: Peptide length, peptides per protein, distribution of coverage (%) and MW(kDa) of the LC-MS/MS analysis of pHSA from company E. Figure S6F: Peptide length, peptides per protein, distribution of coverage (%) and MW(kDa) of the LC-MS/MS analysis of pHSA from company F. Figure S7G: Peptide length, peptides per protein, distribution of coverage (%) and MW(kDa) of the LC-MS/MS analysis of pHSA from company G. Figure S8H: Peptide length, peptides per protein, distribution of coverage (%) and MW(kDa) of the LC-MS/MS analysis of pHSA from company H. Figure S9: GO enrichment analysis of the APs in pHSA. Figure S10: Subcellular localization prediction of the APs in pHSA. Figure S11: COG/KOG enrichment analysis of the APs in pHSA. Figure S12: KEGG pathway enrichment analysis of the APs in pHSA. Supplemental Tables: Table S1A: The protein and peptide identified in rHSA from company A. Table S2B: The protein and peptide identified in rHSA from company B. Table S3C: The protein and peptide identified in pHSA from company C. Table S4D: The protein and peptide identified in pHSA from company D. Table S5E: The protein and peptide identified in pHSA from company E. Table S6F: The protein and peptide identified in pHSA from company F. Table S7G: The protein and peptide identified in pHSA from company G. Table S8H: The protein and peptide identified in pHSA from company H. Table S9: The relative abunda [file peerj-13-19624-s002.zip › Supplementary/Supplementary File/Supplementary File2/3-Functional_enrichment/ident-CC_cirplot.png]

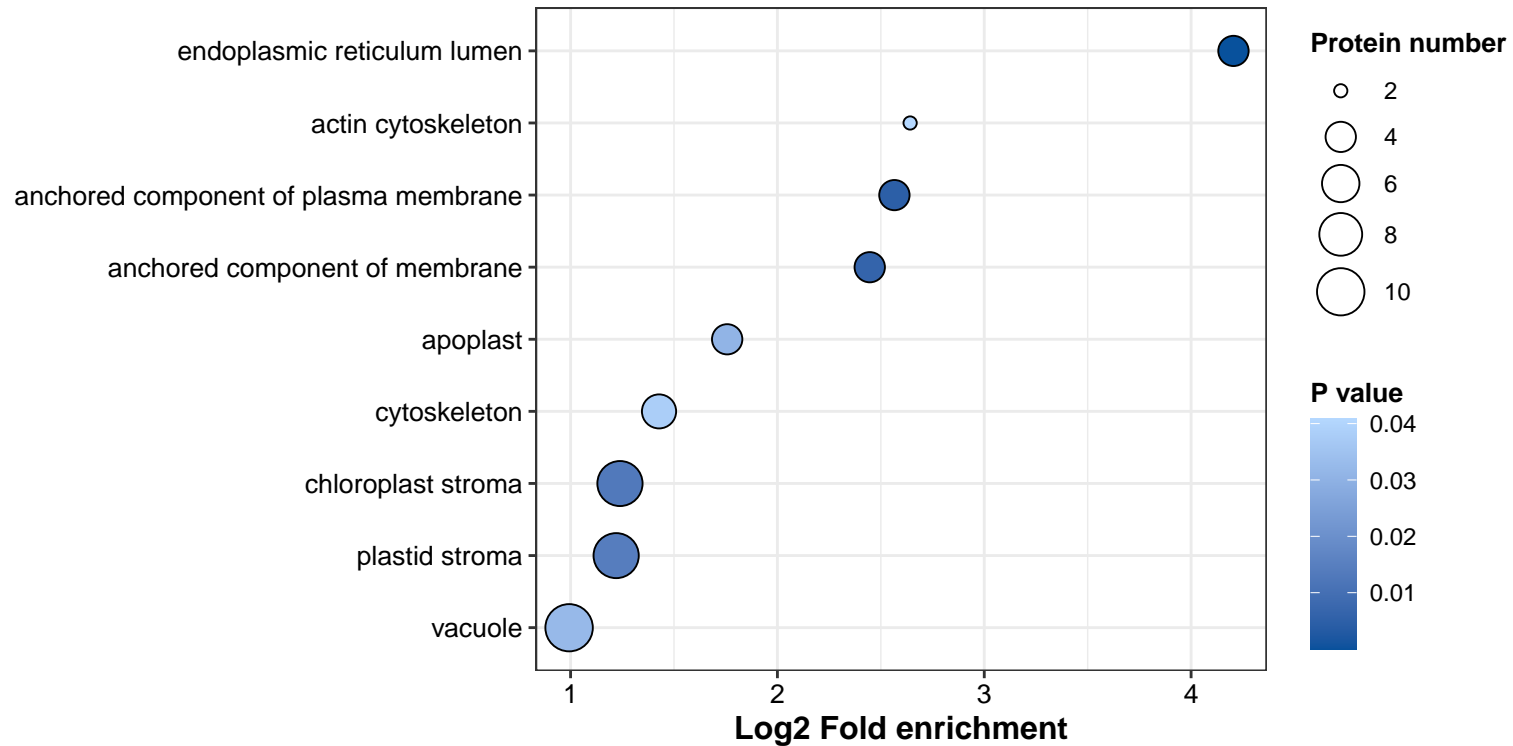

Supplement: Supplemental Information 2 — Supplemental Figures: Figure S1A: Peptide length, peptides per protein, distribution of coverage (%) and MW (kDa) of the LC-MS/MS analysis of rHSA from company A. Figure S2B: Peptide length, peptides per protein, distribution of coverage (%) and MW(kDa) of the LC-MS/MS analysis of rHSA from company B. Figure S3C: Peptide length, peptides per protein, distribution of coverage (%) and MW(kDa) of the LC-MS/MS analysis of pHSA from company C. Figure S4D: Peptide length, peptides per protein, distribution of coverage (%) and MW(kDa) of the LC-MS/MS analysis of pHSA from company D. Figure S5E: Peptide length, peptides per protein, distribution of coverage (%) and MW(kDa) of the LC-MS/MS analysis of pHSA from company E. Figure S6F: Peptide length, peptides per protein, distribution of coverage (%) and MW(kDa) of the LC-MS/MS analysis of pHSA from company F. Figure S7G: Peptide length, peptides per protein, distribution of coverage (%) and MW(kDa) of the LC-MS/MS analysis of pHSA from company G. Figure S8H: Peptide length, peptides per protein, distribution of coverage (%) and MW(kDa) of the LC-MS/MS analysis of pHSA from company H. Figure S9: GO enrichment analysis of the APs in pHSA. Figure S10: Subcellular localization prediction of the APs in pHSA. Figure S11: COG/KOG enrichment analysis of the APs in pHSA. Figure S12: KEGG pathway enrichment analysis of the APs in pHSA. Supplemental Tables: Table S1A: The protein and peptide identified in rHSA from company A. Table S2B: The protein and peptide identified in rHSA from company B. Table S3C: The protein and peptide identified in pHSA from company C. Table S4D: The protein and peptide identified in pHSA from company D. Table S5E: The protein and peptide identified in pHSA from company E. Table S6F: The protein and peptide identified in pHSA from company F. Table S7G: The protein and peptide identified in pHSA from company G. Table S8H: The protein and peptide identified in pHSA from company H. Table S9: The relative abunda [file peerj-13-19624-s002.zip › Supplementary/Supplementary File/Supplementary File2/3-Functional_enrichment/ident-CC_dotplot.pdf]

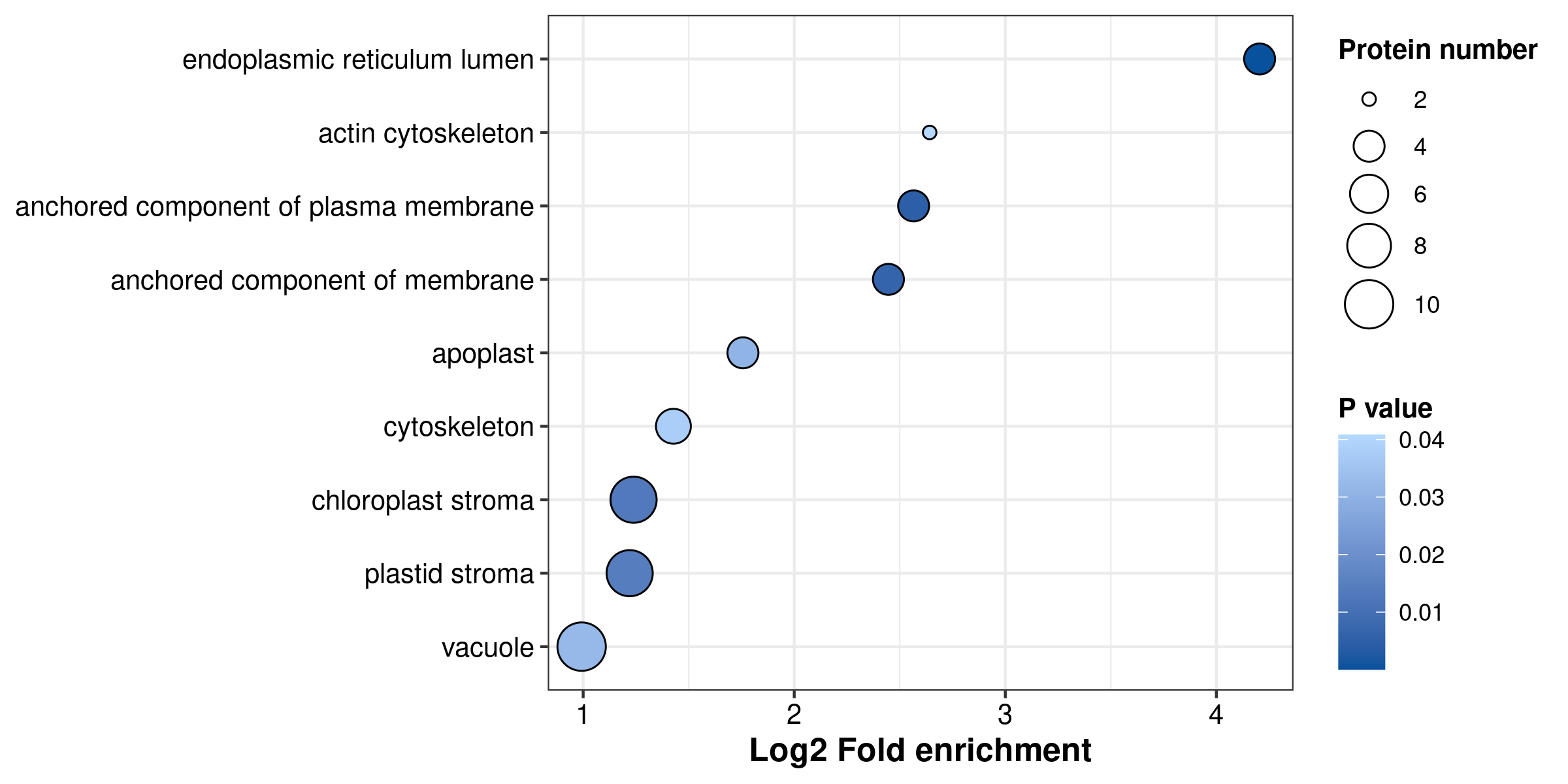

Supplement: Supplemental Information 2 — Supplemental Figures: Figure S1A: Peptide length, peptides per protein, distribution of coverage (%) and MW (kDa) of the LC-MS/MS analysis of rHSA from company A. Figure S2B: Peptide length, peptides per protein, distribution of coverage (%) and MW(kDa) of the LC-MS/MS analysis of rHSA from company B. Figure S3C: Peptide length, peptides per protein, distribution of coverage (%) and MW(kDa) of the LC-MS/MS analysis of pHSA from company C. Figure S4D: Peptide length, peptides per protein, distribution of coverage (%) and MW(kDa) of the LC-MS/MS analysis of pHSA from company D. Figure S5E: Peptide length, peptides per protein, distribution of coverage (%) and MW(kDa) of the LC-MS/MS analysis of pHSA from company E. Figure S6F: Peptide length, peptides per protein, distribution of coverage (%) and MW(kDa) of the LC-MS/MS analysis of pHSA from company F. Figure S7G: Peptide length, peptides per protein, distribution of coverage (%) and MW(kDa) of the LC-MS/MS analysis of pHSA from company G. Figure S8H: Peptide length, peptides per protein, distribution of coverage (%) and MW(kDa) of the LC-MS/MS analysis of pHSA from company H. Figure S9: GO enrichment analysis of the APs in pHSA. Figure S10: Subcellular localization prediction of the APs in pHSA. Figure S11: COG/KOG enrichment analysis of the APs in pHSA. Figure S12: KEGG pathway enrichment analysis of the APs in pHSA. Supplemental Tables: Table S1A: The protein and peptide identified in rHSA from company A. Table S2B: The protein and peptide identified in rHSA from company B. Table S3C: The protein and peptide identified in pHSA from company C. Table S4D: The protein and peptide identified in pHSA from company D. Table S5E: The protein and peptide identified in pHSA from company E. Table S6F: The protein and peptide identified in pHSA from company F. Table S7G: The protein and peptide identified in pHSA from company G. Table S8H: The protein and peptide identified in pHSA from company H. Table S9: The relative abunda [file peerj-13-19624-s002.zip › Supplementary/Supplementary File/Supplementary File2/3-Functional_enrichment/ident-CC_dotplot.png]

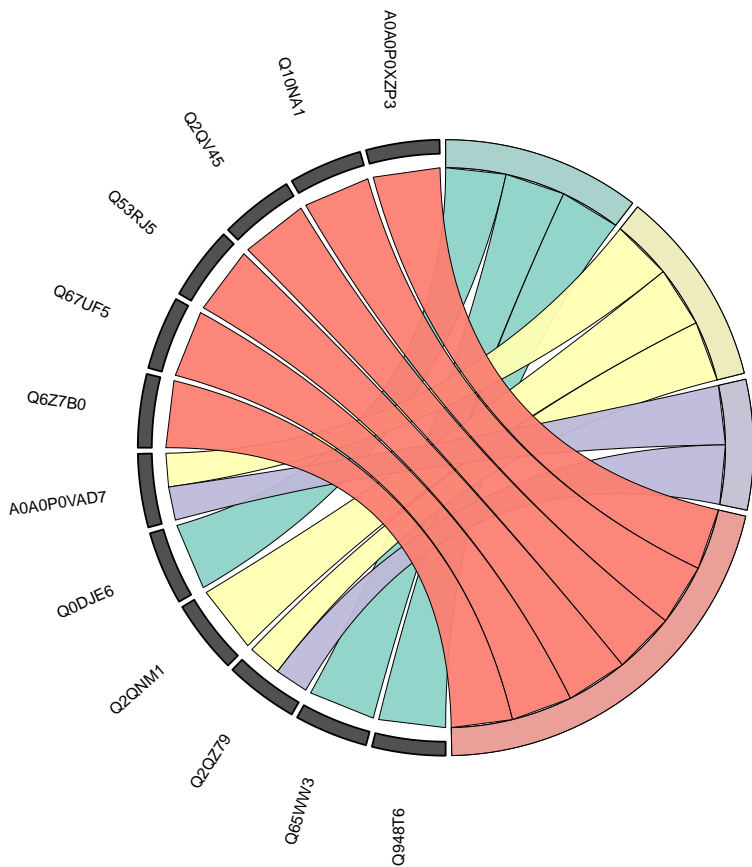

Supplement: Supplemental Information 2 — Supplemental Figures: Figure S1A: Peptide length, peptides per protein, distribution of coverage (%) and MW (kDa) of the LC-MS/MS analysis of rHSA from company A. Figure S2B: Peptide length, peptides per protein, distribution of coverage (%) and MW(kDa) of the LC-MS/MS analysis of rHSA from company B. Figure S3C: Peptide length, peptides per protein, distribution of coverage (%) and MW(kDa) of the LC-MS/MS analysis of pHSA from company C. Figure S4D: Peptide length, peptides per protein, distribution of coverage (%) and MW(kDa) of the LC-MS/MS analysis of pHSA from company D. Figure S5E: Peptide length, peptides per protein, distribution of coverage (%) and MW(kDa) of the LC-MS/MS analysis of pHSA from company E. Figure S6F: Peptide length, peptides per protein, distribution of coverage (%) and MW(kDa) of the LC-MS/MS analysis of pHSA from company F. Figure S7G: Peptide length, peptides per protein, distribution of coverage (%) and MW(kDa) of the LC-MS/MS analysis of pHSA from company G. Figure S8H: Peptide length, peptides per protein, distribution of coverage (%) and MW(kDa) of the LC-MS/MS analysis of pHSA from company H. Figure S9: GO enrichment analysis of the APs in pHSA. Figure S10: Subcellular localization prediction of the APs in pHSA. Figure S11: COG/KOG enrichment analysis of the APs in pHSA. Figure S12: KEGG pathway enrichment analysis of the APs in pHSA. Supplemental Tables: Table S1A: The protein and peptide identified in rHSA from company A. Table S2B: The protein and peptide identified in rHSA from company B. Table S3C: The protein and peptide identified in pHSA from company C. Table S4D: The protein and peptide identified in pHSA from company D. Table S5E: The protein and peptide identified in pHSA from company E. Table S6F: The protein and peptide identified in pHSA from company F. Table S7G: The protein and peptide identified in pHSA from company G. Table S8H: The protein and peptide identified in pHSA from company H. Table S9: The relative abunda [file peerj-13-19624-s002.zip › Supplementary/Supplementary File/Supplementary File2/3-Functional_enrichment/ident-KEGG_pathway_cirplot.pdf]

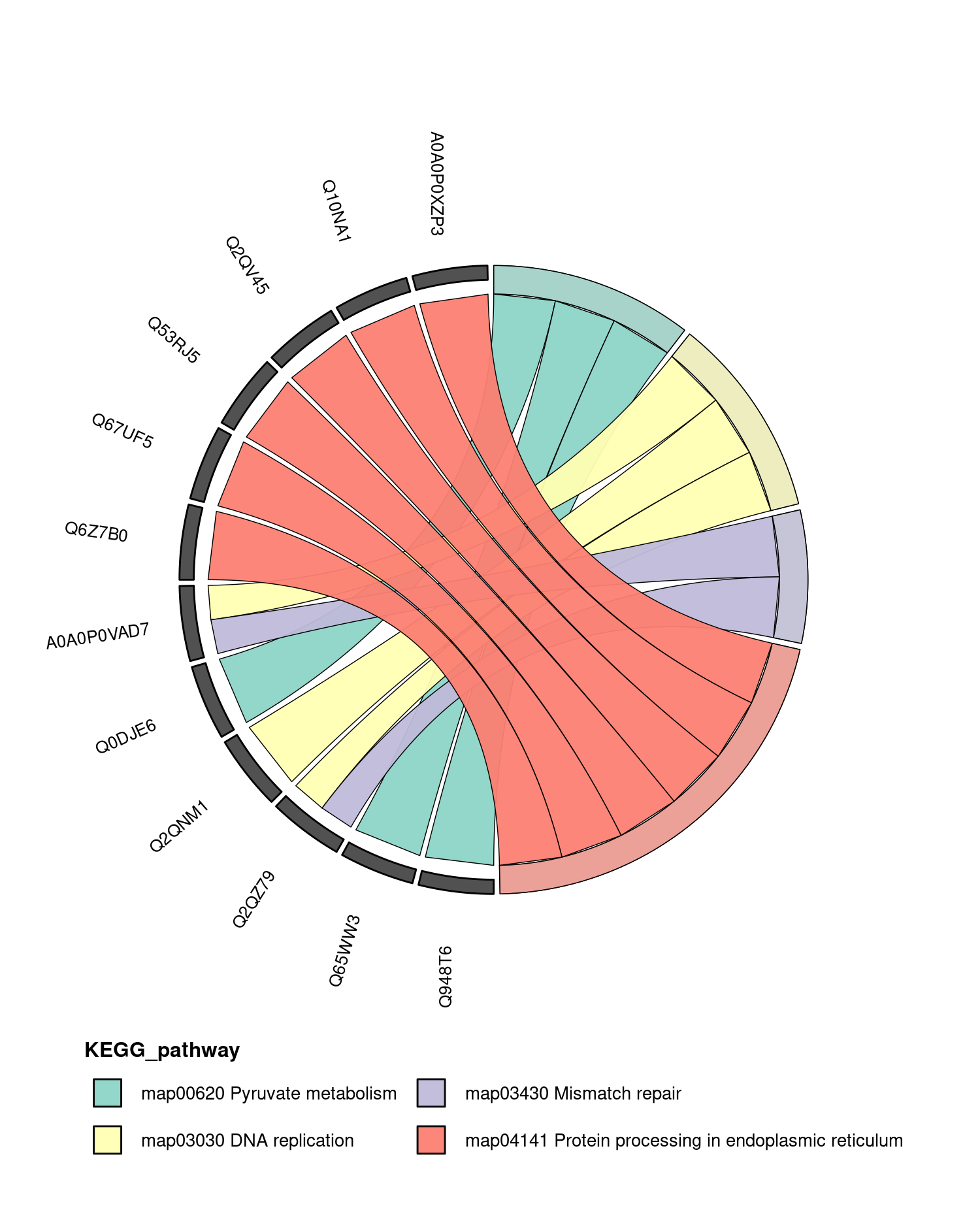

Supplement: Supplemental Information 2 — Supplemental Figures: Figure S1A: Peptide length, peptides per protein, distribution of coverage (%) and MW (kDa) of the LC-MS/MS analysis of rHSA from company A. Figure S2B: Peptide length, peptides per protein, distribution of coverage (%) and MW(kDa) of the LC-MS/MS analysis of rHSA from company B. Figure S3C: Peptide length, peptides per protein, distribution of coverage (%) and MW(kDa) of the LC-MS/MS analysis of pHSA from company C. Figure S4D: Peptide length, peptides per protein, distribution of coverage (%) and MW(kDa) of the LC-MS/MS analysis of pHSA from company D. Figure S5E: Peptide length, peptides per protein, distribution of coverage (%) and MW(kDa) of the LC-MS/MS analysis of pHSA from company E. Figure S6F: Peptide length, peptides per protein, distribution of coverage (%) and MW(kDa) of the LC-MS/MS analysis of pHSA from company F. Figure S7G: Peptide length, peptides per protein, distribution of coverage (%) and MW(kDa) of the LC-MS/MS analysis of pHSA from company G. Figure S8H: Peptide length, peptides per protein, distribution of coverage (%) and MW(kDa) of the LC-MS/MS analysis of pHSA from company H. Figure S9: GO enrichment analysis of the APs in pHSA. Figure S10: Subcellular localization prediction of the APs in pHSA. Figure S11: COG/KOG enrichment analysis of the APs in pHSA. Figure S12: KEGG pathway enrichment analysis of the APs in pHSA. Supplemental Tables: Table S1A: The protein and peptide identified in rHSA from company A. Table S2B: The protein and peptide identified in rHSA from company B. Table S3C: The protein and peptide identified in pHSA from company C. Table S4D: The protein and peptide identified in pHSA from company D. Table S5E: The protein and peptide identified in pHSA from company E. Table S6F: The protein and peptide identified in pHSA from company F. Table S7G: The protein and peptide identified in pHSA from company G. Table S8H: The protein and peptide identified in pHSA from company H. Table S9: The relative abunda [file peerj-13-19624-s002.zip › Supplementary/Supplementary File/Supplementary File2/3-Functional_enrichment/ident-KEGG_pathway_cirplot.png]

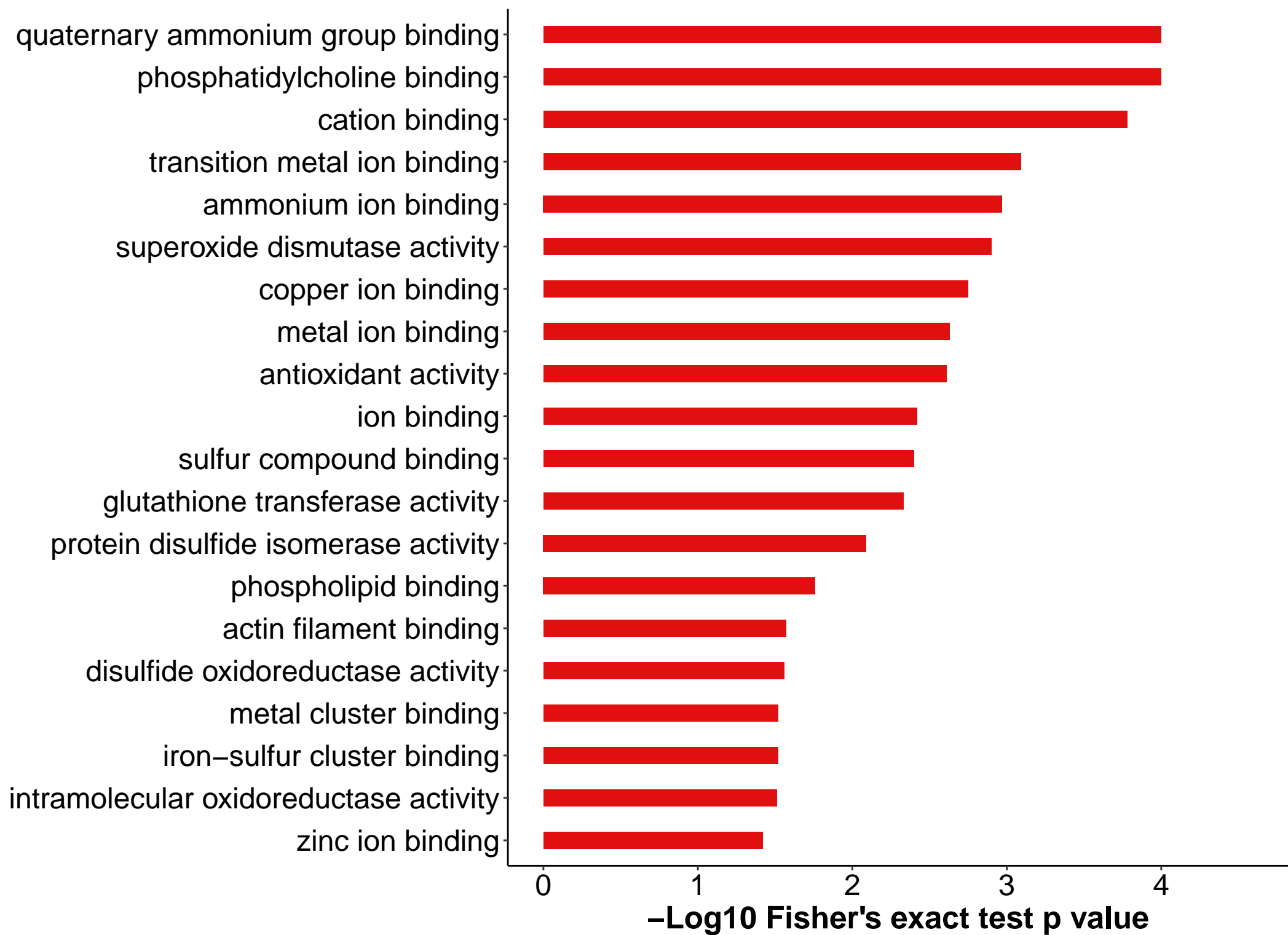

Supplement: Supplemental Information 2 — Supplemental Figures: Figure S1A: Peptide length, peptides per protein, distribution of coverage (%) and MW (kDa) of the LC-MS/MS analysis of rHSA from company A. Figure S2B: Peptide length, peptides per protein, distribution of coverage (%) and MW(kDa) of the LC-MS/MS analysis of rHSA from company B. Figure S3C: Peptide length, peptides per protein, distribution of coverage (%) and MW(kDa) of the LC-MS/MS analysis of pHSA from company C. Figure S4D: Peptide length, peptides per protein, distribution of coverage (%) and MW(kDa) of the LC-MS/MS analysis of pHSA from company D. Figure S5E: Peptide length, peptides per protein, distribution of coverage (%) and MW(kDa) of the LC-MS/MS analysis of pHSA from company E. Figure S6F: Peptide length, peptides per protein, distribution of coverage (%) and MW(kDa) of the LC-MS/MS analysis of pHSA from company F. Figure S7G: Peptide length, peptides per protein, distribution of coverage (%) and MW(kDa) of the LC-MS/MS analysis of pHSA from company G. Figure S8H: Peptide length, peptides per protein, distribution of coverage (%) and MW(kDa) of the LC-MS/MS analysis of pHSA from company H. Figure S9: GO enrichment analysis of the APs in pHSA. Figure S10: Subcellular localization prediction of the APs in pHSA. Figure S11: COG/KOG enrichment analysis of the APs in pHSA. Figure S12: KEGG pathway enrichment analysis of the APs in pHSA. Supplemental Tables: Table S1A: The protein and peptide identified in rHSA from company A. Table S2B: The protein and peptide identified in rHSA from company B. Table S3C: The protein and peptide identified in pHSA from company C. Table S4D: The protein and peptide identified in pHSA from company D. Table S5E: The protein and peptide identified in pHSA from company E. Table S6F: The protein and peptide identified in pHSA from company F. Table S7G: The protein and peptide identified in pHSA from company G. Table S8H: The protein and peptide identified in pHSA from company H. Table S9: The relative abunda [file peerj-13-19624-s002.zip › Supplementary/Supplementary File/Supplementary File2/3-Functional_enrichment/ident-MF_barplot.pdf]

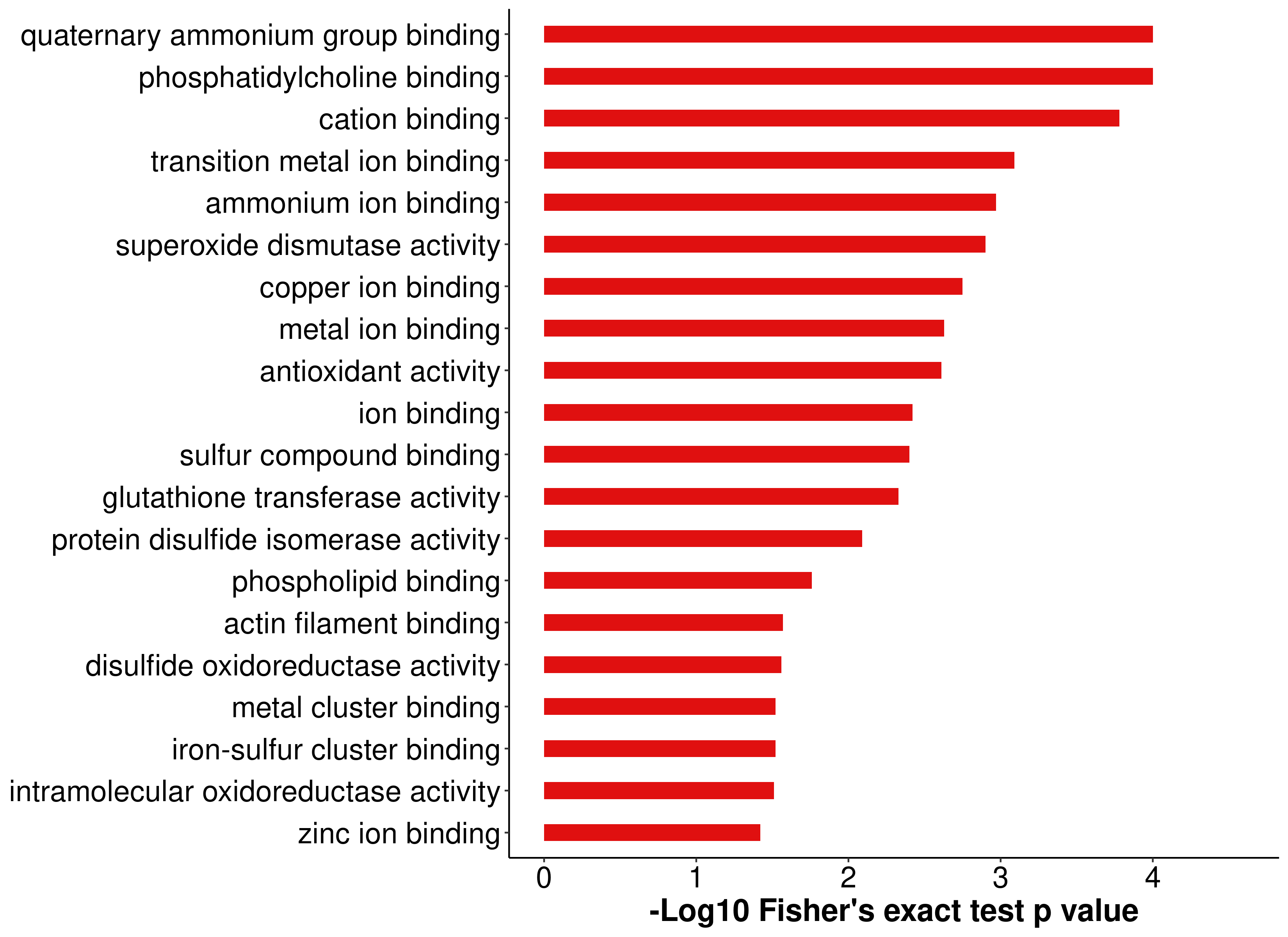

Supplement: Supplemental Information 2 — Supplemental Figures: Figure S1A: Peptide length, peptides per protein, distribution of coverage (%) and MW (kDa) of the LC-MS/MS analysis of rHSA from company A. Figure S2B: Peptide length, peptides per protein, distribution of coverage (%) and MW(kDa) of the LC-MS/MS analysis of rHSA from company B. Figure S3C: Peptide length, peptides per protein, distribution of coverage (%) and MW(kDa) of the LC-MS/MS analysis of pHSA from company C. Figure S4D: Peptide length, peptides per protein, distribution of coverage (%) and MW(kDa) of the LC-MS/MS analysis of pHSA from company D. Figure S5E: Peptide length, peptides per protein, distribution of coverage (%) and MW(kDa) of the LC-MS/MS analysis of pHSA from company E. Figure S6F: Peptide length, peptides per protein, distribution of coverage (%) and MW(kDa) of the LC-MS/MS analysis of pHSA from company F. Figure S7G: Peptide length, peptides per protein, distribution of coverage (%) and MW(kDa) of the LC-MS/MS analysis of pHSA from company G. Figure S8H: Peptide length, peptides per protein, distribution of coverage (%) and MW(kDa) of the LC-MS/MS analysis of pHSA from company H. Figure S9: GO enrichment analysis of the APs in pHSA. Figure S10: Subcellular localization prediction of the APs in pHSA. Figure S11: COG/KOG enrichment analysis of the APs in pHSA. Figure S12: KEGG pathway enrichment analysis of the APs in pHSA. Supplemental Tables: Table S1A: The protein and peptide identified in rHSA from company A. Table S2B: The protein and peptide identified in rHSA from company B. Table S3C: The protein and peptide identified in pHSA from company C. Table S4D: The protein and peptide identified in pHSA from company D. Table S5E: The protein and peptide identified in pHSA from company E. Table S6F: The protein and peptide identified in pHSA from company F. Table S7G: The protein and peptide identified in pHSA from company G. Table S8H: The protein and peptide identified in pHSA from company H. Table S9: The relative abunda [file peerj-13-19624-s002.zip › Supplementary/Supplementary File/Supplementary File2/3-Functional_enrichment/ident-MF_barplot.png]

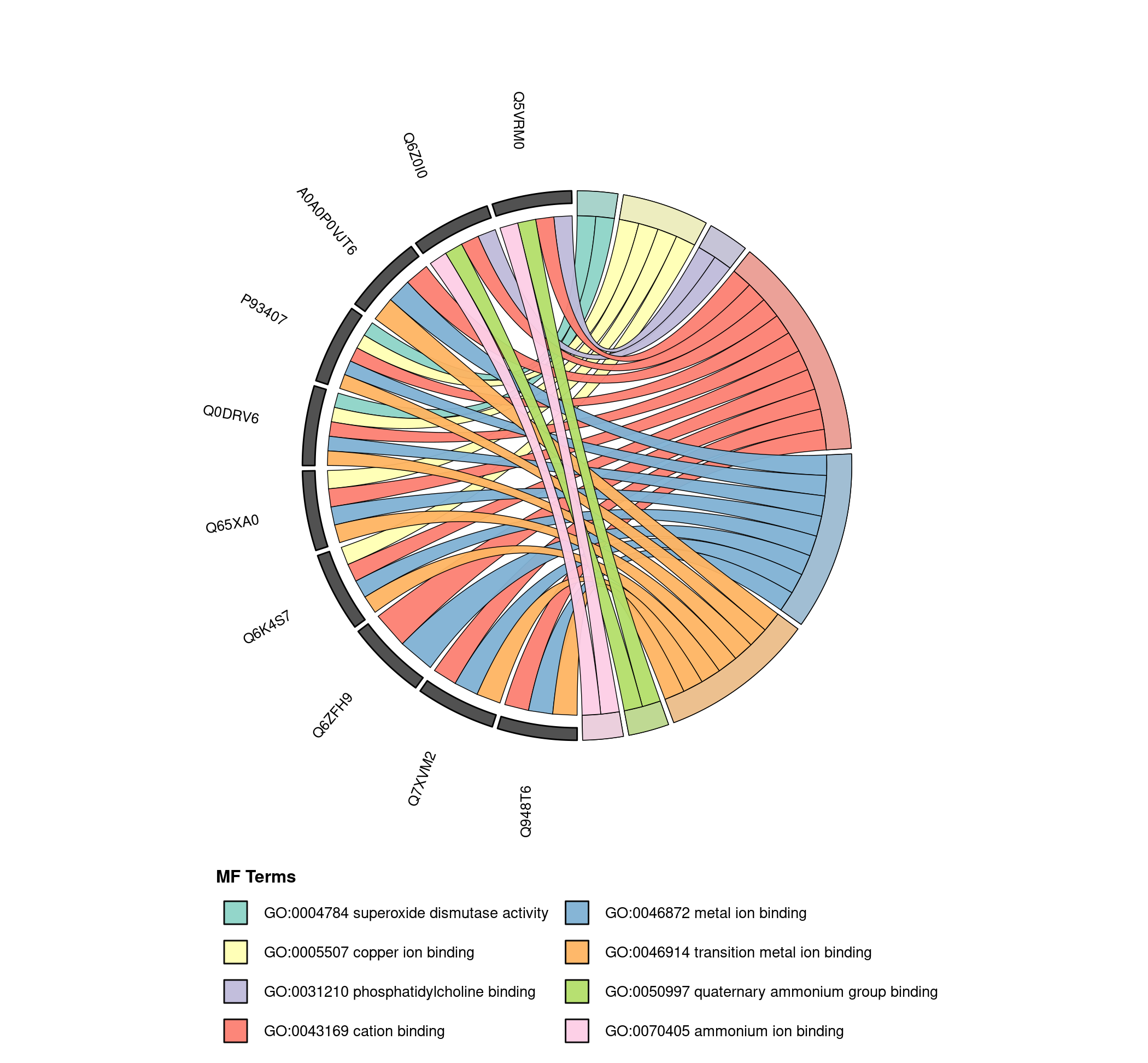

Supplement: Supplemental Information 2 — Supplemental Figures: Figure S1A: Peptide length, peptides per protein, distribution of coverage (%) and MW (kDa) of the LC-MS/MS analysis of rHSA from company A. Figure S2B: Peptide length, peptides per protein, distribution of coverage (%) and MW(kDa) of the LC-MS/MS analysis of rHSA from company B. Figure S3C: Peptide length, peptides per protein, distribution of coverage (%) and MW(kDa) of the LC-MS/MS analysis of pHSA from company C. Figure S4D: Peptide length, peptides per protein, distribution of coverage (%) and MW(kDa) of the LC-MS/MS analysis of pHSA from company D. Figure S5E: Peptide length, peptides per protein, distribution of coverage (%) and MW(kDa) of the LC-MS/MS analysis of pHSA from company E. Figure S6F: Peptide length, peptides per protein, distribution of coverage (%) and MW(kDa) of the LC-MS/MS analysis of pHSA from company F. Figure S7G: Peptide length, peptides per protein, distribution of coverage (%) and MW(kDa) of the LC-MS/MS analysis of pHSA from company G. Figure S8H: Peptide length, peptides per protein, distribution of coverage (%) and MW(kDa) of the LC-MS/MS analysis of pHSA from company H. Figure S9: GO enrichment analysis of the APs in pHSA. Figure S10: Subcellular localization prediction of the APs in pHSA. Figure S11: COG/KOG enrichment analysis of the APs in pHSA. Figure S12: KEGG pathway enrichment analysis of the APs in pHSA. Supplemental Tables: Table S1A: The protein and peptide identified in rHSA from company A. Table S2B: The protein and peptide identified in rHSA from company B. Table S3C: The protein and peptide identified in pHSA from company C. Table S4D: The protein and peptide identified in pHSA from company D. Table S5E: The protein and peptide identified in pHSA from company E. Table S6F: The protein and peptide identified in pHSA from company F. Table S7G: The protein and peptide identified in pHSA from company G. Table S8H: The protein and peptide identified in pHSA from company H. Table S9: The relative abunda [file peerj-13-19624-s002.zip › Supplementary/Supplementary File/Supplementary File2/3-Functional_enrichment/ident-MF_cirplot.png]

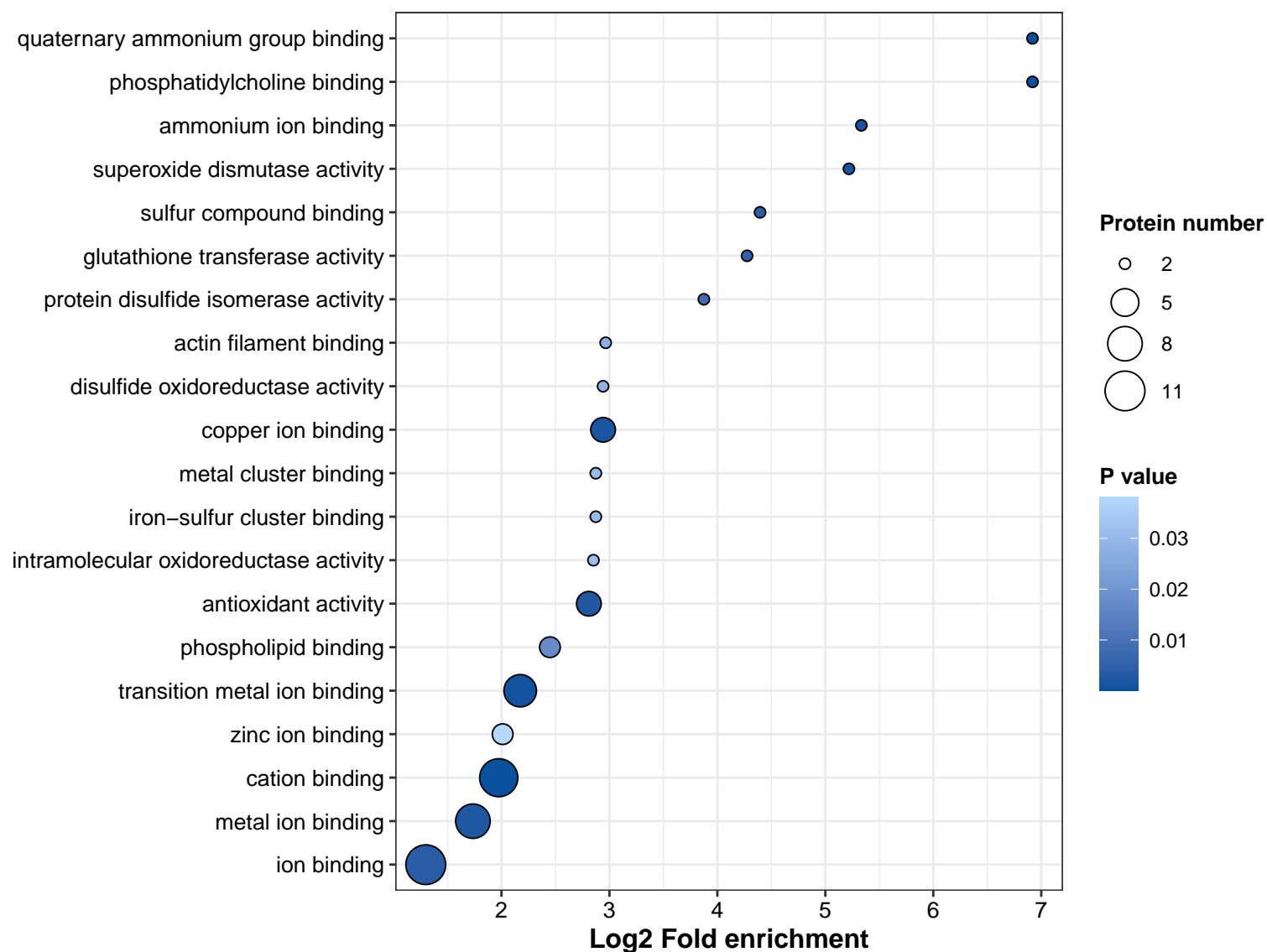

Supplement: Supplemental Information 2 — Supplemental Figures: Figure S1A: Peptide length, peptides per protein, distribution of coverage (%) and MW (kDa) of the LC-MS/MS analysis of rHSA from company A. Figure S2B: Peptide length, peptides per protein, distribution of coverage (%) and MW(kDa) of the LC-MS/MS analysis of rHSA from company B. Figure S3C: Peptide length, peptides per protein, distribution of coverage (%) and MW(kDa) of the LC-MS/MS analysis of pHSA from company C. Figure S4D: Peptide length, peptides per protein, distribution of coverage (%) and MW(kDa) of the LC-MS/MS analysis of pHSA from company D. Figure S5E: Peptide length, peptides per protein, distribution of coverage (%) and MW(kDa) of the LC-MS/MS analysis of pHSA from company E. Figure S6F: Peptide length, peptides per protein, distribution of coverage (%) and MW(kDa) of the LC-MS/MS analysis of pHSA from company F. Figure S7G: Peptide length, peptides per protein, distribution of coverage (%) and MW(kDa) of the LC-MS/MS analysis of pHSA from company G. Figure S8H: Peptide length, peptides per protein, distribution of coverage (%) and MW(kDa) of the LC-MS/MS analysis of pHSA from company H. Figure S9: GO enrichment analysis of the APs in pHSA. Figure S10: Subcellular localization prediction of the APs in pHSA. Figure S11: COG/KOG enrichment analysis of the APs in pHSA. Figure S12: KEGG pathway enrichment analysis of the APs in pHSA. Supplemental Tables: Table S1A: The protein and peptide identified in rHSA from company A. Table S2B: The protein and peptide identified in rHSA from company B. Table S3C: The protein and peptide identified in pHSA from company C. Table S4D: The protein and peptide identified in pHSA from company D. Table S5E: The protein and peptide identified in pHSA from company E. Table S6F: The protein and peptide identified in pHSA from company F. Table S7G: The protein and peptide identified in pHSA from company G. Table S8H: The protein and peptide identified in pHSA from company H. Table S9: The relative abunda [file peerj-13-19624-s002.zip › Supplementary/Supplementary File/Supplementary File2/3-Functional_enrichment/ident-MF_dotplot.pdf]

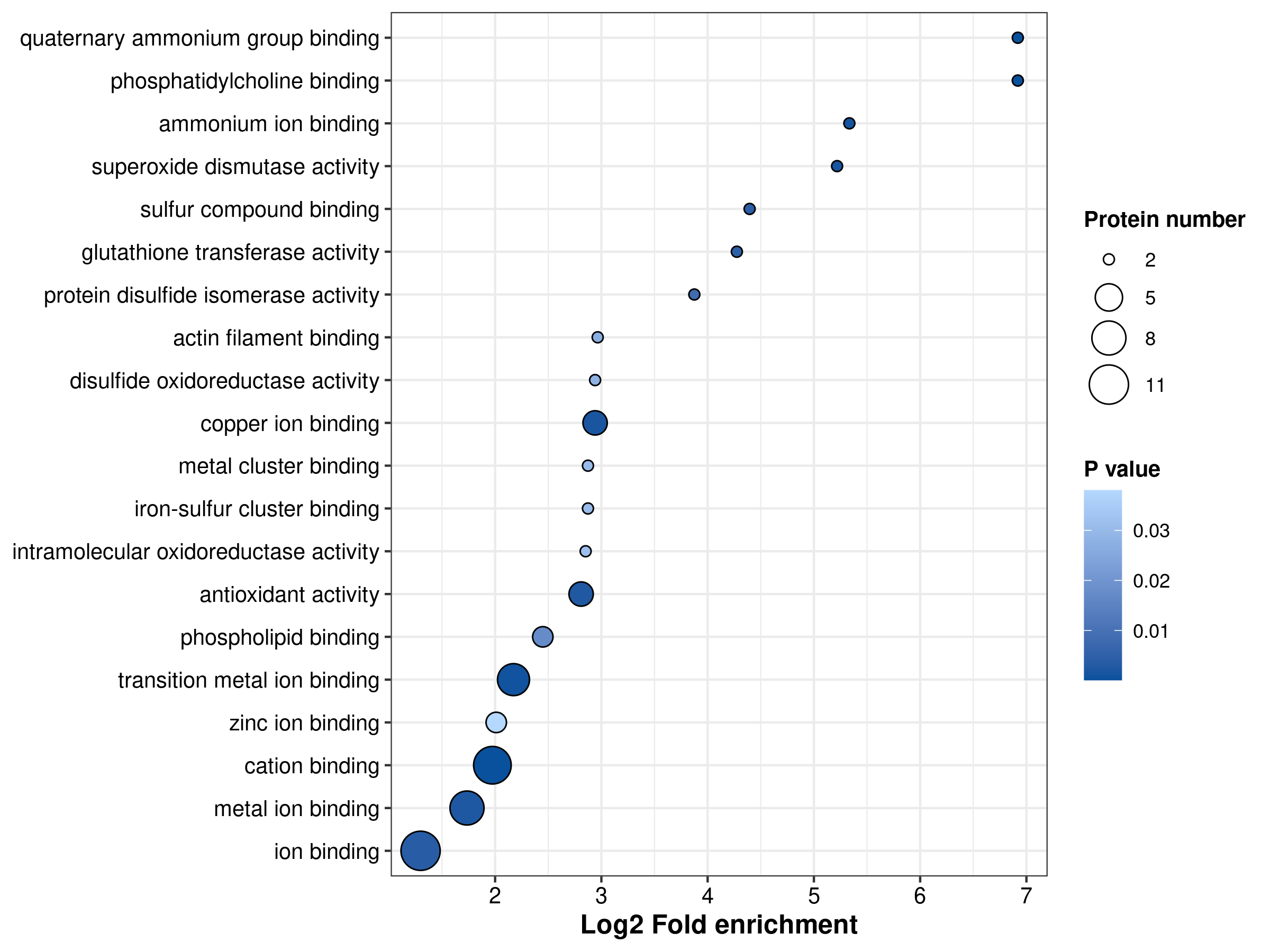

Supplement: Supplemental Information 2 — Supplemental Figures: Figure S1A: Peptide length, peptides per protein, distribution of coverage (%) and MW (kDa) of the LC-MS/MS analysis of rHSA from company A. Figure S2B: Peptide length, peptides per protein, distribution of coverage (%) and MW(kDa) of the LC-MS/MS analysis of rHSA from company B. Figure S3C: Peptide length, peptides per protein, distribution of coverage (%) and MW(kDa) of the LC-MS/MS analysis of pHSA from company C. Figure S4D: Peptide length, peptides per protein, distribution of coverage (%) and MW(kDa) of the LC-MS/MS analysis of pHSA from company D. Figure S5E: Peptide length, peptides per protein, distribution of coverage (%) and MW(kDa) of the LC-MS/MS analysis of pHSA from company E. Figure S6F: Peptide length, peptides per protein, distribution of coverage (%) and MW(kDa) of the LC-MS/MS analysis of pHSA from company F. Figure S7G: Peptide length, peptides per protein, distribution of coverage (%) and MW(kDa) of the LC-MS/MS analysis of pHSA from company G. Figure S8H: Peptide length, peptides per protein, distribution of coverage (%) and MW(kDa) of the LC-MS/MS analysis of pHSA from company H. Figure S9: GO enrichment analysis of the APs in pHSA. Figure S10: Subcellular localization prediction of the APs in pHSA. Figure S11: COG/KOG enrichment analysis of the APs in pHSA. Figure S12: KEGG pathway enrichment analysis of the APs in pHSA. Supplemental Tables: Table S1A: The protein and peptide identified in rHSA from company A. Table S2B: The protein and peptide identified in rHSA from company B. Table S3C: The protein and peptide identified in pHSA from company C. Table S4D: The protein and peptide identified in pHSA from company D. Table S5E: The protein and peptide identified in pHSA from company E. Table S6F: The protein and peptide identified in pHSA from company F. Table S7G: The protein and peptide identified in pHSA from company G. Table S8H: The protein and peptide identified in pHSA from company H. Table S9: The relative abunda [file peerj-13-19624-s002.zip › Supplementary/Supplementary File/Supplementary File2/3-Functional_enrichment/ident-MF_dotplot.png]

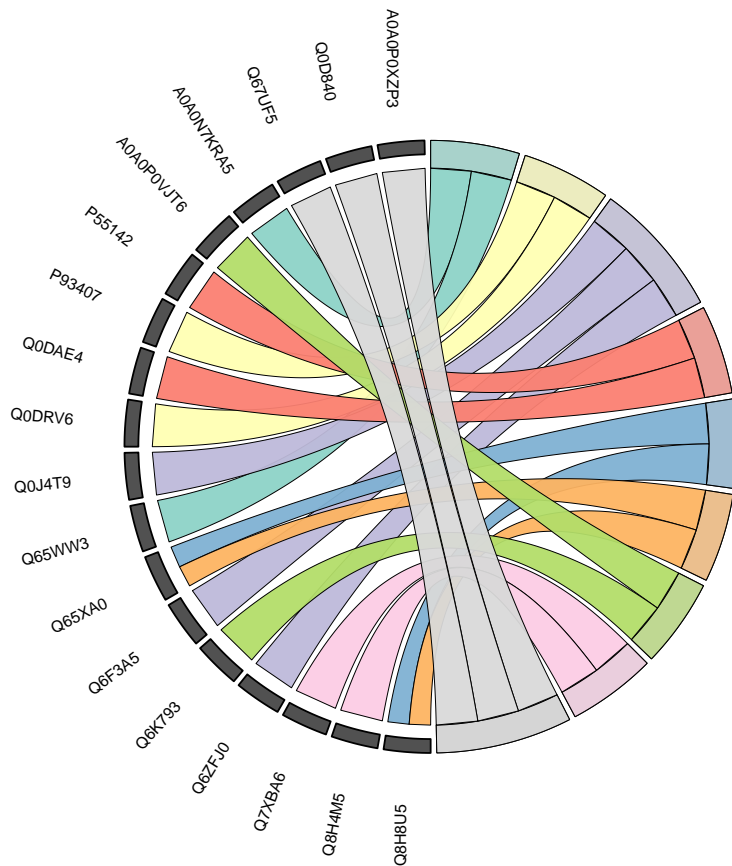

### Protein\_domain

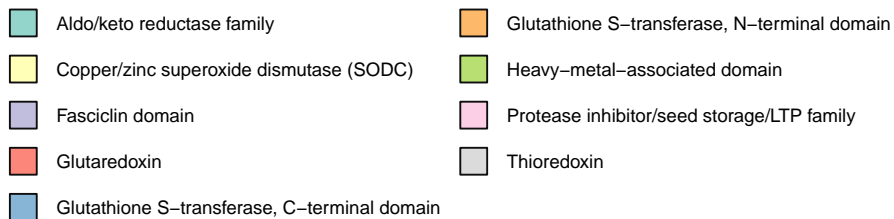

Supplement: Supplemental Information 2 — Supplemental Figures: Figure S1A: Peptide length, peptides per protein, distribution of coverage (%) and MW (kDa) of the LC-MS/MS analysis of rHSA from company A. Figure S2B: Peptide length, peptides per protein, distribution of coverage (%) and MW(kDa) of the LC-MS/MS analysis of rHSA from company B. Figure S3C: Peptide length, peptides per protein, distribution of coverage (%) and MW(kDa) of the LC-MS/MS analysis of pHSA from company C. Figure S4D: Peptide length, peptides per protein, distribution of coverage (%) and MW(kDa) of the LC-MS/MS analysis of pHSA from company D. Figure S5E: Peptide length, peptides per protein, distribution of coverage (%) and MW(kDa) of the LC-MS/MS analysis of pHSA from company E. Figure S6F: Peptide length, peptides per protein, distribution of coverage (%) and MW(kDa) of the LC-MS/MS analysis of pHSA from company F. Figure S7G: Peptide length, peptides per protein, distribution of coverage (%) and MW(kDa) of the LC-MS/MS analysis of pHSA from company G. Figure S8H: Peptide length, peptides per protein, distribution of coverage (%) and MW(kDa) of the LC-MS/MS analysis of pHSA from company H. Figure S9: GO enrichment analysis of the APs in pHSA. Figure S10: Subcellular localization prediction of the APs in pHSA. Figure S11: COG/KOG enrichment analysis of the APs in pHSA. Figure S12: KEGG pathway enrichment analysis of the APs in pHSA. Supplemental Tables: Table S1A: The protein and peptide identified in rHSA from company A. Table S2B: The protein and peptide identified in rHSA from company B. Table S3C: The protein and peptide identified in pHSA from company C. Table S4D: The protein and peptide identified in pHSA from company D. Table S5E: The protein and peptide identified in pHSA from company E. Table S6F: The protein and peptide identified in pHSA from company F. Table S7G: The protein and peptide identified in pHSA from company G. Table S8H: The protein and peptide identified in pHSA from company H. Table S9: The relative abunda [file peerj-13-19624-s002.zip › Supplementary/Supplementary File/Supplementary File2/3-Functional_enrichment/ident-Protein_domain_cirplot.pdf]

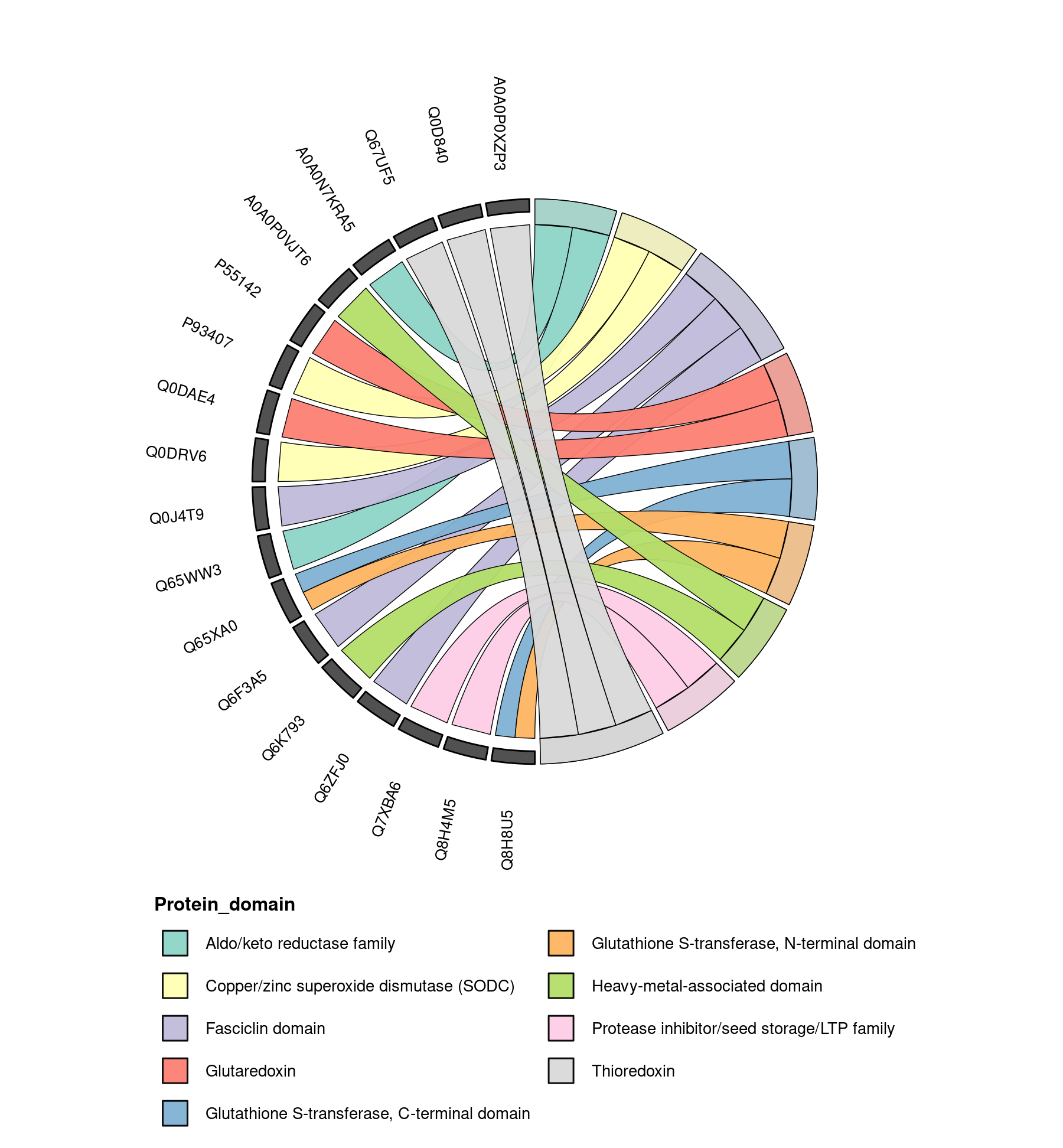

Supplement: Supplemental Information 2 — Supplemental Figures: Figure S1A: Peptide length, peptides per protein, distribution of coverage (%) and MW (kDa) of the LC-MS/MS analysis of rHSA from company A. Figure S2B: Peptide length, peptides per protein, distribution of coverage (%) and MW(kDa) of the LC-MS/MS analysis of rHSA from company B. Figure S3C: Peptide length, peptides per protein, distribution of coverage (%) and MW(kDa) of the LC-MS/MS analysis of pHSA from company C. Figure S4D: Peptide length, peptides per protein, distribution of coverage (%) and MW(kDa) of the LC-MS/MS analysis of pHSA from company D. Figure S5E: Peptide length, peptides per protein, distribution of coverage (%) and MW(kDa) of the LC-MS/MS analysis of pHSA from company E. Figure S6F: Peptide length, peptides per protein, distribution of coverage (%) and MW(kDa) of the LC-MS/MS analysis of pHSA from company F. Figure S7G: Peptide length, peptides per protein, distribution of coverage (%) and MW(kDa) of the LC-MS/MS analysis of pHSA from company G. Figure S8H: Peptide length, peptides per protein, distribution of coverage (%) and MW(kDa) of the LC-MS/MS analysis of pHSA from company H. Figure S9: GO enrichment analysis of the APs in pHSA. Figure S10: Subcellular localization prediction of the APs in pHSA. Figure S11: COG/KOG enrichment analysis of the APs in pHSA. Figure S12: KEGG pathway enrichment analysis of the APs in pHSA. Supplemental Tables: Table S1A: The protein and peptide identified in rHSA from company A. Table S2B: The protein and peptide identified in rHSA from company B. Table S3C: The protein and peptide identified in pHSA from company C. Table S4D: The protein and peptide identified in pHSA from company D. Table S5E: The protein and peptide identified in pHSA from company E. Table S6F: The protein and peptide identified in pHSA from company F. Table S7G: The protein and peptide identified in pHSA from company G. Table S8H: The protein and peptide identified in pHSA from company H. Table S9: The relative abunda [file peerj-13-19624-s002.zip › Supplementary/Supplementary File/Supplementary File2/3-Functional_enrichment/ident-Protein_domain_cirplot.png]

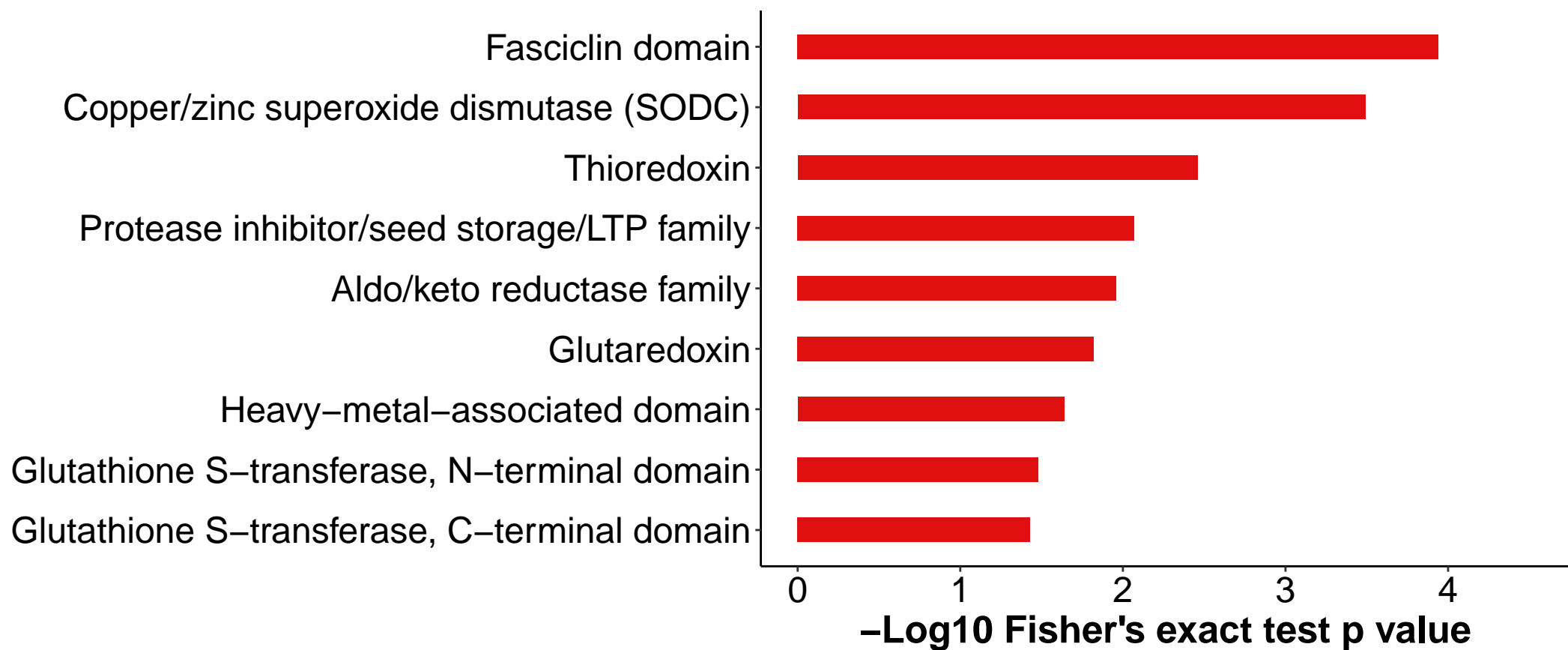

Supplement: Supplemental Information 2 — Supplemental Figures: Figure S1A: Peptide length, peptides per protein, distribution of coverage (%) and MW (kDa) of the LC-MS/MS analysis of rHSA from company A. Figure S2B: Peptide length, peptides per protein, distribution of coverage (%) and MW(kDa) of the LC-MS/MS analysis of rHSA from company B. Figure S3C: Peptide length, peptides per protein, distribution of coverage (%) and MW(kDa) of the LC-MS/MS analysis of pHSA from company C. Figure S4D: Peptide length, peptides per protein, distribution of coverage (%) and MW(kDa) of the LC-MS/MS analysis of pHSA from company D. Figure S5E: Peptide length, peptides per protein, distribution of coverage (%) and MW(kDa) of the LC-MS/MS analysis of pHSA from company E. Figure S6F: Peptide length, peptides per protein, distribution of coverage (%) and MW(kDa) of the LC-MS/MS analysis of pHSA from company F. Figure S7G: Peptide length, peptides per protein, distribution of coverage (%) and MW(kDa) of the LC-MS/MS analysis of pHSA from company G. Figure S8H: Peptide length, peptides per protein, distribution of coverage (%) and MW(kDa) of the LC-MS/MS analysis of pHSA from company H. Figure S9: GO enrichment analysis of the APs in pHSA. Figure S10: Subcellular localization prediction of the APs in pHSA. Figure S11: COG/KOG enrichment analysis of the APs in pHSA. Figure S12: KEGG pathway enrichment analysis of the APs in pHSA. Supplemental Tables: Table S1A: The protein and peptide identified in rHSA from company A. Table S2B: The protein and peptide identified in rHSA from company B. Table S3C: The protein and peptide identified in pHSA from company C. Table S4D: The protein and peptide identified in pHSA from company D. Table S5E: The protein and peptide identified in pHSA from company E. Table S6F: The protein and peptide identified in pHSA from company F. Table S7G: The protein and peptide identified in pHSA from company G. Table S8H: The protein and peptide identified in pHSA from company H. Table S9: The relative abunda [file peerj-13-19624-s002.zip › Supplementary/Supplementary File/Supplementary File2/3-Functional_enrichment/ident-domain_barplot.pdf]

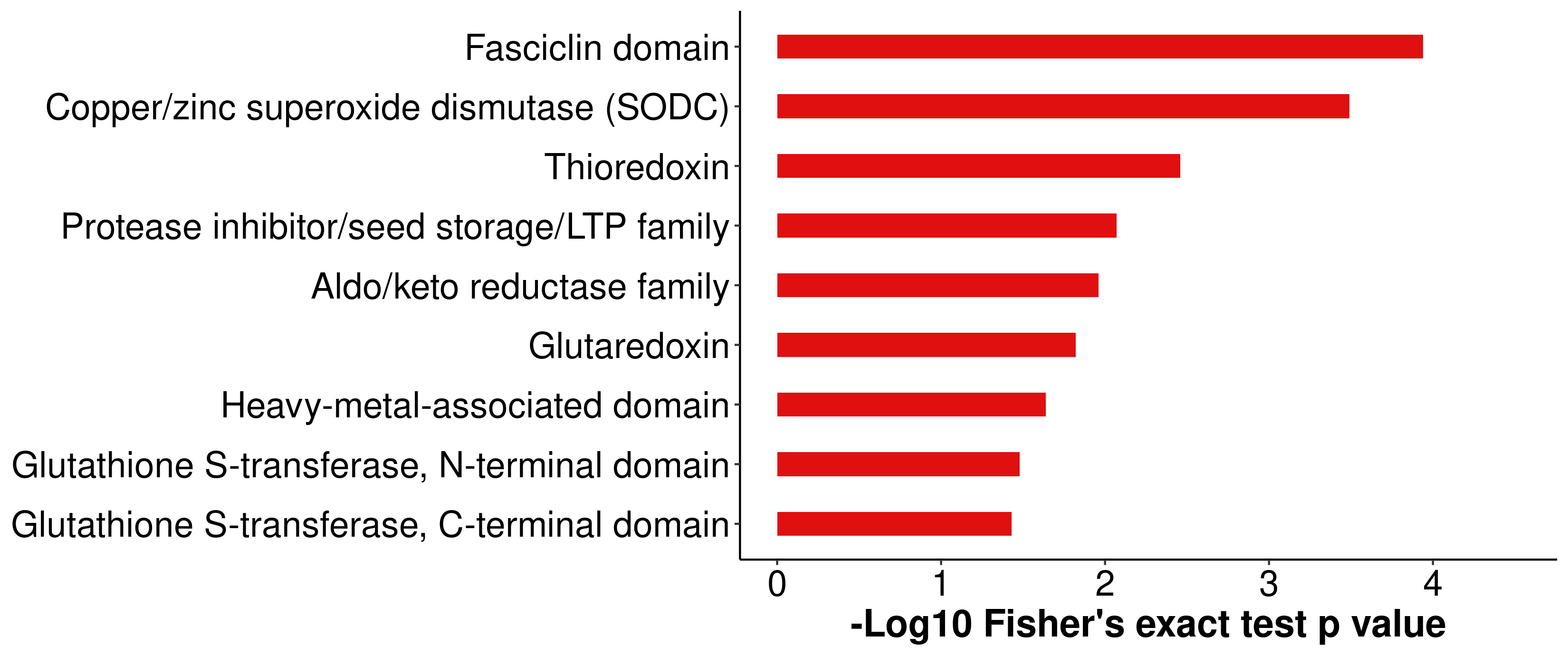

Supplement: Supplemental Information 2 — Supplemental Figures: Figure S1A: Peptide length, peptides per protein, distribution of coverage (%) and MW (kDa) of the LC-MS/MS analysis of rHSA from company A. Figure S2B: Peptide length, peptides per protein, distribution of coverage (%) and MW(kDa) of the LC-MS/MS analysis of rHSA from company B. Figure S3C: Peptide length, peptides per protein, distribution of coverage (%) and MW(kDa) of the LC-MS/MS analysis of pHSA from company C. Figure S4D: Peptide length, peptides per protein, distribution of coverage (%) and MW(kDa) of the LC-MS/MS analysis of pHSA from company D. Figure S5E: Peptide length, peptides per protein, distribution of coverage (%) and MW(kDa) of the LC-MS/MS analysis of pHSA from company E. Figure S6F: Peptide length, peptides per protein, distribution of coverage (%) and MW(kDa) of the LC-MS/MS analysis of pHSA from company F. Figure S7G: Peptide length, peptides per protein, distribution of coverage (%) and MW(kDa) of the LC-MS/MS analysis of pHSA from company G. Figure S8H: Peptide length, peptides per protein, distribution of coverage (%) and MW(kDa) of the LC-MS/MS analysis of pHSA from company H. Figure S9: GO enrichment analysis of the APs in pHSA. Figure S10: Subcellular localization prediction of the APs in pHSA. Figure S11: COG/KOG enrichment analysis of the APs in pHSA. Figure S12: KEGG pathway enrichment analysis of the APs in pHSA. Supplemental Tables: Table S1A: The protein and peptide identified in rHSA from company A. Table S2B: The protein and peptide identified in rHSA from company B. Table S3C: The protein and peptide identified in pHSA from company C. Table S4D: The protein and peptide identified in pHSA from company D. Table S5E: The protein and peptide identified in pHSA from company E. Table S6F: The protein and peptide identified in pHSA from company F. Table S7G: The protein and peptide identified in pHSA from company G. Table S8H: The protein and peptide identified in pHSA from company H. Table S9: The relative abunda [file peerj-13-19624-s002.zip › Supplementary/Supplementary File/Supplementary File2/3-Functional_enrichment/ident-domain_barplot.png]

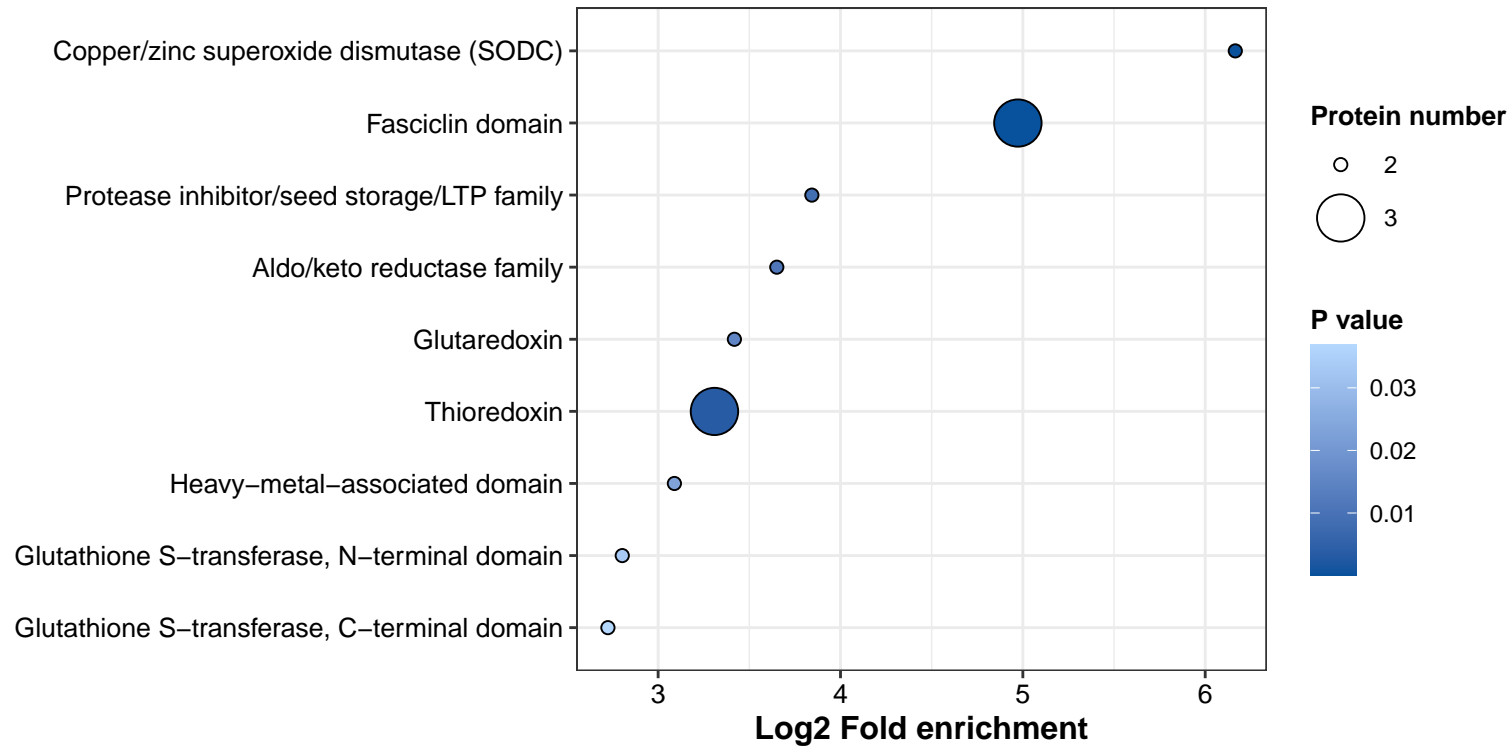

Supplement: Supplemental Information 2 — Supplemental Figures: Figure S1A: Peptide length, peptides per protein, distribution of coverage (%) and MW (kDa) of the LC-MS/MS analysis of rHSA from company A. Figure S2B: Peptide length, peptides per protein, distribution of coverage (%) and MW(kDa) of the LC-MS/MS analysis of rHSA from company B. Figure S3C: Peptide length, peptides per protein, distribution of coverage (%) and MW(kDa) of the LC-MS/MS analysis of pHSA from company C. Figure S4D: Peptide length, peptides per protein, distribution of coverage (%) and MW(kDa) of the LC-MS/MS analysis of pHSA from company D. Figure S5E: Peptide length, peptides per protein, distribution of coverage (%) and MW(kDa) of the LC-MS/MS analysis of pHSA from company E. Figure S6F: Peptide length, peptides per protein, distribution of coverage (%) and MW(kDa) of the LC-MS/MS analysis of pHSA from company F. Figure S7G: Peptide length, peptides per protein, distribution of coverage (%) and MW(kDa) of the LC-MS/MS analysis of pHSA from company G. Figure S8H: Peptide length, peptides per protein, distribution of coverage (%) and MW(kDa) of the LC-MS/MS analysis of pHSA from company H. Figure S9: GO enrichment analysis of the APs in pHSA. Figure S10: Subcellular localization prediction of the APs in pHSA. Figure S11: COG/KOG enrichment analysis of the APs in pHSA. Figure S12: KEGG pathway enrichment analysis of the APs in pHSA. Supplemental Tables: Table S1A: The protein and peptide identified in rHSA from company A. Table S2B: The protein and peptide identified in rHSA from company B. Table S3C: The protein and peptide identified in pHSA from company C. Table S4D: The protein and peptide identified in pHSA from company D. Table S5E: The protein and peptide identified in pHSA from company E. Table S6F: The protein and peptide identified in pHSA from company F. Table S7G: The protein and peptide identified in pHSA from company G. Table S8H: The protein and peptide identified in pHSA from company H. Table S9: The relative abunda [file peerj-13-19624-s002.zip › Supplementary/Supplementary File/Supplementary File2/3-Functional_enrichment/ident-domain_dotplot.pdf]

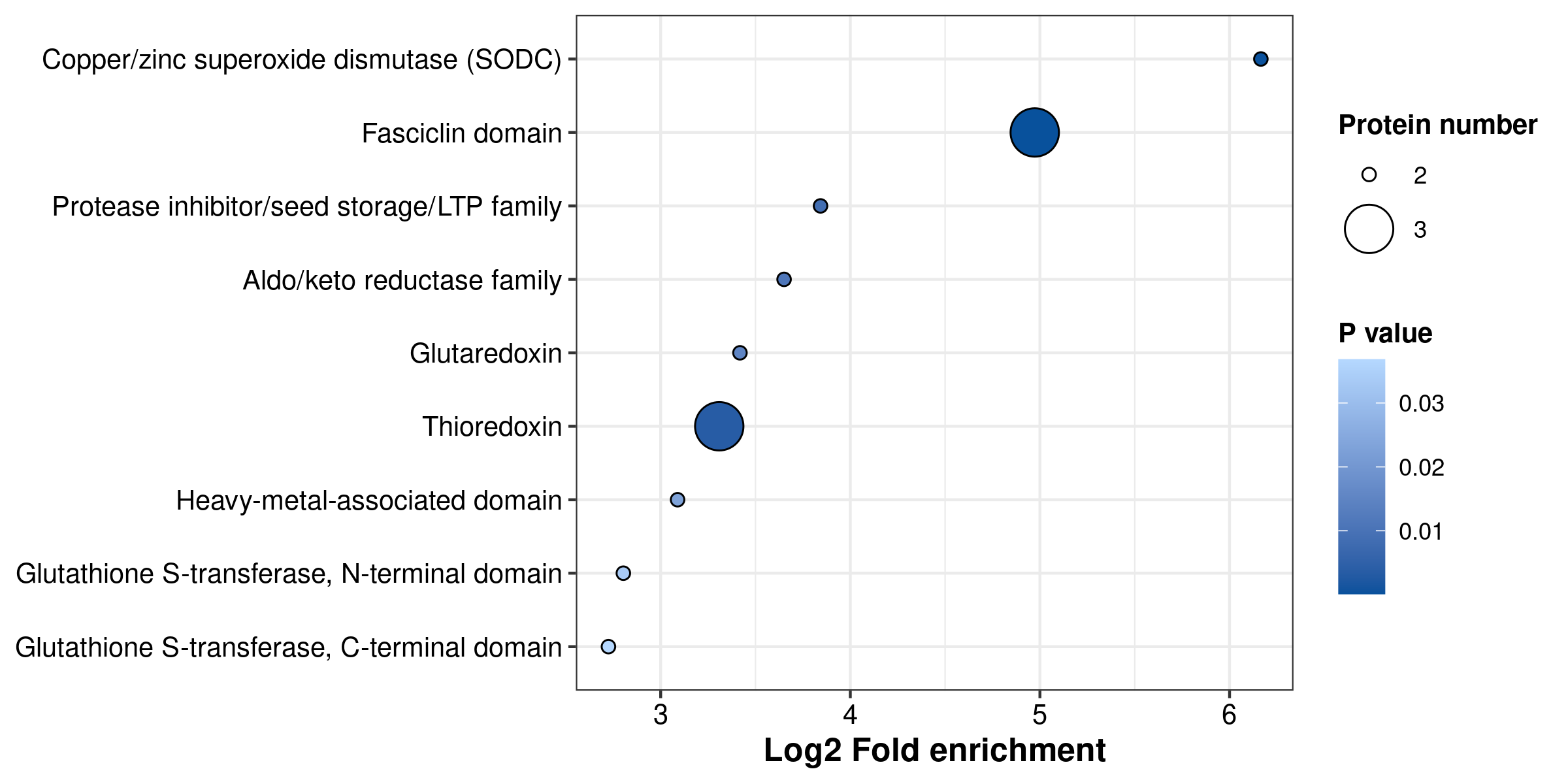

Supplement: Supplemental Information 2 — Supplemental Figures: Figure S1A: Peptide length, peptides per protein, distribution of coverage (%) and MW (kDa) of the LC-MS/MS analysis of rHSA from company A. Figure S2B: Peptide length, peptides per protein, distribution of coverage (%) and MW(kDa) of the LC-MS/MS analysis of rHSA from company B. Figure S3C: Peptide length, peptides per protein, distribution of coverage (%) and MW(kDa) of the LC-MS/MS analysis of pHSA from company C. Figure S4D: Peptide length, peptides per protein, distribution of coverage (%) and MW(kDa) of the LC-MS/MS analysis of pHSA from company D. Figure S5E: Peptide length, peptides per protein, distribution of coverage (%) and MW(kDa) of the LC-MS/MS analysis of pHSA from company E. Figure S6F: Peptide length, peptides per protein, distribution of coverage (%) and MW(kDa) of the LC-MS/MS analysis of pHSA from company F. Figure S7G: Peptide length, peptides per protein, distribution of coverage (%) and MW(kDa) of the LC-MS/MS analysis of pHSA from company G. Figure S8H: Peptide length, peptides per protein, distribution of coverage (%) and MW(kDa) of the LC-MS/MS analysis of pHSA from company H. Figure S9: GO enrichment analysis of the APs in pHSA. Figure S10: Subcellular localization prediction of the APs in pHSA. Figure S11: COG/KOG enrichment analysis of the APs in pHSA. Figure S12: KEGG pathway enrichment analysis of the APs in pHSA. Supplemental Tables: Table S1A: The protein and peptide identified in rHSA from company A. Table S2B: The protein and peptide identified in rHSA from company B. Table S3C: The protein and peptide identified in pHSA from company C. Table S4D: The protein and peptide identified in pHSA from company D. Table S5E: The protein and peptide identified in pHSA from company E. Table S6F: The protein and peptide identified in pHSA from company F. Table S7G: The protein and peptide identified in pHSA from company G. Table S8H: The protein and peptide identified in pHSA from company H. Table S9: The relative abunda [file peerj-13-19624-s002.zip › Supplementary/Supplementary File/Supplementary File2/3-Functional_enrichment/ident-domain_dotplot.png]

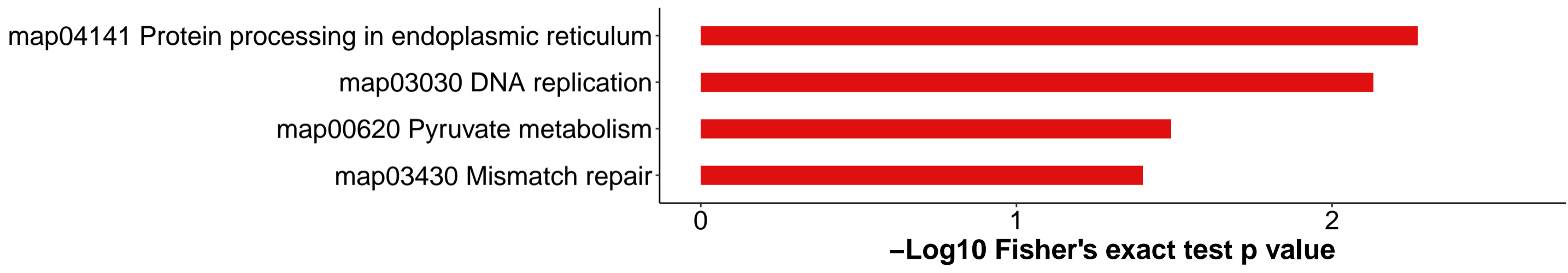

Supplement: Supplemental Information 2 — Supplemental Figures: Figure S1A: Peptide length, peptides per protein, distribution of coverage (%) and MW (kDa) of the LC-MS/MS analysis of rHSA from company A. Figure S2B: Peptide length, peptides per protein, distribution of coverage (%) and MW(kDa) of the LC-MS/MS analysis of rHSA from company B. Figure S3C: Peptide length, peptides per protein, distribution of coverage (%) and MW(kDa) of the LC-MS/MS analysis of pHSA from company C. Figure S4D: Peptide length, peptides per protein, distribution of coverage (%) and MW(kDa) of the LC-MS/MS analysis of pHSA from company D. Figure S5E: Peptide length, peptides per protein, distribution of coverage (%) and MW(kDa) of the LC-MS/MS analysis of pHSA from company E. Figure S6F: Peptide length, peptides per protein, distribution of coverage (%) and MW(kDa) of the LC-MS/MS analysis of pHSA from company F. Figure S7G: Peptide length, peptides per protein, distribution of coverage (%) and MW(kDa) of the LC-MS/MS analysis of pHSA from company G. Figure S8H: Peptide length, peptides per protein, distribution of coverage (%) and MW(kDa) of the LC-MS/MS analysis of pHSA from company H. Figure S9: GO enrichment analysis of the APs in pHSA. Figure S10: Subcellular localization prediction of the APs in pHSA. Figure S11: COG/KOG enrichment analysis of the APs in pHSA. Figure S12: KEGG pathway enrichment analysis of the APs in pHSA. Supplemental Tables: Table S1A: The protein and peptide identified in rHSA from company A. Table S2B: The protein and peptide identified in rHSA from company B. Table S3C: The protein and peptide identified in pHSA from company C. Table S4D: The protein and peptide identified in pHSA from company D. Table S5E: The protein and peptide identified in pHSA from company E. Table S6F: The protein and peptide identified in pHSA from company F. Table S7G: The protein and peptide identified in pHSA from company G. Table S8H: The protein and peptide identified in pHSA from company H. Table S9: The relative abunda [file peerj-13-19624-s002.zip › Supplementary/Supplementary File/Supplementary File2/3-Functional_enrichment/ident-kegg_barplot.pdf]

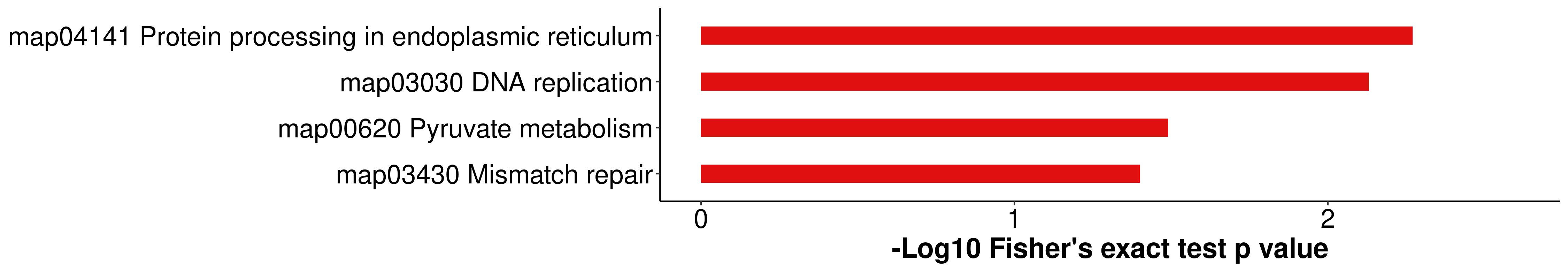

Supplement: Supplemental Information 2 — Supplemental Figures: Figure S1A: Peptide length, peptides per protein, distribution of coverage (%) and MW (kDa) of the LC-MS/MS analysis of rHSA from company A. Figure S2B: Peptide length, peptides per protein, distribution of coverage (%) and MW(kDa) of the LC-MS/MS analysis of rHSA from company B. Figure S3C: Peptide length, peptides per protein, distribution of coverage (%) and MW(kDa) of the LC-MS/MS analysis of pHSA from company C. Figure S4D: Peptide length, peptides per protein, distribution of coverage (%) and MW(kDa) of the LC-MS/MS analysis of pHSA from company D. Figure S5E: Peptide length, peptides per protein, distribution of coverage (%) and MW(kDa) of the LC-MS/MS analysis of pHSA from company E. Figure S6F: Peptide length, peptides per protein, distribution of coverage (%) and MW(kDa) of the LC-MS/MS analysis of pHSA from company F. Figure S7G: Peptide length, peptides per protein, distribution of coverage (%) and MW(kDa) of the LC-MS/MS analysis of pHSA from company G. Figure S8H: Peptide length, peptides per protein, distribution of coverage (%) and MW(kDa) of the LC-MS/MS analysis of pHSA from company H. Figure S9: GO enrichment analysis of the APs in pHSA. Figure S10: Subcellular localization prediction of the APs in pHSA. Figure S11: COG/KOG enrichment analysis of the APs in pHSA. Figure S12: KEGG pathway enrichment analysis of the APs in pHSA. Supplemental Tables: Table S1A: The protein and peptide identified in rHSA from company A. Table S2B: The protein and peptide identified in rHSA from company B. Table S3C: The protein and peptide identified in pHSA from company C. Table S4D: The protein and peptide identified in pHSA from company D. Table S5E: The protein and peptide identified in pHSA from company E. Table S6F: The protein and peptide identified in pHSA from company F. Table S7G: The protein and peptide identified in pHSA from company G. Table S8H: The protein and peptide identified in pHSA from company H. Table S9: The relative abunda [file peerj-13-19624-s002.zip › Supplementary/Supplementary File/Supplementary File2/3-Functional_enrichment/ident-kegg_barplot.png]

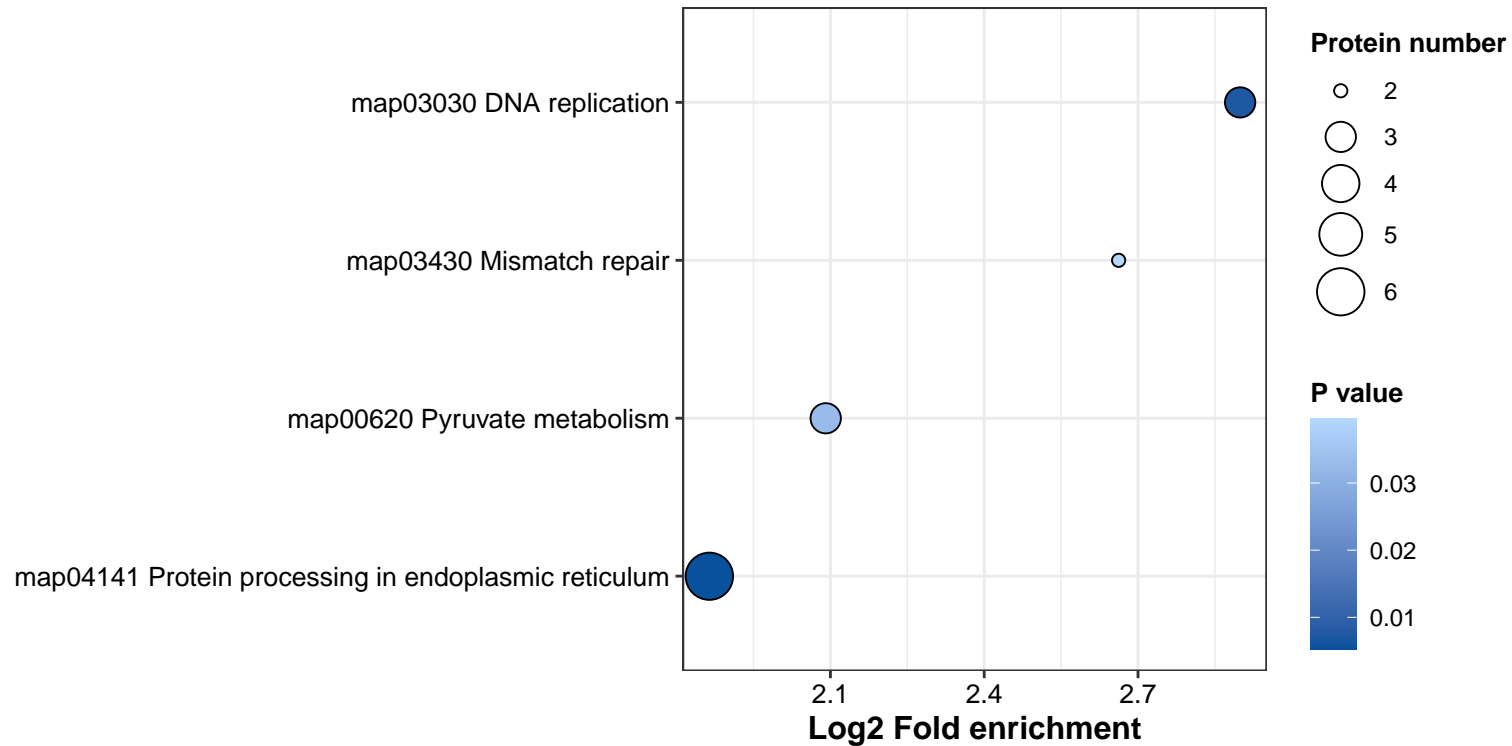

Supplement: Supplemental Information 2 — Supplemental Figures: Figure S1A: Peptide length, peptides per protein, distribution of coverage (%) and MW (kDa) of the LC-MS/MS analysis of rHSA from company A. Figure S2B: Peptide length, peptides per protein, distribution of coverage (%) and MW(kDa) of the LC-MS/MS analysis of rHSA from company B. Figure S3C: Peptide length, peptides per protein, distribution of coverage (%) and MW(kDa) of the LC-MS/MS analysis of pHSA from company C. Figure S4D: Peptide length, peptides per protein, distribution of coverage (%) and MW(kDa) of the LC-MS/MS analysis of pHSA from company D. Figure S5E: Peptide length, peptides per protein, distribution of coverage (%) and MW(kDa) of the LC-MS/MS analysis of pHSA from company E. Figure S6F: Peptide length, peptides per protein, distribution of coverage (%) and MW(kDa) of the LC-MS/MS analysis of pHSA from company F. Figure S7G: Peptide length, peptides per protein, distribution of coverage (%) and MW(kDa) of the LC-MS/MS analysis of pHSA from company G. Figure S8H: Peptide length, peptides per protein, distribution of coverage (%) and MW(kDa) of the LC-MS/MS analysis of pHSA from company H. Figure S9: GO enrichment analysis of the APs in pHSA. Figure S10: Subcellular localization prediction of the APs in pHSA. Figure S11: COG/KOG enrichment analysis of the APs in pHSA. Figure S12: KEGG pathway enrichment analysis of the APs in pHSA. Supplemental Tables: Table S1A: The protein and peptide identified in rHSA from company A. Table S2B: The protein and peptide identified in rHSA from company B. Table S3C: The protein and peptide identified in pHSA from company C. Table S4D: The protein and peptide identified in pHSA from company D. Table S5E: The protein and peptide identified in pHSA from company E. Table S6F: The protein and peptide identified in pHSA from company F. Table S7G: The protein and peptide identified in pHSA from company G. Table S8H: The protein and peptide identified in pHSA from company H. Table S9: The relative abunda [file peerj-13-19624-s002.zip › Supplementary/Supplementary File/Supplementary File2/3-Functional_enrichment/ident-kegg_dotplot.pdf]

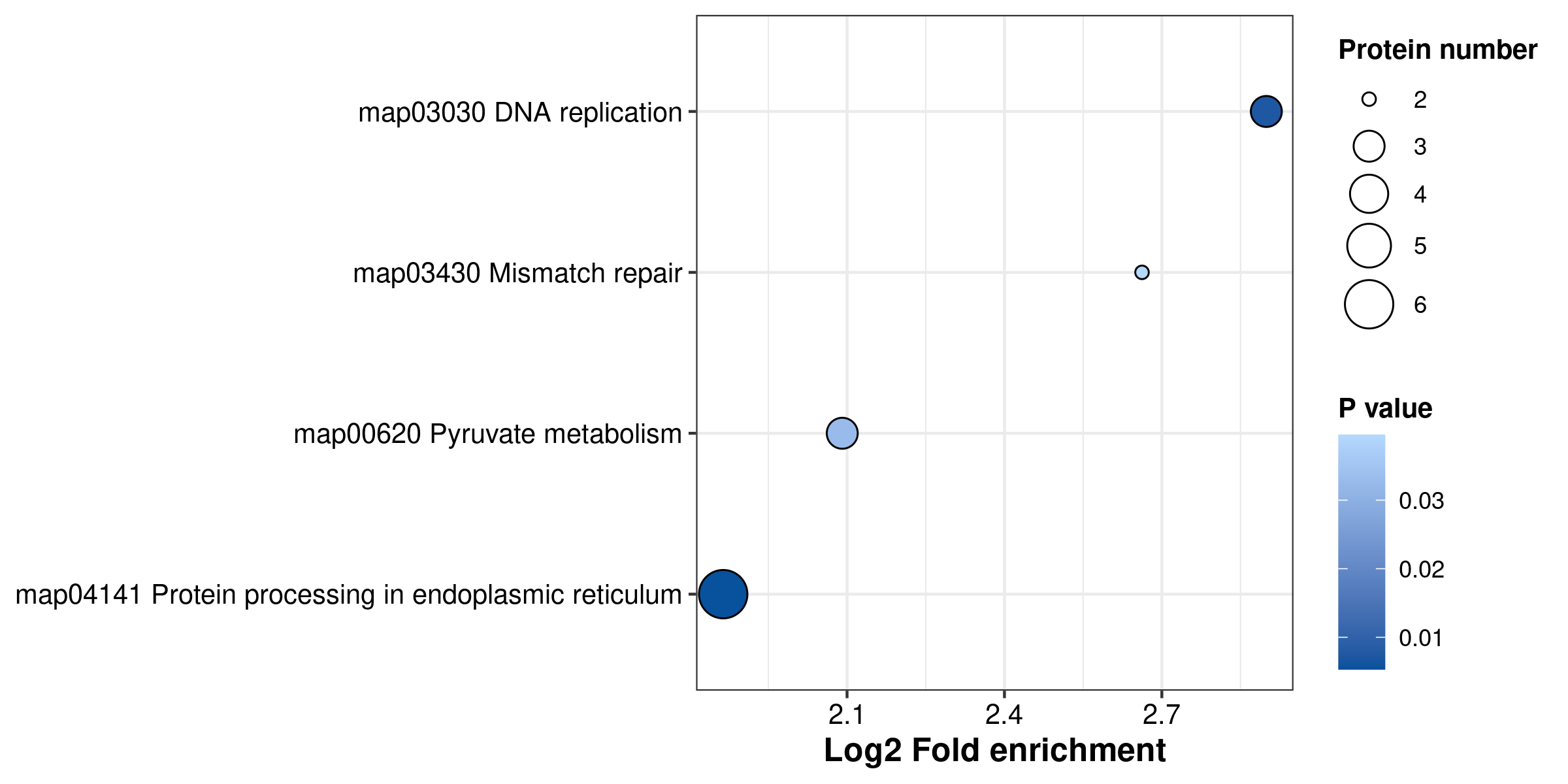

Supplement: Supplemental Information 2 — Supplemental Figures: Figure S1A: Peptide length, peptides per protein, distribution of coverage (%) and MW (kDa) of the LC-MS/MS analysis of rHSA from company A. Figure S2B: Peptide length, peptides per protein, distribution of coverage (%) and MW(kDa) of the LC-MS/MS analysis of rHSA from company B. Figure S3C: Peptide length, peptides per protein, distribution of coverage (%) and MW(kDa) of the LC-MS/MS analysis of pHSA from company C. Figure S4D: Peptide length, peptides per protein, distribution of coverage (%) and MW(kDa) of the LC-MS/MS analysis of pHSA from company D. Figure S5E: Peptide length, peptides per protein, distribution of coverage (%) and MW(kDa) of the LC-MS/MS analysis of pHSA from company E. Figure S6F: Peptide length, peptides per protein, distribution of coverage (%) and MW(kDa) of the LC-MS/MS analysis of pHSA from company F. Figure S7G: Peptide length, peptides per protein, distribution of coverage (%) and MW(kDa) of the LC-MS/MS analysis of pHSA from company G. Figure S8H: Peptide length, peptides per protein, distribution of coverage (%) and MW(kDa) of the LC-MS/MS analysis of pHSA from company H. Figure S9: GO enrichment analysis of the APs in pHSA. Figure S10: Subcellular localization prediction of the APs in pHSA. Figure S11: COG/KOG enrichment analysis of the APs in pHSA. Figure S12: KEGG pathway enrichment analysis of the APs in pHSA. Supplemental Tables: Table S1A: The protein and peptide identified in rHSA from company A. Table S2B: The protein and peptide identified in rHSA from company B. Table S3C: The protein and peptide identified in pHSA from company C. Table S4D: The protein and peptide identified in pHSA from company D. Table S5E: The protein and peptide identified in pHSA from company E. Table S6F: The protein and peptide identified in pHSA from company F. Table S7G: The protein and peptide identified in pHSA from company G. Table S8H: The protein and peptide identified in pHSA from company H. Table S9: The relative abunda [file peerj-13-19624-s002.zip › Supplementary/Supplementary File/Supplementary File2/3-Functional_enrichment/ident-kegg_dotplot.png]

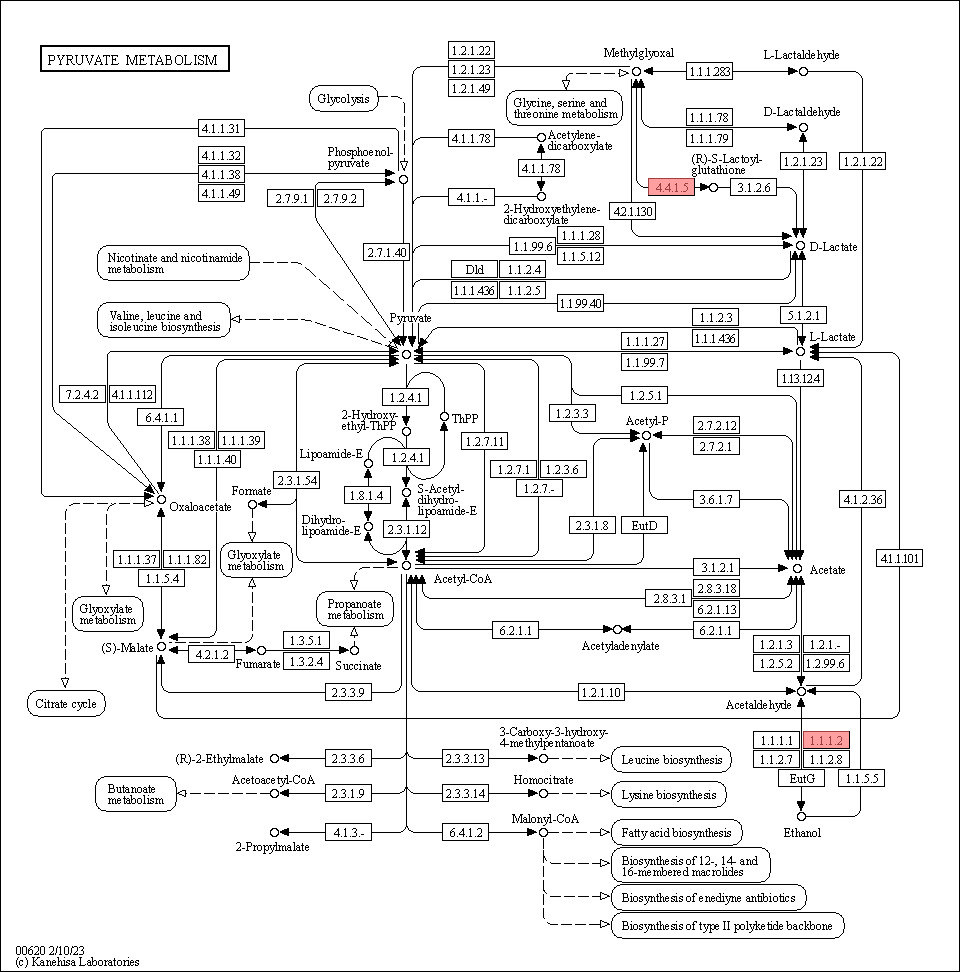

Supplement: Supplemental Information 2 — Supplemental Figures: Figure S1A: Peptide length, peptides per protein, distribution of coverage (%) and MW (kDa) of the LC-MS/MS analysis of rHSA from company A. Figure S2B: Peptide length, peptides per protein, distribution of coverage (%) and MW(kDa) of the LC-MS/MS analysis of rHSA from company B. Figure S3C: Peptide length, peptides per protein, distribution of coverage (%) and MW(kDa) of the LC-MS/MS analysis of pHSA from company C. Figure S4D: Peptide length, peptides per protein, distribution of coverage (%) and MW(kDa) of the LC-MS/MS analysis of pHSA from company D. Figure S5E: Peptide length, peptides per protein, distribution of coverage (%) and MW(kDa) of the LC-MS/MS analysis of pHSA from company E. Figure S6F: Peptide length, peptides per protein, distribution of coverage (%) and MW(kDa) of the LC-MS/MS analysis of pHSA from company F. Figure S7G: Peptide length, peptides per protein, distribution of coverage (%) and MW(kDa) of the LC-MS/MS analysis of pHSA from company G. Figure S8H: Peptide length, peptides per protein, distribution of coverage (%) and MW(kDa) of the LC-MS/MS analysis of pHSA from company H. Figure S9: GO enrichment analysis of the APs in pHSA. Figure S10: Subcellular localization prediction of the APs in pHSA. Figure S11: COG/KOG enrichment analysis of the APs in pHSA. Figure S12: KEGG pathway enrichment analysis of the APs in pHSA. Supplemental Tables: Table S1A: The protein and peptide identified in rHSA from company A. Table S2B: The protein and peptide identified in rHSA from company B. Table S3C: The protein and peptide identified in pHSA from company C. Table S4D: The protein and peptide identified in pHSA from company D. Table S5E: The protein and peptide identified in pHSA from company E. Table S6F: The protein and peptide identified in pHSA from company F. Table S7G: The protein and peptide identified in pHSA from company G. Table S8H: The protein and peptide identified in pHSA from company H. Table S9: The relative abunda [file peerj-13-19624-s002.zip › Supplementary/Supplementary File/Supplementary File2/4-Enrichment_pathway_image/image/map00620 2.png]

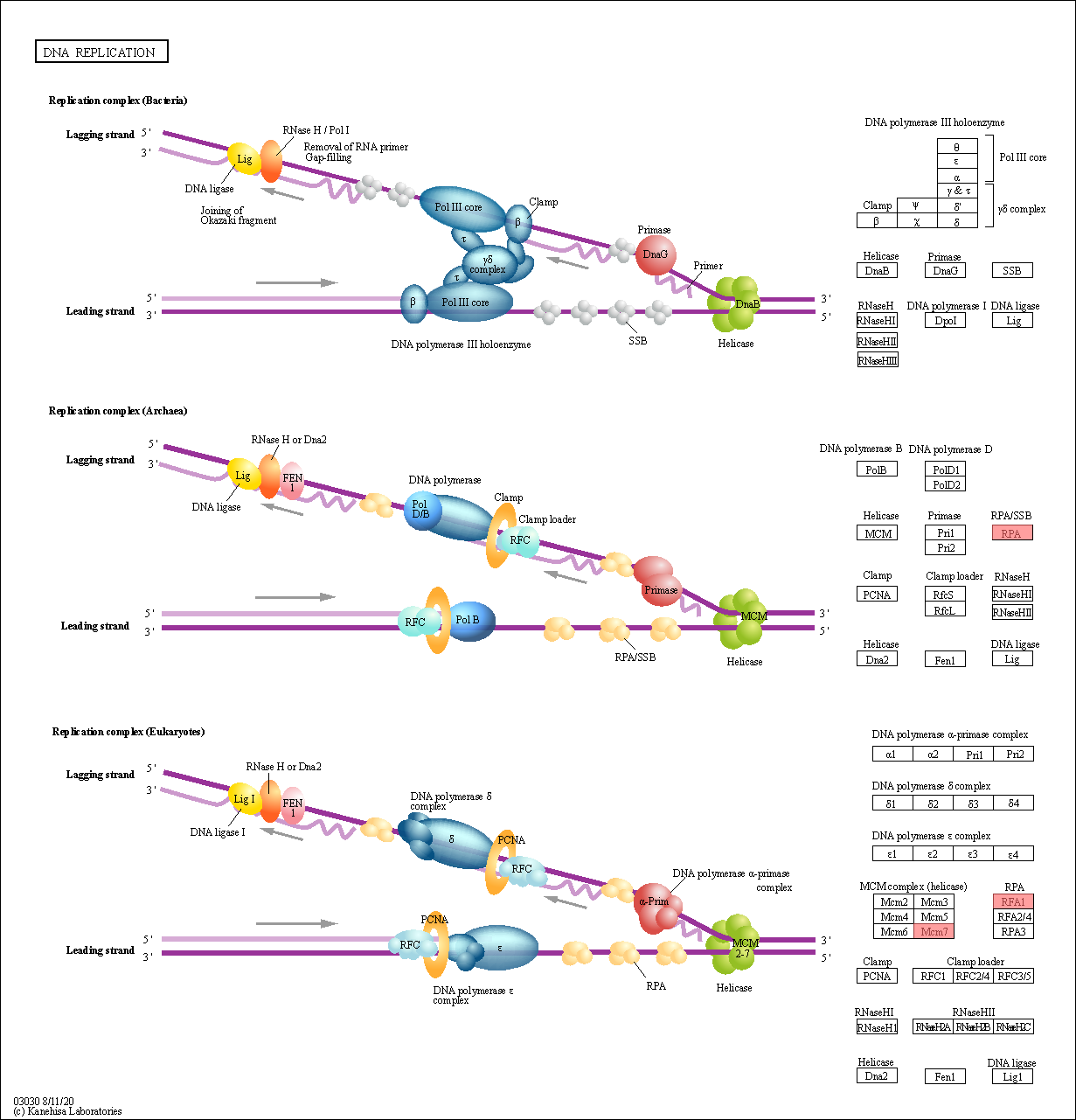

Supplement: Supplemental Information 2 — Supplemental Figures: Figure S1A: Peptide length, peptides per protein, distribution of coverage (%) and MW (kDa) of the LC-MS/MS analysis of rHSA from company A. Figure S2B: Peptide length, peptides per protein, distribution of coverage (%) and MW(kDa) of the LC-MS/MS analysis of rHSA from company B. Figure S3C: Peptide length, peptides per protein, distribution of coverage (%) and MW(kDa) of the LC-MS/MS analysis of pHSA from company C. Figure S4D: Peptide length, peptides per protein, distribution of coverage (%) and MW(kDa) of the LC-MS/MS analysis of pHSA from company D. Figure S5E: Peptide length, peptides per protein, distribution of coverage (%) and MW(kDa) of the LC-MS/MS analysis of pHSA from company E. Figure S6F: Peptide length, peptides per protein, distribution of coverage (%) and MW(kDa) of the LC-MS/MS analysis of pHSA from company F. Figure S7G: Peptide length, peptides per protein, distribution of coverage (%) and MW(kDa) of the LC-MS/MS analysis of pHSA from company G. Figure S8H: Peptide length, peptides per protein, distribution of coverage (%) and MW(kDa) of the LC-MS/MS analysis of pHSA from company H. Figure S9: GO enrichment analysis of the APs in pHSA. Figure S10: Subcellular localization prediction of the APs in pHSA. Figure S11: COG/KOG enrichment analysis of the APs in pHSA. Figure S12: KEGG pathway enrichment analysis of the APs in pHSA. Supplemental Tables: Table S1A: The protein and peptide identified in rHSA from company A. Table S2B: The protein and peptide identified in rHSA from company B. Table S3C: The protein and peptide identified in pHSA from company C. Table S4D: The protein and peptide identified in pHSA from company D. Table S5E: The protein and peptide identified in pHSA from company E. Table S6F: The protein and peptide identified in pHSA from company F. Table S7G: The protein and peptide identified in pHSA from company G. Table S8H: The protein and peptide identified in pHSA from company H. Table S9: The relative abunda [file peerj-13-19624-s002.zip › Supplementary/Supplementary File/Supplementary File2/4-Enrichment_pathway_image/image/map03030 2.png]

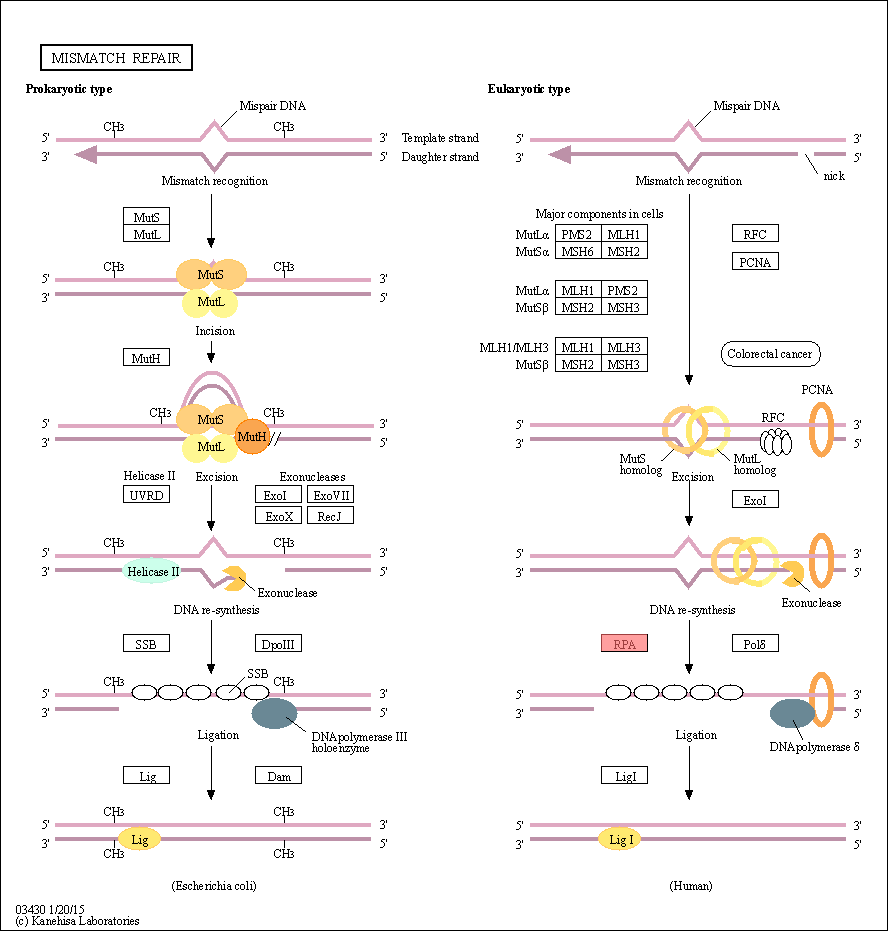

Supplement: Supplemental Information 2 — Supplemental Figures: Figure S1A: Peptide length, peptides per protein, distribution of coverage (%) and MW (kDa) of the LC-MS/MS analysis of rHSA from company A. Figure S2B: Peptide length, peptides per protein, distribution of coverage (%) and MW(kDa) of the LC-MS/MS analysis of rHSA from company B. Figure S3C: Peptide length, peptides per protein, distribution of coverage (%) and MW(kDa) of the LC-MS/MS analysis of pHSA from company C. Figure S4D: Peptide length, peptides per protein, distribution of coverage (%) and MW(kDa) of the LC-MS/MS analysis of pHSA from company D. Figure S5E: Peptide length, peptides per protein, distribution of coverage (%) and MW(kDa) of the LC-MS/MS analysis of pHSA from company E. Figure S6F: Peptide length, peptides per protein, distribution of coverage (%) and MW(kDa) of the LC-MS/MS analysis of pHSA from company F. Figure S7G: Peptide length, peptides per protein, distribution of coverage (%) and MW(kDa) of the LC-MS/MS analysis of pHSA from company G. Figure S8H: Peptide length, peptides per protein, distribution of coverage (%) and MW(kDa) of the LC-MS/MS analysis of pHSA from company H. Figure S9: GO enrichment analysis of the APs in pHSA. Figure S10: Subcellular localization prediction of the APs in pHSA. Figure S11: COG/KOG enrichment analysis of the APs in pHSA. Figure S12: KEGG pathway enrichment analysis of the APs in pHSA. Supplemental Tables: Table S1A: The protein and peptide identified in rHSA from company A. Table S2B: The protein and peptide identified in rHSA from company B. Table S3C: The protein and peptide identified in pHSA from company C. Table S4D: The protein and peptide identified in pHSA from company D. Table S5E: The protein and peptide identified in pHSA from company E. Table S6F: The protein and peptide identified in pHSA from company F. Table S7G: The protein and peptide identified in pHSA from company G. Table S8H: The protein and peptide identified in pHSA from company H. Table S9: The relative abunda [file peerj-13-19624-s002.zip › Supplementary/Supplementary File/Supplementary File2/4-Enrichment_pathway_image/image/map03430 2.png]

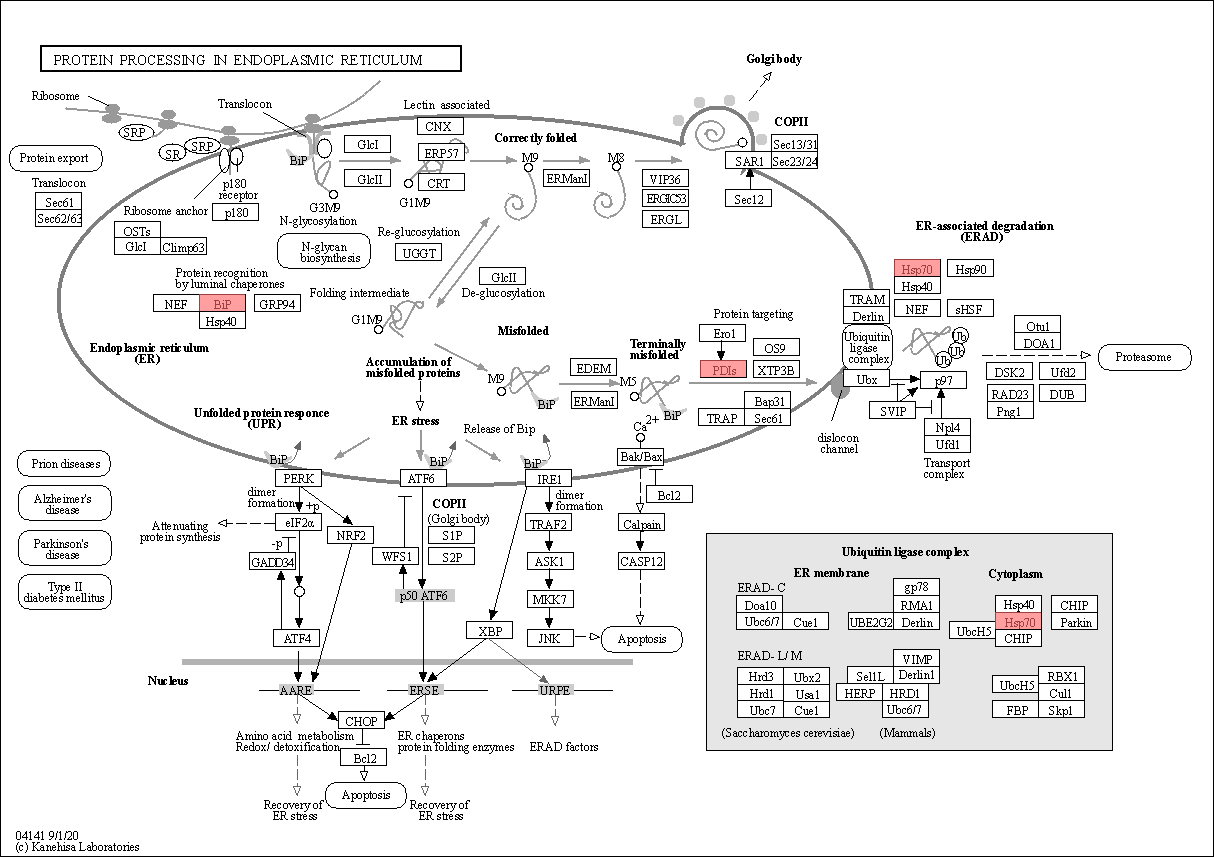

Supplement: Supplemental Information 2 — Supplemental Figures: Figure S1A: Peptide length, peptides per protein, distribution of coverage (%) and MW (kDa) of the LC-MS/MS analysis of rHSA from company A. Figure S2B: Peptide length, peptides per protein, distribution of coverage (%) and MW(kDa) of the LC-MS/MS analysis of rHSA from company B. Figure S3C: Peptide length, peptides per protein, distribution of coverage (%) and MW(kDa) of the LC-MS/MS analysis of pHSA from company C. Figure S4D: Peptide length, peptides per protein, distribution of coverage (%) and MW(kDa) of the LC-MS/MS analysis of pHSA from company D. Figure S5E: Peptide length, peptides per protein, distribution of coverage (%) and MW(kDa) of the LC-MS/MS analysis of pHSA from company E. Figure S6F: Peptide length, peptides per protein, distribution of coverage (%) and MW(kDa) of the LC-MS/MS analysis of pHSA from company F. Figure S7G: Peptide length, peptides per protein, distribution of coverage (%) and MW(kDa) of the LC-MS/MS analysis of pHSA from company G. Figure S8H: Peptide length, peptides per protein, distribution of coverage (%) and MW(kDa) of the LC-MS/MS analysis of pHSA from company H. Figure S9: GO enrichment analysis of the APs in pHSA. Figure S10: Subcellular localization prediction of the APs in pHSA. Figure S11: COG/KOG enrichment analysis of the APs in pHSA. Figure S12: KEGG pathway enrichment analysis of the APs in pHSA. Supplemental Tables: Table S1A: The protein and peptide identified in rHSA from company A. Table S2B: The protein and peptide identified in rHSA from company B. Table S3C: The protein and peptide identified in pHSA from company C. Table S4D: The protein and peptide identified in pHSA from company D. Table S5E: The protein and peptide identified in pHSA from company E. Table S6F: The protein and peptide identified in pHSA from company F. Table S7G: The protein and peptide identified in pHSA from company G. Table S8H: The protein and peptide identified in pHSA from company H. Table S9: The relative abunda [file peerj-13-19624-s002.zip › Supplementary/Supplementary File/Supplementary File2/4-Enrichment_pathway_image/image/map04141 2.png]

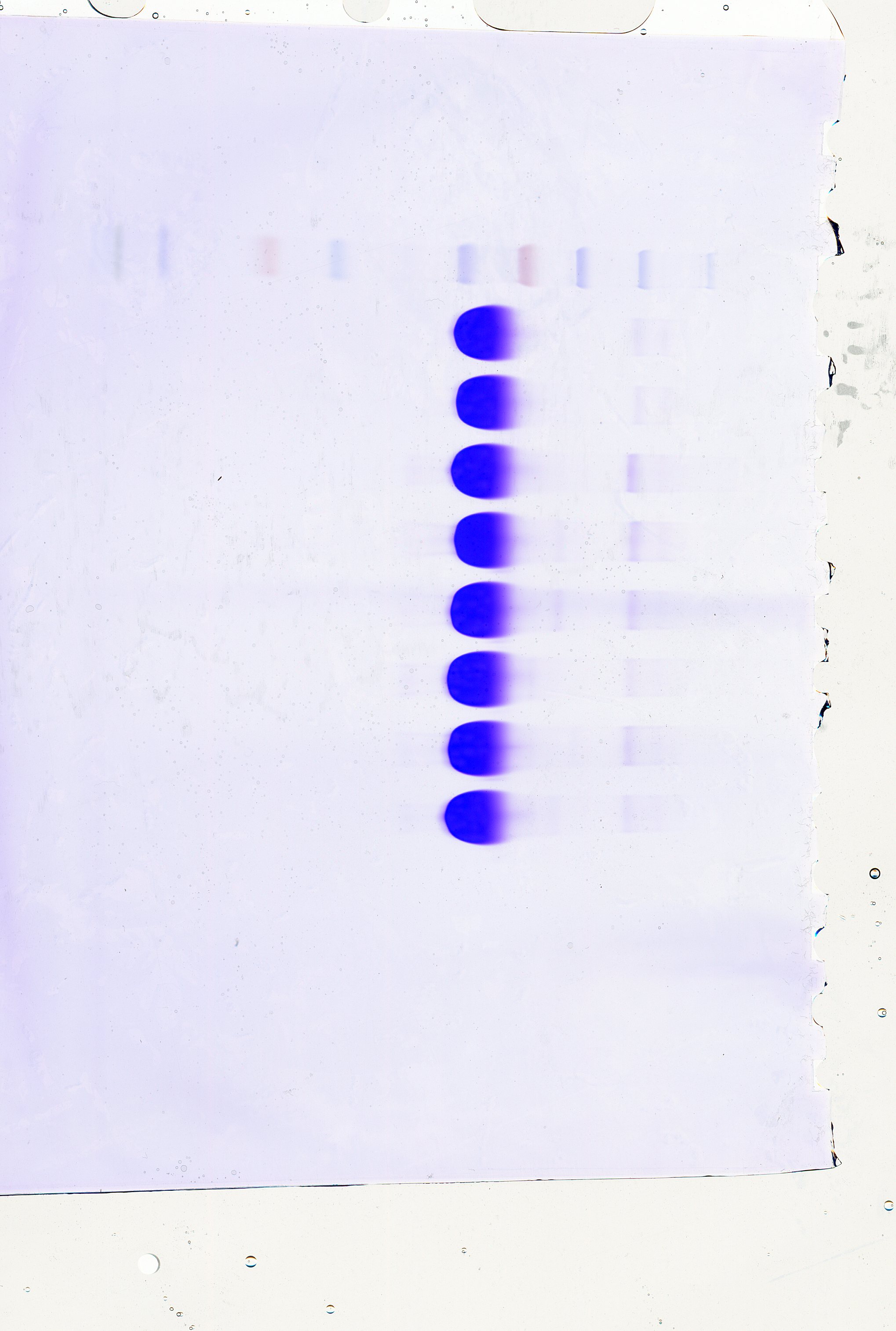

Supplement: Supplemental Information 4 [file peerj-13-19624-s004.jpg]
