# Supplementary material for: Protein composition analysis of human plasma-derived and recombinant human serum albumin preparations based on 4D label-free proteomics
Source: PeerJ. 2025 Jun 30;13:e19624. doi: 10.7717/peerj.19624 (PMC12225626; doi:10.7717/peerj.19624)
Supplement: Supplemental Information 5 [file peerj-13-19624-s005.docx]

Figure Legend

Figure S1 A: Peptide length, peptides per protein, distribution of coverage (%) and MW(kDa) of the LC-MS/MS analysis of rHSA from company A.

Figure S2 B: Peptide length, peptides per protein, distribution of coverage (%) and MW(kDa) of the LC-MS/MS analysis of rHSA from company B.

Figure S3 C: Peptide length, peptides per protein, distribution of coverage (%) and MW(kDa) of the LC-MS/MS analysis of pHSA from company C.

Figure S4 D: Peptide length, peptides per protein, distribution of coverage (%) and MW(kDa) of the LC-MS/MS analysis of pHSA from company D.

Figure S5 E: Peptide length, peptides per protein, distribution of coverage (%) and MW(kDa) of the LC-MS/MS analysis of pHSA from company E.

Figure S6 F: Peptide length, peptides per protein, distribution of coverage (%) and MW(kDa) of the LC-MS/MS analysis of pHSA from company F.

Figure S7 G: Peptide length, peptides per protein, distribution of coverage (%) and MW(kDa) of the LC-MS/MS analysis of pHSA from company G.

Figure S8 H: Peptide length, peptides per protein, distribution of coverage (%) and MW(kDa) of the LC-MS/MS analysis of pHSA from company H.

Figure S9: GO enrichment analysis of the APs in pHSA.

Figure S10: Subcellular localization prediction of the APs in pHSA.

Figure S11: COG/KOG enrichment analysis of the APs in pHSA.

Figure S12: KEGG pathway enrichment analysis of the APs in pHSA.

Table Legend

Table S1 A: The protein and peptide identified in rHSA from company A.

Table S2 B: The protein and peptide identified in rHSA from company B.

Table S3 C: The protein and peptide identified in pHSA from company C.

Table S4 D: The protein and peptide identified in pHSA from company D.

Table S5 E: The protein and peptide identified in pHSA from company E.

Table S6 F: The protein and peptide identified in pHSA from company F.

Table S7 G: The protein and peptide identified in pHSA from company G.

Table S8 H: The protein and peptide identified in pHSA from company H.

Table S9: The relative abundance and protein score of all APs in each sample.

Table S10: Protein annotation of the identified APs in pHSA.

Table S11: GO enrichment of the identified APs in pHSA.

Table S12: Protein domain enrichment of the identified APs in pHSA.

Table S13: KEEG enrichment of the identified APs in pHSA.

Table S14: Reactome pathways analysis of the identified APs in pHSA.

Table S15: WikiPathway analysis of the identified APs in pHSA.

Table S16: The nodes of PPI proteomic network analysis with STRING database of the identified APs in pHSA.

Table S17: The links of PPI proteomic network analysis with STRING database of the identified APs in pHSA.

Table S18: KEEG classification of the identified APs in pHSA.

Table S19: COG classification of the identified APs in pHSA.
